# Sex difference in EGFR pathways in mouse kidney-potential impact on the immune system

**DOI:** 10.1186/s12863-016-0449-3

**Published:** 2016-11-24

**Authors:** Fengxia Liu, Yan Jiao, Yun Jiao, Franklin Garcia-Godoy, Weikuan Gu, Qingyi Liu

**Affiliations:** 1 The Fourth Hospital, Hebei Medical University, Shijiazhuang, Hebei 050011 China; 2 Department of Orthopaedic Surgery and BME-Campbell Clinic, University of Tennessee Health Science Center, Memphis, TN USA; 3 Department of Neuroscience, St Jude Children’s Research Hospital, Memphis, TN USA; 4 Bioscience Research Center, College of Dentistry, University of Tennessee Health Science Center, 875 Union Avenue, Memphis, TN USA; 5 Research Service, Veterans Affairs Medical Center, 1030 Jefferson Avenue, Memphis, TN USA; 6 956 Court Ave, Memphis, TN 38163 USA

**Keywords:** Drug, *Egfr*, Mice, Pathway, Sex

## Abstract

**Background:**

Epidermal growth factor receptor (*Egfr*) has been the target of several drugs for cancers. The potential gender differences in genes in the Egfr axis have been suggested in humans and in animal models. Female and male mice from the same recombinant inbred (RI) strain have the same genomic components except the sex difference. A population of different RI mouse strains allows to conduct precise analysis of molecular pathways and regulation of *Egfr* between female and male mice.

**Methods:**

The whole genome expression profiles of 70 genetically diverse RI strains of mice were used to compare three major molecular aspects of *Egfr* gene: the relative expression levels, gene network and expression quantitative trait loci (eQTL) that regulate the expression of *Egfr* between female and male mice.

**Results:**

Our data showed that there is a significant sex difference in the expression levels in kidney. A considerable number of genes in the gene network of *Egfr* are sex differentially expressed. The expression levels of *Egfr* in mice are statistical significant different between C57BL/6 J (B6) and DBA/2 J (D2) genotypes in male while no difference in female mice. The eQTLs that regulate the expression levels of *Egfr* between female and male mice are also different. Furthermore, the differential expression levels of *Egfr* showed significantly different correlations with two known biological traits between male and female mice.

**Conclusion:**

Overall there is a substantial sex difference in the *Egfr* pathways in mice. These data may have significant impact on drug target design, development, formulation, and dosage determinant for women and men in clinical trials.

**Electronic supplementary material:**

The online version of this article (doi:10.1186/s12863-016-0449-3) contains supplementary material, which is available to authorized users.

## Background

The purpose of this work is to systematically investigate the sex differences of epidermal growth factor receptor (*Egfr*) in the kidney using high quality data from a population of mouse recombinant inbred (RI) strains. Sex differences have been brought to the attention of the public and health research community [1–3], and sex disparities in health throughout the lifespan of humans or mice have been documented [2, 3].

In humans, EGFR has been reported as an important player in pathways of kidney diseases. Liang et al. reported the inhibitory effect of silibinin on EGFR signal-induced renal cell carcinoma progression via suppression of the EGFR/MMP-9 signaling pathway [4]. The effect of EGR on renal cell carcinoma was also reported by others [5, 6]. EGFR activation is required to induce the renal fibrotic genes [7, 8]. EGFR has been received great attention in cancer research because of its connection to cancer development [9, 10]. Drugs targeting the EGFR pathway have been developed in recent years, and some clinic trials are underway. For example, Erlotinib, an EGFR and tyrosine kinase inhibitor, have been used for the treatment of kidney, lung, advanced adenocarcinoma of the oesophagus and gastro-oesophageal junction and hepatocellular carcinoma cancers [11, 12]. Lapatinib ditosylate (LAP), an anti-EGFR drug, has been used for treatment of gastro-oesophageal cancer and renal cancer [13, 14]. Ramucirumab has been used as monotherapy for previously treated advanced gastric or gastro-oesophageal junction adenocarcinoma [15–18]. While the drugs based on anti EGFR are in development and clinical trial stage, its gender specificity should be thoroughly investigated.

The potential gender differences in genes in the Egfr axis have been suggested in humans and in animal models [19–27]; however, the gender specificity on the molecular pathways has yet to be understood. At present, a systematic investigation of sex specificities in the Egfr axis, either in humans or animal models, has not been reported. Recently, substantial progress has been made in elucidating how diverse sex specific systems are integrated into developmental gene networks using animal models. In particular, the RI strains derived from C57BL/6 J (B6) X DBA/2 J (D2) have been used for studies of sex specific traits and genetic regulations [20–22]. Sex specific gene expressions profiling have been analyzed for the liver and other tissues [23–25]. Therefore, we believe that our optimized approach discloses information on sex differences in the *Egfr* axis that will eventually impact the management of all drug design and clinical trials in many diseases including cancer.

Several recent publications showed the sex specificity [20, 21, 24, 26] and tissue specificity [25, 27, 28] of gene expression levels or the association of gene expressions. Recent study indicated that the relative expression levels, gene network and eQTLs that regulate the expression of Egfr in liver are different between female and male mice [29]. We hypothesize that at least one step in the *Egfr* molecular pathway in kidney of the male is different from that of the female mice. We first investigated whether the expression levels of *Egfr* in the kidney is different between female and male mice. We then determined whether the gene network of *Egfr* between female and male mice are the same. We finally examined the genetic loci that regulate the expression of *Egfr* in females and males.

## Methods

### Expression levels of *Egfr*

For the expression data of *Egfr* and its associated genes, we collected the expression data of Egfr axis from whole genome expression data of the mouse kidney [29]. All data are from GeneNetwork (http://www.genenetwork.org/webqtl/main.py) and are available to the public. We used the *Actin beta (B)* as controls for the expression level of *Egfr*. The data set of gene expression profiles of mouse kidney at GeneNetwork were validated using sex specific probe sets such as X inactivation-specific transcript (*Xist*) and Dead/h box 3, y-linked (*Dby*) [29]. When multiple probes are presented for a gene, the probe with the highest expression level was chosen for the analysis while the others were used as references.

### Data set for analysis of gene expression profiles

In this study, we used the Mouse Kidney M430v2 female and male (Aug06) RMA Database from the GeneNetwork (http://www.genenetwork.org/webqtl/main.py). RMA stands for statistical method of robust multi-array average for the raw data analysis [21] The data set includes mRNA expression in the adult kidney of both sexes of 70 genetically diverse strains of mice including 54 BXD RI strains, a set of 15 inbred strains, and 1 F1 hybrid: D2B6F1. Kidney samples were processed using a total of 153 Affymetrix Mouse Expression 430 2.0 arrays. Kidneys from two to six animals per strain were pooled. Forty-two (31 BXD, D2B6F1 and 10 inbred strains) are represented by male and female samples [21].

While samples of both sexes are collected at similar age, performed with same microarray platform, and grown in the same environment, most of them are from different RI strains. However, these RI strains are all derived from the same two progenitors. Also, the gene expression profiles of both the female and male progenitors are generated separately.

### The association of the expression levels of Egfr between female and male mouse populations

The association analysis was conducted using the method as previously reported [23, 29, 30]. R values were compared between female and male mice, following standard criteria for the strong, correlation, and none correlation [29]. Unless noted in the figure legends, when the R value was equal or more than 0.7 or −0.7, the correlation was regarded as strong positive or negative. When the R value was between 0.50 and 0.69 or −0.50 and −0.69, the correlation existed but was not strong. When the R value was between 0.3 and 0.49 or −0.30 and −0.49, the correlation was weak. Any R value between 0 and 0.29 or 0 and −0.29 was regarded as none-correlation [29, 30].

### Gene network construction

The gene networks were constructed using application tools in GeneNetwork. We constructed the gene network based on the Network Graph in combination with the Correlation Matrix [29]. For the Correlation Matrix, the Pearson product-moment correlations (the standard type of correlation) were used for the calculation of correlations [30, 31]. For each sex in each pair of samples, both the Network Graph and Correlation Matrix were obtained with the same set of parameters or criteria. For example, for the Line Threshold in the Network Graph, absolute values greater than 0.30 were used across all samples. The Spring Model layout (force reduction) was used for the graphic method for all graphic samples.

### Transcriptomic loci (eQTL) that regulates the expression level of *Egfr* in female and male mice

Transcriptome mapping with GeneNetwork was used to identify the chromosomal regions that affect the expression of *Egfr* in female and male mice, which includes three major steps. First, *Egfr* probes of gene expression were identified from female and male strains of tissues. Second, interval mapping was done to establish *Egfr* transcriptome maps for the entire genome. Permutations of 5000 tests were used to assess the strength and consistency of the linkages. Third, genomic regions and locations on chromosomes were compared [30–32].

## Results

### Expression levels of *Egfr* between female and male in mouse kidney

**Fig. 1:**
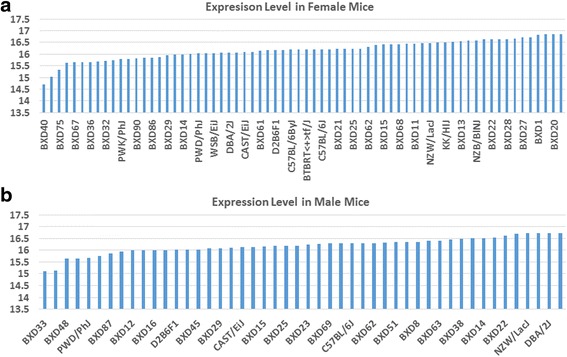
Expression levels of *Actin B* in kidney between female and male mice in BXD strains. Numbers on *left vertical bar* are for the relative levels of expression of *Actin B.* The information of strains and names of standard inbred strains are listed under the *horizontal bar*. **a**. The expression levels of *Actin B* in kidney of female mice. **b**. The expression levels of *Actin B* in kidney of male mice

Basical statistical analysis was conducted with data of gene expression of Actin B and *Egfr* in both sexes from a total of 43 strains [29]. Five probes for *Actin B* on the Affymetrix Mouse Genome 430 2.0 array chip was identified. The Probe of 1436722_a_at was chosen for the analysis because its high expression level and it contains exon 3, 4, 5 and proximal 3′ UTR. Figure 1a and b showed the expression levels of Actin A in female and male mice of different strains. The *P* value from the T test between female and male is 0.55 and the R value from correlation analysis is 0.25.

**Fig. 2:**
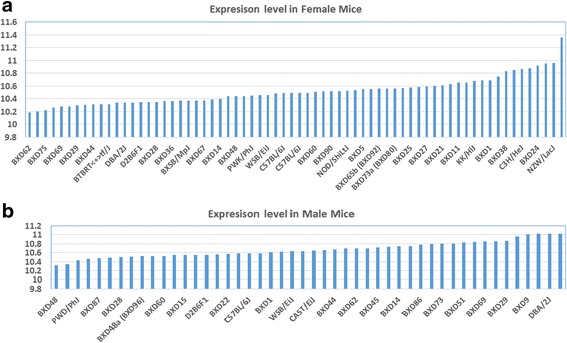
Expression levels of *Egfr* in kidney between female and male mice in BXD strains. Numbers on *left vertical bar* are for the relative levels of expression of *Egfr*. The information of strains and names of standard inbred strains are listed under the *horizontal bar*. **a**. The expression levels of *Egfr* in kidney of female mice. **b**. The expression levels of *Egfr* in kidney of male mice

Seven probes for *Egfr* were found from data from the Affymetrix Mouse Genome 430 2.0 array chip. The Probe 1460420_a_at was chosen for the analysis because its high expression level. Figure 2a and b shows the expression levels of *Egfr* in female and male mice of different strains. For *Egfr*, the P value from T test between female and male mice is 0.00 and the R value is −0.05.

Thus, there was no sex difference in the expression level of *Actin B* in kidney while there was a significant sex difference in the *Egfr* expression levels in these mouse strains.

### Gene network of *Egfr* between female and male in mouse kidney

With Probe 1460420, the top 50 probes of genes with expression levels most correlated to that of *Egfr* were identified from the mouse data of Mouse kidney M430v2 Male (Aug06) RMA Database. These probes represent genes with diversity of biological functions (Additional file 1: Table S1).

**Fig. 3:**
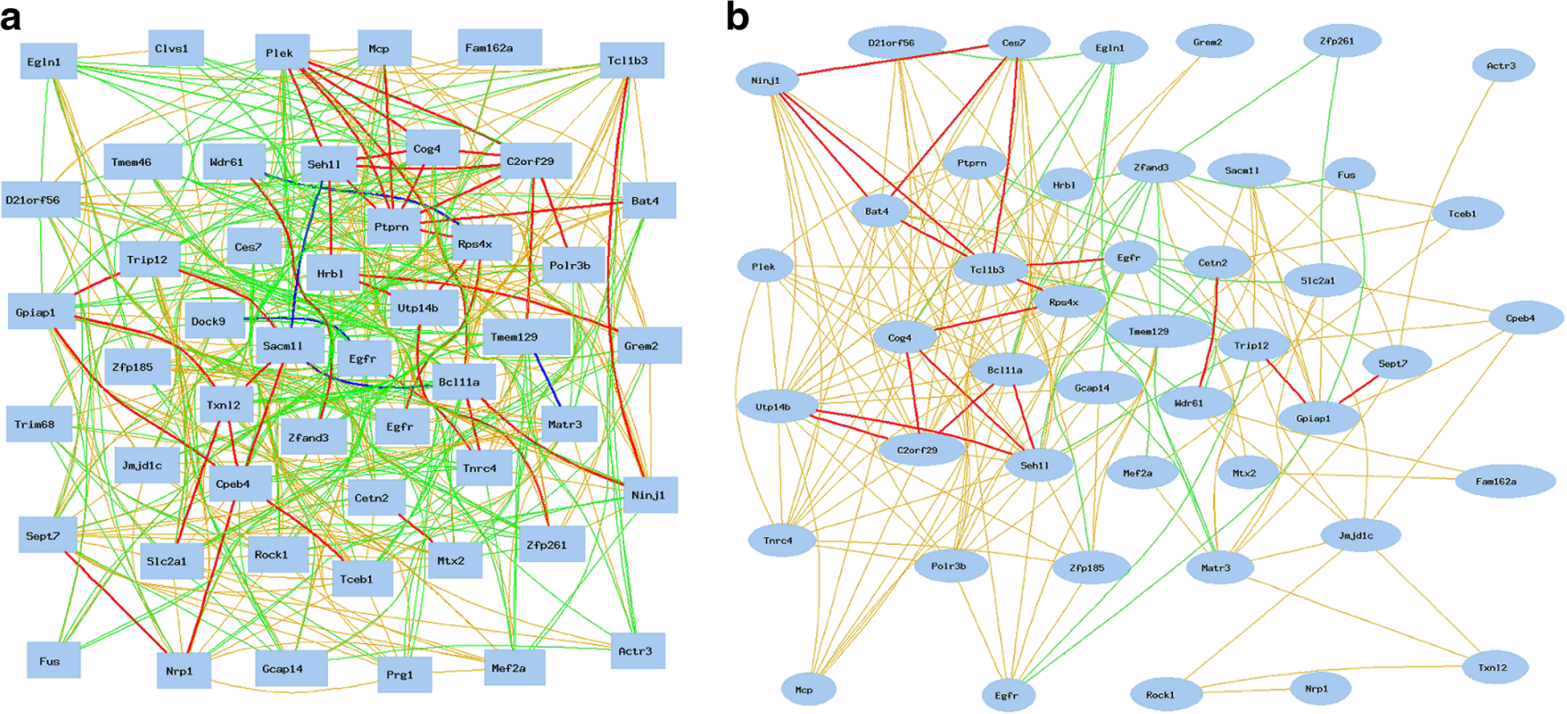
Gene network of *Egfr* in mouse Kidney. The 50 nodes in the graph below show the selected traits. Only nodes with edges are displayed. The 208 edges between the nodes, filtered from the 1225 total edges and drawn as curves, show Pearson correlation coefficients greater than 0.5 or less than −0.5. The graph’s canvas is 40.0 by 40.0 cm, and the node labels are drawn with a 16.0 point font, and the edge labels are drawn with a 16.0 point font. **a**. Gene network of top 50 genes that closely associated to *Egfr* in male mouse kidney. **b**. Gene network of male top 50 genes that closely associated to *Egfr* in female mouse kidney

As shown in Fig. 3a, *Egfr* is either positively or negatively correlated to the top 50 probes of genes. The expression of mouse *Egfr* is strongly positively correlated to ribosomal protein S4, X-linked (*Rps4x*) and zinc inger protein 261 (*Zfp261*); it is strongly negatively correlated to dedicator of cytokinesis 9 (*Dock9*). In order to compare the correlation of these genes to *Egfr* in female mice, we then constructed a gene network using the same probes from female gene expression profiles of the kidney (Fig. 3b).

Several differences in the gene network of the kidney were found between female and male mice. In males, *Egfr* showed a strong negative correlation with *Dock9* but in females, such a negative correlation did not appear. In males, *Egfr* showed a negative correlation with clavesin 1 (retinaldehyde binding protein 1-like 1) (*Clvs1*); HIF1 alpha hypoxia growth and transformation-dependent protein, proapoptotic (*Fam162a*); neuropilin 1 (*Nrp1*); proteoglycan 1, secretory granule (*Prg1*); septic 7 (cell division cycle 10 homolog) (*Sept7*); transcription elongation factor B (SIII), polypeptide 1 (*Tceb1*); and transmembrane protein 46 (*Tmem46*). However, in female mice, these negative correlations did not show up. In addition, in male mice, *Egfr* showed a positive correlation with membrane cofactor protein (*Mcp*); pleckstrin associated EST AK008484 (*Plek*); and trinucleotide repeat containing 4 (*Tnrc4*), although this positive correlation did not show up in female mice.

### Confirmation of sex difference in kidney with multiple probes

The above results are mainly based on *Egfr* probe 1460420. There were seven probes of *Egfr* on the Affymetrix Mouse Genome 430 2.0 array chip. In order to find out whether these sex differences are caused by the bias in one probe, we examined correlations between all the *Egfr* probes and these groups of sex different genes.

**Fig. 4:**
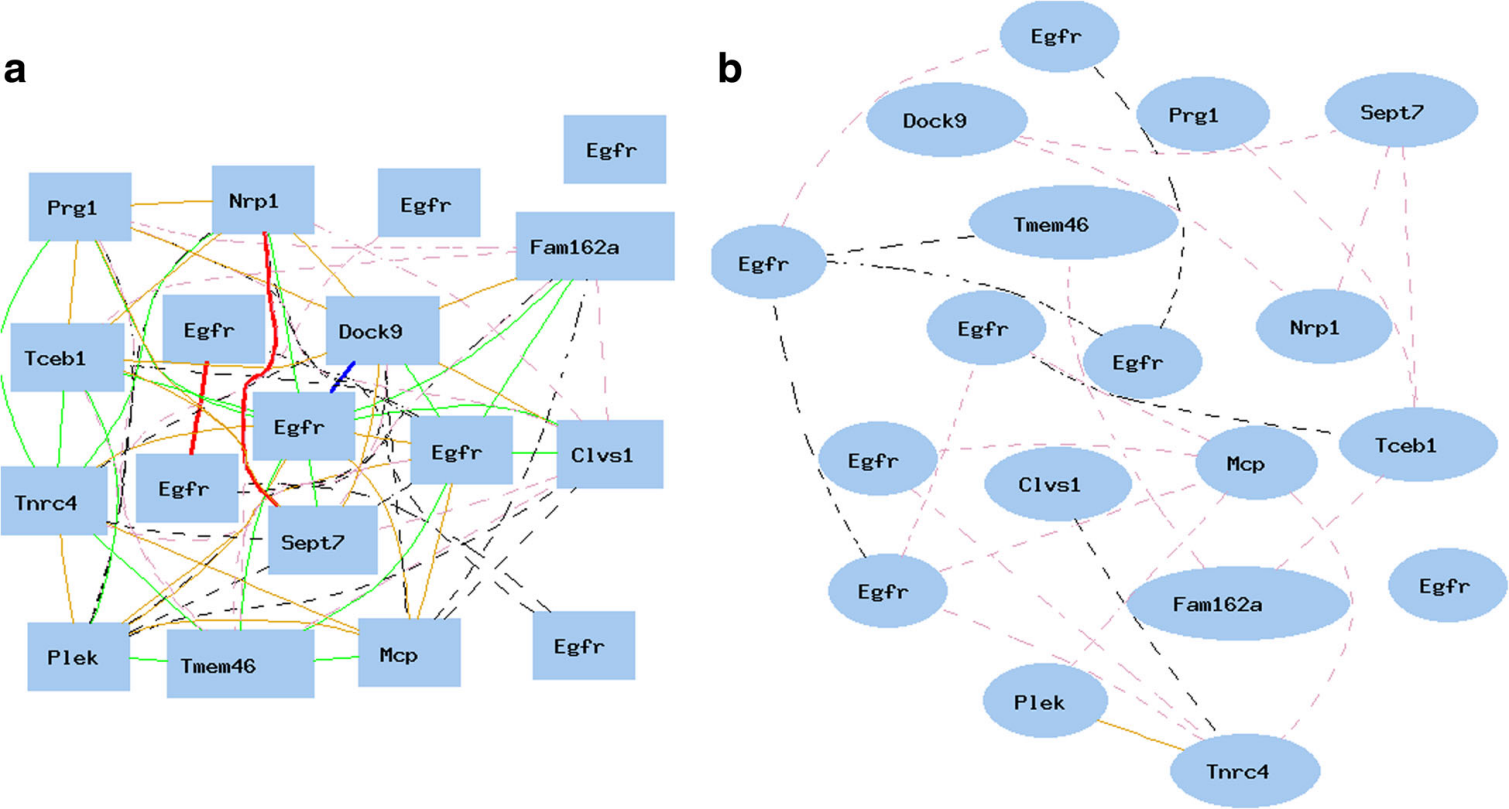
Confirmation of negative and positive correlations between *Egfr* and key genes showing sex difference. The 18 nodes in the graph below show the selected traits. The graph’s canvas is 40.0 by 40.0 cm, and the node labels are drawn with a 16.0 point font, and the edge labels are drawn with a 16.0 point font. *Curves* show Pearson correlation coefficients greater than 0.3 or less than −0.3. **a**. Correlations between *Egfr* and key genes in kidney of male mice. All nodes are displayed. The 73 edges between the nodes, filtered from the 153 total edges and drawn as curves. **b**. Correlations between *Egfr* and key genes in kidney of female mice. Lack of positive and negative correlations between *Egfr* probes and 11 key genes. All nodes are displayed. The 23 edges between the nodes, filtered from the 153 total edges and drawn as curves

We first obtained the data of all seven *Egfr* probes and probes of all 11 genes that showed positive or negative correlations with *Egfr* from kidney of male strains, and then constructed the gene network for all of them. As shown in Fig. 4a, three positive regulated genes (*Mcp, Plek, Tnrc4*) were all being connected positively to at least one of the *Egfr* probes, while eight negatively regulated genes (*Dock9, Clvs1, Fam162a, Nrp1, Prg1, Sept7, Tceb1, Tmem46*) were negatively connected to at least one of the *Egfr* probes.

We next obtained the data of all seven *Egfr* probes and probes of all above 11 genes from kidneys of female mouse strains. Figure 4b showed that there was no significant connection between these 11 genes to any probe of *Egfr*. The R values were all under 0.3, at a non-significant level. These data confirm the significant difference in the molecular network of *Egfr* between female and male mice.

### EQTL regulation of *Egfr* between female and male mice

**Fig. 5:**
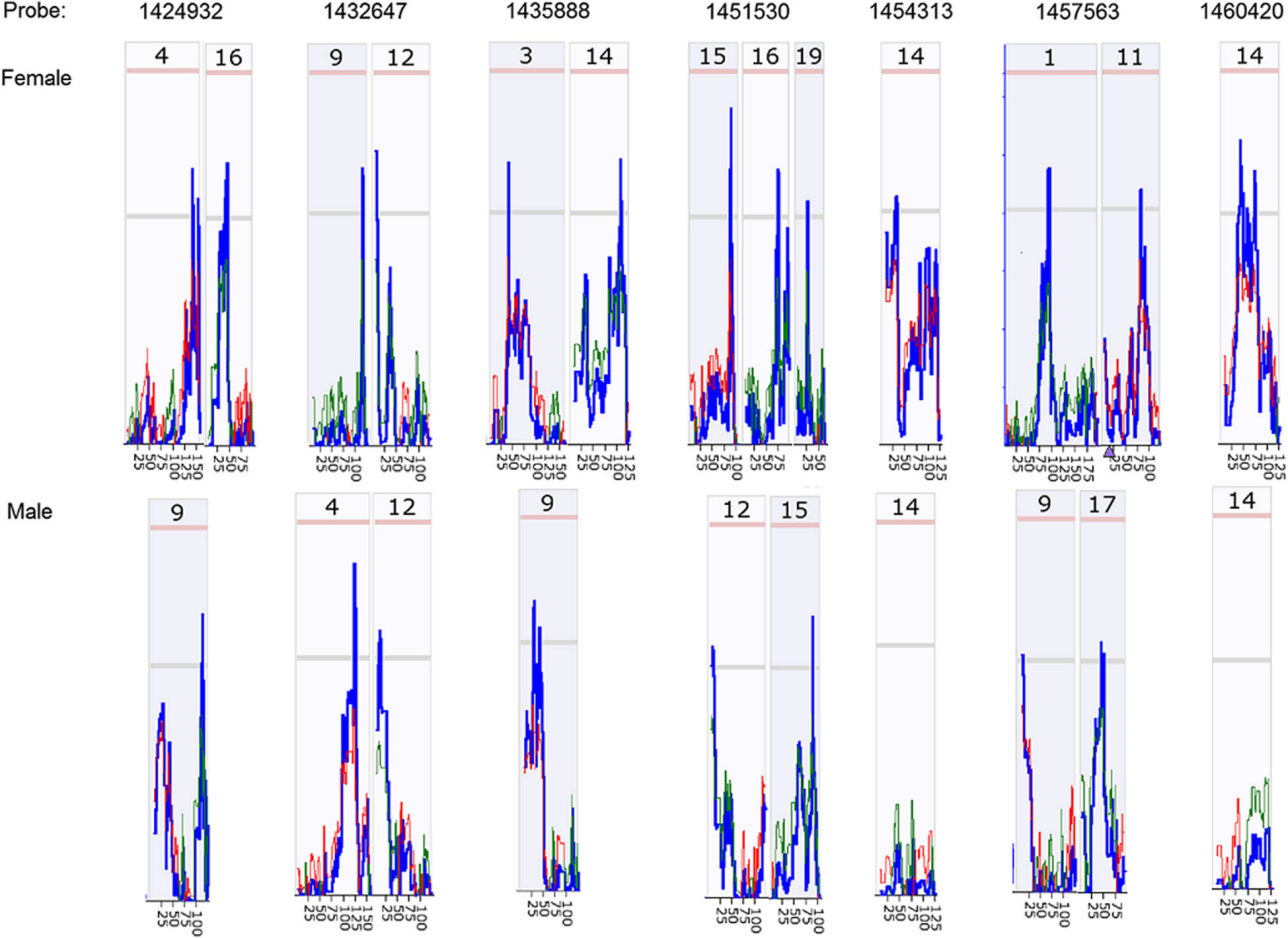
Detection of eQTL for regulation of *Egfr* expression levels in kidney of mouse RI strains using probes of *Egfr. Top line* is the numbers of *Egfr* probes, *second line* is the eQTL detected from female mice. *Bottom line* shows the eQTL detected from male mice. The numbers on top of each individual figure in second and bottom lines indicate the number of chromosome. *Pink color* lines on top of the individual figure indicate the threshold for significant level. Light grey lines indicate the threshold for suggestive level

The eQTLs of all seven probes of *Egfr* in female and male mice were identififed. Figure 5 shows the eQTL mapped from each probe in female and in male mice. The eQTL of these seven probes of *Egfr* in kidneys of female mice were mapped on to chromosomes 1, 3, 9, 11, 12. 14, 15, 16 and 19. The probe, 1460420_a_at with the highest expression level, was mapped on chromosome 14. The other probe, 1451530_ at, mapped an eQTL on chromosome 15, with the highest LRS score of 15.6. We then found eQTLs for the regulation of *Egfr* from the male kidney from these seven probes. The eQTL from these probes were on chromosome 4, 9, 12, 14, 15, 17 and 19. The QTL with highest LRS value is located on chromosome 4, which is 14.2 (Fig. 5).

Although there were overlaps on some chromosomes of eQTL between female and male mice, the locations of most of these eQTL are different between sexes. The eQTLs from three probes were all different from each other between female and male mice. Mapping with probe 1424932_at, eQTLs were detected from chromosome 4 and 16 from female, while the eQTL was located on chromosome 9 from the male. Mapping with probe 1435888_at, eQTLs were detected from chromosome 3 and 14 from female, while the eQTL was located on chromosome 9 from the male. Mapping with probe 1457563_at, eQTLs were detected from chromosome 1 and 11 from female, while the eQTLs were mapped on chromosome 9 and 17 from the male. The other two probes each detected one overlapped eQTL and other non-overlapped eQTLs between female and male. Mapping with probe 1432647_at, eQTLs were detected from chromosomes 9 and 12 from female, while the eQTLs were mapped on chromosomes 4 and 12 from the male. Mapping with probe 1451530_at, eQTLs were detected from chromosome 12, 15, and 19 from females, while the eQTL were mapped on chromosomes 12 and 15 from the males. The final two probes had eQTL from female but not from the male mice. Mapping with probes 1454313_at and 1460420_a_at an eQTL was detected on chromosome 14, for female for both probes, while no eQTL was mapped from the male mice. Overall, the difference is much larger than the similarity.

### Potential candidate for the *Egfr* expression level in male mice on chromosome 4

**Fig. 6:**
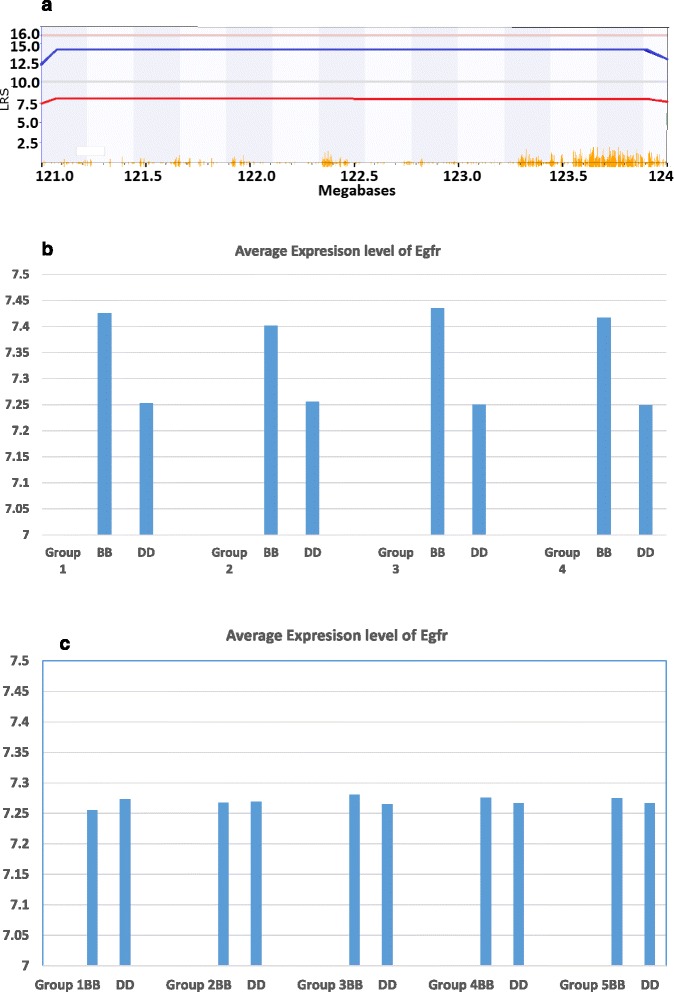
Comparison of level of E*gfr* expression of different genotypes in peak region of eQTL on chromosome 4. **a**. Location of peak region of eQTL of *Egfr* in male mice. *Pink color lines* on top of the individual figure indicate the threshold for significant level. *Light grey lines* indicate the threshold for suggestive level. Numbers on the Y bar are the LRS scores. Numbers on the X bar are the position on the chromosome measured by Mb. **b**. The average expression levels of *Egfr* in male mice with BB and DD genotypes. Numbers on the *Y bar* are the relative expression levels of *Egfr. X bar* indicates the groups of SNP and their genotypes. * *P* value is less than 0.05 but larger than 0.01. ** *P* values are less than 0.01. **c**. The average expression levels of *Egfr* in female mice with BB and DD genotypes. Numbers on the *Y bar* are the relative expression levels of *Egfr. X bar* indicates the groups of SNP and their genotypes

According to the map based on probe #1432647, the peak region of the eQTL on chromosome 4 is between 120.6 Mb and 124 Mb (Fig. 6a). Within the region, 35 genetic elements, including 25 known genes, exist. We further examined the known SNP in this region among BXD strains. Within the region, we find 13 polymorphic markers between B6 and DBA/1. They are divided into 4 haploid groups. The first one is the SNP rs4224744, which is located on 120.476225. The second group include rs3675629 and gnf04.116.914, which located between 120.731447 Mb and 120.875373 Mb. The third group includes 7 markers, gnf04.117.102, CEL-4_120039566, UT_4_121.927901, gnf04.119.329, rs13477959, and rs3714811, which is located between 121.069747 Mb and 124.048406 Mb. The fourth group includes three markers, rs3677161, rs13474356 and rs3704486, which is located between 124.517595 Mb and 125.265631 Mb. We then compared the expression level of *Egfr* with B (B6) and D (DBA/1) genotypes in male mice of each group of haploid types. The P values of T-test for each of these four groups are 0.062186655, 0.035691806, 0.007229499, and 0.009439615, respectively (Fig. 6b). Thus, the candidate genes are most likely located within the region covered by the polymorphic markers in group 3. Within this region, there are 29 genetic elements, Including 18 known genes. These genes are *Ppt1, Cap1, Mfsd2, Mycl1, Trit1, Bmp8b, Oxct2b, Ppie, Hpcal4, Nt5c1a, Heyl, Pabpc4, Bmp8a, Oxct2a, Macf1, Ndufs5, Rhbdl2, Mycbp* and *Rragc*. We next examined the relationship of expression level between these candidate genes and that of *Egfr*. No expression level of these genes showed a strong correlation to that of *Egfr*. In order to examine whether this eQTL is male specific, we examined the *Egfr* expression of B and D genotypes in female. Our data showed that the P values between the B and D genotypes in different haploid groups varied from 0.869525556 to 0.978478759 (Fig. 6c). Thus, there is no difference in the *Egfr* expression level between the B and D genotypes in female mice. These data confirm the difference in the regulation of expression levels of *Egfr* between female and male mice.

### Potential impact of sex difference in *Egfr* expression on immunological traits

**Fig. 7:**
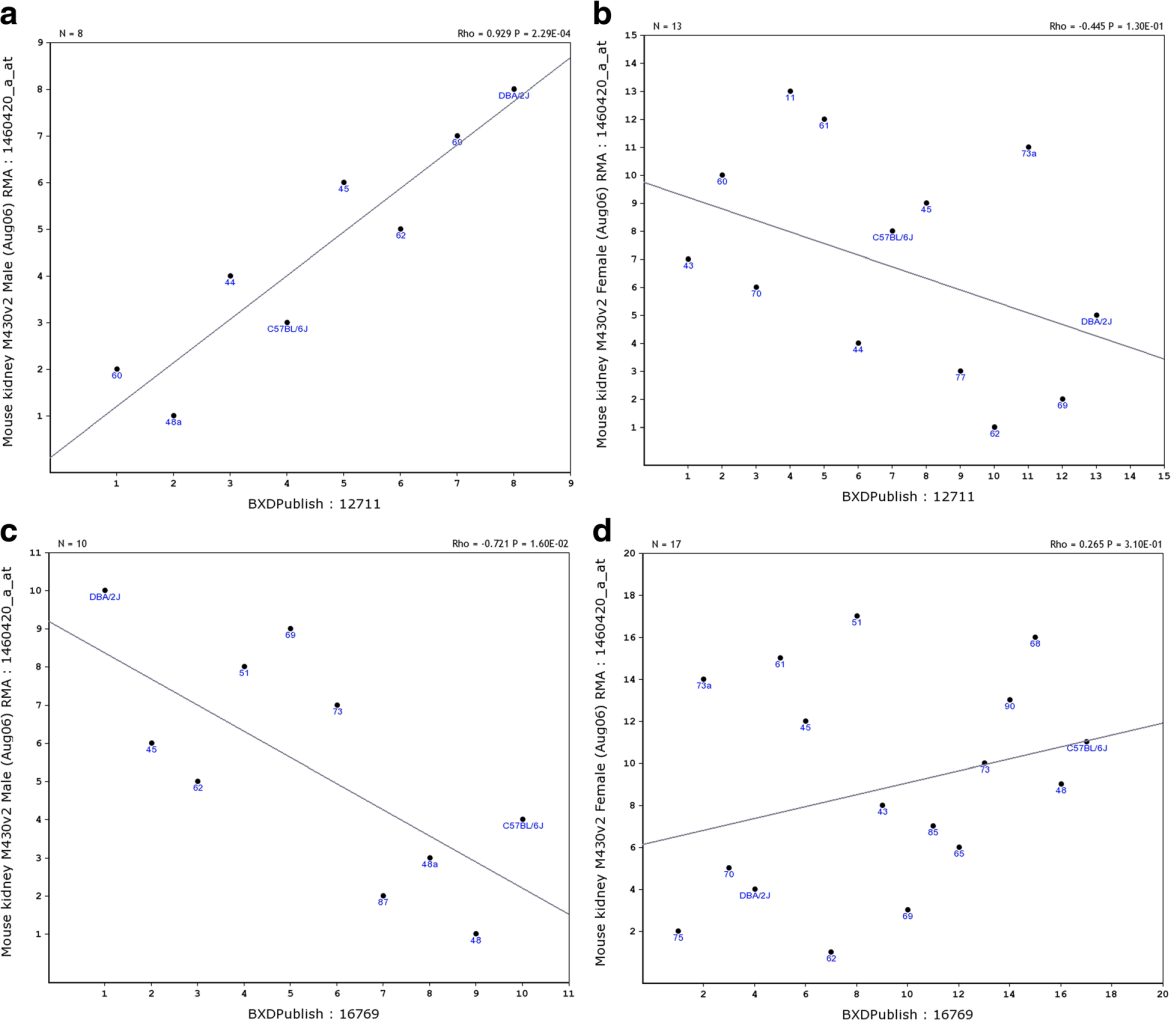
Correlation between the expression level of *Egfr* and known traits between male and female mice. **a**. Expression of *Il-4* from draining lymph node in male mice is strongly positively correlated to that of *Egfr*. Numbers on the *Y bar* indicate the expression levels of *Egfr*. Numbers on the *X bar* indicate expression levels of *Il-4*. **b**. Expression of *Il-4* in female and *Egfr* are weakly negatively correlated. Numbers on the *Y bar* indicate the expression levels of *Egfr*. Numbers on the *X bar* indicate expression levels of *Il-4.* **c**. In the male the expression of alpha-aminoadipate is negatively correlated to that of *Egfr.* Numbers on the *Y bar* indicate the expression levels of *Egfr*. Numbers on the *X bar* indicate expression levels of alpha-aminoadipate in male mice. **d**. In the female the expression of alpha-aminoadipate and that of *Egfr* are weakly positively correlated. Numbers on the *Y bar* indicate the expression levels of *Egfr*. Numbers on the *X bar* indicate expression levels of alpha-aminoadipate in female mice

Because of these sex differences, we expect some biological traits may be affected. In order to estimate the potential impact of such a sex difference in the *Egfr* expression on the immune system, we examined the correlation between the *Egfr* expression level and some available traits in GeneNetwork. While a majority of the straits did not show a difference in terms of correlation with the expression level of *Egfr* in both sexes, some did show significant differences. Figure 6a and b shows the significantly different effect of expression level of *Egfr* between sexes on the level of interleukin-4 (*Il4*) from draining lymph node, 3 weeks post infection (GeneNework ID: 12711). In the male, the expression of Il4 is strongly positively correlated to that of *Egfr*, (Fig. 7a) in female, with a Rho value of 0.929 for Spearman rank correlation. However, in the female, they are negatively correlated (Fig. 7b) with a Rho value of −0.445. Figure 7c and d shows the significantly different effect of expression level of *Egfr* between sexes on the level of alpha-aminoadipate levels measured by LC-MS/MS in pooled plasma after overnight fasting in males at 29 weeks of age [33]. While in the male the expression of alpha-aminoadipate is strongly negatively correlated to that of *Egfr* with a Rho value of −0.721 (Fig. 7c), in female they are weakly positively correlated with a Rho value of 0.265 (Fig. 7d).

### Potential sex difference in *Egfr* ligands

**Fig. 8:**
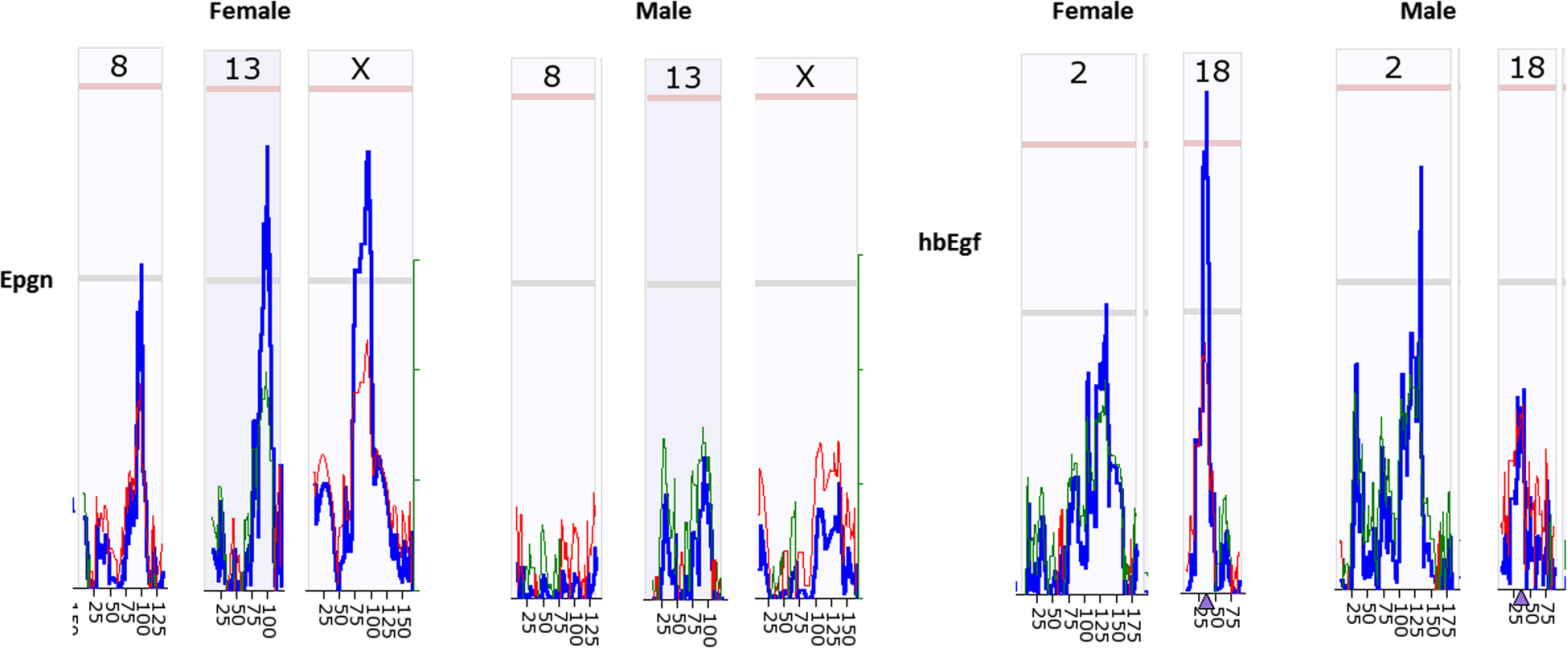
Detection of eQTL for regulation of *Egfr* ligands *Epgn* and *hbEgf* expression levels in kidney of mouse RI strains using probes of *Egfr*. The number on top of each individual figure indicates the number of chromosome. Pink color lines on top of the individual figure indicate the threshold for significant level. Light grey lines indicate the threshold for suggestive level. Numbers on the bottom indicate the Mb on the chromosomes

As function of *Egfr* is regulated by the availability of its ligand, we further reexamined the expression levels of two *Egfr* ligands, the heparin-binding epidermal growth factor (*hbEgf*) and Epithelial Mitogen (*Epgn*). The eQTLs from two ligands were all different from each other between female and male mice. Figure 8 shows the eQTL detected from these two *Egfr* ligands. Mapping with the probe from *Epgn*, 3 eQTLs at suggestive levels were detected from chromosome 8, 13 and X from female, while no eQTL was detected from the male. Mapping with the probe from *hbEgf*, one eQTL at significant level was detected from chromosome 18 from female, while an eQTL at suggestive level on chromosome 2 was detected from the male. These data strengthen the importance of sex difference in drug design and treatment when targeting at *Egfr*.

## Discussion

Our data clearly demonstrated that there are sex differences in the molecular pathways of *Egfr* in the kidney. Gene expression profiles from each RI strain are generated from multiple homozygous mice [20–22, 29]. Female and male mice in the same strains are kept in the same animal facility with the same environment. The reliability of the data was demonstrated by similar expression levels of *Actin B* between female and male mice in these strains. The significant sex difference in the expression levels of *Egfr* in the kidney of these stains provided the solid foundation for this important study. Accordingly, the information on sex differences from these RI strains are important resources for further study.

Majority sex differentially regulated genes from this study have not been reported. However, a few of them have demonstrated their important roles in the pathways of *Egfr*. For example, *NRP1* is widely expressed in cancer cells and in advanced human tumors. Most importantly, NRP1 is the co-receptor of EGFR [34]. It has been reported that NRP1 can control EGFR signaling and tumor growth [35]. The expression of *Nrp1* is strongly negatively associated with that of *Egfr* in female mice but did not show a strong connection with *Egfr* in male mice. In such a case, it is important to know whether such a difference exists during tumor development in human kidney. Accordingly, these differences should be brought to attention for the development of drugs for cancers targeting *Egfr* in kidney diseases. It is also known that in humans VEGF signaling controls the Gonadotropin-releasing hormone (GnRH) neurons survival via NRP1 [36]. Hormones play an important role in the expression of VEGF as well as NRP1 [33–35]*.* The significant difference in correlation with two important traits to *Egfr* confirms that the sex difference may have a significant impact on a significant number of traits including the immune system. For the drug targeted *Egfr* pathway, attention should be paid to the gender differences of the drug response in the clinical trials. Dosages and methods of application of these drugs may need to be modified based on the gender.

It is known that in humans *EGFR* is a hormone related gene [27, 28]. It is reasonable to assume that its gender differential regulation to a certain degree hormone relevant. In addition to the *Nrp1*, we found that several other differentially expressed genes between female and male mice are also hormone related genes. For example, *Prg1* is a progestin-responsive gene [37]. In humans, TCEB1 promotes invasion of prostate cancer cells and is involved in the development of hormone-refractory prostate cancer [38]. TSLP is involved in the regulation of estrogen on the secretion of MCP-1 and IL-8, and the growth of ESCs through JNK and NF-κB signal pathways [39, 40].

Interestingly, the eQTL that regulate the *Egfr* expression levels between female and male mice showed significant differences. Although several eQTL between mice are located on the same chromosomes in both sexes, these same eQTL are usually not being mapped with the same probes between sexes. For example, eQTL on chromosome 4 was mapped in females by probe 1424932_at while it was mapped in males by probe 1432647_at. EQTL on chromosome 9 was mapped in females by probe 1432647_at while it was mapped in males by probe 1424932_at, 1435888_at, and 1457563_at. Furthermore, eQTL was mapped on chromosome 14 by three probes 1435888_at, 1454313_at and 1460420_a_at in females but not by any probe in male mice. This complicated regulation of *Egfr* between females and males suggest that it is very likely that various mechanisms such as different splicing sites, multiple promote sites as well as differences in binding sites may involve in the regulation of *Egfr*. Further studies are necessary to clarify their mechanisms.

Similarly, our data also showed that potentially the expression levels of many *Egfr* ligands and/or relevant genes may also have sex-related differences. We demonstrated that different eQTL regulate the sex difference of *hbEgf* and *Epgn*. We believe that the expression of many *Egfr* ligands may be regulated differently in females and males. The sex differential expression may directly or indirectly affect the disease incidents and response to drug treatment. For example, hbEGF involves in kidney diseases such as glomerulonephritis and diabetic kidney disease [41, 42]. The interaction between *Egfr* and *hbEgf* in females and males may play an essential role on the effect of *hbEgf* on the diseases. Overexpression of *Epgn* during embryonic development induces reversible, epidermal growth factor receptor-dependent sebaceous gland hyperplasia [43]. We also notice that although *Egfr* is ubiquitously expressed [44, 45], its function is regulated by its ligands which are highly spatially restricted. Therefore further analysis of the sex differences of expression of *Egfr* ligands is necessary.

Similar to our previous study, this study is that our analysis is at one time point [29]. The differentially expressed genes between female and male mice may vary at different life stages. Future studies at different ages may lead to a comprehensive understanding of the sex difference in the molecular pathways of the *Egfr* axis in kidney. Similar to many studies, our data set is from the RI strains that were derived from two mouse strains, C57BL/6 J and DBA/2 J. Mouse strain specific pathways have been known [46, 47]. The results may represent sex difference under certain genomic backgrounds. Studies using other strains may reveal different mechanisms..

Like all other studies using animal models, the sex differences from this study may or may not represent that in humans [48]. Thus, not all the sex differentially expressed genes in mouse kidney are differentially expressed between males and females in human kidney. However, this study and our other study [29] strongly suggest that a gender difference in the EGFR pathway in humans exists.

We understand that, although the RNA expression levels are matched to the protein levels in a majority of genes, in some cases, the gene expression level does not necessarily represent the protein level. As to *Egfr*, a considerable number of publications have indicated the consistency between the RNA expression level and protein level [49–51]. However, a relatively large number of genes including its ligands are in the pathways in regulation of *Egfr*. It is important to examine whether their RNA expression levels agree with their protein levels in the future. On the other hand, the disagreement between protein and RNA levels does not necessarily invalidate the importance of the gene expression. We now know that RNA itself plays an important role in many biological functions including regulation of the expression of genes.

## Conclusion

Our study reveals that there are differences in the molecular pathways of *Egfr* in the kidney between female and male mice. Many of sex differential expressed genes are related to hormone metabolism. These data reinforce the importance of being aware of the gender difference in drug development targeting *Egfr* and its ligands and their pathways.

## Additional file

Additional file 1: Table S1. Correlation matrix of top 50 genes that are close correlated to *Egfr* in male mice. (DOCX 82 kb)

## Abbreviations

EGFR: Epidermal growth factor receptor
eQTL: Expression quantitative trait loci
LAP: Lapatinib ditosylate
RI: Recombinant inbred
