# Supplementary material for: Sex difference in EGFR pathways in mouse kidney-potential impact on the immune system
Source: BMC Genet. 2016 Nov 24;17:146. doi: 10.1186/s12863-016-0449-3 (PMC5122204; doi:10.1186/s12863-016-0449-3)
Supplement: Additional file 1: Table S1. — Correlation matrix of top 50 genes that are close correlated to Egfr in male mice. (DOCX 82 kb) [file 12863_2016_449_MOESM1_ESM.docx]

| e a r s o n     r |  | [Trait1](javascript:showDatabase2('MA_M2M_0706_R','1445101_at','');) | [Trait2](javascript:showDatabase2('MA_M2M_0706_R','1451285_at','');) | [Trait3](javascript:showDatabase2('MA_M2M_0706_R','1441347_at','');) | [Trait4](javascript:showDatabase2('MA_M2M_0706_R','1426900_at','');) | [Trait5](javascript:showDatabase2('MA_M2M_0706_R','1434888_a_at','');) | [Trait6](javascript:showDatabase2('MA_M2M_0706_R','1434773_a_at','');) | [Trait7](javascript:showDatabase2('MA_M2M_0706_R','1452851_at','');) | [Trait8](javascript:showDatabase2('MA_M2M_0706_R','1441948_x_at','');) | [Trait9](javascript:showDatabase2('MA_M2M_0706_R','1440160_x_at','');) | [Trait10](javascript:showDatabase2('MA_M2M_0706_R','1424692_at','');) | [Trait11](javascript:showDatabase2('MA_M2M_0706_R','1433112_at','');) | [Trait12](javascript:showDatabase2('MA_M2M_0706_R','1459962_at','');) | [Trait13](javascript:showDatabase2('MA_M2M_0706_R','1455331_at','');) | [Trait14](javascript:showDatabase2('MA_M2M_0706_R','1447399_at','');) | [Trait15](javascript:showDatabase2('MA_M2M_0706_R','1434968_a_at','');) | [Trait16](javascript:showDatabase2('MA_M2M_0706_R','1447335_x_at','');) | [Trait17](javascript:showDatabase2('MA_M2M_0706_R','1442239_at','');) | [Trait18](javascript:showDatabase2('MA_M2M_0706_R','1459865_x_at','');) | [Trait19](javascript:showDatabase2('MA_M2M_0706_R','1438647_x_at','');) | [Trait20](javascript:showDatabase2('MA_M2M_0706_R','1432333_a_at','');) | [Trait21](javascript:showDatabase2('MA_M2M_0706_R','1444028_s_at','');) | [Trait22](javascript:showDatabase2('MA_M2M_0706_R','1460420_a_at','');) | [Trait23](javascript:showDatabase2('MA_M2M_0706_R','1451530_at','');) | [Trait24](javascript:showDatabase2('MA_M2M_0706_R','1423785_at','');) | [Trait25](javascript:showDatabase2('MA_M2M_0706_R','1431972_a_at','');) | [Trait26](javascript:showDatabase2('MA_M2M_0706_R','1448347_a_at','');) | [Trait27](javascript:showDatabase2('MA_M2M_0706_R','1418492_at','');) | [Trait28](javascript:showDatabase2('MA_M2M_0706_R','1427185_at','');) | [Trait29](javascript:showDatabase2('MA_M2M_0706_R','1430500_s_at','');) | [Trait30](javascript:showDatabase2('MA_M2M_0706_R','1447549_x_at','');) | [Trait31](javascript:showDatabase2('MA_M2M_0706_R','1448943_at','');) | [Trait32](javascript:showDatabase2('MA_M2M_0706_R','1433162_at','');) | [Trait33](javascript:showDatabase2('MA_M2M_0706_R','1443494_at','');) | [Trait34](javascript:showDatabase2('MA_M2M_0706_R','1417426_at','');) | [Trait35](javascript:showDatabase2('MA_M2M_0706_R','1416588_at','');) | [Trait36](javascript:showDatabase2('MA_M2M_0706_R','1450994_at','');) | [Trait37](javascript:showDatabase2('MA_M2M_0706_R','1420304_x_at','');) | [Trait38](javascript:showDatabase2('MA_M2M_0706_R','1416500_at','');) | [Trait39](javascript:showDatabase2('MA_M2M_0706_R','1424199_at','');) | [Trait40](javascript:showDatabase2('MA_M2M_0706_R','1437995_x_at','');) | [Trait41](javascript:showDatabase2('MA_M2M_0706_R','1427914_a_at','');) | [Trait42](javascript:showDatabase2('MA_M2M_0706_R','1420570_x_at','');) | [Trait43](javascript:showDatabase2('MA_M2M_0706_R','1431015_at','');) | [Trait44](javascript:showDatabase2('MA_M2M_0706_R','1423852_at','');) | [Trait45](javascript:showDatabase2('MA_M2M_0706_R','1431799_at','');) | [Trait46](javascript:showDatabase2('MA_M2M_0706_R','1423898_a_at','');) | [Trait47](javascript:showDatabase2('MA_M2M_0706_R','1438579_at','');) | [Trait48](javascript:showDatabase2('MA_M2M_0706_R','1448102_a_at','');) | [Trait49](javascript:showDatabase2('MA_M2M_0706_R','1420943_at','');) | [Trait50](javascript:showDatabase2('MA_M2M_0706_R','1447326_s_at','');) |
| --- | --- | --- | --- | --- | --- | --- | --- | --- | --- | --- | --- | --- | --- | --- | --- | --- | --- | --- | --- | --- | --- | --- | --- | --- | --- | --- | --- | --- | --- | --- | --- | --- | --- | --- | --- | --- | --- | --- | --- | --- | --- | --- | --- | --- | --- | --- | --- | --- | --- | --- | --- |
|  | [Trait 1: MA_M2M_0706_R::1445101_at](javascript:showDatabase2('MA_M2M_0706_R','1445101_at','');)  9130221J18Rik on Chr 10 @ 66.628983 Mb  RIKEN cDNA 9130221J18 gene | [n 38](javascript:showDatabase2('MA_M2M_0706_R','1445101_at','')) | [-0.411 38](javascript:showCorrelationPlot2(db='MA_M2M_0706_R',ProbeSetID='1445101_at',CellID='',db2='MA_M2M_0706_R',ProbeSetID2='1451285_at',CellID2='',rank='1')) | [-0.634 38](javascript:showCorrelationPlot2(db='MA_M2M_0706_R',ProbeSetID='1445101_at',CellID='',db2='MA_M2M_0706_R',ProbeSetID2='1441347_at',CellID2='',rank='1')) | [0.536 38](javascript:showCorrelationPlot2(db='MA_M2M_0706_R',ProbeSetID='1445101_at',CellID='',db2='MA_M2M_0706_R',ProbeSetID2='1426900_at',CellID2='',rank='1')) | [0.391 38](javascript:showCorrelationPlot2(db='MA_M2M_0706_R',ProbeSetID='1445101_at',CellID='',db2='MA_M2M_0706_R',ProbeSetID2='1434888_a_at',CellID2='',rank='1')) | [0.534 38](javascript:showCorrelationPlot2(db='MA_M2M_0706_R',ProbeSetID='1445101_at',CellID='',db2='MA_M2M_0706_R',ProbeSetID2='1434773_a_at',CellID2='',rank='1')) | [-0.474 38](javascript:showCorrelationPlot2(db='MA_M2M_0706_R',ProbeSetID='1445101_at',CellID='',db2='MA_M2M_0706_R',ProbeSetID2='1452851_at',CellID2='',rank='1')) | [0.502 38](javascript:showCorrelationPlot2(db='MA_M2M_0706_R',ProbeSetID='1445101_at',CellID='',db2='MA_M2M_0706_R',ProbeSetID2='1441948_x_at',CellID2='',rank='1')) | [-0.727 38](javascript:showCorrelationPlot2(db='MA_M2M_0706_R',ProbeSetID='1445101_at',CellID='',db2='MA_M2M_0706_R',ProbeSetID2='1440160_x_at',CellID2='',rank='1')) | [0.631 38](javascript:showCorrelationPlot2(db='MA_M2M_0706_R',ProbeSetID='1445101_at',CellID='',db2='MA_M2M_0706_R',ProbeSetID2='1424692_at',CellID2='',rank='1')) | [-0.658 38](javascript:showCorrelationPlot2(db='MA_M2M_0706_R',ProbeSetID='1445101_at',CellID='',db2='MA_M2M_0706_R',ProbeSetID2='1433112_at',CellID2='',rank='1')) | [0.349 38](javascript:showCorrelationPlot2(db='MA_M2M_0706_R',ProbeSetID='1445101_at',CellID='',db2='MA_M2M_0706_R',ProbeSetID2='1459962_at',CellID2='',rank='1')) | [-0.578 38](javascript:showCorrelationPlot2(db='MA_M2M_0706_R',ProbeSetID='1445101_at',CellID='',db2='MA_M2M_0706_R',ProbeSetID2='1455331_at',CellID2='',rank='1')) | [-0.572 38](javascript:showCorrelationPlot2(db='MA_M2M_0706_R',ProbeSetID='1445101_at',CellID='',db2='MA_M2M_0706_R',ProbeSetID2='1447399_at',CellID2='',rank='1')) | [0.482 38](javascript:showCorrelationPlot2(db='MA_M2M_0706_R',ProbeSetID='1445101_at',CellID='',db2='MA_M2M_0706_R',ProbeSetID2='1434968_a_at',CellID2='',rank='1')) | [-0.606 38](javascript:showCorrelationPlot2(db='MA_M2M_0706_R',ProbeSetID='1445101_at',CellID='',db2='MA_M2M_0706_R',ProbeSetID2='1447335_x_at',CellID2='',rank='1')) | [-0.614 38](javascript:showCorrelationPlot2(db='MA_M2M_0706_R',ProbeSetID='1445101_at',CellID='',db2='MA_M2M_0706_R',ProbeSetID2='1442239_at',CellID2='',rank='1')) | [-0.530 38](javascript:showCorrelationPlot2(db='MA_M2M_0706_R',ProbeSetID='1445101_at',CellID='',db2='MA_M2M_0706_R',ProbeSetID2='1459865_x_at',CellID2='',rank='1')) | [0.466 38](javascript:showCorrelationPlot2(db='MA_M2M_0706_R',ProbeSetID='1445101_at',CellID='',db2='MA_M2M_0706_R',ProbeSetID2='1438647_x_at',CellID2='',rank='1')) | [-0.585 38](javascript:showCorrelationPlot2(db='MA_M2M_0706_R',ProbeSetID='1445101_at',CellID='',db2='MA_M2M_0706_R',ProbeSetID2='1432333_a_at',CellID2='',rank='1')) | [0.321 38](javascript:showCorrelationPlot2(db='MA_M2M_0706_R',ProbeSetID='1445101_at',CellID='',db2='MA_M2M_0706_R',ProbeSetID2='1444028_s_at',CellID2='',rank='1')) | [-0.621 38](javascript:showCorrelationPlot2(db='MA_M2M_0706_R',ProbeSetID='1445101_at',CellID='',db2='MA_M2M_0706_R',ProbeSetID2='1460420_a_at',CellID2='',rank='1')) | [-0.507 38](javascript:showCorrelationPlot2(db='MA_M2M_0706_R',ProbeSetID='1445101_at',CellID='',db2='MA_M2M_0706_R',ProbeSetID2='1451530_at',CellID2='',rank='1')) | [0.609 38](javascript:showCorrelationPlot2(db='MA_M2M_0706_R',ProbeSetID='1445101_at',CellID='',db2='MA_M2M_0706_R',ProbeSetID2='1423785_at',CellID2='',rank='1')) | [-0.405 38](javascript:showCorrelationPlot2(db='MA_M2M_0706_R',ProbeSetID='1445101_at',CellID='',db2='MA_M2M_0706_R',ProbeSetID2='1431972_a_at',CellID2='',rank='1')) | [0.514 38](javascript:showCorrelationPlot2(db='MA_M2M_0706_R',ProbeSetID='1445101_at',CellID='',db2='MA_M2M_0706_R',ProbeSetID2='1448347_a_at',CellID2='',rank='1')) | [-0.571 38](javascript:showCorrelationPlot2(db='MA_M2M_0706_R',ProbeSetID='1445101_at',CellID='',db2='MA_M2M_0706_R',ProbeSetID2='1418492_at',CellID2='',rank='1')) | [0.447 38](javascript:showCorrelationPlot2(db='MA_M2M_0706_R',ProbeSetID='1445101_at',CellID='',db2='MA_M2M_0706_R',ProbeSetID2='1427185_at',CellID2='',rank='1')) | [0.531 38](javascript:showCorrelationPlot2(db='MA_M2M_0706_R',ProbeSetID='1445101_at',CellID='',db2='MA_M2M_0706_R',ProbeSetID2='1430500_s_at',CellID2='',rank='1')) | [-0.398 38](javascript:showCorrelationPlot2(db='MA_M2M_0706_R',ProbeSetID='1445101_at',CellID='',db2='MA_M2M_0706_R',ProbeSetID2='1447549_x_at',CellID2='',rank='1')) | [0.407 38](javascript:showCorrelationPlot2(db='MA_M2M_0706_R',ProbeSetID='1445101_at',CellID='',db2='MA_M2M_0706_R',ProbeSetID2='1448943_at',CellID2='',rank='1')) | [-0.598 38](javascript:showCorrelationPlot2(db='MA_M2M_0706_R',ProbeSetID='1445101_at',CellID='',db2='MA_M2M_0706_R',ProbeSetID2='1433162_at',CellID2='',rank='1')) | [-0.610 38](javascript:showCorrelationPlot2(db='MA_M2M_0706_R',ProbeSetID='1445101_at',CellID='',db2='MA_M2M_0706_R',ProbeSetID2='1443494_at',CellID2='',rank='1')) | [0.268 38](javascript:showCorrelationPlot2(db='MA_M2M_0706_R',ProbeSetID='1445101_at',CellID='',db2='MA_M2M_0706_R',ProbeSetID2='1417426_at',CellID2='',rank='1')) | [-0.618 38](javascript:showCorrelationPlot2(db='MA_M2M_0706_R',ProbeSetID='1445101_at',CellID='',db2='MA_M2M_0706_R',ProbeSetID2='1416588_at',CellID2='',rank='1')) | [0.382 38](javascript:showCorrelationPlot2(db='MA_M2M_0706_R',ProbeSetID='1445101_at',CellID='',db2='MA_M2M_0706_R',ProbeSetID2='1450994_at',CellID2='',rank='1')) | [-0.528 38](javascript:showCorrelationPlot2(db='MA_M2M_0706_R',ProbeSetID='1445101_at',CellID='',db2='MA_M2M_0706_R',ProbeSetID2='1420304_x_at',CellID2='',rank='1')) | [0.516 38](javascript:showCorrelationPlot2(db='MA_M2M_0706_R',ProbeSetID='1445101_at',CellID='',db2='MA_M2M_0706_R',ProbeSetID2='1416500_at',CellID2='',rank='1')) | [-0.702 38](javascript:showCorrelationPlot2(db='MA_M2M_0706_R',ProbeSetID='1445101_at',CellID='',db2='MA_M2M_0706_R',ProbeSetID2='1424199_at',CellID2='',rank='1')) | [0.476 38](javascript:showCorrelationPlot2(db='MA_M2M_0706_R',ProbeSetID='1445101_at',CellID='',db2='MA_M2M_0706_R',ProbeSetID2='1437995_x_at',CellID2='',rank='1')) | [0.389 38](javascript:showCorrelationPlot2(db='MA_M2M_0706_R',ProbeSetID='1445101_at',CellID='',db2='MA_M2M_0706_R',ProbeSetID2='1427914_a_at',CellID2='',rank='1')) | [-0.498 38](javascript:showCorrelationPlot2(db='MA_M2M_0706_R',ProbeSetID='1445101_at',CellID='',db2='MA_M2M_0706_R',ProbeSetID2='1420570_x_at',CellID2='',rank='1')) | [-0.469 38](javascript:showCorrelationPlot2(db='MA_M2M_0706_R',ProbeSetID='1445101_at',CellID='',db2='MA_M2M_0706_R',ProbeSetID2='1431015_at',CellID2='',rank='1')) | [0.546 38](javascript:showCorrelationPlot2(db='MA_M2M_0706_R',ProbeSetID='1445101_at',CellID='',db2='MA_M2M_0706_R',ProbeSetID2='1423852_at',CellID2='',rank='1')) | [-0.327 38](javascript:showCorrelationPlot2(db='MA_M2M_0706_R',ProbeSetID='1445101_at',CellID='',db2='MA_M2M_0706_R',ProbeSetID2='1431799_at',CellID2='',rank='1')) | [0.528 38](javascript:showCorrelationPlot2(db='MA_M2M_0706_R',ProbeSetID='1445101_at',CellID='',db2='MA_M2M_0706_R',ProbeSetID2='1423898_a_at',CellID2='',rank='1')) | [-0.656 38](javascript:showCorrelationPlot2(db='MA_M2M_0706_R',ProbeSetID='1445101_at',CellID='',db2='MA_M2M_0706_R',ProbeSetID2='1438579_at',CellID2='',rank='1')) | [0.415 38](javascript:showCorrelationPlot2(db='MA_M2M_0706_R',ProbeSetID='1445101_at',CellID='',db2='MA_M2M_0706_R',ProbeSetID2='1448102_a_at',CellID2='',rank='1')) | [-0.598 38](javascript:showCorrelationPlot2(db='MA_M2M_0706_R',ProbeSetID='1445101_at',CellID='',db2='MA_M2M_0706_R',ProbeSetID2='1420943_at',CellID2='',rank='1')) | [-0.625 38](javascript:showCorrelationPlot2(db='MA_M2M_0706_R',ProbeSetID='1445101_at',CellID='',db2='MA_M2M_0706_R',ProbeSetID2='1447326_s_at',CellID2='',rank='1')) |
|  | [Trait 2: MA_M2M_0706_R::1451285_at](javascript:showDatabase2('MA_M2M_0706_R','1451285_at','');)  Fus on Chr 7 @ 135.123689 Mb  fusion (involved in t(12;16) in malignant liposarcoma)  exon 12 (transQTL on Chr 4 in BXD eye data) | [-0.378 38](javascript:showCorrelationPlot2(db='MA_M2M_0706_R',ProbeSetID='1451285_at',CellID='',db2='MA_M2M_0706_R',ProbeSetID2='1445101_at',CellID2='',rank='0')) | [n 38](javascript:showDatabase2('MA_M2M_0706_R','1451285_at','')) | [0.573 38](javascript:showCorrelationPlot2(db='MA_M2M_0706_R',ProbeSetID='1451285_at',CellID='',db2='MA_M2M_0706_R',ProbeSetID2='1441347_at',CellID2='',rank='1')) | [-0.268 38](javascript:showCorrelationPlot2(db='MA_M2M_0706_R',ProbeSetID='1451285_at',CellID='',db2='MA_M2M_0706_R',ProbeSetID2='1426900_at',CellID2='',rank='1')) | [-0.471 38](javascript:showCorrelationPlot2(db='MA_M2M_0706_R',ProbeSetID='1451285_at',CellID='',db2='MA_M2M_0706_R',ProbeSetID2='1434888_a_at',CellID2='',rank='1')) | [-0.391 38](javascript:showCorrelationPlot2(db='MA_M2M_0706_R',ProbeSetID='1451285_at',CellID='',db2='MA_M2M_0706_R',ProbeSetID2='1434773_a_at',CellID2='',rank='1')) | [0.424 38](javascript:showCorrelationPlot2(db='MA_M2M_0706_R',ProbeSetID='1451285_at',CellID='',db2='MA_M2M_0706_R',ProbeSetID2='1452851_at',CellID2='',rank='1')) | [-0.482 38](javascript:showCorrelationPlot2(db='MA_M2M_0706_R',ProbeSetID='1451285_at',CellID='',db2='MA_M2M_0706_R',ProbeSetID2='1441948_x_at',CellID2='',rank='1')) | [0.373 38](javascript:showCorrelationPlot2(db='MA_M2M_0706_R',ProbeSetID='1451285_at',CellID='',db2='MA_M2M_0706_R',ProbeSetID2='1440160_x_at',CellID2='',rank='1')) | [-0.253 38](javascript:showCorrelationPlot2(db='MA_M2M_0706_R',ProbeSetID='1451285_at',CellID='',db2='MA_M2M_0706_R',ProbeSetID2='1424692_at',CellID2='',rank='1')) | [0.315 38](javascript:showCorrelationPlot2(db='MA_M2M_0706_R',ProbeSetID='1451285_at',CellID='',db2='MA_M2M_0706_R',ProbeSetID2='1433112_at',CellID2='',rank='1')) | [-0.418 38](javascript:showCorrelationPlot2(db='MA_M2M_0706_R',ProbeSetID='1451285_at',CellID='',db2='MA_M2M_0706_R',ProbeSetID2='1459962_at',CellID2='',rank='1')) | [0.594 38](javascript:showCorrelationPlot2(db='MA_M2M_0706_R',ProbeSetID='1451285_at',CellID='',db2='MA_M2M_0706_R',ProbeSetID2='1455331_at',CellID2='',rank='1')) | [0.500 38](javascript:showCorrelationPlot2(db='MA_M2M_0706_R',ProbeSetID='1451285_at',CellID='',db2='MA_M2M_0706_R',ProbeSetID2='1447399_at',CellID2='',rank='1')) | [-0.233 38](javascript:showCorrelationPlot2(db='MA_M2M_0706_R',ProbeSetID='1451285_at',CellID='',db2='MA_M2M_0706_R',ProbeSetID2='1434968_a_at',CellID2='',rank='1')) | [0.289 38](javascript:showCorrelationPlot2(db='MA_M2M_0706_R',ProbeSetID='1451285_at',CellID='',db2='MA_M2M_0706_R',ProbeSetID2='1447335_x_at',CellID2='',rank='1')) | [0.419 38](javascript:showCorrelationPlot2(db='MA_M2M_0706_R',ProbeSetID='1451285_at',CellID='',db2='MA_M2M_0706_R',ProbeSetID2='1442239_at',CellID2='',rank='1')) | [0.324 38](javascript:showCorrelationPlot2(db='MA_M2M_0706_R',ProbeSetID='1451285_at',CellID='',db2='MA_M2M_0706_R',ProbeSetID2='1459865_x_at',CellID2='',rank='1')) | [-0.561 38](javascript:showCorrelationPlot2(db='MA_M2M_0706_R',ProbeSetID='1451285_at',CellID='',db2='MA_M2M_0706_R',ProbeSetID2='1438647_x_at',CellID2='',rank='1')) | [0.629 38](javascript:showCorrelationPlot2(db='MA_M2M_0706_R',ProbeSetID='1451285_at',CellID='',db2='MA_M2M_0706_R',ProbeSetID2='1432333_a_at',CellID2='',rank='1')) | [-0.370 38](javascript:showCorrelationPlot2(db='MA_M2M_0706_R',ProbeSetID='1451285_at',CellID='',db2='MA_M2M_0706_R',ProbeSetID2='1444028_s_at',CellID2='',rank='1')) | [0.570 38](javascript:showCorrelationPlot2(db='MA_M2M_0706_R',ProbeSetID='1451285_at',CellID='',db2='MA_M2M_0706_R',ProbeSetID2='1460420_a_at',CellID2='',rank='1')) | [0.434 38](javascript:showCorrelationPlot2(db='MA_M2M_0706_R',ProbeSetID='1451285_at',CellID='',db2='MA_M2M_0706_R',ProbeSetID2='1451530_at',CellID2='',rank='1')) | [-0.432 38](javascript:showCorrelationPlot2(db='MA_M2M_0706_R',ProbeSetID='1451285_at',CellID='',db2='MA_M2M_0706_R',ProbeSetID2='1423785_at',CellID2='',rank='1')) | [0.440 38](javascript:showCorrelationPlot2(db='MA_M2M_0706_R',ProbeSetID='1451285_at',CellID='',db2='MA_M2M_0706_R',ProbeSetID2='1431972_a_at',CellID2='',rank='1')) | [-0.632 38](javascript:showCorrelationPlot2(db='MA_M2M_0706_R',ProbeSetID='1451285_at',CellID='',db2='MA_M2M_0706_R',ProbeSetID2='1448347_a_at',CellID2='',rank='1')) | [0.548 38](javascript:showCorrelationPlot2(db='MA_M2M_0706_R',ProbeSetID='1451285_at',CellID='',db2='MA_M2M_0706_R',ProbeSetID2='1418492_at',CellID2='',rank='1')) | [-0.452 38](javascript:showCorrelationPlot2(db='MA_M2M_0706_R',ProbeSetID='1451285_at',CellID='',db2='MA_M2M_0706_R',ProbeSetID2='1427185_at',CellID2='',rank='1')) | [-0.415 38](javascript:showCorrelationPlot2(db='MA_M2M_0706_R',ProbeSetID='1451285_at',CellID='',db2='MA_M2M_0706_R',ProbeSetID2='1430500_s_at',CellID2='',rank='1')) | [0.437 38](javascript:showCorrelationPlot2(db='MA_M2M_0706_R',ProbeSetID='1451285_at',CellID='',db2='MA_M2M_0706_R',ProbeSetID2='1447549_x_at',CellID2='',rank='1')) | [-0.246 38](javascript:showCorrelationPlot2(db='MA_M2M_0706_R',ProbeSetID='1451285_at',CellID='',db2='MA_M2M_0706_R',ProbeSetID2='1448943_at',CellID2='',rank='1')) | [0.403 38](javascript:showCorrelationPlot2(db='MA_M2M_0706_R',ProbeSetID='1451285_at',CellID='',db2='MA_M2M_0706_R',ProbeSetID2='1433162_at',CellID2='',rank='1')) | [0.463 38](javascript:showCorrelationPlot2(db='MA_M2M_0706_R',ProbeSetID='1451285_at',CellID='',db2='MA_M2M_0706_R',ProbeSetID2='1443494_at',CellID2='',rank='1')) | [-0.379 38](javascript:showCorrelationPlot2(db='MA_M2M_0706_R',ProbeSetID='1451285_at',CellID='',db2='MA_M2M_0706_R',ProbeSetID2='1417426_at',CellID2='',rank='1')) | [0.512 38](javascript:showCorrelationPlot2(db='MA_M2M_0706_R',ProbeSetID='1451285_at',CellID='',db2='MA_M2M_0706_R',ProbeSetID2='1416588_at',CellID2='',rank='1')) | [-0.432 38](javascript:showCorrelationPlot2(db='MA_M2M_0706_R',ProbeSetID='1451285_at',CellID='',db2='MA_M2M_0706_R',ProbeSetID2='1450994_at',CellID2='',rank='1')) | [0.438 38](javascript:showCorrelationPlot2(db='MA_M2M_0706_R',ProbeSetID='1451285_at',CellID='',db2='MA_M2M_0706_R',ProbeSetID2='1420304_x_at',CellID2='',rank='1')) | [-0.470 38](javascript:showCorrelationPlot2(db='MA_M2M_0706_R',ProbeSetID='1451285_at',CellID='',db2='MA_M2M_0706_R',ProbeSetID2='1416500_at',CellID2='',rank='1')) | [0.649 38](javascript:showCorrelationPlot2(db='MA_M2M_0706_R',ProbeSetID='1451285_at',CellID='',db2='MA_M2M_0706_R',ProbeSetID2='1424199_at',CellID2='',rank='1')) | [-0.359 38](javascript:showCorrelationPlot2(db='MA_M2M_0706_R',ProbeSetID='1451285_at',CellID='',db2='MA_M2M_0706_R',ProbeSetID2='1437995_x_at',CellID2='',rank='1')) | [-0.411 38](javascript:showCorrelationPlot2(db='MA_M2M_0706_R',ProbeSetID='1451285_at',CellID='',db2='MA_M2M_0706_R',ProbeSetID2='1427914_a_at',CellID2='',rank='1')) | [0.409 38](javascript:showCorrelationPlot2(db='MA_M2M_0706_R',ProbeSetID='1451285_at',CellID='',db2='MA_M2M_0706_R',ProbeSetID2='1420570_x_at',CellID2='',rank='1')) | [0.465 38](javascript:showCorrelationPlot2(db='MA_M2M_0706_R',ProbeSetID='1451285_at',CellID='',db2='MA_M2M_0706_R',ProbeSetID2='1431015_at',CellID2='',rank='1')) | [-0.470 38](javascript:showCorrelationPlot2(db='MA_M2M_0706_R',ProbeSetID='1451285_at',CellID='',db2='MA_M2M_0706_R',ProbeSetID2='1423852_at',CellID2='',rank='1')) | [0.219 38](javascript:showCorrelationPlot2(db='MA_M2M_0706_R',ProbeSetID='1451285_at',CellID='',db2='MA_M2M_0706_R',ProbeSetID2='1431799_at',CellID2='',rank='1')) | [-0.485 38](javascript:showCorrelationPlot2(db='MA_M2M_0706_R',ProbeSetID='1451285_at',CellID='',db2='MA_M2M_0706_R',ProbeSetID2='1423898_a_at',CellID2='',rank='1')) | [0.620 38](javascript:showCorrelationPlot2(db='MA_M2M_0706_R',ProbeSetID='1451285_at',CellID='',db2='MA_M2M_0706_R',ProbeSetID2='1438579_at',CellID2='',rank='1')) | [-0.441 38](javascript:showCorrelationPlot2(db='MA_M2M_0706_R',ProbeSetID='1451285_at',CellID='',db2='MA_M2M_0706_R',ProbeSetID2='1448102_a_at',CellID2='',rank='1')) | [0.395 38](javascript:showCorrelationPlot2(db='MA_M2M_0706_R',ProbeSetID='1451285_at',CellID='',db2='MA_M2M_0706_R',ProbeSetID2='1420943_at',CellID2='',rank='1')) | [0.403 38](javascript:showCorrelationPlot2(db='MA_M2M_0706_R',ProbeSetID='1451285_at',CellID='',db2='MA_M2M_0706_R',ProbeSetID2='1447326_s_at',CellID2='',rank='1')) |
|  | [Trait 3: MA_M2M_0706_R::1441347_at](javascript:showDatabase2('MA_M2M_0706_R','1441347_at','');)  Hrbl on Chr 5 @ 138.124275 Mb  HIV-1 Rev binding protein-like | [-0.627 38](javascript:showCorrelationPlot2(db='MA_M2M_0706_R',ProbeSetID='1441347_at',CellID='',db2='MA_M2M_0706_R',ProbeSetID2='1445101_at',CellID2='',rank='0')) | [0.564 38](javascript:showCorrelationPlot2(db='MA_M2M_0706_R',ProbeSetID='1441347_at',CellID='',db2='MA_M2M_0706_R',ProbeSetID2='1451285_at',CellID2='',rank='0')) | [n 38](javascript:showDatabase2('MA_M2M_0706_R','1441347_at','')) | [-0.436 38](javascript:showCorrelationPlot2(db='MA_M2M_0706_R',ProbeSetID='1441347_at',CellID='',db2='MA_M2M_0706_R',ProbeSetID2='1426900_at',CellID2='',rank='1')) | [-0.372 38](javascript:showCorrelationPlot2(db='MA_M2M_0706_R',ProbeSetID='1441347_at',CellID='',db2='MA_M2M_0706_R',ProbeSetID2='1434888_a_at',CellID2='',rank='1')) | [-0.348 38](javascript:showCorrelationPlot2(db='MA_M2M_0706_R',ProbeSetID='1441347_at',CellID='',db2='MA_M2M_0706_R',ProbeSetID2='1434773_a_at',CellID2='',rank='1')) | [0.562 38](javascript:showCorrelationPlot2(db='MA_M2M_0706_R',ProbeSetID='1441347_at',CellID='',db2='MA_M2M_0706_R',ProbeSetID2='1452851_at',CellID2='',rank='1')) | [-0.557 38](javascript:showCorrelationPlot2(db='MA_M2M_0706_R',ProbeSetID='1441347_at',CellID='',db2='MA_M2M_0706_R',ProbeSetID2='1441948_x_at',CellID2='',rank='1')) | [0.514 38](javascript:showCorrelationPlot2(db='MA_M2M_0706_R',ProbeSetID='1441347_at',CellID='',db2='MA_M2M_0706_R',ProbeSetID2='1440160_x_at',CellID2='',rank='1')) | [-0.412 38](javascript:showCorrelationPlot2(db='MA_M2M_0706_R',ProbeSetID='1441347_at',CellID='',db2='MA_M2M_0706_R',ProbeSetID2='1424692_at',CellID2='',rank='1')) | [0.655 38](javascript:showCorrelationPlot2(db='MA_M2M_0706_R',ProbeSetID='1441347_at',CellID='',db2='MA_M2M_0706_R',ProbeSetID2='1433112_at',CellID2='',rank='1')) | [-0.552 38](javascript:showCorrelationPlot2(db='MA_M2M_0706_R',ProbeSetID='1441347_at',CellID='',db2='MA_M2M_0706_R',ProbeSetID2='1459962_at',CellID2='',rank='1')) | [0.671 38](javascript:showCorrelationPlot2(db='MA_M2M_0706_R',ProbeSetID='1441347_at',CellID='',db2='MA_M2M_0706_R',ProbeSetID2='1455331_at',CellID2='',rank='1')) | [0.655 38](javascript:showCorrelationPlot2(db='MA_M2M_0706_R',ProbeSetID='1441347_at',CellID='',db2='MA_M2M_0706_R',ProbeSetID2='1447399_at',CellID2='',rank='1')) | [-0.321 38](javascript:showCorrelationPlot2(db='MA_M2M_0706_R',ProbeSetID='1441347_at',CellID='',db2='MA_M2M_0706_R',ProbeSetID2='1434968_a_at',CellID2='',rank='1')) | [0.591 38](javascript:showCorrelationPlot2(db='MA_M2M_0706_R',ProbeSetID='1441347_at',CellID='',db2='MA_M2M_0706_R',ProbeSetID2='1447335_x_at',CellID2='',rank='1')) | [0.595 38](javascript:showCorrelationPlot2(db='MA_M2M_0706_R',ProbeSetID='1441347_at',CellID='',db2='MA_M2M_0706_R',ProbeSetID2='1442239_at',CellID2='',rank='1')) | [0.317 38](javascript:showCorrelationPlot2(db='MA_M2M_0706_R',ProbeSetID='1441347_at',CellID='',db2='MA_M2M_0706_R',ProbeSetID2='1459865_x_at',CellID2='',rank='1')) | [-0.542 38](javascript:showCorrelationPlot2(db='MA_M2M_0706_R',ProbeSetID='1441347_at',CellID='',db2='MA_M2M_0706_R',ProbeSetID2='1438647_x_at',CellID2='',rank='1')) | [0.573 38](javascript:showCorrelationPlot2(db='MA_M2M_0706_R',ProbeSetID='1441347_at',CellID='',db2='MA_M2M_0706_R',ProbeSetID2='1432333_a_at',CellID2='',rank='1')) | [-0.324 38](javascript:showCorrelationPlot2(db='MA_M2M_0706_R',ProbeSetID='1441347_at',CellID='',db2='MA_M2M_0706_R',ProbeSetID2='1444028_s_at',CellID2='',rank='1')) | [0.558 38](javascript:showCorrelationPlot2(db='MA_M2M_0706_R',ProbeSetID='1441347_at',CellID='',db2='MA_M2M_0706_R',ProbeSetID2='1460420_a_at',CellID2='',rank='1')) | [0.673 38](javascript:showCorrelationPlot2(db='MA_M2M_0706_R',ProbeSetID='1441347_at',CellID='',db2='MA_M2M_0706_R',ProbeSetID2='1451530_at',CellID2='',rank='1')) | [-0.616 38](javascript:showCorrelationPlot2(db='MA_M2M_0706_R',ProbeSetID='1441347_at',CellID='',db2='MA_M2M_0706_R',ProbeSetID2='1423785_at',CellID2='',rank='1')) | [0.327 38](javascript:showCorrelationPlot2(db='MA_M2M_0706_R',ProbeSetID='1441347_at',CellID='',db2='MA_M2M_0706_R',ProbeSetID2='1431972_a_at',CellID2='',rank='1')) | [-0.507 38](javascript:showCorrelationPlot2(db='MA_M2M_0706_R',ProbeSetID='1441347_at',CellID='',db2='MA_M2M_0706_R',ProbeSetID2='1448347_a_at',CellID2='',rank='1')) | [0.725 38](javascript:showCorrelationPlot2(db='MA_M2M_0706_R',ProbeSetID='1441347_at',CellID='',db2='MA_M2M_0706_R',ProbeSetID2='1418492_at',CellID2='',rank='1')) | [-0.547 38](javascript:showCorrelationPlot2(db='MA_M2M_0706_R',ProbeSetID='1441347_at',CellID='',db2='MA_M2M_0706_R',ProbeSetID2='1427185_at',CellID2='',rank='1')) | [-0.479 38](javascript:showCorrelationPlot2(db='MA_M2M_0706_R',ProbeSetID='1441347_at',CellID='',db2='MA_M2M_0706_R',ProbeSetID2='1430500_s_at',CellID2='',rank='1')) | [0.575 38](javascript:showCorrelationPlot2(db='MA_M2M_0706_R',ProbeSetID='1441347_at',CellID='',db2='MA_M2M_0706_R',ProbeSetID2='1447549_x_at',CellID2='',rank='1')) | [-0.243 38](javascript:showCorrelationPlot2(db='MA_M2M_0706_R',ProbeSetID='1441347_at',CellID='',db2='MA_M2M_0706_R',ProbeSetID2='1448943_at',CellID2='',rank='1')) | [0.524 38](javascript:showCorrelationPlot2(db='MA_M2M_0706_R',ProbeSetID='1441347_at',CellID='',db2='MA_M2M_0706_R',ProbeSetID2='1433162_at',CellID2='',rank='1')) | [0.606 38](javascript:showCorrelationPlot2(db='MA_M2M_0706_R',ProbeSetID='1441347_at',CellID='',db2='MA_M2M_0706_R',ProbeSetID2='1443494_at',CellID2='',rank='1')) | [-0.279 38](javascript:showCorrelationPlot2(db='MA_M2M_0706_R',ProbeSetID='1441347_at',CellID='',db2='MA_M2M_0706_R',ProbeSetID2='1417426_at',CellID2='',rank='1')) | [0.561 38](javascript:showCorrelationPlot2(db='MA_M2M_0706_R',ProbeSetID='1441347_at',CellID='',db2='MA_M2M_0706_R',ProbeSetID2='1416588_at',CellID2='',rank='1')) | [-0.264 38](javascript:showCorrelationPlot2(db='MA_M2M_0706_R',ProbeSetID='1441347_at',CellID='',db2='MA_M2M_0706_R',ProbeSetID2='1450994_at',CellID2='',rank='1')) | [0.645 38](javascript:showCorrelationPlot2(db='MA_M2M_0706_R',ProbeSetID='1441347_at',CellID='',db2='MA_M2M_0706_R',ProbeSetID2='1420304_x_at',CellID2='',rank='1')) | [-0.557 38](javascript:showCorrelationPlot2(db='MA_M2M_0706_R',ProbeSetID='1441347_at',CellID='',db2='MA_M2M_0706_R',ProbeSetID2='1416500_at',CellID2='',rank='1')) | [0.658 38](javascript:showCorrelationPlot2(db='MA_M2M_0706_R',ProbeSetID='1441347_at',CellID='',db2='MA_M2M_0706_R',ProbeSetID2='1424199_at',CellID2='',rank='1')) | [-0.455 38](javascript:showCorrelationPlot2(db='MA_M2M_0706_R',ProbeSetID='1441347_at',CellID='',db2='MA_M2M_0706_R',ProbeSetID2='1437995_x_at',CellID2='',rank='1')) | [-0.316 38](javascript:showCorrelationPlot2(db='MA_M2M_0706_R',ProbeSetID='1441347_at',CellID='',db2='MA_M2M_0706_R',ProbeSetID2='1427914_a_at',CellID2='',rank='1')) | [0.461 38](javascript:showCorrelationPlot2(db='MA_M2M_0706_R',ProbeSetID='1441347_at',CellID='',db2='MA_M2M_0706_R',ProbeSetID2='1420570_x_at',CellID2='',rank='1')) | [0.480 38](javascript:showCorrelationPlot2(db='MA_M2M_0706_R',ProbeSetID='1441347_at',CellID='',db2='MA_M2M_0706_R',ProbeSetID2='1431015_at',CellID2='',rank='1')) | [-0.485 38](javascript:showCorrelationPlot2(db='MA_M2M_0706_R',ProbeSetID='1441347_at',CellID='',db2='MA_M2M_0706_R',ProbeSetID2='1423852_at',CellID2='',rank='1')) | [0.092 38](javascript:showCorrelationPlot2(db='MA_M2M_0706_R',ProbeSetID='1441347_at',CellID='',db2='MA_M2M_0706_R',ProbeSetID2='1431799_at',CellID2='',rank='1')) | [-0.351 38](javascript:showCorrelationPlot2(db='MA_M2M_0706_R',ProbeSetID='1441347_at',CellID='',db2='MA_M2M_0706_R',ProbeSetID2='1423898_a_at',CellID2='',rank='1')) | [0.734 38](javascript:showCorrelationPlot2(db='MA_M2M_0706_R',ProbeSetID='1441347_at',CellID='',db2='MA_M2M_0706_R',ProbeSetID2='1438579_at',CellID2='',rank='1')) | [-0.570 38](javascript:showCorrelationPlot2(db='MA_M2M_0706_R',ProbeSetID='1441347_at',CellID='',db2='MA_M2M_0706_R',ProbeSetID2='1448102_a_at',CellID2='',rank='1')) | [0.564 38](javascript:showCorrelationPlot2(db='MA_M2M_0706_R',ProbeSetID='1441347_at',CellID='',db2='MA_M2M_0706_R',ProbeSetID2='1420943_at',CellID2='',rank='1')) | [0.468 38](javascript:showCorrelationPlot2(db='MA_M2M_0706_R',ProbeSetID='1441347_at',CellID='',db2='MA_M2M_0706_R',ProbeSetID2='1447326_s_at',CellID2='',rank='1')) |
|  | [Trait 4: MA_M2M_0706_R::1426900_at](javascript:showDatabase2('MA_M2M_0706_R','1426900_at','');)  Jmjd1c on Chr 10 @ 66.718475 Mb  jumonji domain containing 1C  3' UTR | [0.557 38](javascript:showCorrelationPlot2(db='MA_M2M_0706_R',ProbeSetID='1426900_at',CellID='',db2='MA_M2M_0706_R',ProbeSetID2='1445101_at',CellID2='',rank='0')) | [-0.268 38](javascript:showCorrelationPlot2(db='MA_M2M_0706_R',ProbeSetID='1426900_at',CellID='',db2='MA_M2M_0706_R',ProbeSetID2='1451285_at',CellID2='',rank='0')) | [-0.429 38](javascript:showCorrelationPlot2(db='MA_M2M_0706_R',ProbeSetID='1426900_at',CellID='',db2='MA_M2M_0706_R',ProbeSetID2='1441347_at',CellID2='',rank='0')) | [n 38](javascript:showDatabase2('MA_M2M_0706_R','1426900_at','')) | [0.651 38](javascript:showCorrelationPlot2(db='MA_M2M_0706_R',ProbeSetID='1426900_at',CellID='',db2='MA_M2M_0706_R',ProbeSetID2='1434888_a_at',CellID2='',rank='1')) | [0.451 38](javascript:showCorrelationPlot2(db='MA_M2M_0706_R',ProbeSetID='1426900_at',CellID='',db2='MA_M2M_0706_R',ProbeSetID2='1434773_a_at',CellID2='',rank='1')) | [-0.512 38](javascript:showCorrelationPlot2(db='MA_M2M_0706_R',ProbeSetID='1426900_at',CellID='',db2='MA_M2M_0706_R',ProbeSetID2='1452851_at',CellID2='',rank='1')) | [0.431 38](javascript:showCorrelationPlot2(db='MA_M2M_0706_R',ProbeSetID='1426900_at',CellID='',db2='MA_M2M_0706_R',ProbeSetID2='1441948_x_at',CellID2='',rank='1')) | [-0.640 38](javascript:showCorrelationPlot2(db='MA_M2M_0706_R',ProbeSetID='1426900_at',CellID='',db2='MA_M2M_0706_R',ProbeSetID2='1440160_x_at',CellID2='',rank='1')) | [0.575 38](javascript:showCorrelationPlot2(db='MA_M2M_0706_R',ProbeSetID='1426900_at',CellID='',db2='MA_M2M_0706_R',ProbeSetID2='1424692_at',CellID2='',rank='1')) | [-0.389 38](javascript:showCorrelationPlot2(db='MA_M2M_0706_R',ProbeSetID='1426900_at',CellID='',db2='MA_M2M_0706_R',ProbeSetID2='1433112_at',CellID2='',rank='1')) | [0.463 38](javascript:showCorrelationPlot2(db='MA_M2M_0706_R',ProbeSetID='1426900_at',CellID='',db2='MA_M2M_0706_R',ProbeSetID2='1459962_at',CellID2='',rank='1')) | [-0.365 38](javascript:showCorrelationPlot2(db='MA_M2M_0706_R',ProbeSetID='1426900_at',CellID='',db2='MA_M2M_0706_R',ProbeSetID2='1455331_at',CellID2='',rank='1')) | [-0.414 38](javascript:showCorrelationPlot2(db='MA_M2M_0706_R',ProbeSetID='1426900_at',CellID='',db2='MA_M2M_0706_R',ProbeSetID2='1447399_at',CellID2='',rank='1')) | [0.570 38](javascript:showCorrelationPlot2(db='MA_M2M_0706_R',ProbeSetID='1426900_at',CellID='',db2='MA_M2M_0706_R',ProbeSetID2='1434968_a_at',CellID2='',rank='1')) | [-0.609 38](javascript:showCorrelationPlot2(db='MA_M2M_0706_R',ProbeSetID='1426900_at',CellID='',db2='MA_M2M_0706_R',ProbeSetID2='1447335_x_at',CellID2='',rank='1')) | [-0.519 38](javascript:showCorrelationPlot2(db='MA_M2M_0706_R',ProbeSetID='1426900_at',CellID='',db2='MA_M2M_0706_R',ProbeSetID2='1442239_at',CellID2='',rank='1')) | [-0.623 38](javascript:showCorrelationPlot2(db='MA_M2M_0706_R',ProbeSetID='1426900_at',CellID='',db2='MA_M2M_0706_R',ProbeSetID2='1459865_x_at',CellID2='',rank='1')) | [0.399 38](javascript:showCorrelationPlot2(db='MA_M2M_0706_R',ProbeSetID='1426900_at',CellID='',db2='MA_M2M_0706_R',ProbeSetID2='1438647_x_at',CellID2='',rank='1')) | [-0.370 38](javascript:showCorrelationPlot2(db='MA_M2M_0706_R',ProbeSetID='1426900_at',CellID='',db2='MA_M2M_0706_R',ProbeSetID2='1432333_a_at',CellID2='',rank='1')) | [0.600 38](javascript:showCorrelationPlot2(db='MA_M2M_0706_R',ProbeSetID='1426900_at',CellID='',db2='MA_M2M_0706_R',ProbeSetID2='1444028_s_at',CellID2='',rank='1')) | [-0.610 38](javascript:showCorrelationPlot2(db='MA_M2M_0706_R',ProbeSetID='1426900_at',CellID='',db2='MA_M2M_0706_R',ProbeSetID2='1460420_a_at',CellID2='',rank='1')) | [-0.407 38](javascript:showCorrelationPlot2(db='MA_M2M_0706_R',ProbeSetID='1426900_at',CellID='',db2='MA_M2M_0706_R',ProbeSetID2='1451530_at',CellID2='',rank='1')) | [0.743 38](javascript:showCorrelationPlot2(db='MA_M2M_0706_R',ProbeSetID='1426900_at',CellID='',db2='MA_M2M_0706_R',ProbeSetID2='1423785_at',CellID2='',rank='1')) | [-0.549 38](javascript:showCorrelationPlot2(db='MA_M2M_0706_R',ProbeSetID='1426900_at',CellID='',db2='MA_M2M_0706_R',ProbeSetID2='1431972_a_at',CellID2='',rank='1')) | [0.634 38](javascript:showCorrelationPlot2(db='MA_M2M_0706_R',ProbeSetID='1426900_at',CellID='',db2='MA_M2M_0706_R',ProbeSetID2='1448347_a_at',CellID2='',rank='1')) | [-0.507 38](javascript:showCorrelationPlot2(db='MA_M2M_0706_R',ProbeSetID='1426900_at',CellID='',db2='MA_M2M_0706_R',ProbeSetID2='1418492_at',CellID2='',rank='1')) | [0.558 38](javascript:showCorrelationPlot2(db='MA_M2M_0706_R',ProbeSetID='1426900_at',CellID='',db2='MA_M2M_0706_R',ProbeSetID2='1427185_at',CellID2='',rank='1')) | [0.452 38](javascript:showCorrelationPlot2(db='MA_M2M_0706_R',ProbeSetID='1426900_at',CellID='',db2='MA_M2M_0706_R',ProbeSetID2='1430500_s_at',CellID2='',rank='1')) | [-0.436 38](javascript:showCorrelationPlot2(db='MA_M2M_0706_R',ProbeSetID='1426900_at',CellID='',db2='MA_M2M_0706_R',ProbeSetID2='1447549_x_at',CellID2='',rank='1')) | [0.679 38](javascript:showCorrelationPlot2(db='MA_M2M_0706_R',ProbeSetID='1426900_at',CellID='',db2='MA_M2M_0706_R',ProbeSetID2='1448943_at',CellID2='',rank='1')) | [-0.564 38](javascript:showCorrelationPlot2(db='MA_M2M_0706_R',ProbeSetID='1426900_at',CellID='',db2='MA_M2M_0706_R',ProbeSetID2='1433162_at',CellID2='',rank='1')) | [-0.494 38](javascript:showCorrelationPlot2(db='MA_M2M_0706_R',ProbeSetID='1426900_at',CellID='',db2='MA_M2M_0706_R',ProbeSetID2='1443494_at',CellID2='',rank='1')) | [0.524 38](javascript:showCorrelationPlot2(db='MA_M2M_0706_R',ProbeSetID='1426900_at',CellID='',db2='MA_M2M_0706_R',ProbeSetID2='1417426_at',CellID2='',rank='1')) | [-0.635 38](javascript:showCorrelationPlot2(db='MA_M2M_0706_R',ProbeSetID='1426900_at',CellID='',db2='MA_M2M_0706_R',ProbeSetID2='1416588_at',CellID2='',rank='1')) | [0.660 38](javascript:showCorrelationPlot2(db='MA_M2M_0706_R',ProbeSetID='1426900_at',CellID='',db2='MA_M2M_0706_R',ProbeSetID2='1450994_at',CellID2='',rank='1')) | [-0.532 38](javascript:showCorrelationPlot2(db='MA_M2M_0706_R',ProbeSetID='1426900_at',CellID='',db2='MA_M2M_0706_R',ProbeSetID2='1420304_x_at',CellID2='',rank='1')) | [0.539 38](javascript:showCorrelationPlot2(db='MA_M2M_0706_R',ProbeSetID='1426900_at',CellID='',db2='MA_M2M_0706_R',ProbeSetID2='1416500_at',CellID2='',rank='1')) | [-0.538 38](javascript:showCorrelationPlot2(db='MA_M2M_0706_R',ProbeSetID='1426900_at',CellID='',db2='MA_M2M_0706_R',ProbeSetID2='1424199_at',CellID2='',rank='1')) | [0.720 38](javascript:showCorrelationPlot2(db='MA_M2M_0706_R',ProbeSetID='1426900_at',CellID='',db2='MA_M2M_0706_R',ProbeSetID2='1437995_x_at',CellID2='',rank='1')) | [0.508 38](javascript:showCorrelationPlot2(db='MA_M2M_0706_R',ProbeSetID='1426900_at',CellID='',db2='MA_M2M_0706_R',ProbeSetID2='1427914_a_at',CellID2='',rank='1')) | [-0.490 38](javascript:showCorrelationPlot2(db='MA_M2M_0706_R',ProbeSetID='1426900_at',CellID='',db2='MA_M2M_0706_R',ProbeSetID2='1420570_x_at',CellID2='',rank='1')) | [-0.588 38](javascript:showCorrelationPlot2(db='MA_M2M_0706_R',ProbeSetID='1426900_at',CellID='',db2='MA_M2M_0706_R',ProbeSetID2='1431015_at',CellID2='',rank='1')) | [0.138 38](javascript:showCorrelationPlot2(db='MA_M2M_0706_R',ProbeSetID='1426900_at',CellID='',db2='MA_M2M_0706_R',ProbeSetID2='1423852_at',CellID2='',rank='1')) | [-0.368 38](javascript:showCorrelationPlot2(db='MA_M2M_0706_R',ProbeSetID='1426900_at',CellID='',db2='MA_M2M_0706_R',ProbeSetID2='1431799_at',CellID2='',rank='1')) | [0.695 38](javascript:showCorrelationPlot2(db='MA_M2M_0706_R',ProbeSetID='1426900_at',CellID='',db2='MA_M2M_0706_R',ProbeSetID2='1423898_a_at',CellID2='',rank='1')) | [-0.464 38](javascript:showCorrelationPlot2(db='MA_M2M_0706_R',ProbeSetID='1426900_at',CellID='',db2='MA_M2M_0706_R',ProbeSetID2='1438579_at',CellID2='',rank='1')) | [0.470 38](javascript:showCorrelationPlot2(db='MA_M2M_0706_R',ProbeSetID='1426900_at',CellID='',db2='MA_M2M_0706_R',ProbeSetID2='1448102_a_at',CellID2='',rank='1')) | [-0.260 38](javascript:showCorrelationPlot2(db='MA_M2M_0706_R',ProbeSetID='1426900_at',CellID='',db2='MA_M2M_0706_R',ProbeSetID2='1420943_at',CellID2='',rank='1')) | [-0.375 38](javascript:showCorrelationPlot2(db='MA_M2M_0706_R',ProbeSetID='1426900_at',CellID='',db2='MA_M2M_0706_R',ProbeSetID2='1447326_s_at',CellID2='',rank='1')) |
|  | [Trait 5: MA_M2M_0706_R::1434888_a_at](javascript:showDatabase2('MA_M2M_0706_R','1434888_a_at','');)  Matr3 on Chr 18 @ 35.750609 Mb  matrin 3 (nuclear matrix protein, fibrogranular network)  mid to distal 3' UTR | [0.417 38](javascript:showCorrelationPlot2(db='MA_M2M_0706_R',ProbeSetID='1434888_a_at',CellID='',db2='MA_M2M_0706_R',ProbeSetID2='1445101_at',CellID2='',rank='0')) | [-0.457 38](javascript:showCorrelationPlot2(db='MA_M2M_0706_R',ProbeSetID='1434888_a_at',CellID='',db2='MA_M2M_0706_R',ProbeSetID2='1451285_at',CellID2='',rank='0')) | [-0.464 38](javascript:showCorrelationPlot2(db='MA_M2M_0706_R',ProbeSetID='1434888_a_at',CellID='',db2='MA_M2M_0706_R',ProbeSetID2='1441347_at',CellID2='',rank='0')) | [0.620 38](javascript:showCorrelationPlot2(db='MA_M2M_0706_R',ProbeSetID='1434888_a_at',CellID='',db2='MA_M2M_0706_R',ProbeSetID2='1426900_at',CellID2='',rank='0')) | [n 38](javascript:showDatabase2('MA_M2M_0706_R','1434888_a_at','')) | [0.534 38](javascript:showCorrelationPlot2(db='MA_M2M_0706_R',ProbeSetID='1434888_a_at',CellID='',db2='MA_M2M_0706_R',ProbeSetID2='1434773_a_at',CellID2='',rank='1')) | [-0.459 38](javascript:showCorrelationPlot2(db='MA_M2M_0706_R',ProbeSetID='1434888_a_at',CellID='',db2='MA_M2M_0706_R',ProbeSetID2='1452851_at',CellID2='',rank='1')) | [0.518 38](javascript:showCorrelationPlot2(db='MA_M2M_0706_R',ProbeSetID='1434888_a_at',CellID='',db2='MA_M2M_0706_R',ProbeSetID2='1441948_x_at',CellID2='',rank='1')) | [-0.639 38](javascript:showCorrelationPlot2(db='MA_M2M_0706_R',ProbeSetID='1434888_a_at',CellID='',db2='MA_M2M_0706_R',ProbeSetID2='1440160_x_at',CellID2='',rank='1')) | [0.511 38](javascript:showCorrelationPlot2(db='MA_M2M_0706_R',ProbeSetID='1434888_a_at',CellID='',db2='MA_M2M_0706_R',ProbeSetID2='1424692_at',CellID2='',rank='1')) | [-0.303 38](javascript:showCorrelationPlot2(db='MA_M2M_0706_R',ProbeSetID='1434888_a_at',CellID='',db2='MA_M2M_0706_R',ProbeSetID2='1433112_at',CellID2='',rank='1')) | [0.353 38](javascript:showCorrelationPlot2(db='MA_M2M_0706_R',ProbeSetID='1434888_a_at',CellID='',db2='MA_M2M_0706_R',ProbeSetID2='1459962_at',CellID2='',rank='1')) | [-0.309 38](javascript:showCorrelationPlot2(db='MA_M2M_0706_R',ProbeSetID='1434888_a_at',CellID='',db2='MA_M2M_0706_R',ProbeSetID2='1455331_at',CellID2='',rank='1')) | [-0.443 38](javascript:showCorrelationPlot2(db='MA_M2M_0706_R',ProbeSetID='1434888_a_at',CellID='',db2='MA_M2M_0706_R',ProbeSetID2='1447399_at',CellID2='',rank='1')) | [0.676 38](javascript:showCorrelationPlot2(db='MA_M2M_0706_R',ProbeSetID='1434888_a_at',CellID='',db2='MA_M2M_0706_R',ProbeSetID2='1434968_a_at',CellID2='',rank='1')) | [-0.418 38](javascript:showCorrelationPlot2(db='MA_M2M_0706_R',ProbeSetID='1434888_a_at',CellID='',db2='MA_M2M_0706_R',ProbeSetID2='1447335_x_at',CellID2='',rank='1')) | [-0.522 38](javascript:showCorrelationPlot2(db='MA_M2M_0706_R',ProbeSetID='1434888_a_at',CellID='',db2='MA_M2M_0706_R',ProbeSetID2='1442239_at',CellID2='',rank='1')) | [-0.657 38](javascript:showCorrelationPlot2(db='MA_M2M_0706_R',ProbeSetID='1434888_a_at',CellID='',db2='MA_M2M_0706_R',ProbeSetID2='1459865_x_at',CellID2='',rank='1')) | [0.589 38](javascript:showCorrelationPlot2(db='MA_M2M_0706_R',ProbeSetID='1434888_a_at',CellID='',db2='MA_M2M_0706_R',ProbeSetID2='1438647_x_at',CellID2='',rank='1')) | [-0.424 38](javascript:showCorrelationPlot2(db='MA_M2M_0706_R',ProbeSetID='1434888_a_at',CellID='',db2='MA_M2M_0706_R',ProbeSetID2='1432333_a_at',CellID2='',rank='1')) | [0.493 38](javascript:showCorrelationPlot2(db='MA_M2M_0706_R',ProbeSetID='1434888_a_at',CellID='',db2='MA_M2M_0706_R',ProbeSetID2='1444028_s_at',CellID2='',rank='1')) | [-0.558 38](javascript:showCorrelationPlot2(db='MA_M2M_0706_R',ProbeSetID='1434888_a_at',CellID='',db2='MA_M2M_0706_R',ProbeSetID2='1460420_a_at',CellID2='',rank='1')) | [-0.355 38](javascript:showCorrelationPlot2(db='MA_M2M_0706_R',ProbeSetID='1434888_a_at',CellID='',db2='MA_M2M_0706_R',ProbeSetID2='1451530_at',CellID2='',rank='1')) | [0.523 38](javascript:showCorrelationPlot2(db='MA_M2M_0706_R',ProbeSetID='1434888_a_at',CellID='',db2='MA_M2M_0706_R',ProbeSetID2='1423785_at',CellID2='',rank='1')) | [-0.658 38](javascript:showCorrelationPlot2(db='MA_M2M_0706_R',ProbeSetID='1434888_a_at',CellID='',db2='MA_M2M_0706_R',ProbeSetID2='1431972_a_at',CellID2='',rank='1')) | [0.416 38](javascript:showCorrelationPlot2(db='MA_M2M_0706_R',ProbeSetID='1434888_a_at',CellID='',db2='MA_M2M_0706_R',ProbeSetID2='1448347_a_at',CellID2='',rank='1')) | [-0.457 38](javascript:showCorrelationPlot2(db='MA_M2M_0706_R',ProbeSetID='1434888_a_at',CellID='',db2='MA_M2M_0706_R',ProbeSetID2='1418492_at',CellID2='',rank='1')) | [0.576 38](javascript:showCorrelationPlot2(db='MA_M2M_0706_R',ProbeSetID='1434888_a_at',CellID='',db2='MA_M2M_0706_R',ProbeSetID2='1427185_at',CellID2='',rank='1')) | [0.562 38](javascript:showCorrelationPlot2(db='MA_M2M_0706_R',ProbeSetID='1434888_a_at',CellID='',db2='MA_M2M_0706_R',ProbeSetID2='1430500_s_at',CellID2='',rank='1')) | [-0.390 38](javascript:showCorrelationPlot2(db='MA_M2M_0706_R',ProbeSetID='1434888_a_at',CellID='',db2='MA_M2M_0706_R',ProbeSetID2='1447549_x_at',CellID2='',rank='1')) | [0.412 38](javascript:showCorrelationPlot2(db='MA_M2M_0706_R',ProbeSetID='1434888_a_at',CellID='',db2='MA_M2M_0706_R',ProbeSetID2='1448943_at',CellID2='',rank='1')) | [-0.440 38](javascript:showCorrelationPlot2(db='MA_M2M_0706_R',ProbeSetID='1434888_a_at',CellID='',db2='MA_M2M_0706_R',ProbeSetID2='1433162_at',CellID2='',rank='1')) | [-0.484 38](javascript:showCorrelationPlot2(db='MA_M2M_0706_R',ProbeSetID='1434888_a_at',CellID='',db2='MA_M2M_0706_R',ProbeSetID2='1443494_at',CellID2='',rank='1')) | [0.391 38](javascript:showCorrelationPlot2(db='MA_M2M_0706_R',ProbeSetID='1434888_a_at',CellID='',db2='MA_M2M_0706_R',ProbeSetID2='1417426_at',CellID2='',rank='1')) | [-0.552 38](javascript:showCorrelationPlot2(db='MA_M2M_0706_R',ProbeSetID='1434888_a_at',CellID='',db2='MA_M2M_0706_R',ProbeSetID2='1416588_at',CellID2='',rank='1')) | [0.468 38](javascript:showCorrelationPlot2(db='MA_M2M_0706_R',ProbeSetID='1434888_a_at',CellID='',db2='MA_M2M_0706_R',ProbeSetID2='1450994_at',CellID2='',rank='1')) | [-0.585 38](javascript:showCorrelationPlot2(db='MA_M2M_0706_R',ProbeSetID='1434888_a_at',CellID='',db2='MA_M2M_0706_R',ProbeSetID2='1420304_x_at',CellID2='',rank='1')) | [0.506 38](javascript:showCorrelationPlot2(db='MA_M2M_0706_R',ProbeSetID='1434888_a_at',CellID='',db2='MA_M2M_0706_R',ProbeSetID2='1416500_at',CellID2='',rank='1')) | [-0.528 38](javascript:showCorrelationPlot2(db='MA_M2M_0706_R',ProbeSetID='1434888_a_at',CellID='',db2='MA_M2M_0706_R',ProbeSetID2='1424199_at',CellID2='',rank='1')) | [0.575 38](javascript:showCorrelationPlot2(db='MA_M2M_0706_R',ProbeSetID='1434888_a_at',CellID='',db2='MA_M2M_0706_R',ProbeSetID2='1437995_x_at',CellID2='',rank='1')) | [0.413 38](javascript:showCorrelationPlot2(db='MA_M2M_0706_R',ProbeSetID='1434888_a_at',CellID='',db2='MA_M2M_0706_R',ProbeSetID2='1427914_a_at',CellID2='',rank='1')) | [-0.648 38](javascript:showCorrelationPlot2(db='MA_M2M_0706_R',ProbeSetID='1434888_a_at',CellID='',db2='MA_M2M_0706_R',ProbeSetID2='1420570_x_at',CellID2='',rank='1')) | [-0.672 38](javascript:showCorrelationPlot2(db='MA_M2M_0706_R',ProbeSetID='1434888_a_at',CellID='',db2='MA_M2M_0706_R',ProbeSetID2='1431015_at',CellID2='',rank='1')) | [0.337 38](javascript:showCorrelationPlot2(db='MA_M2M_0706_R',ProbeSetID='1434888_a_at',CellID='',db2='MA_M2M_0706_R',ProbeSetID2='1423852_at',CellID2='',rank='1')) | [-0.412 38](javascript:showCorrelationPlot2(db='MA_M2M_0706_R',ProbeSetID='1434888_a_at',CellID='',db2='MA_M2M_0706_R',ProbeSetID2='1431799_at',CellID2='',rank='1')) | [0.608 38](javascript:showCorrelationPlot2(db='MA_M2M_0706_R',ProbeSetID='1434888_a_at',CellID='',db2='MA_M2M_0706_R',ProbeSetID2='1423898_a_at',CellID2='',rank='1')) | [-0.470 38](javascript:showCorrelationPlot2(db='MA_M2M_0706_R',ProbeSetID='1434888_a_at',CellID='',db2='MA_M2M_0706_R',ProbeSetID2='1438579_at',CellID2='',rank='1')) | [0.660 38](javascript:showCorrelationPlot2(db='MA_M2M_0706_R',ProbeSetID='1434888_a_at',CellID='',db2='MA_M2M_0706_R',ProbeSetID2='1448102_a_at',CellID2='',rank='1')) | [-0.370 38](javascript:showCorrelationPlot2(db='MA_M2M_0706_R',ProbeSetID='1434888_a_at',CellID='',db2='MA_M2M_0706_R',ProbeSetID2='1420943_at',CellID2='',rank='1')) | [-0.439 38](javascript:showCorrelationPlot2(db='MA_M2M_0706_R',ProbeSetID='1434888_a_at',CellID='',db2='MA_M2M_0706_R',ProbeSetID2='1447326_s_at',CellID2='',rank='1')) |
|  | [Trait 6: MA_M2M_0706_R::1434773_a_at](javascript:showDatabase2('MA_M2M_0706_R','1434773_a_at','');)  Slc2a1 on Chr 4 @ 118.809716 Mb  solute carrier family 2 (facilitated glucose transporter), member 1 | [0.473 38](javascript:showCorrelationPlot2(db='MA_M2M_0706_R',ProbeSetID='1434773_a_at',CellID='',db2='MA_M2M_0706_R',ProbeSetID2='1445101_at',CellID2='',rank='0')) | [-0.458 38](javascript:showCorrelationPlot2(db='MA_M2M_0706_R',ProbeSetID='1434773_a_at',CellID='',db2='MA_M2M_0706_R',ProbeSetID2='1451285_at',CellID2='',rank='0')) | [-0.438 38](javascript:showCorrelationPlot2(db='MA_M2M_0706_R',ProbeSetID='1434773_a_at',CellID='',db2='MA_M2M_0706_R',ProbeSetID2='1441347_at',CellID2='',rank='0')) | [0.459 38](javascript:showCorrelationPlot2(db='MA_M2M_0706_R',ProbeSetID='1434773_a_at',CellID='',db2='MA_M2M_0706_R',ProbeSetID2='1426900_at',CellID2='',rank='0')) | [0.560 38](javascript:showCorrelationPlot2(db='MA_M2M_0706_R',ProbeSetID='1434773_a_at',CellID='',db2='MA_M2M_0706_R',ProbeSetID2='1434888_a_at',CellID2='',rank='0')) | [n 38](javascript:showDatabase2('MA_M2M_0706_R','1434773_a_at','')) | [-0.510 38](javascript:showCorrelationPlot2(db='MA_M2M_0706_R',ProbeSetID='1434773_a_at',CellID='',db2='MA_M2M_0706_R',ProbeSetID2='1452851_at',CellID2='',rank='1')) | [0.570 38](javascript:showCorrelationPlot2(db='MA_M2M_0706_R',ProbeSetID='1434773_a_at',CellID='',db2='MA_M2M_0706_R',ProbeSetID2='1441948_x_at',CellID2='',rank='1')) | [-0.508 38](javascript:showCorrelationPlot2(db='MA_M2M_0706_R',ProbeSetID='1434773_a_at',CellID='',db2='MA_M2M_0706_R',ProbeSetID2='1440160_x_at',CellID2='',rank='1')) | [0.570 38](javascript:showCorrelationPlot2(db='MA_M2M_0706_R',ProbeSetID='1434773_a_at',CellID='',db2='MA_M2M_0706_R',ProbeSetID2='1424692_at',CellID2='',rank='1')) | [-0.587 38](javascript:showCorrelationPlot2(db='MA_M2M_0706_R',ProbeSetID='1434773_a_at',CellID='',db2='MA_M2M_0706_R',ProbeSetID2='1433112_at',CellID2='',rank='1')) | [0.537 38](javascript:showCorrelationPlot2(db='MA_M2M_0706_R',ProbeSetID='1434773_a_at',CellID='',db2='MA_M2M_0706_R',ProbeSetID2='1459962_at',CellID2='',rank='1')) | [-0.340 38](javascript:showCorrelationPlot2(db='MA_M2M_0706_R',ProbeSetID='1434773_a_at',CellID='',db2='MA_M2M_0706_R',ProbeSetID2='1455331_at',CellID2='',rank='1')) | [-0.426 38](javascript:showCorrelationPlot2(db='MA_M2M_0706_R',ProbeSetID='1434773_a_at',CellID='',db2='MA_M2M_0706_R',ProbeSetID2='1447399_at',CellID2='',rank='1')) | [0.435 38](javascript:showCorrelationPlot2(db='MA_M2M_0706_R',ProbeSetID='1434773_a_at',CellID='',db2='MA_M2M_0706_R',ProbeSetID2='1434968_a_at',CellID2='',rank='1')) | [-0.491 38](javascript:showCorrelationPlot2(db='MA_M2M_0706_R',ProbeSetID='1434773_a_at',CellID='',db2='MA_M2M_0706_R',ProbeSetID2='1447335_x_at',CellID2='',rank='1')) | [-0.486 38](javascript:showCorrelationPlot2(db='MA_M2M_0706_R',ProbeSetID='1434773_a_at',CellID='',db2='MA_M2M_0706_R',ProbeSetID2='1442239_at',CellID2='',rank='1')) | [-0.520 38](javascript:showCorrelationPlot2(db='MA_M2M_0706_R',ProbeSetID='1434773_a_at',CellID='',db2='MA_M2M_0706_R',ProbeSetID2='1459865_x_at',CellID2='',rank='1')) | [0.553 38](javascript:showCorrelationPlot2(db='MA_M2M_0706_R',ProbeSetID='1434773_a_at',CellID='',db2='MA_M2M_0706_R',ProbeSetID2='1438647_x_at',CellID2='',rank='1')) | [-0.549 38](javascript:showCorrelationPlot2(db='MA_M2M_0706_R',ProbeSetID='1434773_a_at',CellID='',db2='MA_M2M_0706_R',ProbeSetID2='1432333_a_at',CellID2='',rank='1')) | [0.458 38](javascript:showCorrelationPlot2(db='MA_M2M_0706_R',ProbeSetID='1434773_a_at',CellID='',db2='MA_M2M_0706_R',ProbeSetID2='1444028_s_at',CellID2='',rank='1')) | [-0.628 38](javascript:showCorrelationPlot2(db='MA_M2M_0706_R',ProbeSetID='1434773_a_at',CellID='',db2='MA_M2M_0706_R',ProbeSetID2='1460420_a_at',CellID2='',rank='1')) | [-0.566 38](javascript:showCorrelationPlot2(db='MA_M2M_0706_R',ProbeSetID='1434773_a_at',CellID='',db2='MA_M2M_0706_R',ProbeSetID2='1451530_at',CellID2='',rank='1')) | [0.392 38](javascript:showCorrelationPlot2(db='MA_M2M_0706_R',ProbeSetID='1434773_a_at',CellID='',db2='MA_M2M_0706_R',ProbeSetID2='1423785_at',CellID2='',rank='1')) | [-0.455 38](javascript:showCorrelationPlot2(db='MA_M2M_0706_R',ProbeSetID='1434773_a_at',CellID='',db2='MA_M2M_0706_R',ProbeSetID2='1431972_a_at',CellID2='',rank='1')) | [0.593 38](javascript:showCorrelationPlot2(db='MA_M2M_0706_R',ProbeSetID='1434773_a_at',CellID='',db2='MA_M2M_0706_R',ProbeSetID2='1448347_a_at',CellID2='',rank='1')) | [-0.424 38](javascript:showCorrelationPlot2(db='MA_M2M_0706_R',ProbeSetID='1434773_a_at',CellID='',db2='MA_M2M_0706_R',ProbeSetID2='1418492_at',CellID2='',rank='1')) | [0.531 38](javascript:showCorrelationPlot2(db='MA_M2M_0706_R',ProbeSetID='1434773_a_at',CellID='',db2='MA_M2M_0706_R',ProbeSetID2='1427185_at',CellID2='',rank='1')) | [0.669 38](javascript:showCorrelationPlot2(db='MA_M2M_0706_R',ProbeSetID='1434773_a_at',CellID='',db2='MA_M2M_0706_R',ProbeSetID2='1430500_s_at',CellID2='',rank='1')) | [-0.423 38](javascript:showCorrelationPlot2(db='MA_M2M_0706_R',ProbeSetID='1434773_a_at',CellID='',db2='MA_M2M_0706_R',ProbeSetID2='1447549_x_at',CellID2='',rank='1')) | [0.497 38](javascript:showCorrelationPlot2(db='MA_M2M_0706_R',ProbeSetID='1434773_a_at',CellID='',db2='MA_M2M_0706_R',ProbeSetID2='1448943_at',CellID2='',rank='1')) | [-0.538 38](javascript:showCorrelationPlot2(db='MA_M2M_0706_R',ProbeSetID='1434773_a_at',CellID='',db2='MA_M2M_0706_R',ProbeSetID2='1433162_at',CellID2='',rank='1')) | [-0.419 38](javascript:showCorrelationPlot2(db='MA_M2M_0706_R',ProbeSetID='1434773_a_at',CellID='',db2='MA_M2M_0706_R',ProbeSetID2='1443494_at',CellID2='',rank='1')) | [0.519 38](javascript:showCorrelationPlot2(db='MA_M2M_0706_R',ProbeSetID='1434773_a_at',CellID='',db2='MA_M2M_0706_R',ProbeSetID2='1417426_at',CellID2='',rank='1')) | [-0.674 38](javascript:showCorrelationPlot2(db='MA_M2M_0706_R',ProbeSetID='1434773_a_at',CellID='',db2='MA_M2M_0706_R',ProbeSetID2='1416588_at',CellID2='',rank='1')) | [0.616 38](javascript:showCorrelationPlot2(db='MA_M2M_0706_R',ProbeSetID='1434773_a_at',CellID='',db2='MA_M2M_0706_R',ProbeSetID2='1450994_at',CellID2='',rank='1')) | [-0.714 38](javascript:showCorrelationPlot2(db='MA_M2M_0706_R',ProbeSetID='1434773_a_at',CellID='',db2='MA_M2M_0706_R',ProbeSetID2='1420304_x_at',CellID2='',rank='1')) | [0.578 38](javascript:showCorrelationPlot2(db='MA_M2M_0706_R',ProbeSetID='1434773_a_at',CellID='',db2='MA_M2M_0706_R',ProbeSetID2='1416500_at',CellID2='',rank='1')) | [-0.533 38](javascript:showCorrelationPlot2(db='MA_M2M_0706_R',ProbeSetID='1434773_a_at',CellID='',db2='MA_M2M_0706_R',ProbeSetID2='1424199_at',CellID2='',rank='1')) | [0.690 38](javascript:showCorrelationPlot2(db='MA_M2M_0706_R',ProbeSetID='1434773_a_at',CellID='',db2='MA_M2M_0706_R',ProbeSetID2='1437995_x_at',CellID2='',rank='1')) | [0.585 38](javascript:showCorrelationPlot2(db='MA_M2M_0706_R',ProbeSetID='1434773_a_at',CellID='',db2='MA_M2M_0706_R',ProbeSetID2='1427914_a_at',CellID2='',rank='1')) | [-0.618 38](javascript:showCorrelationPlot2(db='MA_M2M_0706_R',ProbeSetID='1434773_a_at',CellID='',db2='MA_M2M_0706_R',ProbeSetID2='1420570_x_at',CellID2='',rank='1')) | [-0.495 38](javascript:showCorrelationPlot2(db='MA_M2M_0706_R',ProbeSetID='1434773_a_at',CellID='',db2='MA_M2M_0706_R',ProbeSetID2='1431015_at',CellID2='',rank='1')) | [0.593 38](javascript:showCorrelationPlot2(db='MA_M2M_0706_R',ProbeSetID='1434773_a_at',CellID='',db2='MA_M2M_0706_R',ProbeSetID2='1423852_at',CellID2='',rank='1')) | [-0.504 38](javascript:showCorrelationPlot2(db='MA_M2M_0706_R',ProbeSetID='1434773_a_at',CellID='',db2='MA_M2M_0706_R',ProbeSetID2='1431799_at',CellID2='',rank='1')) | [0.664 38](javascript:showCorrelationPlot2(db='MA_M2M_0706_R',ProbeSetID='1434773_a_at',CellID='',db2='MA_M2M_0706_R',ProbeSetID2='1423898_a_at',CellID2='',rank='1')) | [-0.516 38](javascript:showCorrelationPlot2(db='MA_M2M_0706_R',ProbeSetID='1434773_a_at',CellID='',db2='MA_M2M_0706_R',ProbeSetID2='1438579_at',CellID2='',rank='1')) | [0.642 38](javascript:showCorrelationPlot2(db='MA_M2M_0706_R',ProbeSetID='1434773_a_at',CellID='',db2='MA_M2M_0706_R',ProbeSetID2='1448102_a_at',CellID2='',rank='1')) | [-0.643 38](javascript:showCorrelationPlot2(db='MA_M2M_0706_R',ProbeSetID='1434773_a_at',CellID='',db2='MA_M2M_0706_R',ProbeSetID2='1420943_at',CellID2='',rank='1')) | [-0.646 38](javascript:showCorrelationPlot2(db='MA_M2M_0706_R',ProbeSetID='1434773_a_at',CellID='',db2='MA_M2M_0706_R',ProbeSetID2='1447326_s_at',CellID2='',rank='1')) |
|  | [Trait 7: MA_M2M_0706_R::1452851_at](javascript:showDatabase2('MA_M2M_0706_R','1452851_at','');)  Tnrc4 on Chr 3 @ 94.295527 Mb  trinucleotide repeat containing 4 | [-0.513 38](javascript:showCorrelationPlot2(db='MA_M2M_0706_R',ProbeSetID='1452851_at',CellID='',db2='MA_M2M_0706_R',ProbeSetID2='1445101_at',CellID2='',rank='0')) | [0.355 38](javascript:showCorrelationPlot2(db='MA_M2M_0706_R',ProbeSetID='1452851_at',CellID='',db2='MA_M2M_0706_R',ProbeSetID2='1451285_at',CellID2='',rank='0')) | [0.619 38](javascript:showCorrelationPlot2(db='MA_M2M_0706_R',ProbeSetID='1452851_at',CellID='',db2='MA_M2M_0706_R',ProbeSetID2='1441347_at',CellID2='',rank='0')) | [-0.487 38](javascript:showCorrelationPlot2(db='MA_M2M_0706_R',ProbeSetID='1452851_at',CellID='',db2='MA_M2M_0706_R',ProbeSetID2='1426900_at',CellID2='',rank='0')) | [-0.523 38](javascript:showCorrelationPlot2(db='MA_M2M_0706_R',ProbeSetID='1452851_at',CellID='',db2='MA_M2M_0706_R',ProbeSetID2='1434888_a_at',CellID2='',rank='0')) | [-0.542 38](javascript:showCorrelationPlot2(db='MA_M2M_0706_R',ProbeSetID='1452851_at',CellID='',db2='MA_M2M_0706_R',ProbeSetID2='1434773_a_at',CellID2='',rank='0')) | [n 38](javascript:showDatabase2('MA_M2M_0706_R','1452851_at','')) | [-0.478 38](javascript:showCorrelationPlot2(db='MA_M2M_0706_R',ProbeSetID='1452851_at',CellID='',db2='MA_M2M_0706_R',ProbeSetID2='1441948_x_at',CellID2='',rank='1')) | [0.520 38](javascript:showCorrelationPlot2(db='MA_M2M_0706_R',ProbeSetID='1452851_at',CellID='',db2='MA_M2M_0706_R',ProbeSetID2='1440160_x_at',CellID2='',rank='1')) | [-0.621 38](javascript:showCorrelationPlot2(db='MA_M2M_0706_R',ProbeSetID='1452851_at',CellID='',db2='MA_M2M_0706_R',ProbeSetID2='1424692_at',CellID2='',rank='1')) | [0.530 38](javascript:showCorrelationPlot2(db='MA_M2M_0706_R',ProbeSetID='1452851_at',CellID='',db2='MA_M2M_0706_R',ProbeSetID2='1433112_at',CellID2='',rank='1')) | [-0.373 38](javascript:showCorrelationPlot2(db='MA_M2M_0706_R',ProbeSetID='1452851_at',CellID='',db2='MA_M2M_0706_R',ProbeSetID2='1459962_at',CellID2='',rank='1')) | [0.488 38](javascript:showCorrelationPlot2(db='MA_M2M_0706_R',ProbeSetID='1452851_at',CellID='',db2='MA_M2M_0706_R',ProbeSetID2='1455331_at',CellID2='',rank='1')) | [0.475 38](javascript:showCorrelationPlot2(db='MA_M2M_0706_R',ProbeSetID='1452851_at',CellID='',db2='MA_M2M_0706_R',ProbeSetID2='1447399_at',CellID2='',rank='1')) | [-0.329 38](javascript:showCorrelationPlot2(db='MA_M2M_0706_R',ProbeSetID='1452851_at',CellID='',db2='MA_M2M_0706_R',ProbeSetID2='1434968_a_at',CellID2='',rank='1')) | [0.738 38](javascript:showCorrelationPlot2(db='MA_M2M_0706_R',ProbeSetID='1452851_at',CellID='',db2='MA_M2M_0706_R',ProbeSetID2='1447335_x_at',CellID2='',rank='1')) | [0.632 38](javascript:showCorrelationPlot2(db='MA_M2M_0706_R',ProbeSetID='1452851_at',CellID='',db2='MA_M2M_0706_R',ProbeSetID2='1442239_at',CellID2='',rank='1')) | [0.516 38](javascript:showCorrelationPlot2(db='MA_M2M_0706_R',ProbeSetID='1452851_at',CellID='',db2='MA_M2M_0706_R',ProbeSetID2='1459865_x_at',CellID2='',rank='1')) | [-0.335 38](javascript:showCorrelationPlot2(db='MA_M2M_0706_R',ProbeSetID='1452851_at',CellID='',db2='MA_M2M_0706_R',ProbeSetID2='1438647_x_at',CellID2='',rank='1')) | [0.525 38](javascript:showCorrelationPlot2(db='MA_M2M_0706_R',ProbeSetID='1452851_at',CellID='',db2='MA_M2M_0706_R',ProbeSetID2='1432333_a_at',CellID2='',rank='1')) | [-0.442 38](javascript:showCorrelationPlot2(db='MA_M2M_0706_R',ProbeSetID='1452851_at',CellID='',db2='MA_M2M_0706_R',ProbeSetID2='1444028_s_at',CellID2='',rank='1')) | [0.593 38](javascript:showCorrelationPlot2(db='MA_M2M_0706_R',ProbeSetID='1452851_at',CellID='',db2='MA_M2M_0706_R',ProbeSetID2='1460420_a_at',CellID2='',rank='1')) | [0.537 38](javascript:showCorrelationPlot2(db='MA_M2M_0706_R',ProbeSetID='1452851_at',CellID='',db2='MA_M2M_0706_R',ProbeSetID2='1451530_at',CellID2='',rank='1')) | [-0.564 38](javascript:showCorrelationPlot2(db='MA_M2M_0706_R',ProbeSetID='1452851_at',CellID='',db2='MA_M2M_0706_R',ProbeSetID2='1423785_at',CellID2='',rank='1')) | [0.278 38](javascript:showCorrelationPlot2(db='MA_M2M_0706_R',ProbeSetID='1452851_at',CellID='',db2='MA_M2M_0706_R',ProbeSetID2='1431972_a_at',CellID2='',rank='1')) | [-0.475 38](javascript:showCorrelationPlot2(db='MA_M2M_0706_R',ProbeSetID='1452851_at',CellID='',db2='MA_M2M_0706_R',ProbeSetID2='1448347_a_at',CellID2='',rank='1')) | [0.461 38](javascript:showCorrelationPlot2(db='MA_M2M_0706_R',ProbeSetID='1452851_at',CellID='',db2='MA_M2M_0706_R',ProbeSetID2='1418492_at',CellID2='',rank='1')) | [-0.703 38](javascript:showCorrelationPlot2(db='MA_M2M_0706_R',ProbeSetID='1452851_at',CellID='',db2='MA_M2M_0706_R',ProbeSetID2='1427185_at',CellID2='',rank='1')) | [-0.432 38](javascript:showCorrelationPlot2(db='MA_M2M_0706_R',ProbeSetID='1452851_at',CellID='',db2='MA_M2M_0706_R',ProbeSetID2='1430500_s_at',CellID2='',rank='1')) | [0.483 38](javascript:showCorrelationPlot2(db='MA_M2M_0706_R',ProbeSetID='1452851_at',CellID='',db2='MA_M2M_0706_R',ProbeSetID2='1447549_x_at',CellID2='',rank='1')) | [-0.578 38](javascript:showCorrelationPlot2(db='MA_M2M_0706_R',ProbeSetID='1452851_at',CellID='',db2='MA_M2M_0706_R',ProbeSetID2='1448943_at',CellID2='',rank='1')) | [0.630 38](javascript:showCorrelationPlot2(db='MA_M2M_0706_R',ProbeSetID='1452851_at',CellID='',db2='MA_M2M_0706_R',ProbeSetID2='1433162_at',CellID2='',rank='1')) | [0.511 38](javascript:showCorrelationPlot2(db='MA_M2M_0706_R',ProbeSetID='1452851_at',CellID='',db2='MA_M2M_0706_R',ProbeSetID2='1443494_at',CellID2='',rank='1')) | [-0.598 38](javascript:showCorrelationPlot2(db='MA_M2M_0706_R',ProbeSetID='1452851_at',CellID='',db2='MA_M2M_0706_R',ProbeSetID2='1417426_at',CellID2='',rank='1')) | [0.645 38](javascript:showCorrelationPlot2(db='MA_M2M_0706_R',ProbeSetID='1452851_at',CellID='',db2='MA_M2M_0706_R',ProbeSetID2='1416588_at',CellID2='',rank='1')) | [-0.470 38](javascript:showCorrelationPlot2(db='MA_M2M_0706_R',ProbeSetID='1452851_at',CellID='',db2='MA_M2M_0706_R',ProbeSetID2='1450994_at',CellID2='',rank='1')) | [0.494 38](javascript:showCorrelationPlot2(db='MA_M2M_0706_R',ProbeSetID='1452851_at',CellID='',db2='MA_M2M_0706_R',ProbeSetID2='1420304_x_at',CellID2='',rank='1')) | [-0.600 38](javascript:showCorrelationPlot2(db='MA_M2M_0706_R',ProbeSetID='1452851_at',CellID='',db2='MA_M2M_0706_R',ProbeSetID2='1416500_at',CellID2='',rank='1')) | [0.499 38](javascript:showCorrelationPlot2(db='MA_M2M_0706_R',ProbeSetID='1452851_at',CellID='',db2='MA_M2M_0706_R',ProbeSetID2='1424199_at',CellID2='',rank='1')) | [-0.491 38](javascript:showCorrelationPlot2(db='MA_M2M_0706_R',ProbeSetID='1452851_at',CellID='',db2='MA_M2M_0706_R',ProbeSetID2='1437995_x_at',CellID2='',rank='1')) | [-0.450 38](javascript:showCorrelationPlot2(db='MA_M2M_0706_R',ProbeSetID='1452851_at',CellID='',db2='MA_M2M_0706_R',ProbeSetID2='1427914_a_at',CellID2='',rank='1')) | [0.547 38](javascript:showCorrelationPlot2(db='MA_M2M_0706_R',ProbeSetID='1452851_at',CellID='',db2='MA_M2M_0706_R',ProbeSetID2='1420570_x_at',CellID2='',rank='1')) | [0.539 38](javascript:showCorrelationPlot2(db='MA_M2M_0706_R',ProbeSetID='1452851_at',CellID='',db2='MA_M2M_0706_R',ProbeSetID2='1431015_at',CellID2='',rank='1')) | [-0.512 38](javascript:showCorrelationPlot2(db='MA_M2M_0706_R',ProbeSetID='1452851_at',CellID='',db2='MA_M2M_0706_R',ProbeSetID2='1423852_at',CellID2='',rank='1')) | [0.336 38](javascript:showCorrelationPlot2(db='MA_M2M_0706_R',ProbeSetID='1452851_at',CellID='',db2='MA_M2M_0706_R',ProbeSetID2='1431799_at',CellID2='',rank='1')) | [-0.516 38](javascript:showCorrelationPlot2(db='MA_M2M_0706_R',ProbeSetID='1452851_at',CellID='',db2='MA_M2M_0706_R',ProbeSetID2='1423898_a_at',CellID2='',rank='1')) | [0.668 38](javascript:showCorrelationPlot2(db='MA_M2M_0706_R',ProbeSetID='1452851_at',CellID='',db2='MA_M2M_0706_R',ProbeSetID2='1438579_at',CellID2='',rank='1')) | [-0.492 38](javascript:showCorrelationPlot2(db='MA_M2M_0706_R',ProbeSetID='1452851_at',CellID='',db2='MA_M2M_0706_R',ProbeSetID2='1448102_a_at',CellID2='',rank='1')) | [0.338 38](javascript:showCorrelationPlot2(db='MA_M2M_0706_R',ProbeSetID='1452851_at',CellID='',db2='MA_M2M_0706_R',ProbeSetID2='1420943_at',CellID2='',rank='1')) | [0.407 38](javascript:showCorrelationPlot2(db='MA_M2M_0706_R',ProbeSetID='1452851_at',CellID='',db2='MA_M2M_0706_R',ProbeSetID2='1447326_s_at',CellID2='',rank='1')) |
|  | [Trait 8: MA_M2M_0706_R::1441948_x_at](javascript:showDatabase2('MA_M2M_0706_R','1441948_x_at','');)  Zfand3 on Chr 17 @ 30.346839 Mb  zinc finger, AN1-type domain 3  distal 3' UTR | [0.580 38](javascript:showCorrelationPlot2(db='MA_M2M_0706_R',ProbeSetID='1441948_x_at',CellID='',db2='MA_M2M_0706_R',ProbeSetID2='1445101_at',CellID2='',rank='0')) | [-0.516 38](javascript:showCorrelationPlot2(db='MA_M2M_0706_R',ProbeSetID='1441948_x_at',CellID='',db2='MA_M2M_0706_R',ProbeSetID2='1451285_at',CellID2='',rank='0')) | [-0.650 38](javascript:showCorrelationPlot2(db='MA_M2M_0706_R',ProbeSetID='1441948_x_at',CellID='',db2='MA_M2M_0706_R',ProbeSetID2='1441347_at',CellID2='',rank='0')) | [0.454 38](javascript:showCorrelationPlot2(db='MA_M2M_0706_R',ProbeSetID='1441948_x_at',CellID='',db2='MA_M2M_0706_R',ProbeSetID2='1426900_at',CellID2='',rank='0')) | [0.595 38](javascript:showCorrelationPlot2(db='MA_M2M_0706_R',ProbeSetID='1441948_x_at',CellID='',db2='MA_M2M_0706_R',ProbeSetID2='1434888_a_at',CellID2='',rank='0')) | [0.585 38](javascript:showCorrelationPlot2(db='MA_M2M_0706_R',ProbeSetID='1441948_x_at',CellID='',db2='MA_M2M_0706_R',ProbeSetID2='1434773_a_at',CellID2='',rank='0')) | [-0.547 38](javascript:showCorrelationPlot2(db='MA_M2M_0706_R',ProbeSetID='1441948_x_at',CellID='',db2='MA_M2M_0706_R',ProbeSetID2='1452851_at',CellID2='',rank='0')) | [n 38](javascript:showDatabase2('MA_M2M_0706_R','1441948_x_at','')) | [-0.567 38](javascript:showCorrelationPlot2(db='MA_M2M_0706_R',ProbeSetID='1441948_x_at',CellID='',db2='MA_M2M_0706_R',ProbeSetID2='1440160_x_at',CellID2='',rank='1')) | [0.425 38](javascript:showCorrelationPlot2(db='MA_M2M_0706_R',ProbeSetID='1441948_x_at',CellID='',db2='MA_M2M_0706_R',ProbeSetID2='1424692_at',CellID2='',rank='1')) | [-0.544 38](javascript:showCorrelationPlot2(db='MA_M2M_0706_R',ProbeSetID='1441948_x_at',CellID='',db2='MA_M2M_0706_R',ProbeSetID2='1433112_at',CellID2='',rank='1')) | [0.601 38](javascript:showCorrelationPlot2(db='MA_M2M_0706_R',ProbeSetID='1441948_x_at',CellID='',db2='MA_M2M_0706_R',ProbeSetID2='1459962_at',CellID2='',rank='1')) | [-0.607 38](javascript:showCorrelationPlot2(db='MA_M2M_0706_R',ProbeSetID='1441948_x_at',CellID='',db2='MA_M2M_0706_R',ProbeSetID2='1455331_at',CellID2='',rank='1')) | [-0.665 38](javascript:showCorrelationPlot2(db='MA_M2M_0706_R',ProbeSetID='1441948_x_at',CellID='',db2='MA_M2M_0706_R',ProbeSetID2='1447399_at',CellID2='',rank='1')) | [0.344 38](javascript:showCorrelationPlot2(db='MA_M2M_0706_R',ProbeSetID='1441948_x_at',CellID='',db2='MA_M2M_0706_R',ProbeSetID2='1434968_a_at',CellID2='',rank='1')) | [-0.476 38](javascript:showCorrelationPlot2(db='MA_M2M_0706_R',ProbeSetID='1441948_x_at',CellID='',db2='MA_M2M_0706_R',ProbeSetID2='1447335_x_at',CellID2='',rank='1')) | [-0.650 38](javascript:showCorrelationPlot2(db='MA_M2M_0706_R',ProbeSetID='1441948_x_at',CellID='',db2='MA_M2M_0706_R',ProbeSetID2='1442239_at',CellID2='',rank='1')) | [-0.344 38](javascript:showCorrelationPlot2(db='MA_M2M_0706_R',ProbeSetID='1441948_x_at',CellID='',db2='MA_M2M_0706_R',ProbeSetID2='1459865_x_at',CellID2='',rank='1')) | [0.529 38](javascript:showCorrelationPlot2(db='MA_M2M_0706_R',ProbeSetID='1441948_x_at',CellID='',db2='MA_M2M_0706_R',ProbeSetID2='1438647_x_at',CellID2='',rank='1')) | [-0.556 38](javascript:showCorrelationPlot2(db='MA_M2M_0706_R',ProbeSetID='1441948_x_at',CellID='',db2='MA_M2M_0706_R',ProbeSetID2='1432333_a_at',CellID2='',rank='1')) | [0.405 38](javascript:showCorrelationPlot2(db='MA_M2M_0706_R',ProbeSetID='1441948_x_at',CellID='',db2='MA_M2M_0706_R',ProbeSetID2='1444028_s_at',CellID2='',rank='1')) | [-0.543 38](javascript:showCorrelationPlot2(db='MA_M2M_0706_R',ProbeSetID='1441948_x_at',CellID='',db2='MA_M2M_0706_R',ProbeSetID2='1460420_a_at',CellID2='',rank='1')) | [-0.455 38](javascript:showCorrelationPlot2(db='MA_M2M_0706_R',ProbeSetID='1441948_x_at',CellID='',db2='MA_M2M_0706_R',ProbeSetID2='1451530_at',CellID2='',rank='1')) | [0.388 38](javascript:showCorrelationPlot2(db='MA_M2M_0706_R',ProbeSetID='1441948_x_at',CellID='',db2='MA_M2M_0706_R',ProbeSetID2='1423785_at',CellID2='',rank='1')) | [-0.271 38](javascript:showCorrelationPlot2(db='MA_M2M_0706_R',ProbeSetID='1441948_x_at',CellID='',db2='MA_M2M_0706_R',ProbeSetID2='1431972_a_at',CellID2='',rank='1')) | [0.495 38](javascript:showCorrelationPlot2(db='MA_M2M_0706_R',ProbeSetID='1441948_x_at',CellID='',db2='MA_M2M_0706_R',ProbeSetID2='1448347_a_at',CellID2='',rank='1')) | [-0.407 38](javascript:showCorrelationPlot2(db='MA_M2M_0706_R',ProbeSetID='1441948_x_at',CellID='',db2='MA_M2M_0706_R',ProbeSetID2='1418492_at',CellID2='',rank='1')) | [0.533 38](javascript:showCorrelationPlot2(db='MA_M2M_0706_R',ProbeSetID='1441948_x_at',CellID='',db2='MA_M2M_0706_R',ProbeSetID2='1427185_at',CellID2='',rank='1')) | [0.428 38](javascript:showCorrelationPlot2(db='MA_M2M_0706_R',ProbeSetID='1441948_x_at',CellID='',db2='MA_M2M_0706_R',ProbeSetID2='1430500_s_at',CellID2='',rank='1')) | [-0.372 38](javascript:showCorrelationPlot2(db='MA_M2M_0706_R',ProbeSetID='1441948_x_at',CellID='',db2='MA_M2M_0706_R',ProbeSetID2='1447549_x_at',CellID2='',rank='1')) | [0.319 38](javascript:showCorrelationPlot2(db='MA_M2M_0706_R',ProbeSetID='1441948_x_at',CellID='',db2='MA_M2M_0706_R',ProbeSetID2='1448943_at',CellID2='',rank='1')) | [-0.422 38](javascript:showCorrelationPlot2(db='MA_M2M_0706_R',ProbeSetID='1441948_x_at',CellID='',db2='MA_M2M_0706_R',ProbeSetID2='1433162_at',CellID2='',rank='1')) | [-0.388 38](javascript:showCorrelationPlot2(db='MA_M2M_0706_R',ProbeSetID='1441948_x_at',CellID='',db2='MA_M2M_0706_R',ProbeSetID2='1443494_at',CellID2='',rank='1')) | [0.467 38](javascript:showCorrelationPlot2(db='MA_M2M_0706_R',ProbeSetID='1441948_x_at',CellID='',db2='MA_M2M_0706_R',ProbeSetID2='1417426_at',CellID2='',rank='1')) | [-0.555 38](javascript:showCorrelationPlot2(db='MA_M2M_0706_R',ProbeSetID='1441948_x_at',CellID='',db2='MA_M2M_0706_R',ProbeSetID2='1416588_at',CellID2='',rank='1')) | [0.281 38](javascript:showCorrelationPlot2(db='MA_M2M_0706_R',ProbeSetID='1441948_x_at',CellID='',db2='MA_M2M_0706_R',ProbeSetID2='1450994_at',CellID2='',rank='1')) | [-0.593 38](javascript:showCorrelationPlot2(db='MA_M2M_0706_R',ProbeSetID='1441948_x_at',CellID='',db2='MA_M2M_0706_R',ProbeSetID2='1420304_x_at',CellID2='',rank='1')) | [0.556 38](javascript:showCorrelationPlot2(db='MA_M2M_0706_R',ProbeSetID='1441948_x_at',CellID='',db2='MA_M2M_0706_R',ProbeSetID2='1416500_at',CellID2='',rank='1')) | [-0.677 38](javascript:showCorrelationPlot2(db='MA_M2M_0706_R',ProbeSetID='1441948_x_at',CellID='',db2='MA_M2M_0706_R',ProbeSetID2='1424199_at',CellID2='',rank='1')) | [0.453 38](javascript:showCorrelationPlot2(db='MA_M2M_0706_R',ProbeSetID='1441948_x_at',CellID='',db2='MA_M2M_0706_R',ProbeSetID2='1437995_x_at',CellID2='',rank='1')) | [0.431 38](javascript:showCorrelationPlot2(db='MA_M2M_0706_R',ProbeSetID='1441948_x_at',CellID='',db2='MA_M2M_0706_R',ProbeSetID2='1427914_a_at',CellID2='',rank='1')) | [-0.510 38](javascript:showCorrelationPlot2(db='MA_M2M_0706_R',ProbeSetID='1441948_x_at',CellID='',db2='MA_M2M_0706_R',ProbeSetID2='1420570_x_at',CellID2='',rank='1')) | [-0.544 38](javascript:showCorrelationPlot2(db='MA_M2M_0706_R',ProbeSetID='1441948_x_at',CellID='',db2='MA_M2M_0706_R',ProbeSetID2='1431015_at',CellID2='',rank='1')) | [0.513 38](javascript:showCorrelationPlot2(db='MA_M2M_0706_R',ProbeSetID='1441948_x_at',CellID='',db2='MA_M2M_0706_R',ProbeSetID2='1423852_at',CellID2='',rank='1')) | [-0.182 38](javascript:showCorrelationPlot2(db='MA_M2M_0706_R',ProbeSetID='1441948_x_at',CellID='',db2='MA_M2M_0706_R',ProbeSetID2='1431799_at',CellID2='',rank='1')) | [0.519 38](javascript:showCorrelationPlot2(db='MA_M2M_0706_R',ProbeSetID='1441948_x_at',CellID='',db2='MA_M2M_0706_R',ProbeSetID2='1423898_a_at',CellID2='',rank='1')) | [-0.569 38](javascript:showCorrelationPlot2(db='MA_M2M_0706_R',ProbeSetID='1441948_x_at',CellID='',db2='MA_M2M_0706_R',ProbeSetID2='1438579_at',CellID2='',rank='1')) | [0.663 38](javascript:showCorrelationPlot2(db='MA_M2M_0706_R',ProbeSetID='1441948_x_at',CellID='',db2='MA_M2M_0706_R',ProbeSetID2='1448102_a_at',CellID2='',rank='1')) | [-0.666 38](javascript:showCorrelationPlot2(db='MA_M2M_0706_R',ProbeSetID='1441948_x_at',CellID='',db2='MA_M2M_0706_R',ProbeSetID2='1420943_at',CellID2='',rank='1')) | [-0.549 38](javascript:showCorrelationPlot2(db='MA_M2M_0706_R',ProbeSetID='1441948_x_at',CellID='',db2='MA_M2M_0706_R',ProbeSetID2='1447326_s_at',CellID2='',rank='1')) |
|  | [Trait 9: MA_M2M_0706_R::1440160_x_at](javascript:showDatabase2('MA_M2M_0706_R','1440160_x_at','');)  2010321M09Rik on Chr 9 @ 64.834554 Mb  RIKEN cDNA 2010321M09 gene  distal 3' UTR | [-0.746 38](javascript:showCorrelationPlot2(db='MA_M2M_0706_R',ProbeSetID='1440160_x_at',CellID='',db2='MA_M2M_0706_R',ProbeSetID2='1445101_at',CellID2='',rank='0')) | [0.304 38](javascript:showCorrelationPlot2(db='MA_M2M_0706_R',ProbeSetID='1440160_x_at',CellID='',db2='MA_M2M_0706_R',ProbeSetID2='1451285_at',CellID2='',rank='0')) | [0.622 38](javascript:showCorrelationPlot2(db='MA_M2M_0706_R',ProbeSetID='1440160_x_at',CellID='',db2='MA_M2M_0706_R',ProbeSetID2='1441347_at',CellID2='',rank='0')) | [-0.644 38](javascript:showCorrelationPlot2(db='MA_M2M_0706_R',ProbeSetID='1440160_x_at',CellID='',db2='MA_M2M_0706_R',ProbeSetID2='1426900_at',CellID2='',rank='0')) | [-0.607 38](javascript:showCorrelationPlot2(db='MA_M2M_0706_R',ProbeSetID='1440160_x_at',CellID='',db2='MA_M2M_0706_R',ProbeSetID2='1434888_a_at',CellID2='',rank='0')) | [-0.486 38](javascript:showCorrelationPlot2(db='MA_M2M_0706_R',ProbeSetID='1440160_x_at',CellID='',db2='MA_M2M_0706_R',ProbeSetID2='1434773_a_at',CellID2='',rank='0')) | [0.659 38](javascript:showCorrelationPlot2(db='MA_M2M_0706_R',ProbeSetID='1440160_x_at',CellID='',db2='MA_M2M_0706_R',ProbeSetID2='1452851_at',CellID2='',rank='0')) | [-0.600 38](javascript:showCorrelationPlot2(db='MA_M2M_0706_R',ProbeSetID='1440160_x_at',CellID='',db2='MA_M2M_0706_R',ProbeSetID2='1441948_x_at',CellID2='',rank='0')) | [n 38](javascript:showDatabase2('MA_M2M_0706_R','1440160_x_at','')) | [-0.718 38](javascript:showCorrelationPlot2(db='MA_M2M_0706_R',ProbeSetID='1440160_x_at',CellID='',db2='MA_M2M_0706_R',ProbeSetID2='1424692_at',CellID2='',rank='1')) | [0.522 38](javascript:showCorrelationPlot2(db='MA_M2M_0706_R',ProbeSetID='1440160_x_at',CellID='',db2='MA_M2M_0706_R',ProbeSetID2='1433112_at',CellID2='',rank='1')) | [-0.404 38](javascript:showCorrelationPlot2(db='MA_M2M_0706_R',ProbeSetID='1440160_x_at',CellID='',db2='MA_M2M_0706_R',ProbeSetID2='1459962_at',CellID2='',rank='1')) | [0.569 38](javascript:showCorrelationPlot2(db='MA_M2M_0706_R',ProbeSetID='1440160_x_at',CellID='',db2='MA_M2M_0706_R',ProbeSetID2='1455331_at',CellID2='',rank='1')) | [0.674 38](javascript:showCorrelationPlot2(db='MA_M2M_0706_R',ProbeSetID='1440160_x_at',CellID='',db2='MA_M2M_0706_R',ProbeSetID2='1447399_at',CellID2='',rank='1')) | [-0.586 38](javascript:showCorrelationPlot2(db='MA_M2M_0706_R',ProbeSetID='1440160_x_at',CellID='',db2='MA_M2M_0706_R',ProbeSetID2='1434968_a_at',CellID2='',rank='1')) | [0.649 38](javascript:showCorrelationPlot2(db='MA_M2M_0706_R',ProbeSetID='1440160_x_at',CellID='',db2='MA_M2M_0706_R',ProbeSetID2='1447335_x_at',CellID2='',rank='1')) | [0.691 38](javascript:showCorrelationPlot2(db='MA_M2M_0706_R',ProbeSetID='1440160_x_at',CellID='',db2='MA_M2M_0706_R',ProbeSetID2='1442239_at',CellID2='',rank='1')) | [0.751 38](javascript:showCorrelationPlot2(db='MA_M2M_0706_R',ProbeSetID='1440160_x_at',CellID='',db2='MA_M2M_0706_R',ProbeSetID2='1459865_x_at',CellID2='',rank='1')) | [-0.524 38](javascript:showCorrelationPlot2(db='MA_M2M_0706_R',ProbeSetID='1440160_x_at',CellID='',db2='MA_M2M_0706_R',ProbeSetID2='1438647_x_at',CellID2='',rank='1')) | [0.449 38](javascript:showCorrelationPlot2(db='MA_M2M_0706_R',ProbeSetID='1440160_x_at',CellID='',db2='MA_M2M_0706_R',ProbeSetID2='1432333_a_at',CellID2='',rank='1')) | [-0.280 38](javascript:showCorrelationPlot2(db='MA_M2M_0706_R',ProbeSetID='1440160_x_at',CellID='',db2='MA_M2M_0706_R',ProbeSetID2='1444028_s_at',CellID2='',rank='1')) | [0.603 38](javascript:showCorrelationPlot2(db='MA_M2M_0706_R',ProbeSetID='1440160_x_at',CellID='',db2='MA_M2M_0706_R',ProbeSetID2='1460420_a_at',CellID2='',rank='1')) | [0.358 38](javascript:showCorrelationPlot2(db='MA_M2M_0706_R',ProbeSetID='1440160_x_at',CellID='',db2='MA_M2M_0706_R',ProbeSetID2='1451530_at',CellID2='',rank='1')) | [-0.525 38](javascript:showCorrelationPlot2(db='MA_M2M_0706_R',ProbeSetID='1440160_x_at',CellID='',db2='MA_M2M_0706_R',ProbeSetID2='1423785_at',CellID2='',rank='1')) | [0.434 38](javascript:showCorrelationPlot2(db='MA_M2M_0706_R',ProbeSetID='1440160_x_at',CellID='',db2='MA_M2M_0706_R',ProbeSetID2='1431972_a_at',CellID2='',rank='1')) | [-0.381 38](javascript:showCorrelationPlot2(db='MA_M2M_0706_R',ProbeSetID='1440160_x_at',CellID='',db2='MA_M2M_0706_R',ProbeSetID2='1448347_a_at',CellID2='',rank='1')) | [0.637 38](javascript:showCorrelationPlot2(db='MA_M2M_0706_R',ProbeSetID='1440160_x_at',CellID='',db2='MA_M2M_0706_R',ProbeSetID2='1418492_at',CellID2='',rank='1')) | [-0.465 38](javascript:showCorrelationPlot2(db='MA_M2M_0706_R',ProbeSetID='1440160_x_at',CellID='',db2='MA_M2M_0706_R',ProbeSetID2='1427185_at',CellID2='',rank='1')) | [-0.530 38](javascript:showCorrelationPlot2(db='MA_M2M_0706_R',ProbeSetID='1440160_x_at',CellID='',db2='MA_M2M_0706_R',ProbeSetID2='1430500_s_at',CellID2='',rank='1')) | [0.490 38](javascript:showCorrelationPlot2(db='MA_M2M_0706_R',ProbeSetID='1440160_x_at',CellID='',db2='MA_M2M_0706_R',ProbeSetID2='1447549_x_at',CellID2='',rank='1')) | [-0.379 38](javascript:showCorrelationPlot2(db='MA_M2M_0706_R',ProbeSetID='1440160_x_at',CellID='',db2='MA_M2M_0706_R',ProbeSetID2='1448943_at',CellID2='',rank='1')) | [0.672 38](javascript:showCorrelationPlot2(db='MA_M2M_0706_R',ProbeSetID='1440160_x_at',CellID='',db2='MA_M2M_0706_R',ProbeSetID2='1433162_at',CellID2='',rank='1')) | [0.568 38](javascript:showCorrelationPlot2(db='MA_M2M_0706_R',ProbeSetID='1440160_x_at',CellID='',db2='MA_M2M_0706_R',ProbeSetID2='1443494_at',CellID2='',rank='1')) | [-0.357 38](javascript:showCorrelationPlot2(db='MA_M2M_0706_R',ProbeSetID='1440160_x_at',CellID='',db2='MA_M2M_0706_R',ProbeSetID2='1417426_at',CellID2='',rank='1')) | [0.673 38](javascript:showCorrelationPlot2(db='MA_M2M_0706_R',ProbeSetID='1440160_x_at',CellID='',db2='MA_M2M_0706_R',ProbeSetID2='1416588_at',CellID2='',rank='1')) | [-0.455 38](javascript:showCorrelationPlot2(db='MA_M2M_0706_R',ProbeSetID='1440160_x_at',CellID='',db2='MA_M2M_0706_R',ProbeSetID2='1450994_at',CellID2='',rank='1')) | [0.539 38](javascript:showCorrelationPlot2(db='MA_M2M_0706_R',ProbeSetID='1440160_x_at',CellID='',db2='MA_M2M_0706_R',ProbeSetID2='1420304_x_at',CellID2='',rank='1')) | [-0.489 38](javascript:showCorrelationPlot2(db='MA_M2M_0706_R',ProbeSetID='1440160_x_at',CellID='',db2='MA_M2M_0706_R',ProbeSetID2='1416500_at',CellID2='',rank='1')) | [0.701 38](javascript:showCorrelationPlot2(db='MA_M2M_0706_R',ProbeSetID='1440160_x_at',CellID='',db2='MA_M2M_0706_R',ProbeSetID2='1424199_at',CellID2='',rank='1')) | [-0.445 38](javascript:showCorrelationPlot2(db='MA_M2M_0706_R',ProbeSetID='1440160_x_at',CellID='',db2='MA_M2M_0706_R',ProbeSetID2='1437995_x_at',CellID2='',rank='1')) | [-0.457 38](javascript:showCorrelationPlot2(db='MA_M2M_0706_R',ProbeSetID='1440160_x_at',CellID='',db2='MA_M2M_0706_R',ProbeSetID2='1427914_a_at',CellID2='',rank='1')) | [0.754 38](javascript:showCorrelationPlot2(db='MA_M2M_0706_R',ProbeSetID='1440160_x_at',CellID='',db2='MA_M2M_0706_R',ProbeSetID2='1420570_x_at',CellID2='',rank='1')) | [0.535 38](javascript:showCorrelationPlot2(db='MA_M2M_0706_R',ProbeSetID='1440160_x_at',CellID='',db2='MA_M2M_0706_R',ProbeSetID2='1431015_at',CellID2='',rank='1')) | [-0.462 38](javascript:showCorrelationPlot2(db='MA_M2M_0706_R',ProbeSetID='1440160_x_at',CellID='',db2='MA_M2M_0706_R',ProbeSetID2='1423852_at',CellID2='',rank='1')) | [0.346 38](javascript:showCorrelationPlot2(db='MA_M2M_0706_R',ProbeSetID='1440160_x_at',CellID='',db2='MA_M2M_0706_R',ProbeSetID2='1431799_at',CellID2='',rank='1')) | [-0.549 38](javascript:showCorrelationPlot2(db='MA_M2M_0706_R',ProbeSetID='1440160_x_at',CellID='',db2='MA_M2M_0706_R',ProbeSetID2='1423898_a_at',CellID2='',rank='1')) | [0.618 38](javascript:showCorrelationPlot2(db='MA_M2M_0706_R',ProbeSetID='1440160_x_at',CellID='',db2='MA_M2M_0706_R',ProbeSetID2='1438579_at',CellID2='',rank='1')) | [-0.534 38](javascript:showCorrelationPlot2(db='MA_M2M_0706_R',ProbeSetID='1440160_x_at',CellID='',db2='MA_M2M_0706_R',ProbeSetID2='1448102_a_at',CellID2='',rank='1')) | [0.442 38](javascript:showCorrelationPlot2(db='MA_M2M_0706_R',ProbeSetID='1440160_x_at',CellID='',db2='MA_M2M_0706_R',ProbeSetID2='1420943_at',CellID2='',rank='1')) | [0.494 38](javascript:showCorrelationPlot2(db='MA_M2M_0706_R',ProbeSetID='1440160_x_at',CellID='',db2='MA_M2M_0706_R',ProbeSetID2='1447326_s_at',CellID2='',rank='1')) |
|  | [Trait 10: MA_M2M_0706_R::1424692_at](javascript:showDatabase2('MA_M2M_0706_R','1424692_at','');)  2810055F11Rik on Chr 12 @ 73.174543 Mb  RIKEN cDNA 2810055F11 gene | [0.634 38](javascript:showCorrelationPlot2(db='MA_M2M_0706_R',ProbeSetID='1424692_at',CellID='',db2='MA_M2M_0706_R',ProbeSetID2='1445101_at',CellID2='',rank='0')) | [-0.231 38](javascript:showCorrelationPlot2(db='MA_M2M_0706_R',ProbeSetID='1424692_at',CellID='',db2='MA_M2M_0706_R',ProbeSetID2='1451285_at',CellID2='',rank='0')) | [-0.424 38](javascript:showCorrelationPlot2(db='MA_M2M_0706_R',ProbeSetID='1424692_at',CellID='',db2='MA_M2M_0706_R',ProbeSetID2='1441347_at',CellID2='',rank='0')) | [0.554 38](javascript:showCorrelationPlot2(db='MA_M2M_0706_R',ProbeSetID='1424692_at',CellID='',db2='MA_M2M_0706_R',ProbeSetID2='1426900_at',CellID2='',rank='0')) | [0.561 38](javascript:showCorrelationPlot2(db='MA_M2M_0706_R',ProbeSetID='1424692_at',CellID='',db2='MA_M2M_0706_R',ProbeSetID2='1434888_a_at',CellID2='',rank='0')) | [0.540 38](javascript:showCorrelationPlot2(db='MA_M2M_0706_R',ProbeSetID='1424692_at',CellID='',db2='MA_M2M_0706_R',ProbeSetID2='1434773_a_at',CellID2='',rank='0')) | [-0.626 38](javascript:showCorrelationPlot2(db='MA_M2M_0706_R',ProbeSetID='1424692_at',CellID='',db2='MA_M2M_0706_R',ProbeSetID2='1452851_at',CellID2='',rank='0')) | [0.492 38](javascript:showCorrelationPlot2(db='MA_M2M_0706_R',ProbeSetID='1424692_at',CellID='',db2='MA_M2M_0706_R',ProbeSetID2='1441948_x_at',CellID2='',rank='0')) | [-0.728 38](javascript:showCorrelationPlot2(db='MA_M2M_0706_R',ProbeSetID='1424692_at',CellID='',db2='MA_M2M_0706_R',ProbeSetID2='1440160_x_at',CellID2='',rank='0')) | [n 38](javascript:showDatabase2('MA_M2M_0706_R','1424692_at','')) | [-0.455 38](javascript:showCorrelationPlot2(db='MA_M2M_0706_R',ProbeSetID='1424692_at',CellID='',db2='MA_M2M_0706_R',ProbeSetID2='1433112_at',CellID2='',rank='1')) | [0.282 38](javascript:showCorrelationPlot2(db='MA_M2M_0706_R',ProbeSetID='1424692_at',CellID='',db2='MA_M2M_0706_R',ProbeSetID2='1459962_at',CellID2='',rank='1')) | [-0.336 38](javascript:showCorrelationPlot2(db='MA_M2M_0706_R',ProbeSetID='1424692_at',CellID='',db2='MA_M2M_0706_R',ProbeSetID2='1455331_at',CellID2='',rank='1')) | [-0.474 38](javascript:showCorrelationPlot2(db='MA_M2M_0706_R',ProbeSetID='1424692_at',CellID='',db2='MA_M2M_0706_R',ProbeSetID2='1447399_at',CellID2='',rank='1')) | [0.564 38](javascript:showCorrelationPlot2(db='MA_M2M_0706_R',ProbeSetID='1424692_at',CellID='',db2='MA_M2M_0706_R',ProbeSetID2='1434968_a_at',CellID2='',rank='1')) | [-0.488 38](javascript:showCorrelationPlot2(db='MA_M2M_0706_R',ProbeSetID='1424692_at',CellID='',db2='MA_M2M_0706_R',ProbeSetID2='1447335_x_at',CellID2='',rank='1')) | [-0.441 38](javascript:showCorrelationPlot2(db='MA_M2M_0706_R',ProbeSetID='1424692_at',CellID='',db2='MA_M2M_0706_R',ProbeSetID2='1442239_at',CellID2='',rank='1')) | [-0.607 38](javascript:showCorrelationPlot2(db='MA_M2M_0706_R',ProbeSetID='1424692_at',CellID='',db2='MA_M2M_0706_R',ProbeSetID2='1459865_x_at',CellID2='',rank='1')) | [0.509 38](javascript:showCorrelationPlot2(db='MA_M2M_0706_R',ProbeSetID='1424692_at',CellID='',db2='MA_M2M_0706_R',ProbeSetID2='1438647_x_at',CellID2='',rank='1')) | [-0.465 38](javascript:showCorrelationPlot2(db='MA_M2M_0706_R',ProbeSetID='1424692_at',CellID='',db2='MA_M2M_0706_R',ProbeSetID2='1432333_a_at',CellID2='',rank='1')) | [0.277 38](javascript:showCorrelationPlot2(db='MA_M2M_0706_R',ProbeSetID='1424692_at',CellID='',db2='MA_M2M_0706_R',ProbeSetID2='1444028_s_at',CellID2='',rank='1')) | [-0.543 38](javascript:showCorrelationPlot2(db='MA_M2M_0706_R',ProbeSetID='1424692_at',CellID='',db2='MA_M2M_0706_R',ProbeSetID2='1460420_a_at',CellID2='',rank='1')) | [-0.272 38](javascript:showCorrelationPlot2(db='MA_M2M_0706_R',ProbeSetID='1424692_at',CellID='',db2='MA_M2M_0706_R',ProbeSetID2='1451530_at',CellID2='',rank='1')) | [0.488 38](javascript:showCorrelationPlot2(db='MA_M2M_0706_R',ProbeSetID='1424692_at',CellID='',db2='MA_M2M_0706_R',ProbeSetID2='1423785_at',CellID2='',rank='1')) | [-0.338 38](javascript:showCorrelationPlot2(db='MA_M2M_0706_R',ProbeSetID='1424692_at',CellID='',db2='MA_M2M_0706_R',ProbeSetID2='1431972_a_at',CellID2='',rank='1')) | [0.353 38](javascript:showCorrelationPlot2(db='MA_M2M_0706_R',ProbeSetID='1424692_at',CellID='',db2='MA_M2M_0706_R',ProbeSetID2='1448347_a_at',CellID2='',rank='1')) | [-0.392 38](javascript:showCorrelationPlot2(db='MA_M2M_0706_R',ProbeSetID='1424692_at',CellID='',db2='MA_M2M_0706_R',ProbeSetID2='1418492_at',CellID2='',rank='1')) | [0.499 38](javascript:showCorrelationPlot2(db='MA_M2M_0706_R',ProbeSetID='1424692_at',CellID='',db2='MA_M2M_0706_R',ProbeSetID2='1427185_at',CellID2='',rank='1')) | [0.500 38](javascript:showCorrelationPlot2(db='MA_M2M_0706_R',ProbeSetID='1424692_at',CellID='',db2='MA_M2M_0706_R',ProbeSetID2='1430500_s_at',CellID2='',rank='1')) | [-0.239 38](javascript:showCorrelationPlot2(db='MA_M2M_0706_R',ProbeSetID='1424692_at',CellID='',db2='MA_M2M_0706_R',ProbeSetID2='1447549_x_at',CellID2='',rank='1')) | [0.450 38](javascript:showCorrelationPlot2(db='MA_M2M_0706_R',ProbeSetID='1424692_at',CellID='',db2='MA_M2M_0706_R',ProbeSetID2='1448943_at',CellID2='',rank='1')) | [-0.594 38](javascript:showCorrelationPlot2(db='MA_M2M_0706_R',ProbeSetID='1424692_at',CellID='',db2='MA_M2M_0706_R',ProbeSetID2='1433162_at',CellID2='',rank='1')) | [-0.326 38](javascript:showCorrelationPlot2(db='MA_M2M_0706_R',ProbeSetID='1424692_at',CellID='',db2='MA_M2M_0706_R',ProbeSetID2='1443494_at',CellID2='',rank='1')) | [0.392 38](javascript:showCorrelationPlot2(db='MA_M2M_0706_R',ProbeSetID='1424692_at',CellID='',db2='MA_M2M_0706_R',ProbeSetID2='1417426_at',CellID2='',rank='1')) | [-0.666 38](javascript:showCorrelationPlot2(db='MA_M2M_0706_R',ProbeSetID='1424692_at',CellID='',db2='MA_M2M_0706_R',ProbeSetID2='1416588_at',CellID2='',rank='1')) | [0.456 38](javascript:showCorrelationPlot2(db='MA_M2M_0706_R',ProbeSetID='1424692_at',CellID='',db2='MA_M2M_0706_R',ProbeSetID2='1450994_at',CellID2='',rank='1')) | [-0.433 38](javascript:showCorrelationPlot2(db='MA_M2M_0706_R',ProbeSetID='1424692_at',CellID='',db2='MA_M2M_0706_R',ProbeSetID2='1420304_x_at',CellID2='',rank='1')) | [0.326 38](javascript:showCorrelationPlot2(db='MA_M2M_0706_R',ProbeSetID='1424692_at',CellID='',db2='MA_M2M_0706_R',ProbeSetID2='1416500_at',CellID2='',rank='1')) | [-0.405 38](javascript:showCorrelationPlot2(db='MA_M2M_0706_R',ProbeSetID='1424692_at',CellID='',db2='MA_M2M_0706_R',ProbeSetID2='1424199_at',CellID2='',rank='1')) | [0.445 38](javascript:showCorrelationPlot2(db='MA_M2M_0706_R',ProbeSetID='1424692_at',CellID='',db2='MA_M2M_0706_R',ProbeSetID2='1437995_x_at',CellID2='',rank='1')) | [0.419 38](javascript:showCorrelationPlot2(db='MA_M2M_0706_R',ProbeSetID='1424692_at',CellID='',db2='MA_M2M_0706_R',ProbeSetID2='1427914_a_at',CellID2='',rank='1')) | [-0.511 38](javascript:showCorrelationPlot2(db='MA_M2M_0706_R',ProbeSetID='1424692_at',CellID='',db2='MA_M2M_0706_R',ProbeSetID2='1420570_x_at',CellID2='',rank='1')) | [-0.282 38](javascript:showCorrelationPlot2(db='MA_M2M_0706_R',ProbeSetID='1424692_at',CellID='',db2='MA_M2M_0706_R',ProbeSetID2='1431015_at',CellID2='',rank='1')) | [0.415 38](javascript:showCorrelationPlot2(db='MA_M2M_0706_R',ProbeSetID='1424692_at',CellID='',db2='MA_M2M_0706_R',ProbeSetID2='1423852_at',CellID2='',rank='1')) | [-0.410 38](javascript:showCorrelationPlot2(db='MA_M2M_0706_R',ProbeSetID='1424692_at',CellID='',db2='MA_M2M_0706_R',ProbeSetID2='1431799_at',CellID2='',rank='1')) | [0.547 38](javascript:showCorrelationPlot2(db='MA_M2M_0706_R',ProbeSetID='1424692_at',CellID='',db2='MA_M2M_0706_R',ProbeSetID2='1423898_a_at',CellID2='',rank='1')) | [-0.504 38](javascript:showCorrelationPlot2(db='MA_M2M_0706_R',ProbeSetID='1424692_at',CellID='',db2='MA_M2M_0706_R',ProbeSetID2='1438579_at',CellID2='',rank='1')) | [0.433 38](javascript:showCorrelationPlot2(db='MA_M2M_0706_R',ProbeSetID='1424692_at',CellID='',db2='MA_M2M_0706_R',ProbeSetID2='1448102_a_at',CellID2='',rank='1')) | [-0.354 38](javascript:showCorrelationPlot2(db='MA_M2M_0706_R',ProbeSetID='1424692_at',CellID='',db2='MA_M2M_0706_R',ProbeSetID2='1420943_at',CellID2='',rank='1')) | [-0.338 38](javascript:showCorrelationPlot2(db='MA_M2M_0706_R',ProbeSetID='1424692_at',CellID='',db2='MA_M2M_0706_R',ProbeSetID2='1447326_s_at',CellID2='',rank='1')) |
|  | [Trait 11: MA_M2M_0706_R::1433112_at](javascript:showDatabase2('MA_M2M_0706_R','1433112_at','');)  4933424L07Rik on Chr 12 @ 107.397039 Mb  RIKEN cDNA 4933424L07 gene | [-0.690 38](javascript:showCorrelationPlot2(db='MA_M2M_0706_R',ProbeSetID='1433112_at',CellID='',db2='MA_M2M_0706_R',ProbeSetID2='1445101_at',CellID2='',rank='0')) | [0.373 38](javascript:showCorrelationPlot2(db='MA_M2M_0706_R',ProbeSetID='1433112_at',CellID='',db2='MA_M2M_0706_R',ProbeSetID2='1451285_at',CellID2='',rank='0')) | [0.679 38](javascript:showCorrelationPlot2(db='MA_M2M_0706_R',ProbeSetID='1433112_at',CellID='',db2='MA_M2M_0706_R',ProbeSetID2='1441347_at',CellID2='',rank='0')) | [-0.460 38](javascript:showCorrelationPlot2(db='MA_M2M_0706_R',ProbeSetID='1433112_at',CellID='',db2='MA_M2M_0706_R',ProbeSetID2='1426900_at',CellID2='',rank='0')) | [-0.407 38](javascript:showCorrelationPlot2(db='MA_M2M_0706_R',ProbeSetID='1433112_at',CellID='',db2='MA_M2M_0706_R',ProbeSetID2='1434888_a_at',CellID2='',rank='0')) | [-0.631 38](javascript:showCorrelationPlot2(db='MA_M2M_0706_R',ProbeSetID='1433112_at',CellID='',db2='MA_M2M_0706_R',ProbeSetID2='1434773_a_at',CellID2='',rank='0')) | [0.550 38](javascript:showCorrelationPlot2(db='MA_M2M_0706_R',ProbeSetID='1433112_at',CellID='',db2='MA_M2M_0706_R',ProbeSetID2='1452851_at',CellID2='',rank='0')) | [-0.600 38](javascript:showCorrelationPlot2(db='MA_M2M_0706_R',ProbeSetID='1433112_at',CellID='',db2='MA_M2M_0706_R',ProbeSetID2='1441948_x_at',CellID2='',rank='0')) | [0.609 38](javascript:showCorrelationPlot2(db='MA_M2M_0706_R',ProbeSetID='1433112_at',CellID='',db2='MA_M2M_0706_R',ProbeSetID2='1440160_x_at',CellID2='',rank='0')) | [-0.474 38](javascript:showCorrelationPlot2(db='MA_M2M_0706_R',ProbeSetID='1433112_at',CellID='',db2='MA_M2M_0706_R',ProbeSetID2='1424692_at',CellID2='',rank='0')) | [n 38](javascript:showDatabase2('MA_M2M_0706_R','1433112_at','')) | [-0.703 38](javascript:showCorrelationPlot2(db='MA_M2M_0706_R',ProbeSetID='1433112_at',CellID='',db2='MA_M2M_0706_R',ProbeSetID2='1459962_at',CellID2='',rank='1')) | [0.603 38](javascript:showCorrelationPlot2(db='MA_M2M_0706_R',ProbeSetID='1433112_at',CellID='',db2='MA_M2M_0706_R',ProbeSetID2='1455331_at',CellID2='',rank='1')) | [0.683 38](javascript:showCorrelationPlot2(db='MA_M2M_0706_R',ProbeSetID='1433112_at',CellID='',db2='MA_M2M_0706_R',ProbeSetID2='1447399_at',CellID2='',rank='1')) | [-0.409 38](javascript:showCorrelationPlot2(db='MA_M2M_0706_R',ProbeSetID='1433112_at',CellID='',db2='MA_M2M_0706_R',ProbeSetID2='1434968_a_at',CellID2='',rank='1')) | [0.605 38](javascript:showCorrelationPlot2(db='MA_M2M_0706_R',ProbeSetID='1433112_at',CellID='',db2='MA_M2M_0706_R',ProbeSetID2='1447335_x_at',CellID2='',rank='1')) | [0.677 38](javascript:showCorrelationPlot2(db='MA_M2M_0706_R',ProbeSetID='1433112_at',CellID='',db2='MA_M2M_0706_R',ProbeSetID2='1442239_at',CellID2='',rank='1')) | [0.431 38](javascript:showCorrelationPlot2(db='MA_M2M_0706_R',ProbeSetID='1433112_at',CellID='',db2='MA_M2M_0706_R',ProbeSetID2='1459865_x_at',CellID2='',rank='1')) | [-0.327 38](javascript:showCorrelationPlot2(db='MA_M2M_0706_R',ProbeSetID='1433112_at',CellID='',db2='MA_M2M_0706_R',ProbeSetID2='1438647_x_at',CellID2='',rank='1')) | [0.420 38](javascript:showCorrelationPlot2(db='MA_M2M_0706_R',ProbeSetID='1433112_at',CellID='',db2='MA_M2M_0706_R',ProbeSetID2='1432333_a_at',CellID2='',rank='1')) | [-0.430 38](javascript:showCorrelationPlot2(db='MA_M2M_0706_R',ProbeSetID='1433112_at',CellID='',db2='MA_M2M_0706_R',ProbeSetID2='1444028_s_at',CellID2='',rank='1')) | [0.575 38](javascript:showCorrelationPlot2(db='MA_M2M_0706_R',ProbeSetID='1433112_at',CellID='',db2='MA_M2M_0706_R',ProbeSetID2='1460420_a_at',CellID2='',rank='1')) | [0.645 38](javascript:showCorrelationPlot2(db='MA_M2M_0706_R',ProbeSetID='1433112_at',CellID='',db2='MA_M2M_0706_R',ProbeSetID2='1451530_at',CellID2='',rank='1')) | [-0.478 38](javascript:showCorrelationPlot2(db='MA_M2M_0706_R',ProbeSetID='1433112_at',CellID='',db2='MA_M2M_0706_R',ProbeSetID2='1423785_at',CellID2='',rank='1')) | [0.284 38](javascript:showCorrelationPlot2(db='MA_M2M_0706_R',ProbeSetID='1433112_at',CellID='',db2='MA_M2M_0706_R',ProbeSetID2='1431972_a_at',CellID2='',rank='1')) | [-0.530 38](javascript:showCorrelationPlot2(db='MA_M2M_0706_R',ProbeSetID='1433112_at',CellID='',db2='MA_M2M_0706_R',ProbeSetID2='1448347_a_at',CellID2='',rank='1')) | [0.545 38](javascript:showCorrelationPlot2(db='MA_M2M_0706_R',ProbeSetID='1433112_at',CellID='',db2='MA_M2M_0706_R',ProbeSetID2='1418492_at',CellID2='',rank='1')) | [-0.342 38](javascript:showCorrelationPlot2(db='MA_M2M_0706_R',ProbeSetID='1433112_at',CellID='',db2='MA_M2M_0706_R',ProbeSetID2='1427185_at',CellID2='',rank='1')) | [-0.453 38](javascript:showCorrelationPlot2(db='MA_M2M_0706_R',ProbeSetID='1433112_at',CellID='',db2='MA_M2M_0706_R',ProbeSetID2='1430500_s_at',CellID2='',rank='1')) | [0.476 38](javascript:showCorrelationPlot2(db='MA_M2M_0706_R',ProbeSetID='1433112_at',CellID='',db2='MA_M2M_0706_R',ProbeSetID2='1447549_x_at',CellID2='',rank='1')) | [-0.371 38](javascript:showCorrelationPlot2(db='MA_M2M_0706_R',ProbeSetID='1433112_at',CellID='',db2='MA_M2M_0706_R',ProbeSetID2='1448943_at',CellID2='',rank='1')) | [0.661 38](javascript:showCorrelationPlot2(db='MA_M2M_0706_R',ProbeSetID='1433112_at',CellID='',db2='MA_M2M_0706_R',ProbeSetID2='1433162_at',CellID2='',rank='1')) | [0.462 38](javascript:showCorrelationPlot2(db='MA_M2M_0706_R',ProbeSetID='1433112_at',CellID='',db2='MA_M2M_0706_R',ProbeSetID2='1443494_at',CellID2='',rank='1')) | [-0.316 38](javascript:showCorrelationPlot2(db='MA_M2M_0706_R',ProbeSetID='1433112_at',CellID='',db2='MA_M2M_0706_R',ProbeSetID2='1417426_at',CellID2='',rank='1')) | [0.548 38](javascript:showCorrelationPlot2(db='MA_M2M_0706_R',ProbeSetID='1433112_at',CellID='',db2='MA_M2M_0706_R',ProbeSetID2='1416588_at',CellID2='',rank='1')) | [-0.414 38](javascript:showCorrelationPlot2(db='MA_M2M_0706_R',ProbeSetID='1433112_at',CellID='',db2='MA_M2M_0706_R',ProbeSetID2='1450994_at',CellID2='',rank='1')) | [0.703 38](javascript:showCorrelationPlot2(db='MA_M2M_0706_R',ProbeSetID='1433112_at',CellID='',db2='MA_M2M_0706_R',ProbeSetID2='1420304_x_at',CellID2='',rank='1')) | [-0.509 38](javascript:showCorrelationPlot2(db='MA_M2M_0706_R',ProbeSetID='1433112_at',CellID='',db2='MA_M2M_0706_R',ProbeSetID2='1416500_at',CellID2='',rank='1')) | [0.684 38](javascript:showCorrelationPlot2(db='MA_M2M_0706_R',ProbeSetID='1433112_at',CellID='',db2='MA_M2M_0706_R',ProbeSetID2='1424199_at',CellID2='',rank='1')) | [-0.321 38](javascript:showCorrelationPlot2(db='MA_M2M_0706_R',ProbeSetID='1433112_at',CellID='',db2='MA_M2M_0706_R',ProbeSetID2='1437995_x_at',CellID2='',rank='1')) | [-0.424 38](javascript:showCorrelationPlot2(db='MA_M2M_0706_R',ProbeSetID='1433112_at',CellID='',db2='MA_M2M_0706_R',ProbeSetID2='1427914_a_at',CellID2='',rank='1')) | [0.590 38](javascript:showCorrelationPlot2(db='MA_M2M_0706_R',ProbeSetID='1433112_at',CellID='',db2='MA_M2M_0706_R',ProbeSetID2='1420570_x_at',CellID2='',rank='1')) | [0.456 38](javascript:showCorrelationPlot2(db='MA_M2M_0706_R',ProbeSetID='1433112_at',CellID='',db2='MA_M2M_0706_R',ProbeSetID2='1431015_at',CellID2='',rank='1')) | [-0.705 38](javascript:showCorrelationPlot2(db='MA_M2M_0706_R',ProbeSetID='1433112_at',CellID='',db2='MA_M2M_0706_R',ProbeSetID2='1423852_at',CellID2='',rank='1')) | [0.340 38](javascript:showCorrelationPlot2(db='MA_M2M_0706_R',ProbeSetID='1433112_at',CellID='',db2='MA_M2M_0706_R',ProbeSetID2='1431799_at',CellID2='',rank='1')) | [-0.449 38](javascript:showCorrelationPlot2(db='MA_M2M_0706_R',ProbeSetID='1433112_at',CellID='',db2='MA_M2M_0706_R',ProbeSetID2='1423898_a_at',CellID2='',rank='1')) | [0.602 38](javascript:showCorrelationPlot2(db='MA_M2M_0706_R',ProbeSetID='1433112_at',CellID='',db2='MA_M2M_0706_R',ProbeSetID2='1438579_at',CellID2='',rank='1')) | [-0.461 38](javascript:showCorrelationPlot2(db='MA_M2M_0706_R',ProbeSetID='1433112_at',CellID='',db2='MA_M2M_0706_R',ProbeSetID2='1448102_a_at',CellID2='',rank='1')) | [0.518 38](javascript:showCorrelationPlot2(db='MA_M2M_0706_R',ProbeSetID='1433112_at',CellID='',db2='MA_M2M_0706_R',ProbeSetID2='1420943_at',CellID2='',rank='1')) | [0.627 38](javascript:showCorrelationPlot2(db='MA_M2M_0706_R',ProbeSetID='1433112_at',CellID='',db2='MA_M2M_0706_R',ProbeSetID2='1447326_s_at',CellID2='',rank='1')) |
|  | [Trait 12: MA_M2M_0706_R::1459962_at](javascript:showDatabase2('MA_M2M_0706_R','1459962_at','');)  9330112F22Rik on Chr 1 @ 162.000288 Mb  ESTs | [0.393 38](javascript:showCorrelationPlot2(db='MA_M2M_0706_R',ProbeSetID='1459962_at',CellID='',db2='MA_M2M_0706_R',ProbeSetID2='1445101_at',CellID2='',rank='0')) | [-0.451 38](javascript:showCorrelationPlot2(db='MA_M2M_0706_R',ProbeSetID='1459962_at',CellID='',db2='MA_M2M_0706_R',ProbeSetID2='1451285_at',CellID2='',rank='0')) | [-0.586 38](javascript:showCorrelationPlot2(db='MA_M2M_0706_R',ProbeSetID='1459962_at',CellID='',db2='MA_M2M_0706_R',ProbeSetID2='1441347_at',CellID2='',rank='0')) | [0.421 38](javascript:showCorrelationPlot2(db='MA_M2M_0706_R',ProbeSetID='1459962_at',CellID='',db2='MA_M2M_0706_R',ProbeSetID2='1426900_at',CellID2='',rank='0')) | [0.442 38](javascript:showCorrelationPlot2(db='MA_M2M_0706_R',ProbeSetID='1459962_at',CellID='',db2='MA_M2M_0706_R',ProbeSetID2='1434888_a_at',CellID2='',rank='0')) | [0.585 38](javascript:showCorrelationPlot2(db='MA_M2M_0706_R',ProbeSetID='1459962_at',CellID='',db2='MA_M2M_0706_R',ProbeSetID2='1434773_a_at',CellID2='',rank='0')) | [-0.385 38](javascript:showCorrelationPlot2(db='MA_M2M_0706_R',ProbeSetID='1459962_at',CellID='',db2='MA_M2M_0706_R',ProbeSetID2='1452851_at',CellID2='',rank='0')) | [0.605 38](javascript:showCorrelationPlot2(db='MA_M2M_0706_R',ProbeSetID='1459962_at',CellID='',db2='MA_M2M_0706_R',ProbeSetID2='1441948_x_at',CellID2='',rank='0')) | [-0.431 38](javascript:showCorrelationPlot2(db='MA_M2M_0706_R',ProbeSetID='1459962_at',CellID='',db2='MA_M2M_0706_R',ProbeSetID2='1440160_x_at',CellID2='',rank='0')) | [0.327 38](javascript:showCorrelationPlot2(db='MA_M2M_0706_R',ProbeSetID='1459962_at',CellID='',db2='MA_M2M_0706_R',ProbeSetID2='1424692_at',CellID2='',rank='0')) | [-0.727 38](javascript:showCorrelationPlot2(db='MA_M2M_0706_R',ProbeSetID='1459962_at',CellID='',db2='MA_M2M_0706_R',ProbeSetID2='1433112_at',CellID2='',rank='0')) | [n 38](javascript:showDatabase2('MA_M2M_0706_R','1459962_at','')) | [-0.564 38](javascript:showCorrelationPlot2(db='MA_M2M_0706_R',ProbeSetID='1459962_at',CellID='',db2='MA_M2M_0706_R',ProbeSetID2='1455331_at',CellID2='',rank='1')) | [-0.651 38](javascript:showCorrelationPlot2(db='MA_M2M_0706_R',ProbeSetID='1459962_at',CellID='',db2='MA_M2M_0706_R',ProbeSetID2='1447399_at',CellID2='',rank='1')) | [0.250 38](javascript:showCorrelationPlot2(db='MA_M2M_0706_R',ProbeSetID='1459962_at',CellID='',db2='MA_M2M_0706_R',ProbeSetID2='1434968_a_at',CellID2='',rank='1')) | [-0.373 38](javascript:showCorrelationPlot2(db='MA_M2M_0706_R',ProbeSetID='1459962_at',CellID='',db2='MA_M2M_0706_R',ProbeSetID2='1447335_x_at',CellID2='',rank='1')) | [-0.521 38](javascript:showCorrelationPlot2(db='MA_M2M_0706_R',ProbeSetID='1459962_at',CellID='',db2='MA_M2M_0706_R',ProbeSetID2='1442239_at',CellID2='',rank='1')) | [-0.321 38](javascript:showCorrelationPlot2(db='MA_M2M_0706_R',ProbeSetID='1459962_at',CellID='',db2='MA_M2M_0706_R',ProbeSetID2='1459865_x_at',CellID2='',rank='1')) | [0.406 38](javascript:showCorrelationPlot2(db='MA_M2M_0706_R',ProbeSetID='1459962_at',CellID='',db2='MA_M2M_0706_R',ProbeSetID2='1438647_x_at',CellID2='',rank='1')) | [-0.441 38](javascript:showCorrelationPlot2(db='MA_M2M_0706_R',ProbeSetID='1459962_at',CellID='',db2='MA_M2M_0706_R',ProbeSetID2='1432333_a_at',CellID2='',rank='1')) | [0.576 38](javascript:showCorrelationPlot2(db='MA_M2M_0706_R',ProbeSetID='1459962_at',CellID='',db2='MA_M2M_0706_R',ProbeSetID2='1444028_s_at',CellID2='',rank='1')) | [-0.606 38](javascript:showCorrelationPlot2(db='MA_M2M_0706_R',ProbeSetID='1459962_at',CellID='',db2='MA_M2M_0706_R',ProbeSetID2='1460420_a_at',CellID2='',rank='1')) | [-0.550 38](javascript:showCorrelationPlot2(db='MA_M2M_0706_R',ProbeSetID='1459962_at',CellID='',db2='MA_M2M_0706_R',ProbeSetID2='1451530_at',CellID2='',rank='1')) | [0.511 38](javascript:showCorrelationPlot2(db='MA_M2M_0706_R',ProbeSetID='1459962_at',CellID='',db2='MA_M2M_0706_R',ProbeSetID2='1423785_at',CellID2='',rank='1')) | [-0.450 38](javascript:showCorrelationPlot2(db='MA_M2M_0706_R',ProbeSetID='1459962_at',CellID='',db2='MA_M2M_0706_R',ProbeSetID2='1431972_a_at',CellID2='',rank='1')) | [0.588 38](javascript:showCorrelationPlot2(db='MA_M2M_0706_R',ProbeSetID='1459962_at',CellID='',db2='MA_M2M_0706_R',ProbeSetID2='1448347_a_at',CellID2='',rank='1')) | [-0.512 38](javascript:showCorrelationPlot2(db='MA_M2M_0706_R',ProbeSetID='1459962_at',CellID='',db2='MA_M2M_0706_R',ProbeSetID2='1418492_at',CellID2='',rank='1')) | [0.430 38](javascript:showCorrelationPlot2(db='MA_M2M_0706_R',ProbeSetID='1459962_at',CellID='',db2='MA_M2M_0706_R',ProbeSetID2='1427185_at',CellID2='',rank='1')) | [0.282 38](javascript:showCorrelationPlot2(db='MA_M2M_0706_R',ProbeSetID='1459962_at',CellID='',db2='MA_M2M_0706_R',ProbeSetID2='1430500_s_at',CellID2='',rank='1')) | [-0.419 38](javascript:showCorrelationPlot2(db='MA_M2M_0706_R',ProbeSetID='1459962_at',CellID='',db2='MA_M2M_0706_R',ProbeSetID2='1447549_x_at',CellID2='',rank='1')) | [0.353 38](javascript:showCorrelationPlot2(db='MA_M2M_0706_R',ProbeSetID='1459962_at',CellID='',db2='MA_M2M_0706_R',ProbeSetID2='1448943_at',CellID2='',rank='1')) | [-0.517 38](javascript:showCorrelationPlot2(db='MA_M2M_0706_R',ProbeSetID='1459962_at',CellID='',db2='MA_M2M_0706_R',ProbeSetID2='1433162_at',CellID2='',rank='1')) | [-0.383 38](javascript:showCorrelationPlot2(db='MA_M2M_0706_R',ProbeSetID='1459962_at',CellID='',db2='MA_M2M_0706_R',ProbeSetID2='1443494_at',CellID2='',rank='1')) | [0.521 38](javascript:showCorrelationPlot2(db='MA_M2M_0706_R',ProbeSetID='1459962_at',CellID='',db2='MA_M2M_0706_R',ProbeSetID2='1417426_at',CellID2='',rank='1')) | [-0.483 38](javascript:showCorrelationPlot2(db='MA_M2M_0706_R',ProbeSetID='1459962_at',CellID='',db2='MA_M2M_0706_R',ProbeSetID2='1416588_at',CellID2='',rank='1')) | [0.496 38](javascript:showCorrelationPlot2(db='MA_M2M_0706_R',ProbeSetID='1459962_at',CellID='',db2='MA_M2M_0706_R',ProbeSetID2='1450994_at',CellID2='',rank='1')) | [-0.665 38](javascript:showCorrelationPlot2(db='MA_M2M_0706_R',ProbeSetID='1459962_at',CellID='',db2='MA_M2M_0706_R',ProbeSetID2='1420304_x_at',CellID2='',rank='1')) | [0.437 38](javascript:showCorrelationPlot2(db='MA_M2M_0706_R',ProbeSetID='1459962_at',CellID='',db2='MA_M2M_0706_R',ProbeSetID2='1416500_at',CellID2='',rank='1')) | [-0.617 38](javascript:showCorrelationPlot2(db='MA_M2M_0706_R',ProbeSetID='1459962_at',CellID='',db2='MA_M2M_0706_R',ProbeSetID2='1424199_at',CellID2='',rank='1')) | [0.387 38](javascript:showCorrelationPlot2(db='MA_M2M_0706_R',ProbeSetID='1459962_at',CellID='',db2='MA_M2M_0706_R',ProbeSetID2='1437995_x_at',CellID2='',rank='1')) | [0.423 38](javascript:showCorrelationPlot2(db='MA_M2M_0706_R',ProbeSetID='1459962_at',CellID='',db2='MA_M2M_0706_R',ProbeSetID2='1427914_a_at',CellID2='',rank='1')) | [-0.455 38](javascript:showCorrelationPlot2(db='MA_M2M_0706_R',ProbeSetID='1459962_at',CellID='',db2='MA_M2M_0706_R',ProbeSetID2='1420570_x_at',CellID2='',rank='1')) | [-0.465 38](javascript:showCorrelationPlot2(db='MA_M2M_0706_R',ProbeSetID='1459962_at',CellID='',db2='MA_M2M_0706_R',ProbeSetID2='1431015_at',CellID2='',rank='1')) | [0.590 38](javascript:showCorrelationPlot2(db='MA_M2M_0706_R',ProbeSetID='1459962_at',CellID='',db2='MA_M2M_0706_R',ProbeSetID2='1423852_at',CellID2='',rank='1')) | [-0.369 38](javascript:showCorrelationPlot2(db='MA_M2M_0706_R',ProbeSetID='1459962_at',CellID='',db2='MA_M2M_0706_R',ProbeSetID2='1431799_at',CellID2='',rank='1')) | [0.491 38](javascript:showCorrelationPlot2(db='MA_M2M_0706_R',ProbeSetID='1459962_at',CellID='',db2='MA_M2M_0706_R',ProbeSetID2='1423898_a_at',CellID2='',rank='1')) | [-0.465 38](javascript:showCorrelationPlot2(db='MA_M2M_0706_R',ProbeSetID='1459962_at',CellID='',db2='MA_M2M_0706_R',ProbeSetID2='1438579_at',CellID2='',rank='1')) | [0.463 38](javascript:showCorrelationPlot2(db='MA_M2M_0706_R',ProbeSetID='1459962_at',CellID='',db2='MA_M2M_0706_R',ProbeSetID2='1448102_a_at',CellID2='',rank='1')) | [-0.504 38](javascript:showCorrelationPlot2(db='MA_M2M_0706_R',ProbeSetID='1459962_at',CellID='',db2='MA_M2M_0706_R',ProbeSetID2='1420943_at',CellID2='',rank='1')) | [-0.436 38](javascript:showCorrelationPlot2(db='MA_M2M_0706_R',ProbeSetID='1459962_at',CellID='',db2='MA_M2M_0706_R',ProbeSetID2='1447326_s_at',CellID2='',rank='1')) |
|  | [Trait 13: MA_M2M_0706_R::1455331_at](javascript:showDatabase2('MA_M2M_0706_R','1455331_at','');)  9430067K14Rik on Chr 1 @ 64.835914 Mb  RIKEN cDNA 9430067K14 gene | [-0.618 38](javascript:showCorrelationPlot2(db='MA_M2M_0706_R',ProbeSetID='1455331_at',CellID='',db2='MA_M2M_0706_R',ProbeSetID2='1445101_at',CellID2='',rank='0')) | [0.513 38](javascript:showCorrelationPlot2(db='MA_M2M_0706_R',ProbeSetID='1455331_at',CellID='',db2='MA_M2M_0706_R',ProbeSetID2='1451285_at',CellID2='',rank='0')) | [0.681 38](javascript:showCorrelationPlot2(db='MA_M2M_0706_R',ProbeSetID='1455331_at',CellID='',db2='MA_M2M_0706_R',ProbeSetID2='1441347_at',CellID2='',rank='0')) | [-0.465 38](javascript:showCorrelationPlot2(db='MA_M2M_0706_R',ProbeSetID='1455331_at',CellID='',db2='MA_M2M_0706_R',ProbeSetID2='1426900_at',CellID2='',rank='0')) | [-0.323 38](javascript:showCorrelationPlot2(db='MA_M2M_0706_R',ProbeSetID='1455331_at',CellID='',db2='MA_M2M_0706_R',ProbeSetID2='1434888_a_at',CellID2='',rank='0')) | [-0.421 38](javascript:showCorrelationPlot2(db='MA_M2M_0706_R',ProbeSetID='1455331_at',CellID='',db2='MA_M2M_0706_R',ProbeSetID2='1434773_a_at',CellID2='',rank='0')) | [0.574 38](javascript:showCorrelationPlot2(db='MA_M2M_0706_R',ProbeSetID='1455331_at',CellID='',db2='MA_M2M_0706_R',ProbeSetID2='1452851_at',CellID2='',rank='0')) | [-0.651 38](javascript:showCorrelationPlot2(db='MA_M2M_0706_R',ProbeSetID='1455331_at',CellID='',db2='MA_M2M_0706_R',ProbeSetID2='1441948_x_at',CellID2='',rank='0')) | [0.668 38](javascript:showCorrelationPlot2(db='MA_M2M_0706_R',ProbeSetID='1455331_at',CellID='',db2='MA_M2M_0706_R',ProbeSetID2='1440160_x_at',CellID2='',rank='0')) | [-0.363 38](javascript:showCorrelationPlot2(db='MA_M2M_0706_R',ProbeSetID='1455331_at',CellID='',db2='MA_M2M_0706_R',ProbeSetID2='1424692_at',CellID2='',rank='0')) | [0.698 38](javascript:showCorrelationPlot2(db='MA_M2M_0706_R',ProbeSetID='1455331_at',CellID='',db2='MA_M2M_0706_R',ProbeSetID2='1433112_at',CellID2='',rank='0')) | [-0.542 38](javascript:showCorrelationPlot2(db='MA_M2M_0706_R',ProbeSetID='1455331_at',CellID='',db2='MA_M2M_0706_R',ProbeSetID2='1459962_at',CellID2='',rank='0')) | [n 38](javascript:showDatabase2('MA_M2M_0706_R','1455331_at','')) | [0.703 38](javascript:showCorrelationPlot2(db='MA_M2M_0706_R',ProbeSetID='1455331_at',CellID='',db2='MA_M2M_0706_R',ProbeSetID2='1447399_at',CellID2='',rank='1')) | [-0.363 38](javascript:showCorrelationPlot2(db='MA_M2M_0706_R',ProbeSetID='1455331_at',CellID='',db2='MA_M2M_0706_R',ProbeSetID2='1434968_a_at',CellID2='',rank='1')) | [0.552 38](javascript:showCorrelationPlot2(db='MA_M2M_0706_R',ProbeSetID='1455331_at',CellID='',db2='MA_M2M_0706_R',ProbeSetID2='1447335_x_at',CellID2='',rank='1')) | [0.608 38](javascript:showCorrelationPlot2(db='MA_M2M_0706_R',ProbeSetID='1455331_at',CellID='',db2='MA_M2M_0706_R',ProbeSetID2='1442239_at',CellID2='',rank='1')) | [0.331 38](javascript:showCorrelationPlot2(db='MA_M2M_0706_R',ProbeSetID='1455331_at',CellID='',db2='MA_M2M_0706_R',ProbeSetID2='1459865_x_at',CellID2='',rank='1')) | [-0.284 38](javascript:showCorrelationPlot2(db='MA_M2M_0706_R',ProbeSetID='1455331_at',CellID='',db2='MA_M2M_0706_R',ProbeSetID2='1438647_x_at',CellID2='',rank='1')) | [0.642 38](javascript:showCorrelationPlot2(db='MA_M2M_0706_R',ProbeSetID='1455331_at',CellID='',db2='MA_M2M_0706_R',ProbeSetID2='1432333_a_at',CellID2='',rank='1')) | [-0.381 38](javascript:showCorrelationPlot2(db='MA_M2M_0706_R',ProbeSetID='1455331_at',CellID='',db2='MA_M2M_0706_R',ProbeSetID2='1444028_s_at',CellID2='',rank='1')) | [0.618 38](javascript:showCorrelationPlot2(db='MA_M2M_0706_R',ProbeSetID='1455331_at',CellID='',db2='MA_M2M_0706_R',ProbeSetID2='1460420_a_at',CellID2='',rank='1')) | [0.467 38](javascript:showCorrelationPlot2(db='MA_M2M_0706_R',ProbeSetID='1455331_at',CellID='',db2='MA_M2M_0706_R',ProbeSetID2='1451530_at',CellID2='',rank='1')) | [-0.539 38](javascript:showCorrelationPlot2(db='MA_M2M_0706_R',ProbeSetID='1455331_at',CellID='',db2='MA_M2M_0706_R',ProbeSetID2='1423785_at',CellID2='',rank='1')) | [0.331 38](javascript:showCorrelationPlot2(db='MA_M2M_0706_R',ProbeSetID='1455331_at',CellID='',db2='MA_M2M_0706_R',ProbeSetID2='1431972_a_at',CellID2='',rank='1')) | [-0.561 38](javascript:showCorrelationPlot2(db='MA_M2M_0706_R',ProbeSetID='1455331_at',CellID='',db2='MA_M2M_0706_R',ProbeSetID2='1448347_a_at',CellID2='',rank='1')) | [0.555 38](javascript:showCorrelationPlot2(db='MA_M2M_0706_R',ProbeSetID='1455331_at',CellID='',db2='MA_M2M_0706_R',ProbeSetID2='1418492_at',CellID2='',rank='1')) | [-0.495 38](javascript:showCorrelationPlot2(db='MA_M2M_0706_R',ProbeSetID='1455331_at',CellID='',db2='MA_M2M_0706_R',ProbeSetID2='1427185_at',CellID2='',rank='1')) | [-0.319 38](javascript:showCorrelationPlot2(db='MA_M2M_0706_R',ProbeSetID='1455331_at',CellID='',db2='MA_M2M_0706_R',ProbeSetID2='1430500_s_at',CellID2='',rank='1')) | [0.682 38](javascript:showCorrelationPlot2(db='MA_M2M_0706_R',ProbeSetID='1455331_at',CellID='',db2='MA_M2M_0706_R',ProbeSetID2='1447549_x_at',CellID2='',rank='1')) | [-0.302 38](javascript:showCorrelationPlot2(db='MA_M2M_0706_R',ProbeSetID='1455331_at',CellID='',db2='MA_M2M_0706_R',ProbeSetID2='1448943_at',CellID2='',rank='1')) | [0.516 38](javascript:showCorrelationPlot2(db='MA_M2M_0706_R',ProbeSetID='1455331_at',CellID='',db2='MA_M2M_0706_R',ProbeSetID2='1433162_at',CellID2='',rank='1')) | [0.394 38](javascript:showCorrelationPlot2(db='MA_M2M_0706_R',ProbeSetID='1455331_at',CellID='',db2='MA_M2M_0706_R',ProbeSetID2='1443494_at',CellID2='',rank='1')) | [-0.403 38](javascript:showCorrelationPlot2(db='MA_M2M_0706_R',ProbeSetID='1455331_at',CellID='',db2='MA_M2M_0706_R',ProbeSetID2='1417426_at',CellID2='',rank='1')) | [0.591 38](javascript:showCorrelationPlot2(db='MA_M2M_0706_R',ProbeSetID='1455331_at',CellID='',db2='MA_M2M_0706_R',ProbeSetID2='1416588_at',CellID2='',rank='1')) | [-0.296 38](javascript:showCorrelationPlot2(db='MA_M2M_0706_R',ProbeSetID='1455331_at',CellID='',db2='MA_M2M_0706_R',ProbeSetID2='1450994_at',CellID2='',rank='1')) | [0.524 38](javascript:showCorrelationPlot2(db='MA_M2M_0706_R',ProbeSetID='1455331_at',CellID='',db2='MA_M2M_0706_R',ProbeSetID2='1420304_x_at',CellID2='',rank='1')) | [-0.602 38](javascript:showCorrelationPlot2(db='MA_M2M_0706_R',ProbeSetID='1455331_at',CellID='',db2='MA_M2M_0706_R',ProbeSetID2='1416500_at',CellID2='',rank='1')) | [0.719 38](javascript:showCorrelationPlot2(db='MA_M2M_0706_R',ProbeSetID='1455331_at',CellID='',db2='MA_M2M_0706_R',ProbeSetID2='1424199_at',CellID2='',rank='1')) | [-0.284 38](javascript:showCorrelationPlot2(db='MA_M2M_0706_R',ProbeSetID='1455331_at',CellID='',db2='MA_M2M_0706_R',ProbeSetID2='1437995_x_at',CellID2='',rank='1')) | [-0.310 38](javascript:showCorrelationPlot2(db='MA_M2M_0706_R',ProbeSetID='1455331_at',CellID='',db2='MA_M2M_0706_R',ProbeSetID2='1427914_a_at',CellID2='',rank='1')) | [0.522 38](javascript:showCorrelationPlot2(db='MA_M2M_0706_R',ProbeSetID='1455331_at',CellID='',db2='MA_M2M_0706_R',ProbeSetID2='1420570_x_at',CellID2='',rank='1')) | [0.450 38](javascript:showCorrelationPlot2(db='MA_M2M_0706_R',ProbeSetID='1455331_at',CellID='',db2='MA_M2M_0706_R',ProbeSetID2='1431015_at',CellID2='',rank='1')) | [-0.581 38](javascript:showCorrelationPlot2(db='MA_M2M_0706_R',ProbeSetID='1455331_at',CellID='',db2='MA_M2M_0706_R',ProbeSetID2='1423852_at',CellID2='',rank='1')) | [0.103 38](javascript:showCorrelationPlot2(db='MA_M2M_0706_R',ProbeSetID='1455331_at',CellID='',db2='MA_M2M_0706_R',ProbeSetID2='1431799_at',CellID2='',rank='1')) | [-0.354 38](javascript:showCorrelationPlot2(db='MA_M2M_0706_R',ProbeSetID='1455331_at',CellID='',db2='MA_M2M_0706_R',ProbeSetID2='1423898_a_at',CellID2='',rank='1')) | [0.667 38](javascript:showCorrelationPlot2(db='MA_M2M_0706_R',ProbeSetID='1455331_at',CellID='',db2='MA_M2M_0706_R',ProbeSetID2='1438579_at',CellID2='',rank='1')) | [-0.385 38](javascript:showCorrelationPlot2(db='MA_M2M_0706_R',ProbeSetID='1455331_at',CellID='',db2='MA_M2M_0706_R',ProbeSetID2='1448102_a_at',CellID2='',rank='1')) | [0.562 38](javascript:showCorrelationPlot2(db='MA_M2M_0706_R',ProbeSetID='1455331_at',CellID='',db2='MA_M2M_0706_R',ProbeSetID2='1420943_at',CellID2='',rank='1')) | [0.508 38](javascript:showCorrelationPlot2(db='MA_M2M_0706_R',ProbeSetID='1455331_at',CellID='',db2='MA_M2M_0706_R',ProbeSetID2='1447326_s_at',CellID2='',rank='1')) |
|  | [Trait 14: MA_M2M_0706_R::1447399_at](javascript:showDatabase2('MA_M2M_0706_R','1447399_at','');)  A330019N05Rik on Chr 10 @ 33.247113 Mb  RIKEN cDNA A330019N05  last intron | [-0.582 38](javascript:showCorrelationPlot2(db='MA_M2M_0706_R',ProbeSetID='1447399_at',CellID='',db2='MA_M2M_0706_R',ProbeSetID2='1445101_at',CellID2='',rank='0')) | [0.466 38](javascript:showCorrelationPlot2(db='MA_M2M_0706_R',ProbeSetID='1447399_at',CellID='',db2='MA_M2M_0706_R',ProbeSetID2='1451285_at',CellID2='',rank='0')) | [0.713 38](javascript:showCorrelationPlot2(db='MA_M2M_0706_R',ProbeSetID='1447399_at',CellID='',db2='MA_M2M_0706_R',ProbeSetID2='1441347_at',CellID2='',rank='0')) | [-0.413 38](javascript:showCorrelationPlot2(db='MA_M2M_0706_R',ProbeSetID='1447399_at',CellID='',db2='MA_M2M_0706_R',ProbeSetID2='1426900_at',CellID2='',rank='0')) | [-0.489 38](javascript:showCorrelationPlot2(db='MA_M2M_0706_R',ProbeSetID='1447399_at',CellID='',db2='MA_M2M_0706_R',ProbeSetID2='1434888_a_at',CellID2='',rank='0')) | [-0.505 38](javascript:showCorrelationPlot2(db='MA_M2M_0706_R',ProbeSetID='1447399_at',CellID='',db2='MA_M2M_0706_R',ProbeSetID2='1434773_a_at',CellID2='',rank='0')) | [0.552 38](javascript:showCorrelationPlot2(db='MA_M2M_0706_R',ProbeSetID='1447399_at',CellID='',db2='MA_M2M_0706_R',ProbeSetID2='1452851_at',CellID2='',rank='0')) | [-0.726 38](javascript:showCorrelationPlot2(db='MA_M2M_0706_R',ProbeSetID='1447399_at',CellID='',db2='MA_M2M_0706_R',ProbeSetID2='1441948_x_at',CellID2='',rank='0')) | [0.692 38](javascript:showCorrelationPlot2(db='MA_M2M_0706_R',ProbeSetID='1447399_at',CellID='',db2='MA_M2M_0706_R',ProbeSetID2='1440160_x_at',CellID2='',rank='0')) | [-0.486 38](javascript:showCorrelationPlot2(db='MA_M2M_0706_R',ProbeSetID='1447399_at',CellID='',db2='MA_M2M_0706_R',ProbeSetID2='1424692_at',CellID2='',rank='0')) | [0.742 38](javascript:showCorrelationPlot2(db='MA_M2M_0706_R',ProbeSetID='1447399_at',CellID='',db2='MA_M2M_0706_R',ProbeSetID2='1433112_at',CellID2='',rank='0')) | [-0.654 38](javascript:showCorrelationPlot2(db='MA_M2M_0706_R',ProbeSetID='1447399_at',CellID='',db2='MA_M2M_0706_R',ProbeSetID2='1459962_at',CellID2='',rank='0')) | [0.720 38](javascript:showCorrelationPlot2(db='MA_M2M_0706_R',ProbeSetID='1447399_at',CellID='',db2='MA_M2M_0706_R',ProbeSetID2='1455331_at',CellID2='',rank='0')) | [n 38](javascript:showDatabase2('MA_M2M_0706_R','1447399_at','')) | [-0.378 38](javascript:showCorrelationPlot2(db='MA_M2M_0706_R',ProbeSetID='1447399_at',CellID='',db2='MA_M2M_0706_R',ProbeSetID2='1434968_a_at',CellID2='',rank='1')) | [0.491 38](javascript:showCorrelationPlot2(db='MA_M2M_0706_R',ProbeSetID='1447399_at',CellID='',db2='MA_M2M_0706_R',ProbeSetID2='1447335_x_at',CellID2='',rank='1')) | [0.608 38](javascript:showCorrelationPlot2(db='MA_M2M_0706_R',ProbeSetID='1447399_at',CellID='',db2='MA_M2M_0706_R',ProbeSetID2='1442239_at',CellID2='',rank='1')) | [0.495 38](javascript:showCorrelationPlot2(db='MA_M2M_0706_R',ProbeSetID='1447399_at',CellID='',db2='MA_M2M_0706_R',ProbeSetID2='1459865_x_at',CellID2='',rank='1')) | [-0.530 38](javascript:showCorrelationPlot2(db='MA_M2M_0706_R',ProbeSetID='1447399_at',CellID='',db2='MA_M2M_0706_R',ProbeSetID2='1438647_x_at',CellID2='',rank='1')) | [0.527 38](javascript:showCorrelationPlot2(db='MA_M2M_0706_R',ProbeSetID='1447399_at',CellID='',db2='MA_M2M_0706_R',ProbeSetID2='1432333_a_at',CellID2='',rank='1')) | [-0.483 38](javascript:showCorrelationPlot2(db='MA_M2M_0706_R',ProbeSetID='1447399_at',CellID='',db2='MA_M2M_0706_R',ProbeSetID2='1444028_s_at',CellID2='',rank='1')) | [0.671 38](javascript:showCorrelationPlot2(db='MA_M2M_0706_R',ProbeSetID='1447399_at',CellID='',db2='MA_M2M_0706_R',ProbeSetID2='1460420_a_at',CellID2='',rank='1')) | [0.492 38](javascript:showCorrelationPlot2(db='MA_M2M_0706_R',ProbeSetID='1447399_at',CellID='',db2='MA_M2M_0706_R',ProbeSetID2='1451530_at',CellID2='',rank='1')) | [-0.446 38](javascript:showCorrelationPlot2(db='MA_M2M_0706_R',ProbeSetID='1447399_at',CellID='',db2='MA_M2M_0706_R',ProbeSetID2='1423785_at',CellID2='',rank='1')) | [0.252 38](javascript:showCorrelationPlot2(db='MA_M2M_0706_R',ProbeSetID='1447399_at',CellID='',db2='MA_M2M_0706_R',ProbeSetID2='1431972_a_at',CellID2='',rank='1')) | [-0.430 38](javascript:showCorrelationPlot2(db='MA_M2M_0706_R',ProbeSetID='1447399_at',CellID='',db2='MA_M2M_0706_R',ProbeSetID2='1448347_a_at',CellID2='',rank='1')) | [0.635 38](javascript:showCorrelationPlot2(db='MA_M2M_0706_R',ProbeSetID='1447399_at',CellID='',db2='MA_M2M_0706_R',ProbeSetID2='1418492_at',CellID2='',rank='1')) | [-0.443 38](javascript:showCorrelationPlot2(db='MA_M2M_0706_R',ProbeSetID='1447399_at',CellID='',db2='MA_M2M_0706_R',ProbeSetID2='1427185_at',CellID2='',rank='1')) | [-0.385 38](javascript:showCorrelationPlot2(db='MA_M2M_0706_R',ProbeSetID='1447399_at',CellID='',db2='MA_M2M_0706_R',ProbeSetID2='1430500_s_at',CellID2='',rank='1')) | [0.471 38](javascript:showCorrelationPlot2(db='MA_M2M_0706_R',ProbeSetID='1447399_at',CellID='',db2='MA_M2M_0706_R',ProbeSetID2='1447549_x_at',CellID2='',rank='1')) | [-0.355 38](javascript:showCorrelationPlot2(db='MA_M2M_0706_R',ProbeSetID='1447399_at',CellID='',db2='MA_M2M_0706_R',ProbeSetID2='1448943_at',CellID2='',rank='1')) | [0.465 38](javascript:showCorrelationPlot2(db='MA_M2M_0706_R',ProbeSetID='1447399_at',CellID='',db2='MA_M2M_0706_R',ProbeSetID2='1433162_at',CellID2='',rank='1')) | [0.415 38](javascript:showCorrelationPlot2(db='MA_M2M_0706_R',ProbeSetID='1447399_at',CellID='',db2='MA_M2M_0706_R',ProbeSetID2='1443494_at',CellID2='',rank='1')) | [-0.463 38](javascript:showCorrelationPlot2(db='MA_M2M_0706_R',ProbeSetID='1447399_at',CellID='',db2='MA_M2M_0706_R',ProbeSetID2='1417426_at',CellID2='',rank='1')) | [0.600 38](javascript:showCorrelationPlot2(db='MA_M2M_0706_R',ProbeSetID='1447399_at',CellID='',db2='MA_M2M_0706_R',ProbeSetID2='1416588_at',CellID2='',rank='1')) | [-0.460 38](javascript:showCorrelationPlot2(db='MA_M2M_0706_R',ProbeSetID='1447399_at',CellID='',db2='MA_M2M_0706_R',ProbeSetID2='1450994_at',CellID2='',rank='1')) | [0.682 38](javascript:showCorrelationPlot2(db='MA_M2M_0706_R',ProbeSetID='1447399_at',CellID='',db2='MA_M2M_0706_R',ProbeSetID2='1420304_x_at',CellID2='',rank='1')) | [-0.404 38](javascript:showCorrelationPlot2(db='MA_M2M_0706_R',ProbeSetID='1447399_at',CellID='',db2='MA_M2M_0706_R',ProbeSetID2='1416500_at',CellID2='',rank='1')) | [0.714 38](javascript:showCorrelationPlot2(db='MA_M2M_0706_R',ProbeSetID='1447399_at',CellID='',db2='MA_M2M_0706_R',ProbeSetID2='1424199_at',CellID2='',rank='1')) | [-0.275 38](javascript:showCorrelationPlot2(db='MA_M2M_0706_R',ProbeSetID='1447399_at',CellID='',db2='MA_M2M_0706_R',ProbeSetID2='1437995_x_at',CellID2='',rank='1')) | [-0.495 38](javascript:showCorrelationPlot2(db='MA_M2M_0706_R',ProbeSetID='1447399_at',CellID='',db2='MA_M2M_0706_R',ProbeSetID2='1427914_a_at',CellID2='',rank='1')) | [0.561 38](javascript:showCorrelationPlot2(db='MA_M2M_0706_R',ProbeSetID='1447399_at',CellID='',db2='MA_M2M_0706_R',ProbeSetID2='1420570_x_at',CellID2='',rank='1')) | [0.406 38](javascript:showCorrelationPlot2(db='MA_M2M_0706_R',ProbeSetID='1447399_at',CellID='',db2='MA_M2M_0706_R',ProbeSetID2='1431015_at',CellID2='',rank='1')) | [-0.654 38](javascript:showCorrelationPlot2(db='MA_M2M_0706_R',ProbeSetID='1447399_at',CellID='',db2='MA_M2M_0706_R',ProbeSetID2='1423852_at',CellID2='',rank='1')) | [0.293 38](javascript:showCorrelationPlot2(db='MA_M2M_0706_R',ProbeSetID='1447399_at',CellID='',db2='MA_M2M_0706_R',ProbeSetID2='1431799_at',CellID2='',rank='1')) | [-0.354 38](javascript:showCorrelationPlot2(db='MA_M2M_0706_R',ProbeSetID='1447399_at',CellID='',db2='MA_M2M_0706_R',ProbeSetID2='1423898_a_at',CellID2='',rank='1')) | [0.733 38](javascript:showCorrelationPlot2(db='MA_M2M_0706_R',ProbeSetID='1447399_at',CellID='',db2='MA_M2M_0706_R',ProbeSetID2='1438579_at',CellID2='',rank='1')) | [-0.560 38](javascript:showCorrelationPlot2(db='MA_M2M_0706_R',ProbeSetID='1447399_at',CellID='',db2='MA_M2M_0706_R',ProbeSetID2='1448102_a_at',CellID2='',rank='1')) | [0.540 38](javascript:showCorrelationPlot2(db='MA_M2M_0706_R',ProbeSetID='1447399_at',CellID='',db2='MA_M2M_0706_R',ProbeSetID2='1420943_at',CellID2='',rank='1')) | [0.515 38](javascript:showCorrelationPlot2(db='MA_M2M_0706_R',ProbeSetID='1447399_at',CellID='',db2='MA_M2M_0706_R',ProbeSetID2='1447326_s_at',CellID2='',rank='1')) |
|  | [Trait 15: MA_M2M_0706_R::1434968_a_at](javascript:showDatabase2('MA_M2M_0706_R','1434968_a_at','');)  Actr3 on Chr 1 @ 127.289563 Mb  ARP3 actin-related protein 3 homolog (yeast)  distal 3' UTR | [0.535 38](javascript:showCorrelationPlot2(db='MA_M2M_0706_R',ProbeSetID='1434968_a_at',CellID='',db2='MA_M2M_0706_R',ProbeSetID2='1445101_at',CellID2='',rank='0')) | [-0.217 38](javascript:showCorrelationPlot2(db='MA_M2M_0706_R',ProbeSetID='1434968_a_at',CellID='',db2='MA_M2M_0706_R',ProbeSetID2='1451285_at',CellID2='',rank='0')) | [-0.326 38](javascript:showCorrelationPlot2(db='MA_M2M_0706_R',ProbeSetID='1434968_a_at',CellID='',db2='MA_M2M_0706_R',ProbeSetID2='1441347_at',CellID2='',rank='0')) | [0.576 38](javascript:showCorrelationPlot2(db='MA_M2M_0706_R',ProbeSetID='1434968_a_at',CellID='',db2='MA_M2M_0706_R',ProbeSetID2='1426900_at',CellID2='',rank='0')) | [0.628 38](javascript:showCorrelationPlot2(db='MA_M2M_0706_R',ProbeSetID='1434968_a_at',CellID='',db2='MA_M2M_0706_R',ProbeSetID2='1434888_a_at',CellID2='',rank='0')) | [0.379 38](javascript:showCorrelationPlot2(db='MA_M2M_0706_R',ProbeSetID='1434968_a_at',CellID='',db2='MA_M2M_0706_R',ProbeSetID2='1434773_a_at',CellID2='',rank='0')) | [-0.423 38](javascript:showCorrelationPlot2(db='MA_M2M_0706_R',ProbeSetID='1434968_a_at',CellID='',db2='MA_M2M_0706_R',ProbeSetID2='1452851_at',CellID2='',rank='0')) | [0.402 38](javascript:showCorrelationPlot2(db='MA_M2M_0706_R',ProbeSetID='1434968_a_at',CellID='',db2='MA_M2M_0706_R',ProbeSetID2='1441948_x_at',CellID2='',rank='0')) | [-0.641 38](javascript:showCorrelationPlot2(db='MA_M2M_0706_R',ProbeSetID='1434968_a_at',CellID='',db2='MA_M2M_0706_R',ProbeSetID2='1440160_x_at',CellID2='',rank='0')) | [0.638 38](javascript:showCorrelationPlot2(db='MA_M2M_0706_R',ProbeSetID='1434968_a_at',CellID='',db2='MA_M2M_0706_R',ProbeSetID2='1424692_at',CellID2='',rank='0')) | [-0.436 38](javascript:showCorrelationPlot2(db='MA_M2M_0706_R',ProbeSetID='1434968_a_at',CellID='',db2='MA_M2M_0706_R',ProbeSetID2='1433112_at',CellID2='',rank='0')) | [0.267 38](javascript:showCorrelationPlot2(db='MA_M2M_0706_R',ProbeSetID='1434968_a_at',CellID='',db2='MA_M2M_0706_R',ProbeSetID2='1459962_at',CellID2='',rank='0')) | [-0.475 38](javascript:showCorrelationPlot2(db='MA_M2M_0706_R',ProbeSetID='1434968_a_at',CellID='',db2='MA_M2M_0706_R',ProbeSetID2='1455331_at',CellID2='',rank='0')) | [-0.385 38](javascript:showCorrelationPlot2(db='MA_M2M_0706_R',ProbeSetID='1434968_a_at',CellID='',db2='MA_M2M_0706_R',ProbeSetID2='1447399_at',CellID2='',rank='0')) | [n 38](javascript:showDatabase2('MA_M2M_0706_R','1434968_a_at','')) | [-0.408 38](javascript:showCorrelationPlot2(db='MA_M2M_0706_R',ProbeSetID='1434968_a_at',CellID='',db2='MA_M2M_0706_R',ProbeSetID2='1447335_x_at',CellID2='',rank='1')) | [-0.510 38](javascript:showCorrelationPlot2(db='MA_M2M_0706_R',ProbeSetID='1434968_a_at',CellID='',db2='MA_M2M_0706_R',ProbeSetID2='1442239_at',CellID2='',rank='1')) | [-0.494 38](javascript:showCorrelationPlot2(db='MA_M2M_0706_R',ProbeSetID='1434968_a_at',CellID='',db2='MA_M2M_0706_R',ProbeSetID2='1459865_x_at',CellID2='',rank='1')) | [0.374 38](javascript:showCorrelationPlot2(db='MA_M2M_0706_R',ProbeSetID='1434968_a_at',CellID='',db2='MA_M2M_0706_R',ProbeSetID2='1438647_x_at',CellID2='',rank='1')) | [-0.298 38](javascript:showCorrelationPlot2(db='MA_M2M_0706_R',ProbeSetID='1434968_a_at',CellID='',db2='MA_M2M_0706_R',ProbeSetID2='1432333_a_at',CellID2='',rank='1')) | [0.424 38](javascript:showCorrelationPlot2(db='MA_M2M_0706_R',ProbeSetID='1434968_a_at',CellID='',db2='MA_M2M_0706_R',ProbeSetID2='1444028_s_at',CellID2='',rank='1')) | [-0.592 38](javascript:showCorrelationPlot2(db='MA_M2M_0706_R',ProbeSetID='1434968_a_at',CellID='',db2='MA_M2M_0706_R',ProbeSetID2='1460420_a_at',CellID2='',rank='1')) | [-0.213 38](javascript:showCorrelationPlot2(db='MA_M2M_0706_R',ProbeSetID='1434968_a_at',CellID='',db2='MA_M2M_0706_R',ProbeSetID2='1451530_at',CellID2='',rank='1')) | [0.451 38](javascript:showCorrelationPlot2(db='MA_M2M_0706_R',ProbeSetID='1434968_a_at',CellID='',db2='MA_M2M_0706_R',ProbeSetID2='1423785_at',CellID2='',rank='1')) | [-0.532 38](javascript:showCorrelationPlot2(db='MA_M2M_0706_R',ProbeSetID='1434968_a_at',CellID='',db2='MA_M2M_0706_R',ProbeSetID2='1431972_a_at',CellID2='',rank='1')) | [0.405 38](javascript:showCorrelationPlot2(db='MA_M2M_0706_R',ProbeSetID='1434968_a_at',CellID='',db2='MA_M2M_0706_R',ProbeSetID2='1448347_a_at',CellID2='',rank='1')) | [-0.403 38](javascript:showCorrelationPlot2(db='MA_M2M_0706_R',ProbeSetID='1434968_a_at',CellID='',db2='MA_M2M_0706_R',ProbeSetID2='1418492_at',CellID2='',rank='1')) | [0.369 38](javascript:showCorrelationPlot2(db='MA_M2M_0706_R',ProbeSetID='1434968_a_at',CellID='',db2='MA_M2M_0706_R',ProbeSetID2='1427185_at',CellID2='',rank='1')) | [0.496 38](javascript:showCorrelationPlot2(db='MA_M2M_0706_R',ProbeSetID='1434968_a_at',CellID='',db2='MA_M2M_0706_R',ProbeSetID2='1430500_s_at',CellID2='',rank='1')) | [-0.285 38](javascript:showCorrelationPlot2(db='MA_M2M_0706_R',ProbeSetID='1434968_a_at',CellID='',db2='MA_M2M_0706_R',ProbeSetID2='1447549_x_at',CellID2='',rank='1')) | [0.482 38](javascript:showCorrelationPlot2(db='MA_M2M_0706_R',ProbeSetID='1434968_a_at',CellID='',db2='MA_M2M_0706_R',ProbeSetID2='1448943_at',CellID2='',rank='1')) | [-0.390 38](javascript:showCorrelationPlot2(db='MA_M2M_0706_R',ProbeSetID='1434968_a_at',CellID='',db2='MA_M2M_0706_R',ProbeSetID2='1433162_at',CellID2='',rank='1')) | [-0.392 38](javascript:showCorrelationPlot2(db='MA_M2M_0706_R',ProbeSetID='1434968_a_at',CellID='',db2='MA_M2M_0706_R',ProbeSetID2='1443494_at',CellID2='',rank='1')) | [0.224 38](javascript:showCorrelationPlot2(db='MA_M2M_0706_R',ProbeSetID='1434968_a_at',CellID='',db2='MA_M2M_0706_R',ProbeSetID2='1417426_at',CellID2='',rank='1')) | [-0.513 38](javascript:showCorrelationPlot2(db='MA_M2M_0706_R',ProbeSetID='1434968_a_at',CellID='',db2='MA_M2M_0706_R',ProbeSetID2='1416588_at',CellID2='',rank='1')) | [0.271 38](javascript:showCorrelationPlot2(db='MA_M2M_0706_R',ProbeSetID='1434968_a_at',CellID='',db2='MA_M2M_0706_R',ProbeSetID2='1450994_at',CellID2='',rank='1')) | [-0.493 38](javascript:showCorrelationPlot2(db='MA_M2M_0706_R',ProbeSetID='1434968_a_at',CellID='',db2='MA_M2M_0706_R',ProbeSetID2='1420304_x_at',CellID2='',rank='1')) | [0.372 38](javascript:showCorrelationPlot2(db='MA_M2M_0706_R',ProbeSetID='1434968_a_at',CellID='',db2='MA_M2M_0706_R',ProbeSetID2='1416500_at',CellID2='',rank='1')) | [-0.397 38](javascript:showCorrelationPlot2(db='MA_M2M_0706_R',ProbeSetID='1434968_a_at',CellID='',db2='MA_M2M_0706_R',ProbeSetID2='1424199_at',CellID2='',rank='1')) | [0.517 38](javascript:showCorrelationPlot2(db='MA_M2M_0706_R',ProbeSetID='1434968_a_at',CellID='',db2='MA_M2M_0706_R',ProbeSetID2='1437995_x_at',CellID2='',rank='1')) | [0.294 38](javascript:showCorrelationPlot2(db='MA_M2M_0706_R',ProbeSetID='1434968_a_at',CellID='',db2='MA_M2M_0706_R',ProbeSetID2='1427914_a_at',CellID2='',rank='1')) | [-0.517 38](javascript:showCorrelationPlot2(db='MA_M2M_0706_R',ProbeSetID='1434968_a_at',CellID='',db2='MA_M2M_0706_R',ProbeSetID2='1420570_x_at',CellID2='',rank='1')) | [-0.544 38](javascript:showCorrelationPlot2(db='MA_M2M_0706_R',ProbeSetID='1434968_a_at',CellID='',db2='MA_M2M_0706_R',ProbeSetID2='1431015_at',CellID2='',rank='1')) | [0.374 38](javascript:showCorrelationPlot2(db='MA_M2M_0706_R',ProbeSetID='1434968_a_at',CellID='',db2='MA_M2M_0706_R',ProbeSetID2='1423852_at',CellID2='',rank='1')) | [-0.375 38](javascript:showCorrelationPlot2(db='MA_M2M_0706_R',ProbeSetID='1434968_a_at',CellID='',db2='MA_M2M_0706_R',ProbeSetID2='1431799_at',CellID2='',rank='1')) | [0.382 38](javascript:showCorrelationPlot2(db='MA_M2M_0706_R',ProbeSetID='1434968_a_at',CellID='',db2='MA_M2M_0706_R',ProbeSetID2='1423898_a_at',CellID2='',rank='1')) | [-0.428 38](javascript:showCorrelationPlot2(db='MA_M2M_0706_R',ProbeSetID='1434968_a_at',CellID='',db2='MA_M2M_0706_R',ProbeSetID2='1438579_at',CellID2='',rank='1')) | [0.475 38](javascript:showCorrelationPlot2(db='MA_M2M_0706_R',ProbeSetID='1434968_a_at',CellID='',db2='MA_M2M_0706_R',ProbeSetID2='1448102_a_at',CellID2='',rank='1')) | [-0.316 38](javascript:showCorrelationPlot2(db='MA_M2M_0706_R',ProbeSetID='1434968_a_at',CellID='',db2='MA_M2M_0706_R',ProbeSetID2='1420943_at',CellID2='',rank='1')) | [-0.456 38](javascript:showCorrelationPlot2(db='MA_M2M_0706_R',ProbeSetID='1434968_a_at',CellID='',db2='MA_M2M_0706_R',ProbeSetID2='1447326_s_at',CellID2='',rank='1')) |
|  | [Trait 16: MA_M2M_0706_R::1447335_x_at](javascript:showDatabase2('MA_M2M_0706_R','1447335_x_at','');)  Bcl11a on Chr 11 @ 23.977912 Mb  B-cell CLL/lymphoma 11A (zinc finger protein) | [-0.638 38](javascript:showCorrelationPlot2(db='MA_M2M_0706_R',ProbeSetID='1447335_x_at',CellID='',db2='MA_M2M_0706_R',ProbeSetID2='1445101_at',CellID2='',rank='0')) | [0.374 38](javascript:showCorrelationPlot2(db='MA_M2M_0706_R',ProbeSetID='1447335_x_at',CellID='',db2='MA_M2M_0706_R',ProbeSetID2='1451285_at',CellID2='',rank='0')) | [0.680 38](javascript:showCorrelationPlot2(db='MA_M2M_0706_R',ProbeSetID='1447335_x_at',CellID='',db2='MA_M2M_0706_R',ProbeSetID2='1441347_at',CellID2='',rank='0')) | [-0.571 38](javascript:showCorrelationPlot2(db='MA_M2M_0706_R',ProbeSetID='1447335_x_at',CellID='',db2='MA_M2M_0706_R',ProbeSetID2='1426900_at',CellID2='',rank='0')) | [-0.451 38](javascript:showCorrelationPlot2(db='MA_M2M_0706_R',ProbeSetID='1447335_x_at',CellID='',db2='MA_M2M_0706_R',ProbeSetID2='1434888_a_at',CellID2='',rank='0')) | [-0.533 38](javascript:showCorrelationPlot2(db='MA_M2M_0706_R',ProbeSetID='1447335_x_at',CellID='',db2='MA_M2M_0706_R',ProbeSetID2='1434773_a_at',CellID2='',rank='0')) | [0.770 38](javascript:showCorrelationPlot2(db='MA_M2M_0706_R',ProbeSetID='1447335_x_at',CellID='',db2='MA_M2M_0706_R',ProbeSetID2='1452851_at',CellID2='',rank='0')) | [-0.554 38](javascript:showCorrelationPlot2(db='MA_M2M_0706_R',ProbeSetID='1447335_x_at',CellID='',db2='MA_M2M_0706_R',ProbeSetID2='1441948_x_at',CellID2='',rank='0')) | [0.702 38](javascript:showCorrelationPlot2(db='MA_M2M_0706_R',ProbeSetID='1447335_x_at',CellID='',db2='MA_M2M_0706_R',ProbeSetID2='1440160_x_at',CellID2='',rank='0')) | [-0.502 38](javascript:showCorrelationPlot2(db='MA_M2M_0706_R',ProbeSetID='1447335_x_at',CellID='',db2='MA_M2M_0706_R',ProbeSetID2='1424692_at',CellID2='',rank='0')) | [0.659 38](javascript:showCorrelationPlot2(db='MA_M2M_0706_R',ProbeSetID='1447335_x_at',CellID='',db2='MA_M2M_0706_R',ProbeSetID2='1433112_at',CellID2='',rank='0')) | [-0.409 38](javascript:showCorrelationPlot2(db='MA_M2M_0706_R',ProbeSetID='1447335_x_at',CellID='',db2='MA_M2M_0706_R',ProbeSetID2='1459962_at',CellID2='',rank='0')) | [0.661 38](javascript:showCorrelationPlot2(db='MA_M2M_0706_R',ProbeSetID='1447335_x_at',CellID='',db2='MA_M2M_0706_R',ProbeSetID2='1455331_at',CellID2='',rank='0')) | [0.582 38](javascript:showCorrelationPlot2(db='MA_M2M_0706_R',ProbeSetID='1447335_x_at',CellID='',db2='MA_M2M_0706_R',ProbeSetID2='1447399_at',CellID2='',rank='0')) | [-0.436 38](javascript:showCorrelationPlot2(db='MA_M2M_0706_R',ProbeSetID='1447335_x_at',CellID='',db2='MA_M2M_0706_R',ProbeSetID2='1434968_a_at',CellID2='',rank='0')) | [n 38](javascript:showDatabase2('MA_M2M_0706_R','1447335_x_at','')) | [0.694 38](javascript:showCorrelationPlot2(db='MA_M2M_0706_R',ProbeSetID='1447335_x_at',CellID='',db2='MA_M2M_0706_R',ProbeSetID2='1442239_at',CellID2='',rank='1')) | [0.585 38](javascript:showCorrelationPlot2(db='MA_M2M_0706_R',ProbeSetID='1447335_x_at',CellID='',db2='MA_M2M_0706_R',ProbeSetID2='1459865_x_at',CellID2='',rank='1')) | [-0.288 38](javascript:showCorrelationPlot2(db='MA_M2M_0706_R',ProbeSetID='1447335_x_at',CellID='',db2='MA_M2M_0706_R',ProbeSetID2='1438647_x_at',CellID2='',rank='1')) | [0.497 38](javascript:showCorrelationPlot2(db='MA_M2M_0706_R',ProbeSetID='1447335_x_at',CellID='',db2='MA_M2M_0706_R',ProbeSetID2='1432333_a_at',CellID2='',rank='1')) | [-0.451 38](javascript:showCorrelationPlot2(db='MA_M2M_0706_R',ProbeSetID='1447335_x_at',CellID='',db2='MA_M2M_0706_R',ProbeSetID2='1444028_s_at',CellID2='',rank='1')) | [0.588 38](javascript:showCorrelationPlot2(db='MA_M2M_0706_R',ProbeSetID='1447335_x_at',CellID='',db2='MA_M2M_0706_R',ProbeSetID2='1460420_a_at',CellID2='',rank='1')) | [0.616 38](javascript:showCorrelationPlot2(db='MA_M2M_0706_R',ProbeSetID='1447335_x_at',CellID='',db2='MA_M2M_0706_R',ProbeSetID2='1451530_at',CellID2='',rank='1')) | [-0.595 38](javascript:showCorrelationPlot2(db='MA_M2M_0706_R',ProbeSetID='1447335_x_at',CellID='',db2='MA_M2M_0706_R',ProbeSetID2='1423785_at',CellID2='',rank='1')) | [0.313 38](javascript:showCorrelationPlot2(db='MA_M2M_0706_R',ProbeSetID='1447335_x_at',CellID='',db2='MA_M2M_0706_R',ProbeSetID2='1431972_a_at',CellID2='',rank='1')) | [-0.553 38](javascript:showCorrelationPlot2(db='MA_M2M_0706_R',ProbeSetID='1447335_x_at',CellID='',db2='MA_M2M_0706_R',ProbeSetID2='1448347_a_at',CellID2='',rank='1')) | [0.579 38](javascript:showCorrelationPlot2(db='MA_M2M_0706_R',ProbeSetID='1447335_x_at',CellID='',db2='MA_M2M_0706_R',ProbeSetID2='1418492_at',CellID2='',rank='1')) | [-0.576 38](javascript:showCorrelationPlot2(db='MA_M2M_0706_R',ProbeSetID='1447335_x_at',CellID='',db2='MA_M2M_0706_R',ProbeSetID2='1427185_at',CellID2='',rank='1')) | [-0.564 38](javascript:showCorrelationPlot2(db='MA_M2M_0706_R',ProbeSetID='1447335_x_at',CellID='',db2='MA_M2M_0706_R',ProbeSetID2='1430500_s_at',CellID2='',rank='1')) | [0.717 38](javascript:showCorrelationPlot2(db='MA_M2M_0706_R',ProbeSetID='1447335_x_at',CellID='',db2='MA_M2M_0706_R',ProbeSetID2='1447549_x_at',CellID2='',rank='1')) | [-0.575 38](javascript:showCorrelationPlot2(db='MA_M2M_0706_R',ProbeSetID='1447335_x_at',CellID='',db2='MA_M2M_0706_R',ProbeSetID2='1448943_at',CellID2='',rank='1')) | [0.675 38](javascript:showCorrelationPlot2(db='MA_M2M_0706_R',ProbeSetID='1447335_x_at',CellID='',db2='MA_M2M_0706_R',ProbeSetID2='1433162_at',CellID2='',rank='1')) | [0.571 38](javascript:showCorrelationPlot2(db='MA_M2M_0706_R',ProbeSetID='1447335_x_at',CellID='',db2='MA_M2M_0706_R',ProbeSetID2='1443494_at',CellID2='',rank='1')) | [-0.425 38](javascript:showCorrelationPlot2(db='MA_M2M_0706_R',ProbeSetID='1447335_x_at',CellID='',db2='MA_M2M_0706_R',ProbeSetID2='1417426_at',CellID2='',rank='1')) | [0.599 38](javascript:showCorrelationPlot2(db='MA_M2M_0706_R',ProbeSetID='1447335_x_at',CellID='',db2='MA_M2M_0706_R',ProbeSetID2='1416588_at',CellID2='',rank='1')) | [-0.426 38](javascript:showCorrelationPlot2(db='MA_M2M_0706_R',ProbeSetID='1447335_x_at',CellID='',db2='MA_M2M_0706_R',ProbeSetID2='1450994_at',CellID2='',rank='1')) | [0.591 38](javascript:showCorrelationPlot2(db='MA_M2M_0706_R',ProbeSetID='1447335_x_at',CellID='',db2='MA_M2M_0706_R',ProbeSetID2='1420304_x_at',CellID2='',rank='1')) | [-0.714 38](javascript:showCorrelationPlot2(db='MA_M2M_0706_R',ProbeSetID='1447335_x_at',CellID='',db2='MA_M2M_0706_R',ProbeSetID2='1416500_at',CellID2='',rank='1')) | [0.561 38](javascript:showCorrelationPlot2(db='MA_M2M_0706_R',ProbeSetID='1447335_x_at',CellID='',db2='MA_M2M_0706_R',ProbeSetID2='1424199_at',CellID2='',rank='1')) | [-0.632 38](javascript:showCorrelationPlot2(db='MA_M2M_0706_R',ProbeSetID='1447335_x_at',CellID='',db2='MA_M2M_0706_R',ProbeSetID2='1437995_x_at',CellID2='',rank='1')) | [-0.477 38](javascript:showCorrelationPlot2(db='MA_M2M_0706_R',ProbeSetID='1447335_x_at',CellID='',db2='MA_M2M_0706_R',ProbeSetID2='1427914_a_at',CellID2='',rank='1')) | [0.669 38](javascript:showCorrelationPlot2(db='MA_M2M_0706_R',ProbeSetID='1447335_x_at',CellID='',db2='MA_M2M_0706_R',ProbeSetID2='1420570_x_at',CellID2='',rank='1')) | [0.637 38](javascript:showCorrelationPlot2(db='MA_M2M_0706_R',ProbeSetID='1447335_x_at',CellID='',db2='MA_M2M_0706_R',ProbeSetID2='1431015_at',CellID2='',rank='1')) | [-0.478 38](javascript:showCorrelationPlot2(db='MA_M2M_0706_R',ProbeSetID='1447335_x_at',CellID='',db2='MA_M2M_0706_R',ProbeSetID2='1423852_at',CellID2='',rank='1')) | [0.307 38](javascript:showCorrelationPlot2(db='MA_M2M_0706_R',ProbeSetID='1447335_x_at',CellID='',db2='MA_M2M_0706_R',ProbeSetID2='1431799_at',CellID2='',rank='1')) | [-0.478 38](javascript:showCorrelationPlot2(db='MA_M2M_0706_R',ProbeSetID='1447335_x_at',CellID='',db2='MA_M2M_0706_R',ProbeSetID2='1423898_a_at',CellID2='',rank='1')) | [0.632 38](javascript:showCorrelationPlot2(db='MA_M2M_0706_R',ProbeSetID='1447335_x_at',CellID='',db2='MA_M2M_0706_R',ProbeSetID2='1438579_at',CellID2='',rank='1')) | [-0.518 38](javascript:showCorrelationPlot2(db='MA_M2M_0706_R',ProbeSetID='1447335_x_at',CellID='',db2='MA_M2M_0706_R',ProbeSetID2='1448102_a_at',CellID2='',rank='1')) | [0.451 38](javascript:showCorrelationPlot2(db='MA_M2M_0706_R',ProbeSetID='1447335_x_at',CellID='',db2='MA_M2M_0706_R',ProbeSetID2='1420943_at',CellID2='',rank='1')) | [0.574 38](javascript:showCorrelationPlot2(db='MA_M2M_0706_R',ProbeSetID='1447335_x_at',CellID='',db2='MA_M2M_0706_R',ProbeSetID2='1447326_s_at',CellID2='',rank='1')) |
|  | [Trait 17: MA_M2M_0706_R::1442239_at](javascript:showDatabase2('MA_M2M_0706_R','1442239_at','');)  C2orf29 on Chr 18 @ 50.168944 Mb  human chromosome 2, open reading frame 29 | [-0.668 38](javascript:showCorrelationPlot2(db='MA_M2M_0706_R',ProbeSetID='1442239_at',CellID='',db2='MA_M2M_0706_R',ProbeSetID2='1445101_at',CellID2='',rank='0')) | [0.386 38](javascript:showCorrelationPlot2(db='MA_M2M_0706_R',ProbeSetID='1442239_at',CellID='',db2='MA_M2M_0706_R',ProbeSetID2='1451285_at',CellID2='',rank='0')) | [0.635 38](javascript:showCorrelationPlot2(db='MA_M2M_0706_R',ProbeSetID='1442239_at',CellID='',db2='MA_M2M_0706_R',ProbeSetID2='1441347_at',CellID2='',rank='0')) | [-0.536 38](javascript:showCorrelationPlot2(db='MA_M2M_0706_R',ProbeSetID='1442239_at',CellID='',db2='MA_M2M_0706_R',ProbeSetID2='1426900_at',CellID2='',rank='0')) | [-0.523 38](javascript:showCorrelationPlot2(db='MA_M2M_0706_R',ProbeSetID='1442239_at',CellID='',db2='MA_M2M_0706_R',ProbeSetID2='1434888_a_at',CellID2='',rank='0')) | [-0.397 38](javascript:showCorrelationPlot2(db='MA_M2M_0706_R',ProbeSetID='1442239_at',CellID='',db2='MA_M2M_0706_R',ProbeSetID2='1434773_a_at',CellID2='',rank='0')) | [0.657 38](javascript:showCorrelationPlot2(db='MA_M2M_0706_R',ProbeSetID='1442239_at',CellID='',db2='MA_M2M_0706_R',ProbeSetID2='1452851_at',CellID2='',rank='0')) | [-0.633 38](javascript:showCorrelationPlot2(db='MA_M2M_0706_R',ProbeSetID='1442239_at',CellID='',db2='MA_M2M_0706_R',ProbeSetID2='1441948_x_at',CellID2='',rank='0')) | [0.741 38](javascript:showCorrelationPlot2(db='MA_M2M_0706_R',ProbeSetID='1442239_at',CellID='',db2='MA_M2M_0706_R',ProbeSetID2='1440160_x_at',CellID2='',rank='0')) | [-0.473 38](javascript:showCorrelationPlot2(db='MA_M2M_0706_R',ProbeSetID='1442239_at',CellID='',db2='MA_M2M_0706_R',ProbeSetID2='1424692_at',CellID2='',rank='0')) | [0.674 38](javascript:showCorrelationPlot2(db='MA_M2M_0706_R',ProbeSetID='1442239_at',CellID='',db2='MA_M2M_0706_R',ProbeSetID2='1433112_at',CellID2='',rank='0')) | [-0.514 38](javascript:showCorrelationPlot2(db='MA_M2M_0706_R',ProbeSetID='1442239_at',CellID='',db2='MA_M2M_0706_R',ProbeSetID2='1459962_at',CellID2='',rank='0')) | [0.703 38](javascript:showCorrelationPlot2(db='MA_M2M_0706_R',ProbeSetID='1442239_at',CellID='',db2='MA_M2M_0706_R',ProbeSetID2='1455331_at',CellID2='',rank='0')) | [0.636 38](javascript:showCorrelationPlot2(db='MA_M2M_0706_R',ProbeSetID='1442239_at',CellID='',db2='MA_M2M_0706_R',ProbeSetID2='1447399_at',CellID2='',rank='0')) | [-0.616 38](javascript:showCorrelationPlot2(db='MA_M2M_0706_R',ProbeSetID='1442239_at',CellID='',db2='MA_M2M_0706_R',ProbeSetID2='1434968_a_at',CellID2='',rank='0')) | [0.710 38](javascript:showCorrelationPlot2(db='MA_M2M_0706_R',ProbeSetID='1442239_at',CellID='',db2='MA_M2M_0706_R',ProbeSetID2='1447335_x_at',CellID2='',rank='0')) | [n 38](javascript:showDatabase2('MA_M2M_0706_R','1442239_at','')) | [0.618 38](javascript:showCorrelationPlot2(db='MA_M2M_0706_R',ProbeSetID='1442239_at',CellID='',db2='MA_M2M_0706_R',ProbeSetID2='1459865_x_at',CellID2='',rank='1')) | [-0.452 38](javascript:showCorrelationPlot2(db='MA_M2M_0706_R',ProbeSetID='1442239_at',CellID='',db2='MA_M2M_0706_R',ProbeSetID2='1438647_x_at',CellID2='',rank='1')) | [0.456 38](javascript:showCorrelationPlot2(db='MA_M2M_0706_R',ProbeSetID='1442239_at',CellID='',db2='MA_M2M_0706_R',ProbeSetID2='1432333_a_at',CellID2='',rank='1')) | [-0.444 38](javascript:showCorrelationPlot2(db='MA_M2M_0706_R',ProbeSetID='1442239_at',CellID='',db2='MA_M2M_0706_R',ProbeSetID2='1444028_s_at',CellID2='',rank='1')) | [0.657 38](javascript:showCorrelationPlot2(db='MA_M2M_0706_R',ProbeSetID='1442239_at',CellID='',db2='MA_M2M_0706_R',ProbeSetID2='1460420_a_at',CellID2='',rank='1')) | [0.629 38](javascript:showCorrelationPlot2(db='MA_M2M_0706_R',ProbeSetID='1442239_at',CellID='',db2='MA_M2M_0706_R',ProbeSetID2='1451530_at',CellID2='',rank='1')) | [-0.557 38](javascript:showCorrelationPlot2(db='MA_M2M_0706_R',ProbeSetID='1442239_at',CellID='',db2='MA_M2M_0706_R',ProbeSetID2='1423785_at',CellID2='',rank='1')) | [0.397 38](javascript:showCorrelationPlot2(db='MA_M2M_0706_R',ProbeSetID='1442239_at',CellID='',db2='MA_M2M_0706_R',ProbeSetID2='1431972_a_at',CellID2='',rank='1')) | [-0.523 38](javascript:showCorrelationPlot2(db='MA_M2M_0706_R',ProbeSetID='1442239_at',CellID='',db2='MA_M2M_0706_R',ProbeSetID2='1448347_a_at',CellID2='',rank='1')) | [0.571 38](javascript:showCorrelationPlot2(db='MA_M2M_0706_R',ProbeSetID='1442239_at',CellID='',db2='MA_M2M_0706_R',ProbeSetID2='1418492_at',CellID2='',rank='1')) | [-0.407 38](javascript:showCorrelationPlot2(db='MA_M2M_0706_R',ProbeSetID='1442239_at',CellID='',db2='MA_M2M_0706_R',ProbeSetID2='1427185_at',CellID2='',rank='1')) | [-0.562 38](javascript:showCorrelationPlot2(db='MA_M2M_0706_R',ProbeSetID='1442239_at',CellID='',db2='MA_M2M_0706_R',ProbeSetID2='1430500_s_at',CellID2='',rank='1')) | [0.572 38](javascript:showCorrelationPlot2(db='MA_M2M_0706_R',ProbeSetID='1442239_at',CellID='',db2='MA_M2M_0706_R',ProbeSetID2='1447549_x_at',CellID2='',rank='1')) | [-0.420 38](javascript:showCorrelationPlot2(db='MA_M2M_0706_R',ProbeSetID='1442239_at',CellID='',db2='MA_M2M_0706_R',ProbeSetID2='1448943_at',CellID2='',rank='1')) | [0.686 38](javascript:showCorrelationPlot2(db='MA_M2M_0706_R',ProbeSetID='1442239_at',CellID='',db2='MA_M2M_0706_R',ProbeSetID2='1433162_at',CellID2='',rank='1')) | [0.704 38](javascript:showCorrelationPlot2(db='MA_M2M_0706_R',ProbeSetID='1442239_at',CellID='',db2='MA_M2M_0706_R',ProbeSetID2='1443494_at',CellID2='',rank='1')) | [-0.324 38](javascript:showCorrelationPlot2(db='MA_M2M_0706_R',ProbeSetID='1442239_at',CellID='',db2='MA_M2M_0706_R',ProbeSetID2='1417426_at',CellID2='',rank='1')) | [0.706 38](javascript:showCorrelationPlot2(db='MA_M2M_0706_R',ProbeSetID='1442239_at',CellID='',db2='MA_M2M_0706_R',ProbeSetID2='1416588_at',CellID2='',rank='1')) | [-0.422 38](javascript:showCorrelationPlot2(db='MA_M2M_0706_R',ProbeSetID='1442239_at',CellID='',db2='MA_M2M_0706_R',ProbeSetID2='1450994_at',CellID2='',rank='1')) | [0.646 38](javascript:showCorrelationPlot2(db='MA_M2M_0706_R',ProbeSetID='1442239_at',CellID='',db2='MA_M2M_0706_R',ProbeSetID2='1420304_x_at',CellID2='',rank='1')) | [-0.668 38](javascript:showCorrelationPlot2(db='MA_M2M_0706_R',ProbeSetID='1442239_at',CellID='',db2='MA_M2M_0706_R',ProbeSetID2='1416500_at',CellID2='',rank='1')) | [0.761 38](javascript:showCorrelationPlot2(db='MA_M2M_0706_R',ProbeSetID='1442239_at',CellID='',db2='MA_M2M_0706_R',ProbeSetID2='1424199_at',CellID2='',rank='1')) | [-0.414 38](javascript:showCorrelationPlot2(db='MA_M2M_0706_R',ProbeSetID='1442239_at',CellID='',db2='MA_M2M_0706_R',ProbeSetID2='1437995_x_at',CellID2='',rank='1')) | [-0.543 38](javascript:showCorrelationPlot2(db='MA_M2M_0706_R',ProbeSetID='1442239_at',CellID='',db2='MA_M2M_0706_R',ProbeSetID2='1427914_a_at',CellID2='',rank='1')) | [0.644 38](javascript:showCorrelationPlot2(db='MA_M2M_0706_R',ProbeSetID='1442239_at',CellID='',db2='MA_M2M_0706_R',ProbeSetID2='1420570_x_at',CellID2='',rank='1')) | [0.777 38](javascript:showCorrelationPlot2(db='MA_M2M_0706_R',ProbeSetID='1442239_at',CellID='',db2='MA_M2M_0706_R',ProbeSetID2='1431015_at',CellID2='',rank='1')) | [-0.523 38](javascript:showCorrelationPlot2(db='MA_M2M_0706_R',ProbeSetID='1442239_at',CellID='',db2='MA_M2M_0706_R',ProbeSetID2='1423852_at',CellID2='',rank='1')) | [0.401 38](javascript:showCorrelationPlot2(db='MA_M2M_0706_R',ProbeSetID='1442239_at',CellID='',db2='MA_M2M_0706_R',ProbeSetID2='1431799_at',CellID2='',rank='1')) | [-0.572 38](javascript:showCorrelationPlot2(db='MA_M2M_0706_R',ProbeSetID='1442239_at',CellID='',db2='MA_M2M_0706_R',ProbeSetID2='1423898_a_at',CellID2='',rank='1')) | [0.603 38](javascript:showCorrelationPlot2(db='MA_M2M_0706_R',ProbeSetID='1442239_at',CellID='',db2='MA_M2M_0706_R',ProbeSetID2='1438579_at',CellID2='',rank='1')) | [-0.604 38](javascript:showCorrelationPlot2(db='MA_M2M_0706_R',ProbeSetID='1442239_at',CellID='',db2='MA_M2M_0706_R',ProbeSetID2='1448102_a_at',CellID2='',rank='1')) | [0.472 38](javascript:showCorrelationPlot2(db='MA_M2M_0706_R',ProbeSetID='1442239_at',CellID='',db2='MA_M2M_0706_R',ProbeSetID2='1420943_at',CellID2='',rank='1')) | [0.649 38](javascript:showCorrelationPlot2(db='MA_M2M_0706_R',ProbeSetID='1442239_at',CellID='',db2='MA_M2M_0706_R',ProbeSetID2='1447326_s_at',CellID2='',rank='1')) |
|  | [Trait 18: MA_M2M_0706_R::1459865_x_at](javascript:showDatabase2('MA_M2M_0706_R','1459865_x_at','');)  Ces7 on Chr 8 @ 96.015941 Mb  carboxylesterase 7 (lens-associated)  3' UTR of short form | [-0.504 38](javascript:showCorrelationPlot2(db='MA_M2M_0706_R',ProbeSetID='1459865_x_at',CellID='',db2='MA_M2M_0706_R',ProbeSetID2='1445101_at',CellID2='',rank='0')) | [0.319 38](javascript:showCorrelationPlot2(db='MA_M2M_0706_R',ProbeSetID='1459865_x_at',CellID='',db2='MA_M2M_0706_R',ProbeSetID2='1451285_at',CellID2='',rank='0')) | [0.442 38](javascript:showCorrelationPlot2(db='MA_M2M_0706_R',ProbeSetID='1459865_x_at',CellID='',db2='MA_M2M_0706_R',ProbeSetID2='1441347_at',CellID2='',rank='0')) | [-0.584 38](javascript:showCorrelationPlot2(db='MA_M2M_0706_R',ProbeSetID='1459865_x_at',CellID='',db2='MA_M2M_0706_R',ProbeSetID2='1426900_at',CellID2='',rank='0')) | [-0.646 38](javascript:showCorrelationPlot2(db='MA_M2M_0706_R',ProbeSetID='1459865_x_at',CellID='',db2='MA_M2M_0706_R',ProbeSetID2='1434888_a_at',CellID2='',rank='0')) | [-0.560 38](javascript:showCorrelationPlot2(db='MA_M2M_0706_R',ProbeSetID='1459865_x_at',CellID='',db2='MA_M2M_0706_R',ProbeSetID2='1434773_a_at',CellID2='',rank='0')) | [0.588 38](javascript:showCorrelationPlot2(db='MA_M2M_0706_R',ProbeSetID='1459865_x_at',CellID='',db2='MA_M2M_0706_R',ProbeSetID2='1452851_at',CellID2='',rank='0')) | [-0.413 38](javascript:showCorrelationPlot2(db='MA_M2M_0706_R',ProbeSetID='1459865_x_at',CellID='',db2='MA_M2M_0706_R',ProbeSetID2='1441948_x_at',CellID2='',rank='0')) | [0.719 38](javascript:showCorrelationPlot2(db='MA_M2M_0706_R',ProbeSetID='1459865_x_at',CellID='',db2='MA_M2M_0706_R',ProbeSetID2='1440160_x_at',CellID2='',rank='0')) | [-0.590 38](javascript:showCorrelationPlot2(db='MA_M2M_0706_R',ProbeSetID='1459865_x_at',CellID='',db2='MA_M2M_0706_R',ProbeSetID2='1424692_at',CellID2='',rank='0')) | [0.508 38](javascript:showCorrelationPlot2(db='MA_M2M_0706_R',ProbeSetID='1459865_x_at',CellID='',db2='MA_M2M_0706_R',ProbeSetID2='1433112_at',CellID2='',rank='0')) | [-0.379 38](javascript:showCorrelationPlot2(db='MA_M2M_0706_R',ProbeSetID='1459865_x_at',CellID='',db2='MA_M2M_0706_R',ProbeSetID2='1459962_at',CellID2='',rank='0')) | [0.457 38](javascript:showCorrelationPlot2(db='MA_M2M_0706_R',ProbeSetID='1459865_x_at',CellID='',db2='MA_M2M_0706_R',ProbeSetID2='1455331_at',CellID2='',rank='0')) | [0.517 38](javascript:showCorrelationPlot2(db='MA_M2M_0706_R',ProbeSetID='1459865_x_at',CellID='',db2='MA_M2M_0706_R',ProbeSetID2='1447399_at',CellID2='',rank='0')) | [-0.505 38](javascript:showCorrelationPlot2(db='MA_M2M_0706_R',ProbeSetID='1459865_x_at',CellID='',db2='MA_M2M_0706_R',ProbeSetID2='1434968_a_at',CellID2='',rank='0')) | [0.683 38](javascript:showCorrelationPlot2(db='MA_M2M_0706_R',ProbeSetID='1459865_x_at',CellID='',db2='MA_M2M_0706_R',ProbeSetID2='1447335_x_at',CellID2='',rank='0')) | [0.589 38](javascript:showCorrelationPlot2(db='MA_M2M_0706_R',ProbeSetID='1459865_x_at',CellID='',db2='MA_M2M_0706_R',ProbeSetID2='1442239_at',CellID2='',rank='0')) | [n 38](javascript:showDatabase2('MA_M2M_0706_R','1459865_x_at','')) | [-0.596 38](javascript:showCorrelationPlot2(db='MA_M2M_0706_R',ProbeSetID='1459865_x_at',CellID='',db2='MA_M2M_0706_R',ProbeSetID2='1438647_x_at',CellID2='',rank='1')) | [0.393 38](javascript:showCorrelationPlot2(db='MA_M2M_0706_R',ProbeSetID='1459865_x_at',CellID='',db2='MA_M2M_0706_R',ProbeSetID2='1432333_a_at',CellID2='',rank='1')) | [-0.306 38](javascript:showCorrelationPlot2(db='MA_M2M_0706_R',ProbeSetID='1459865_x_at',CellID='',db2='MA_M2M_0706_R',ProbeSetID2='1444028_s_at',CellID2='',rank='1')) | [0.592 38](javascript:showCorrelationPlot2(db='MA_M2M_0706_R',ProbeSetID='1459865_x_at',CellID='',db2='MA_M2M_0706_R',ProbeSetID2='1460420_a_at',CellID2='',rank='1')) | [0.312 38](javascript:showCorrelationPlot2(db='MA_M2M_0706_R',ProbeSetID='1459865_x_at',CellID='',db2='MA_M2M_0706_R',ProbeSetID2='1451530_at',CellID2='',rank='1')) | [-0.470 38](javascript:showCorrelationPlot2(db='MA_M2M_0706_R',ProbeSetID='1459865_x_at',CellID='',db2='MA_M2M_0706_R',ProbeSetID2='1423785_at',CellID2='',rank='1')) | [0.404 38](javascript:showCorrelationPlot2(db='MA_M2M_0706_R',ProbeSetID='1459865_x_at',CellID='',db2='MA_M2M_0706_R',ProbeSetID2='1431972_a_at',CellID2='',rank='1')) | [-0.414 38](javascript:showCorrelationPlot2(db='MA_M2M_0706_R',ProbeSetID='1459865_x_at',CellID='',db2='MA_M2M_0706_R',ProbeSetID2='1448347_a_at',CellID2='',rank='1')) | [0.626 38](javascript:showCorrelationPlot2(db='MA_M2M_0706_R',ProbeSetID='1459865_x_at',CellID='',db2='MA_M2M_0706_R',ProbeSetID2='1418492_at',CellID2='',rank='1')) | [-0.458 38](javascript:showCorrelationPlot2(db='MA_M2M_0706_R',ProbeSetID='1459865_x_at',CellID='',db2='MA_M2M_0706_R',ProbeSetID2='1427185_at',CellID2='',rank='1')) | [-0.636 38](javascript:showCorrelationPlot2(db='MA_M2M_0706_R',ProbeSetID='1459865_x_at',CellID='',db2='MA_M2M_0706_R',ProbeSetID2='1430500_s_at',CellID2='',rank='1')) | [0.509 38](javascript:showCorrelationPlot2(db='MA_M2M_0706_R',ProbeSetID='1459865_x_at',CellID='',db2='MA_M2M_0706_R',ProbeSetID2='1447549_x_at',CellID2='',rank='1')) | [-0.461 38](javascript:showCorrelationPlot2(db='MA_M2M_0706_R',ProbeSetID='1459865_x_at',CellID='',db2='MA_M2M_0706_R',ProbeSetID2='1448943_at',CellID2='',rank='1')) | [0.716 38](javascript:showCorrelationPlot2(db='MA_M2M_0706_R',ProbeSetID='1459865_x_at',CellID='',db2='MA_M2M_0706_R',ProbeSetID2='1433162_at',CellID2='',rank='1')) | [0.538 38](javascript:showCorrelationPlot2(db='MA_M2M_0706_R',ProbeSetID='1459865_x_at',CellID='',db2='MA_M2M_0706_R',ProbeSetID2='1443494_at',CellID2='',rank='1')) | [-0.391 38](javascript:showCorrelationPlot2(db='MA_M2M_0706_R',ProbeSetID='1459865_x_at',CellID='',db2='MA_M2M_0706_R',ProbeSetID2='1417426_at',CellID2='',rank='1')) | [0.633 38](javascript:showCorrelationPlot2(db='MA_M2M_0706_R',ProbeSetID='1459865_x_at',CellID='',db2='MA_M2M_0706_R',ProbeSetID2='1416588_at',CellID2='',rank='1')) | [-0.626 38](javascript:showCorrelationPlot2(db='MA_M2M_0706_R',ProbeSetID='1459865_x_at',CellID='',db2='MA_M2M_0706_R',ProbeSetID2='1450994_at',CellID2='',rank='1')) | [0.503 38](javascript:showCorrelationPlot2(db='MA_M2M_0706_R',ProbeSetID='1459865_x_at',CellID='',db2='MA_M2M_0706_R',ProbeSetID2='1420304_x_at',CellID2='',rank='1')) | [-0.494 38](javascript:showCorrelationPlot2(db='MA_M2M_0706_R',ProbeSetID='1459865_x_at',CellID='',db2='MA_M2M_0706_R',ProbeSetID2='1416500_at',CellID2='',rank='1')) | [0.627 38](javascript:showCorrelationPlot2(db='MA_M2M_0706_R',ProbeSetID='1459865_x_at',CellID='',db2='MA_M2M_0706_R',ProbeSetID2='1424199_at',CellID2='',rank='1')) | [-0.456 38](javascript:showCorrelationPlot2(db='MA_M2M_0706_R',ProbeSetID='1459865_x_at',CellID='',db2='MA_M2M_0706_R',ProbeSetID2='1437995_x_at',CellID2='',rank='1')) | [-0.610 38](javascript:showCorrelationPlot2(db='MA_M2M_0706_R',ProbeSetID='1459865_x_at',CellID='',db2='MA_M2M_0706_R',ProbeSetID2='1427914_a_at',CellID2='',rank='1')) | [0.704 38](javascript:showCorrelationPlot2(db='MA_M2M_0706_R',ProbeSetID='1459865_x_at',CellID='',db2='MA_M2M_0706_R',ProbeSetID2='1420570_x_at',CellID2='',rank='1')) | [0.539 38](javascript:showCorrelationPlot2(db='MA_M2M_0706_R',ProbeSetID='1459865_x_at',CellID='',db2='MA_M2M_0706_R',ProbeSetID2='1431015_at',CellID2='',rank='1')) | [-0.319 38](javascript:showCorrelationPlot2(db='MA_M2M_0706_R',ProbeSetID='1459865_x_at',CellID='',db2='MA_M2M_0706_R',ProbeSetID2='1423852_at',CellID2='',rank='1')) | [0.675 38](javascript:showCorrelationPlot2(db='MA_M2M_0706_R',ProbeSetID='1459865_x_at',CellID='',db2='MA_M2M_0706_R',ProbeSetID2='1431799_at',CellID2='',rank='1')) | [-0.617 38](javascript:showCorrelationPlot2(db='MA_M2M_0706_R',ProbeSetID='1459865_x_at',CellID='',db2='MA_M2M_0706_R',ProbeSetID2='1423898_a_at',CellID2='',rank='1')) | [0.413 38](javascript:showCorrelationPlot2(db='MA_M2M_0706_R',ProbeSetID='1459865_x_at',CellID='',db2='MA_M2M_0706_R',ProbeSetID2='1438579_at',CellID2='',rank='1')) | [-0.427 38](javascript:showCorrelationPlot2(db='MA_M2M_0706_R',ProbeSetID='1459865_x_at',CellID='',db2='MA_M2M_0706_R',ProbeSetID2='1448102_a_at',CellID2='',rank='1')) | [0.298 38](javascript:showCorrelationPlot2(db='MA_M2M_0706_R',ProbeSetID='1459865_x_at',CellID='',db2='MA_M2M_0706_R',ProbeSetID2='1420943_at',CellID2='',rank='1')) | [0.536 38](javascript:showCorrelationPlot2(db='MA_M2M_0706_R',ProbeSetID='1459865_x_at',CellID='',db2='MA_M2M_0706_R',ProbeSetID2='1447326_s_at',CellID2='',rank='1')) |
|  | [Trait 19: MA_M2M_0706_R::1438647_x_at](javascript:showDatabase2('MA_M2M_0706_R','1438647_x_at','');)  Cetn2 on Chr X @ 70.159085 Mb  centrin 2 | [0.472 38](javascript:showCorrelationPlot2(db='MA_M2M_0706_R',ProbeSetID='1438647_x_at',CellID='',db2='MA_M2M_0706_R',ProbeSetID2='1445101_at',CellID2='',rank='0')) | [-0.574 38](javascript:showCorrelationPlot2(db='MA_M2M_0706_R',ProbeSetID='1438647_x_at',CellID='',db2='MA_M2M_0706_R',ProbeSetID2='1451285_at',CellID2='',rank='0')) | [-0.625 38](javascript:showCorrelationPlot2(db='MA_M2M_0706_R',ProbeSetID='1438647_x_at',CellID='',db2='MA_M2M_0706_R',ProbeSetID2='1441347_at',CellID2='',rank='0')) | [0.387 38](javascript:showCorrelationPlot2(db='MA_M2M_0706_R',ProbeSetID='1438647_x_at',CellID='',db2='MA_M2M_0706_R',ProbeSetID2='1426900_at',CellID2='',rank='0')) | [0.666 38](javascript:showCorrelationPlot2(db='MA_M2M_0706_R',ProbeSetID='1438647_x_at',CellID='',db2='MA_M2M_0706_R',ProbeSetID2='1434888_a_at',CellID2='',rank='0')) | [0.604 38](javascript:showCorrelationPlot2(db='MA_M2M_0706_R',ProbeSetID='1438647_x_at',CellID='',db2='MA_M2M_0706_R',ProbeSetID2='1434773_a_at',CellID2='',rank='0')) | [-0.371 38](javascript:showCorrelationPlot2(db='MA_M2M_0706_R',ProbeSetID='1438647_x_at',CellID='',db2='MA_M2M_0706_R',ProbeSetID2='1452851_at',CellID2='',rank='0')) | [0.640 38](javascript:showCorrelationPlot2(db='MA_M2M_0706_R',ProbeSetID='1438647_x_at',CellID='',db2='MA_M2M_0706_R',ProbeSetID2='1441948_x_at',CellID2='',rank='0')) | [-0.497 38](javascript:showCorrelationPlot2(db='MA_M2M_0706_R',ProbeSetID='1438647_x_at',CellID='',db2='MA_M2M_0706_R',ProbeSetID2='1440160_x_at',CellID2='',rank='0')) | [0.538 38](javascript:showCorrelationPlot2(db='MA_M2M_0706_R',ProbeSetID='1438647_x_at',CellID='',db2='MA_M2M_0706_R',ProbeSetID2='1424692_at',CellID2='',rank='0')) | [-0.396 38](javascript:showCorrelationPlot2(db='MA_M2M_0706_R',ProbeSetID='1438647_x_at',CellID='',db2='MA_M2M_0706_R',ProbeSetID2='1433112_at',CellID2='',rank='0')) | [0.521 38](javascript:showCorrelationPlot2(db='MA_M2M_0706_R',ProbeSetID='1438647_x_at',CellID='',db2='MA_M2M_0706_R',ProbeSetID2='1459962_at',CellID2='',rank='0')) | [-0.261 38](javascript:showCorrelationPlot2(db='MA_M2M_0706_R',ProbeSetID='1438647_x_at',CellID='',db2='MA_M2M_0706_R',ProbeSetID2='1455331_at',CellID2='',rank='0')) | [-0.557 38](javascript:showCorrelationPlot2(db='MA_M2M_0706_R',ProbeSetID='1438647_x_at',CellID='',db2='MA_M2M_0706_R',ProbeSetID2='1447399_at',CellID2='',rank='0')) | [0.367 38](javascript:showCorrelationPlot2(db='MA_M2M_0706_R',ProbeSetID='1438647_x_at',CellID='',db2='MA_M2M_0706_R',ProbeSetID2='1434968_a_at',CellID2='',rank='0')) | [-0.374 38](javascript:showCorrelationPlot2(db='MA_M2M_0706_R',ProbeSetID='1438647_x_at',CellID='',db2='MA_M2M_0706_R',ProbeSetID2='1447335_x_at',CellID2='',rank='0')) | [-0.405 38](javascript:showCorrelationPlot2(db='MA_M2M_0706_R',ProbeSetID='1438647_x_at',CellID='',db2='MA_M2M_0706_R',ProbeSetID2='1442239_at',CellID2='',rank='0')) | [-0.559 38](javascript:showCorrelationPlot2(db='MA_M2M_0706_R',ProbeSetID='1438647_x_at',CellID='',db2='MA_M2M_0706_R',ProbeSetID2='1459865_x_at',CellID2='',rank='0')) | [n 38](javascript:showDatabase2('MA_M2M_0706_R','1438647_x_at','')) | [-0.498 38](javascript:showCorrelationPlot2(db='MA_M2M_0706_R',ProbeSetID='1438647_x_at',CellID='',db2='MA_M2M_0706_R',ProbeSetID2='1432333_a_at',CellID2='',rank='1')) | [0.288 38](javascript:showCorrelationPlot2(db='MA_M2M_0706_R',ProbeSetID='1438647_x_at',CellID='',db2='MA_M2M_0706_R',ProbeSetID2='1444028_s_at',CellID2='',rank='1')) | [-0.586 38](javascript:showCorrelationPlot2(db='MA_M2M_0706_R',ProbeSetID='1438647_x_at',CellID='',db2='MA_M2M_0706_R',ProbeSetID2='1460420_a_at',CellID2='',rank='1')) | [-0.414 38](javascript:showCorrelationPlot2(db='MA_M2M_0706_R',ProbeSetID='1438647_x_at',CellID='',db2='MA_M2M_0706_R',ProbeSetID2='1451530_at',CellID2='',rank='1')) | [0.321 38](javascript:showCorrelationPlot2(db='MA_M2M_0706_R',ProbeSetID='1438647_x_at',CellID='',db2='MA_M2M_0706_R',ProbeSetID2='1423785_at',CellID2='',rank='1')) | [-0.468 38](javascript:showCorrelationPlot2(db='MA_M2M_0706_R',ProbeSetID='1438647_x_at',CellID='',db2='MA_M2M_0706_R',ProbeSetID2='1431972_a_at',CellID2='',rank='1')) | [0.434 38](javascript:showCorrelationPlot2(db='MA_M2M_0706_R',ProbeSetID='1438647_x_at',CellID='',db2='MA_M2M_0706_R',ProbeSetID2='1448347_a_at',CellID2='',rank='1')) | [-0.581 38](javascript:showCorrelationPlot2(db='MA_M2M_0706_R',ProbeSetID='1438647_x_at',CellID='',db2='MA_M2M_0706_R',ProbeSetID2='1418492_at',CellID2='',rank='1')) | [0.439 38](javascript:showCorrelationPlot2(db='MA_M2M_0706_R',ProbeSetID='1438647_x_at',CellID='',db2='MA_M2M_0706_R',ProbeSetID2='1427185_at',CellID2='',rank='1')) | [0.662 38](javascript:showCorrelationPlot2(db='MA_M2M_0706_R',ProbeSetID='1438647_x_at',CellID='',db2='MA_M2M_0706_R',ProbeSetID2='1430500_s_at',CellID2='',rank='1')) | [-0.410 38](javascript:showCorrelationPlot2(db='MA_M2M_0706_R',ProbeSetID='1438647_x_at',CellID='',db2='MA_M2M_0706_R',ProbeSetID2='1447549_x_at',CellID2='',rank='1')) | [0.252 38](javascript:showCorrelationPlot2(db='MA_M2M_0706_R',ProbeSetID='1438647_x_at',CellID='',db2='MA_M2M_0706_R',ProbeSetID2='1448943_at',CellID2='',rank='1')) | [-0.396 38](javascript:showCorrelationPlot2(db='MA_M2M_0706_R',ProbeSetID='1438647_x_at',CellID='',db2='MA_M2M_0706_R',ProbeSetID2='1433162_at',CellID2='',rank='1')) | [-0.583 38](javascript:showCorrelationPlot2(db='MA_M2M_0706_R',ProbeSetID='1438647_x_at',CellID='',db2='MA_M2M_0706_R',ProbeSetID2='1443494_at',CellID2='',rank='1')) | [0.343 38](javascript:showCorrelationPlot2(db='MA_M2M_0706_R',ProbeSetID='1438647_x_at',CellID='',db2='MA_M2M_0706_R',ProbeSetID2='1417426_at',CellID2='',rank='1')) | [-0.515 38](javascript:showCorrelationPlot2(db='MA_M2M_0706_R',ProbeSetID='1438647_x_at',CellID='',db2='MA_M2M_0706_R',ProbeSetID2='1416588_at',CellID2='',rank='1')) | [0.424 38](javascript:showCorrelationPlot2(db='MA_M2M_0706_R',ProbeSetID='1438647_x_at',CellID='',db2='MA_M2M_0706_R',ProbeSetID2='1450994_at',CellID2='',rank='1')) | [-0.637 38](javascript:showCorrelationPlot2(db='MA_M2M_0706_R',ProbeSetID='1438647_x_at',CellID='',db2='MA_M2M_0706_R',ProbeSetID2='1420304_x_at',CellID2='',rank='1')) | [0.486 38](javascript:showCorrelationPlot2(db='MA_M2M_0706_R',ProbeSetID='1438647_x_at',CellID='',db2='MA_M2M_0706_R',ProbeSetID2='1416500_at',CellID2='',rank='1')) | [-0.590 38](javascript:showCorrelationPlot2(db='MA_M2M_0706_R',ProbeSetID='1438647_x_at',CellID='',db2='MA_M2M_0706_R',ProbeSetID2='1424199_at',CellID2='',rank='1')) | [0.516 38](javascript:showCorrelationPlot2(db='MA_M2M_0706_R',ProbeSetID='1438647_x_at',CellID='',db2='MA_M2M_0706_R',ProbeSetID2='1437995_x_at',CellID2='',rank='1')) | [0.576 38](javascript:showCorrelationPlot2(db='MA_M2M_0706_R',ProbeSetID='1438647_x_at',CellID='',db2='MA_M2M_0706_R',ProbeSetID2='1427914_a_at',CellID2='',rank='1')) | [-0.471 38](javascript:showCorrelationPlot2(db='MA_M2M_0706_R',ProbeSetID='1438647_x_at',CellID='',db2='MA_M2M_0706_R',ProbeSetID2='1420570_x_at',CellID2='',rank='1')) | [-0.414 38](javascript:showCorrelationPlot2(db='MA_M2M_0706_R',ProbeSetID='1438647_x_at',CellID='',db2='MA_M2M_0706_R',ProbeSetID2='1431015_at',CellID2='',rank='1')) | [0.374 38](javascript:showCorrelationPlot2(db='MA_M2M_0706_R',ProbeSetID='1438647_x_at',CellID='',db2='MA_M2M_0706_R',ProbeSetID2='1423852_at',CellID2='',rank='1')) | [-0.519 38](javascript:showCorrelationPlot2(db='MA_M2M_0706_R',ProbeSetID='1438647_x_at',CellID='',db2='MA_M2M_0706_R',ProbeSetID2='1431799_at',CellID2='',rank='1')) | [0.528 38](javascript:showCorrelationPlot2(db='MA_M2M_0706_R',ProbeSetID='1438647_x_at',CellID='',db2='MA_M2M_0706_R',ProbeSetID2='1423898_a_at',CellID2='',rank='1')) | [-0.518 38](javascript:showCorrelationPlot2(db='MA_M2M_0706_R',ProbeSetID='1438647_x_at',CellID='',db2='MA_M2M_0706_R',ProbeSetID2='1438579_at',CellID2='',rank='1')) | [0.676 38](javascript:showCorrelationPlot2(db='MA_M2M_0706_R',ProbeSetID='1438647_x_at',CellID='',db2='MA_M2M_0706_R',ProbeSetID2='1448102_a_at',CellID2='',rank='1')) | [-0.538 38](javascript:showCorrelationPlot2(db='MA_M2M_0706_R',ProbeSetID='1438647_x_at',CellID='',db2='MA_M2M_0706_R',ProbeSetID2='1420943_at',CellID2='',rank='1')) | [-0.533 38](javascript:showCorrelationPlot2(db='MA_M2M_0706_R',ProbeSetID='1438647_x_at',CellID='',db2='MA_M2M_0706_R',ProbeSetID2='1447326_s_at',CellID2='',rank='1')) |
|  | [Trait 20: MA_M2M_0706_R::1432333_a_at](javascript:showDatabase2('MA_M2M_0706_R','1432333_a_at','');)  D21orf56 on Chr 10 @ 76.026819 Mb  human chromosome 21 open reading frame 56 | [-0.557 38](javascript:showCorrelationPlot2(db='MA_M2M_0706_R',ProbeSetID='1432333_a_at',CellID='',db2='MA_M2M_0706_R',ProbeSetID2='1445101_at',CellID2='',rank='0')) | [0.574 38](javascript:showCorrelationPlot2(db='MA_M2M_0706_R',ProbeSetID='1432333_a_at',CellID='',db2='MA_M2M_0706_R',ProbeSetID2='1451285_at',CellID2='',rank='0')) | [0.569 38](javascript:showCorrelationPlot2(db='MA_M2M_0706_R',ProbeSetID='1432333_a_at',CellID='',db2='MA_M2M_0706_R',ProbeSetID2='1441347_at',CellID2='',rank='0')) | [-0.383 38](javascript:showCorrelationPlot2(db='MA_M2M_0706_R',ProbeSetID='1432333_a_at',CellID='',db2='MA_M2M_0706_R',ProbeSetID2='1426900_at',CellID2='',rank='0')) | [-0.431 38](javascript:showCorrelationPlot2(db='MA_M2M_0706_R',ProbeSetID='1432333_a_at',CellID='',db2='MA_M2M_0706_R',ProbeSetID2='1434888_a_at',CellID2='',rank='0')) | [-0.626 38](javascript:showCorrelationPlot2(db='MA_M2M_0706_R',ProbeSetID='1432333_a_at',CellID='',db2='MA_M2M_0706_R',ProbeSetID2='1434773_a_at',CellID2='',rank='0')) | [0.515 38](javascript:showCorrelationPlot2(db='MA_M2M_0706_R',ProbeSetID='1432333_a_at',CellID='',db2='MA_M2M_0706_R',ProbeSetID2='1452851_at',CellID2='',rank='0')) | [-0.600 38](javascript:showCorrelationPlot2(db='MA_M2M_0706_R',ProbeSetID='1432333_a_at',CellID='',db2='MA_M2M_0706_R',ProbeSetID2='1441948_x_at',CellID2='',rank='0')) | [0.461 38](javascript:showCorrelationPlot2(db='MA_M2M_0706_R',ProbeSetID='1432333_a_at',CellID='',db2='MA_M2M_0706_R',ProbeSetID2='1440160_x_at',CellID2='',rank='0')) | [-0.425 38](javascript:showCorrelationPlot2(db='MA_M2M_0706_R',ProbeSetID='1432333_a_at',CellID='',db2='MA_M2M_0706_R',ProbeSetID2='1424692_at',CellID2='',rank='0')) | [0.555 38](javascript:showCorrelationPlot2(db='MA_M2M_0706_R',ProbeSetID='1432333_a_at',CellID='',db2='MA_M2M_0706_R',ProbeSetID2='1433112_at',CellID2='',rank='0')) | [-0.496 38](javascript:showCorrelationPlot2(db='MA_M2M_0706_R',ProbeSetID='1432333_a_at',CellID='',db2='MA_M2M_0706_R',ProbeSetID2='1459962_at',CellID2='',rank='0')) | [0.640 38](javascript:showCorrelationPlot2(db='MA_M2M_0706_R',ProbeSetID='1432333_a_at',CellID='',db2='MA_M2M_0706_R',ProbeSetID2='1455331_at',CellID2='',rank='0')) | [0.567 38](javascript:showCorrelationPlot2(db='MA_M2M_0706_R',ProbeSetID='1432333_a_at',CellID='',db2='MA_M2M_0706_R',ProbeSetID2='1447399_at',CellID2='',rank='0')) | [-0.348 38](javascript:showCorrelationPlot2(db='MA_M2M_0706_R',ProbeSetID='1432333_a_at',CellID='',db2='MA_M2M_0706_R',ProbeSetID2='1434968_a_at',CellID2='',rank='0')) | [0.593 38](javascript:showCorrelationPlot2(db='MA_M2M_0706_R',ProbeSetID='1432333_a_at',CellID='',db2='MA_M2M_0706_R',ProbeSetID2='1447335_x_at',CellID2='',rank='0')) | [0.434 38](javascript:showCorrelationPlot2(db='MA_M2M_0706_R',ProbeSetID='1432333_a_at',CellID='',db2='MA_M2M_0706_R',ProbeSetID2='1442239_at',CellID2='',rank='0')) | [0.511 38](javascript:showCorrelationPlot2(db='MA_M2M_0706_R',ProbeSetID='1432333_a_at',CellID='',db2='MA_M2M_0706_R',ProbeSetID2='1459865_x_at',CellID2='',rank='0')) | [-0.497 38](javascript:showCorrelationPlot2(db='MA_M2M_0706_R',ProbeSetID='1432333_a_at',CellID='',db2='MA_M2M_0706_R',ProbeSetID2='1438647_x_at',CellID2='',rank='0')) | [n 38](javascript:showDatabase2('MA_M2M_0706_R','1432333_a_at','')) | [-0.427 38](javascript:showCorrelationPlot2(db='MA_M2M_0706_R',ProbeSetID='1432333_a_at',CellID='',db2='MA_M2M_0706_R',ProbeSetID2='1444028_s_at',CellID2='',rank='1')) | [0.599 38](javascript:showCorrelationPlot2(db='MA_M2M_0706_R',ProbeSetID='1432333_a_at',CellID='',db2='MA_M2M_0706_R',ProbeSetID2='1460420_a_at',CellID2='',rank='1')) | [0.456 38](javascript:showCorrelationPlot2(db='MA_M2M_0706_R',ProbeSetID='1432333_a_at',CellID='',db2='MA_M2M_0706_R',ProbeSetID2='1451530_at',CellID2='',rank='1')) | [-0.641 38](javascript:showCorrelationPlot2(db='MA_M2M_0706_R',ProbeSetID='1432333_a_at',CellID='',db2='MA_M2M_0706_R',ProbeSetID2='1423785_at',CellID2='',rank='1')) | [0.503 38](javascript:showCorrelationPlot2(db='MA_M2M_0706_R',ProbeSetID='1432333_a_at',CellID='',db2='MA_M2M_0706_R',ProbeSetID2='1431972_a_at',CellID2='',rank='1')) | [-0.580 38](javascript:showCorrelationPlot2(db='MA_M2M_0706_R',ProbeSetID='1432333_a_at',CellID='',db2='MA_M2M_0706_R',ProbeSetID2='1448347_a_at',CellID2='',rank='1')) | [0.455 38](javascript:showCorrelationPlot2(db='MA_M2M_0706_R',ProbeSetID='1432333_a_at',CellID='',db2='MA_M2M_0706_R',ProbeSetID2='1418492_at',CellID2='',rank='1')) | [-0.639 38](javascript:showCorrelationPlot2(db='MA_M2M_0706_R',ProbeSetID='1432333_a_at',CellID='',db2='MA_M2M_0706_R',ProbeSetID2='1427185_at',CellID2='',rank='1')) | [-0.375 38](javascript:showCorrelationPlot2(db='MA_M2M_0706_R',ProbeSetID='1432333_a_at',CellID='',db2='MA_M2M_0706_R',ProbeSetID2='1430500_s_at',CellID2='',rank='1')) | [0.425 38](javascript:showCorrelationPlot2(db='MA_M2M_0706_R',ProbeSetID='1432333_a_at',CellID='',db2='MA_M2M_0706_R',ProbeSetID2='1447549_x_at',CellID2='',rank='1')) | [-0.453 38](javascript:showCorrelationPlot2(db='MA_M2M_0706_R',ProbeSetID='1432333_a_at',CellID='',db2='MA_M2M_0706_R',ProbeSetID2='1448943_at',CellID2='',rank='1')) | [0.545 38](javascript:showCorrelationPlot2(db='MA_M2M_0706_R',ProbeSetID='1432333_a_at',CellID='',db2='MA_M2M_0706_R',ProbeSetID2='1433162_at',CellID2='',rank='1')) | [0.436 38](javascript:showCorrelationPlot2(db='MA_M2M_0706_R',ProbeSetID='1432333_a_at',CellID='',db2='MA_M2M_0706_R',ProbeSetID2='1443494_at',CellID2='',rank='1')) | [-0.343 38](javascript:showCorrelationPlot2(db='MA_M2M_0706_R',ProbeSetID='1432333_a_at',CellID='',db2='MA_M2M_0706_R',ProbeSetID2='1417426_at',CellID2='',rank='1')) | [0.598 38](javascript:showCorrelationPlot2(db='MA_M2M_0706_R',ProbeSetID='1432333_a_at',CellID='',db2='MA_M2M_0706_R',ProbeSetID2='1416588_at',CellID2='',rank='1')) | [-0.309 38](javascript:showCorrelationPlot2(db='MA_M2M_0706_R',ProbeSetID='1432333_a_at',CellID='',db2='MA_M2M_0706_R',ProbeSetID2='1450994_at',CellID2='',rank='1')) | [0.455 38](javascript:showCorrelationPlot2(db='MA_M2M_0706_R',ProbeSetID='1432333_a_at',CellID='',db2='MA_M2M_0706_R',ProbeSetID2='1420304_x_at',CellID2='',rank='1')) | [-0.644 38](javascript:showCorrelationPlot2(db='MA_M2M_0706_R',ProbeSetID='1432333_a_at',CellID='',db2='MA_M2M_0706_R',ProbeSetID2='1416500_at',CellID2='',rank='1')) | [0.609 38](javascript:showCorrelationPlot2(db='MA_M2M_0706_R',ProbeSetID='1432333_a_at',CellID='',db2='MA_M2M_0706_R',ProbeSetID2='1424199_at',CellID2='',rank='1')) | [-0.563 38](javascript:showCorrelationPlot2(db='MA_M2M_0706_R',ProbeSetID='1432333_a_at',CellID='',db2='MA_M2M_0706_R',ProbeSetID2='1437995_x_at',CellID2='',rank='1')) | [-0.269 38](javascript:showCorrelationPlot2(db='MA_M2M_0706_R',ProbeSetID='1432333_a_at',CellID='',db2='MA_M2M_0706_R',ProbeSetID2='1427914_a_at',CellID2='',rank='1')) | [0.375 38](javascript:showCorrelationPlot2(db='MA_M2M_0706_R',ProbeSetID='1432333_a_at',CellID='',db2='MA_M2M_0706_R',ProbeSetID2='1420570_x_at',CellID2='',rank='1')) | [0.367 38](javascript:showCorrelationPlot2(db='MA_M2M_0706_R',ProbeSetID='1432333_a_at',CellID='',db2='MA_M2M_0706_R',ProbeSetID2='1431015_at',CellID2='',rank='1')) | [-0.557 38](javascript:showCorrelationPlot2(db='MA_M2M_0706_R',ProbeSetID='1432333_a_at',CellID='',db2='MA_M2M_0706_R',ProbeSetID2='1423852_at',CellID2='',rank='1')) | [0.304 38](javascript:showCorrelationPlot2(db='MA_M2M_0706_R',ProbeSetID='1432333_a_at',CellID='',db2='MA_M2M_0706_R',ProbeSetID2='1431799_at',CellID2='',rank='1')) | [-0.587 38](javascript:showCorrelationPlot2(db='MA_M2M_0706_R',ProbeSetID='1432333_a_at',CellID='',db2='MA_M2M_0706_R',ProbeSetID2='1423898_a_at',CellID2='',rank='1')) | [0.643 38](javascript:showCorrelationPlot2(db='MA_M2M_0706_R',ProbeSetID='1432333_a_at',CellID='',db2='MA_M2M_0706_R',ProbeSetID2='1438579_at',CellID2='',rank='1')) | [-0.440 38](javascript:showCorrelationPlot2(db='MA_M2M_0706_R',ProbeSetID='1432333_a_at',CellID='',db2='MA_M2M_0706_R',ProbeSetID2='1448102_a_at',CellID2='',rank='1')) | [0.633 38](javascript:showCorrelationPlot2(db='MA_M2M_0706_R',ProbeSetID='1432333_a_at',CellID='',db2='MA_M2M_0706_R',ProbeSetID2='1420943_at',CellID2='',rank='1')) | [0.507 38](javascript:showCorrelationPlot2(db='MA_M2M_0706_R',ProbeSetID='1432333_a_at',CellID='',db2='MA_M2M_0706_R',ProbeSetID2='1447326_s_at',CellID2='',rank='1')) |
|  | [Trait 21: MA_M2M_0706_R::1444028_s_at](javascript:showDatabase2('MA_M2M_0706_R','1444028_s_at','');)  Dock9 on Chr 14 @ 121.941537 Mb  dedicator of cytokinesis 9  mid distal 3' UTR | [0.345 38](javascript:showCorrelationPlot2(db='MA_M2M_0706_R',ProbeSetID='1444028_s_at',CellID='',db2='MA_M2M_0706_R',ProbeSetID2='1445101_at',CellID2='',rank='0')) | [-0.432 38](javascript:showCorrelationPlot2(db='MA_M2M_0706_R',ProbeSetID='1444028_s_at',CellID='',db2='MA_M2M_0706_R',ProbeSetID2='1451285_at',CellID2='',rank='0')) | [-0.402 38](javascript:showCorrelationPlot2(db='MA_M2M_0706_R',ProbeSetID='1444028_s_at',CellID='',db2='MA_M2M_0706_R',ProbeSetID2='1441347_at',CellID2='',rank='0')) | [0.580 38](javascript:showCorrelationPlot2(db='MA_M2M_0706_R',ProbeSetID='1444028_s_at',CellID='',db2='MA_M2M_0706_R',ProbeSetID2='1426900_at',CellID2='',rank='0')) | [0.509 38](javascript:showCorrelationPlot2(db='MA_M2M_0706_R',ProbeSetID='1444028_s_at',CellID='',db2='MA_M2M_0706_R',ProbeSetID2='1434888_a_at',CellID2='',rank='0')) | [0.561 38](javascript:showCorrelationPlot2(db='MA_M2M_0706_R',ProbeSetID='1444028_s_at',CellID='',db2='MA_M2M_0706_R',ProbeSetID2='1434773_a_at',CellID2='',rank='0')) | [-0.486 38](javascript:showCorrelationPlot2(db='MA_M2M_0706_R',ProbeSetID='1444028_s_at',CellID='',db2='MA_M2M_0706_R',ProbeSetID2='1452851_at',CellID2='',rank='0')) | [0.415 38](javascript:showCorrelationPlot2(db='MA_M2M_0706_R',ProbeSetID='1444028_s_at',CellID='',db2='MA_M2M_0706_R',ProbeSetID2='1441948_x_at',CellID2='',rank='0')) | [-0.355 38](javascript:showCorrelationPlot2(db='MA_M2M_0706_R',ProbeSetID='1444028_s_at',CellID='',db2='MA_M2M_0706_R',ProbeSetID2='1440160_x_at',CellID2='',rank='0')) | [0.374 38](javascript:showCorrelationPlot2(db='MA_M2M_0706_R',ProbeSetID='1444028_s_at',CellID='',db2='MA_M2M_0706_R',ProbeSetID2='1424692_at',CellID2='',rank='0')) | [-0.555 38](javascript:showCorrelationPlot2(db='MA_M2M_0706_R',ProbeSetID='1444028_s_at',CellID='',db2='MA_M2M_0706_R',ProbeSetID2='1433112_at',CellID2='',rank='0')) | [0.596 38](javascript:showCorrelationPlot2(db='MA_M2M_0706_R',ProbeSetID='1444028_s_at',CellID='',db2='MA_M2M_0706_R',ProbeSetID2='1459962_at',CellID2='',rank='0')) | [-0.488 38](javascript:showCorrelationPlot2(db='MA_M2M_0706_R',ProbeSetID='1444028_s_at',CellID='',db2='MA_M2M_0706_R',ProbeSetID2='1455331_at',CellID2='',rank='0')) | [-0.526 38](javascript:showCorrelationPlot2(db='MA_M2M_0706_R',ProbeSetID='1444028_s_at',CellID='',db2='MA_M2M_0706_R',ProbeSetID2='1447399_at',CellID2='',rank='0')) | [0.457 38](javascript:showCorrelationPlot2(db='MA_M2M_0706_R',ProbeSetID='1444028_s_at',CellID='',db2='MA_M2M_0706_R',ProbeSetID2='1434968_a_at',CellID2='',rank='0')) | [-0.525 38](javascript:showCorrelationPlot2(db='MA_M2M_0706_R',ProbeSetID='1444028_s_at',CellID='',db2='MA_M2M_0706_R',ProbeSetID2='1447335_x_at',CellID2='',rank='0')) | [-0.485 38](javascript:showCorrelationPlot2(db='MA_M2M_0706_R',ProbeSetID='1444028_s_at',CellID='',db2='MA_M2M_0706_R',ProbeSetID2='1442239_at',CellID2='',rank='0')) | [-0.404 38](javascript:showCorrelationPlot2(db='MA_M2M_0706_R',ProbeSetID='1444028_s_at',CellID='',db2='MA_M2M_0706_R',ProbeSetID2='1459865_x_at',CellID2='',rank='0')) | [0.330 38](javascript:showCorrelationPlot2(db='MA_M2M_0706_R',ProbeSetID='1444028_s_at',CellID='',db2='MA_M2M_0706_R',ProbeSetID2='1438647_x_at',CellID2='',rank='0')) | [-0.500 38](javascript:showCorrelationPlot2(db='MA_M2M_0706_R',ProbeSetID='1444028_s_at',CellID='',db2='MA_M2M_0706_R',ProbeSetID2='1432333_a_at',CellID2='',rank='0')) | [n 38](javascript:showDatabase2('MA_M2M_0706_R','1444028_s_at','')) | [-0.714 38](javascript:showCorrelationPlot2(db='MA_M2M_0706_R',ProbeSetID='1444028_s_at',CellID='',db2='MA_M2M_0706_R',ProbeSetID2='1460420_a_at',CellID2='',rank='1')) | [-0.483 38](javascript:showCorrelationPlot2(db='MA_M2M_0706_R',ProbeSetID='1444028_s_at',CellID='',db2='MA_M2M_0706_R',ProbeSetID2='1451530_at',CellID2='',rank='1')) | [0.544 38](javascript:showCorrelationPlot2(db='MA_M2M_0706_R',ProbeSetID='1444028_s_at',CellID='',db2='MA_M2M_0706_R',ProbeSetID2='1423785_at',CellID2='',rank='1')) | [-0.603 38](javascript:showCorrelationPlot2(db='MA_M2M_0706_R',ProbeSetID='1444028_s_at',CellID='',db2='MA_M2M_0706_R',ProbeSetID2='1431972_a_at',CellID2='',rank='1')) | [0.658 38](javascript:showCorrelationPlot2(db='MA_M2M_0706_R',ProbeSetID='1444028_s_at',CellID='',db2='MA_M2M_0706_R',ProbeSetID2='1448347_a_at',CellID2='',rank='1')) | [-0.275 38](javascript:showCorrelationPlot2(db='MA_M2M_0706_R',ProbeSetID='1444028_s_at',CellID='',db2='MA_M2M_0706_R',ProbeSetID2='1418492_at',CellID2='',rank='1')) | [0.442 38](javascript:showCorrelationPlot2(db='MA_M2M_0706_R',ProbeSetID='1444028_s_at',CellID='',db2='MA_M2M_0706_R',ProbeSetID2='1427185_at',CellID2='',rank='1')) | [0.256 38](javascript:showCorrelationPlot2(db='MA_M2M_0706_R',ProbeSetID='1444028_s_at',CellID='',db2='MA_M2M_0706_R',ProbeSetID2='1430500_s_at',CellID2='',rank='1')) | [-0.362 38](javascript:showCorrelationPlot2(db='MA_M2M_0706_R',ProbeSetID='1444028_s_at',CellID='',db2='MA_M2M_0706_R',ProbeSetID2='1447549_x_at',CellID2='',rank='1')) | [0.701 38](javascript:showCorrelationPlot2(db='MA_M2M_0706_R',ProbeSetID='1444028_s_at',CellID='',db2='MA_M2M_0706_R',ProbeSetID2='1448943_at',CellID2='',rank='1')) | [-0.312 38](javascript:showCorrelationPlot2(db='MA_M2M_0706_R',ProbeSetID='1444028_s_at',CellID='',db2='MA_M2M_0706_R',ProbeSetID2='1433162_at',CellID2='',rank='1')) | [-0.335 38](javascript:showCorrelationPlot2(db='MA_M2M_0706_R',ProbeSetID='1444028_s_at',CellID='',db2='MA_M2M_0706_R',ProbeSetID2='1443494_at',CellID2='',rank='1')) | [0.561 38](javascript:showCorrelationPlot2(db='MA_M2M_0706_R',ProbeSetID='1444028_s_at',CellID='',db2='MA_M2M_0706_R',ProbeSetID2='1417426_at',CellID2='',rank='1')) | [-0.535 38](javascript:showCorrelationPlot2(db='MA_M2M_0706_R',ProbeSetID='1444028_s_at',CellID='',db2='MA_M2M_0706_R',ProbeSetID2='1416588_at',CellID2='',rank='1')) | [0.554 38](javascript:showCorrelationPlot2(db='MA_M2M_0706_R',ProbeSetID='1444028_s_at',CellID='',db2='MA_M2M_0706_R',ProbeSetID2='1450994_at',CellID2='',rank='1')) | [-0.588 38](javascript:showCorrelationPlot2(db='MA_M2M_0706_R',ProbeSetID='1444028_s_at',CellID='',db2='MA_M2M_0706_R',ProbeSetID2='1420304_x_at',CellID2='',rank='1')) | [0.379 38](javascript:showCorrelationPlot2(db='MA_M2M_0706_R',ProbeSetID='1444028_s_at',CellID='',db2='MA_M2M_0706_R',ProbeSetID2='1416500_at',CellID2='',rank='1')) | [-0.338 38](javascript:showCorrelationPlot2(db='MA_M2M_0706_R',ProbeSetID='1444028_s_at',CellID='',db2='MA_M2M_0706_R',ProbeSetID2='1424199_at',CellID2='',rank='1')) | [0.577 38](javascript:showCorrelationPlot2(db='MA_M2M_0706_R',ProbeSetID='1444028_s_at',CellID='',db2='MA_M2M_0706_R',ProbeSetID2='1437995_x_at',CellID2='',rank='1')) | [0.427 38](javascript:showCorrelationPlot2(db='MA_M2M_0706_R',ProbeSetID='1444028_s_at',CellID='',db2='MA_M2M_0706_R',ProbeSetID2='1427914_a_at',CellID2='',rank='1')) | [-0.342 38](javascript:showCorrelationPlot2(db='MA_M2M_0706_R',ProbeSetID='1444028_s_at',CellID='',db2='MA_M2M_0706_R',ProbeSetID2='1420570_x_at',CellID2='',rank='1')) | [-0.570 38](javascript:showCorrelationPlot2(db='MA_M2M_0706_R',ProbeSetID='1444028_s_at',CellID='',db2='MA_M2M_0706_R',ProbeSetID2='1431015_at',CellID2='',rank='1')) | [0.476 38](javascript:showCorrelationPlot2(db='MA_M2M_0706_R',ProbeSetID='1444028_s_at',CellID='',db2='MA_M2M_0706_R',ProbeSetID2='1423852_at',CellID2='',rank='1')) | [-0.542 38](javascript:showCorrelationPlot2(db='MA_M2M_0706_R',ProbeSetID='1444028_s_at',CellID='',db2='MA_M2M_0706_R',ProbeSetID2='1431799_at',CellID2='',rank='1')) | [0.432 38](javascript:showCorrelationPlot2(db='MA_M2M_0706_R',ProbeSetID='1444028_s_at',CellID='',db2='MA_M2M_0706_R',ProbeSetID2='1423898_a_at',CellID2='',rank='1')) | [-0.486 38](javascript:showCorrelationPlot2(db='MA_M2M_0706_R',ProbeSetID='1444028_s_at',CellID='',db2='MA_M2M_0706_R',ProbeSetID2='1438579_at',CellID2='',rank='1')) | [0.468 38](javascript:showCorrelationPlot2(db='MA_M2M_0706_R',ProbeSetID='1444028_s_at',CellID='',db2='MA_M2M_0706_R',ProbeSetID2='1448102_a_at',CellID2='',rank='1')) | [-0.372 38](javascript:showCorrelationPlot2(db='MA_M2M_0706_R',ProbeSetID='1444028_s_at',CellID='',db2='MA_M2M_0706_R',ProbeSetID2='1420943_at',CellID2='',rank='1')) | [-0.447 38](javascript:showCorrelationPlot2(db='MA_M2M_0706_R',ProbeSetID='1444028_s_at',CellID='',db2='MA_M2M_0706_R',ProbeSetID2='1447326_s_at',CellID2='',rank='1')) |
|  | [Trait 22: MA_M2M_0706_R::1460420_a_at](javascript:showDatabase2('MA_M2M_0706_R','1460420_a_at','');)  Egfr on Chr 11 @ 16.781505 Mb  epidermal growth factor receptor | [-0.615 38](javascript:showCorrelationPlot2(db='MA_M2M_0706_R',ProbeSetID='1460420_a_at',CellID='',db2='MA_M2M_0706_R',ProbeSetID2='1445101_at',CellID2='',rank='0')) | [0.595 38](javascript:showCorrelationPlot2(db='MA_M2M_0706_R',ProbeSetID='1460420_a_at',CellID='',db2='MA_M2M_0706_R',ProbeSetID2='1451285_at',CellID2='',rank='0')) | [0.571 38](javascript:showCorrelationPlot2(db='MA_M2M_0706_R',ProbeSetID='1460420_a_at',CellID='',db2='MA_M2M_0706_R',ProbeSetID2='1441347_at',CellID2='',rank='0')) | [-0.607 38](javascript:showCorrelationPlot2(db='MA_M2M_0706_R',ProbeSetID='1460420_a_at',CellID='',db2='MA_M2M_0706_R',ProbeSetID2='1426900_at',CellID2='',rank='0')) | [-0.609 38](javascript:showCorrelationPlot2(db='MA_M2M_0706_R',ProbeSetID='1460420_a_at',CellID='',db2='MA_M2M_0706_R',ProbeSetID2='1434888_a_at',CellID2='',rank='0')) | [-0.621 38](javascript:showCorrelationPlot2(db='MA_M2M_0706_R',ProbeSetID='1460420_a_at',CellID='',db2='MA_M2M_0706_R',ProbeSetID2='1434773_a_at',CellID2='',rank='0')) | [0.597 38](javascript:showCorrelationPlot2(db='MA_M2M_0706_R',ProbeSetID='1460420_a_at',CellID='',db2='MA_M2M_0706_R',ProbeSetID2='1452851_at',CellID2='',rank='0')) | [-0.619 38](javascript:showCorrelationPlot2(db='MA_M2M_0706_R',ProbeSetID='1460420_a_at',CellID='',db2='MA_M2M_0706_R',ProbeSetID2='1441948_x_at',CellID2='',rank='0')) | [0.612 38](javascript:showCorrelationPlot2(db='MA_M2M_0706_R',ProbeSetID='1460420_a_at',CellID='',db2='MA_M2M_0706_R',ProbeSetID2='1440160_x_at',CellID2='',rank='0')) | [-0.611 38](javascript:showCorrelationPlot2(db='MA_M2M_0706_R',ProbeSetID='1460420_a_at',CellID='',db2='MA_M2M_0706_R',ProbeSetID2='1424692_at',CellID2='',rank='0')) | [0.651 38](javascript:showCorrelationPlot2(db='MA_M2M_0706_R',ProbeSetID='1460420_a_at',CellID='',db2='MA_M2M_0706_R',ProbeSetID2='1433112_at',CellID2='',rank='0')) | [-0.645 38](javascript:showCorrelationPlot2(db='MA_M2M_0706_R',ProbeSetID='1460420_a_at',CellID='',db2='MA_M2M_0706_R',ProbeSetID2='1459962_at',CellID2='',rank='0')) | [0.632 38](javascript:showCorrelationPlot2(db='MA_M2M_0706_R',ProbeSetID='1460420_a_at',CellID='',db2='MA_M2M_0706_R',ProbeSetID2='1455331_at',CellID2='',rank='0')) | [0.644 38](javascript:showCorrelationPlot2(db='MA_M2M_0706_R',ProbeSetID='1460420_a_at',CellID='',db2='MA_M2M_0706_R',ProbeSetID2='1447399_at',CellID2='',rank='0')) | [-0.618 38](javascript:showCorrelationPlot2(db='MA_M2M_0706_R',ProbeSetID='1460420_a_at',CellID='',db2='MA_M2M_0706_R',ProbeSetID2='1434968_a_at',CellID2='',rank='0')) | [0.640 38](javascript:showCorrelationPlot2(db='MA_M2M_0706_R',ProbeSetID='1460420_a_at',CellID='',db2='MA_M2M_0706_R',ProbeSetID2='1447335_x_at',CellID2='',rank='0')) | [0.644 38](javascript:showCorrelationPlot2(db='MA_M2M_0706_R',ProbeSetID='1460420_a_at',CellID='',db2='MA_M2M_0706_R',ProbeSetID2='1442239_at',CellID2='',rank='0')) | [0.645 38](javascript:showCorrelationPlot2(db='MA_M2M_0706_R',ProbeSetID='1460420_a_at',CellID='',db2='MA_M2M_0706_R',ProbeSetID2='1459865_x_at',CellID2='',rank='0')) | [-0.603 38](javascript:showCorrelationPlot2(db='MA_M2M_0706_R',ProbeSetID='1460420_a_at',CellID='',db2='MA_M2M_0706_R',ProbeSetID2='1438647_x_at',CellID2='',rank='0')) | [0.629 38](javascript:showCorrelationPlot2(db='MA_M2M_0706_R',ProbeSetID='1460420_a_at',CellID='',db2='MA_M2M_0706_R',ProbeSetID2='1432333_a_at',CellID2='',rank='0')) | [-0.715 38](javascript:showCorrelationPlot2(db='MA_M2M_0706_R',ProbeSetID='1460420_a_at',CellID='',db2='MA_M2M_0706_R',ProbeSetID2='1444028_s_at',CellID2='',rank='0')) | [n 38](javascript:showDatabase2('MA_M2M_0706_R','1460420_a_at','')) | [0.623 38](javascript:showCorrelationPlot2(db='MA_M2M_0706_R',ProbeSetID='1460420_a_at',CellID='',db2='MA_M2M_0706_R',ProbeSetID2='1451530_at',CellID2='',rank='1')) | [-0.642 38](javascript:showCorrelationPlot2(db='MA_M2M_0706_R',ProbeSetID='1460420_a_at',CellID='',db2='MA_M2M_0706_R',ProbeSetID2='1423785_at',CellID2='',rank='1')) | [0.611 38](javascript:showCorrelationPlot2(db='MA_M2M_0706_R',ProbeSetID='1460420_a_at',CellID='',db2='MA_M2M_0706_R',ProbeSetID2='1431972_a_at',CellID2='',rank='1')) | [-0.614 38](javascript:showCorrelationPlot2(db='MA_M2M_0706_R',ProbeSetID='1460420_a_at',CellID='',db2='MA_M2M_0706_R',ProbeSetID2='1448347_a_at',CellID2='',rank='1')) | [0.671 38](javascript:showCorrelationPlot2(db='MA_M2M_0706_R',ProbeSetID='1460420_a_at',CellID='',db2='MA_M2M_0706_R',ProbeSetID2='1418492_at',CellID2='',rank='1')) | [-0.623 38](javascript:showCorrelationPlot2(db='MA_M2M_0706_R',ProbeSetID='1460420_a_at',CellID='',db2='MA_M2M_0706_R',ProbeSetID2='1427185_at',CellID2='',rank='1')) | [-0.585 38](javascript:showCorrelationPlot2(db='MA_M2M_0706_R',ProbeSetID='1460420_a_at',CellID='',db2='MA_M2M_0706_R',ProbeSetID2='1430500_s_at',CellID2='',rank='1')) | [0.607 38](javascript:showCorrelationPlot2(db='MA_M2M_0706_R',ProbeSetID='1460420_a_at',CellID='',db2='MA_M2M_0706_R',ProbeSetID2='1447549_x_at',CellID2='',rank='1')) | [-0.615 38](javascript:showCorrelationPlot2(db='MA_M2M_0706_R',ProbeSetID='1460420_a_at',CellID='',db2='MA_M2M_0706_R',ProbeSetID2='1448943_at',CellID2='',rank='1')) | [0.541 38](javascript:showCorrelationPlot2(db='MA_M2M_0706_R',ProbeSetID='1460420_a_at',CellID='',db2='MA_M2M_0706_R',ProbeSetID2='1433162_at',CellID2='',rank='1')) | [0.623 38](javascript:showCorrelationPlot2(db='MA_M2M_0706_R',ProbeSetID='1460420_a_at',CellID='',db2='MA_M2M_0706_R',ProbeSetID2='1443494_at',CellID2='',rank='1')) | [-0.623 38](javascript:showCorrelationPlot2(db='MA_M2M_0706_R',ProbeSetID='1460420_a_at',CellID='',db2='MA_M2M_0706_R',ProbeSetID2='1417426_at',CellID2='',rank='1')) | [0.682 38](javascript:showCorrelationPlot2(db='MA_M2M_0706_R',ProbeSetID='1460420_a_at',CellID='',db2='MA_M2M_0706_R',ProbeSetID2='1416588_at',CellID2='',rank='1')) | [-0.666 38](javascript:showCorrelationPlot2(db='MA_M2M_0706_R',ProbeSetID='1460420_a_at',CellID='',db2='MA_M2M_0706_R',ProbeSetID2='1450994_at',CellID2='',rank='1')) | [0.714 38](javascript:showCorrelationPlot2(db='MA_M2M_0706_R',ProbeSetID='1460420_a_at',CellID='',db2='MA_M2M_0706_R',ProbeSetID2='1420304_x_at',CellID2='',rank='1')) | [-0.554 38](javascript:showCorrelationPlot2(db='MA_M2M_0706_R',ProbeSetID='1460420_a_at',CellID='',db2='MA_M2M_0706_R',ProbeSetID2='1416500_at',CellID2='',rank='1')) | [0.597 38](javascript:showCorrelationPlot2(db='MA_M2M_0706_R',ProbeSetID='1460420_a_at',CellID='',db2='MA_M2M_0706_R',ProbeSetID2='1424199_at',CellID2='',rank='1')) | [-0.618 38](javascript:showCorrelationPlot2(db='MA_M2M_0706_R',ProbeSetID='1460420_a_at',CellID='',db2='MA_M2M_0706_R',ProbeSetID2='1437995_x_at',CellID2='',rank='1')) | [-0.547 38](javascript:showCorrelationPlot2(db='MA_M2M_0706_R',ProbeSetID='1460420_a_at',CellID='',db2='MA_M2M_0706_R',ProbeSetID2='1427914_a_at',CellID2='',rank='1')) | [0.630 38](javascript:showCorrelationPlot2(db='MA_M2M_0706_R',ProbeSetID='1460420_a_at',CellID='',db2='MA_M2M_0706_R',ProbeSetID2='1420570_x_at',CellID2='',rank='1')) | [0.612 38](javascript:showCorrelationPlot2(db='MA_M2M_0706_R',ProbeSetID='1460420_a_at',CellID='',db2='MA_M2M_0706_R',ProbeSetID2='1431015_at',CellID2='',rank='1')) | [-0.673 38](javascript:showCorrelationPlot2(db='MA_M2M_0706_R',ProbeSetID='1460420_a_at',CellID='',db2='MA_M2M_0706_R',ProbeSetID2='1423852_at',CellID2='',rank='1')) | [0.642 38](javascript:showCorrelationPlot2(db='MA_M2M_0706_R',ProbeSetID='1460420_a_at',CellID='',db2='MA_M2M_0706_R',ProbeSetID2='1431799_at',CellID2='',rank='1')) | [-0.521 38](javascript:showCorrelationPlot2(db='MA_M2M_0706_R',ProbeSetID='1460420_a_at',CellID='',db2='MA_M2M_0706_R',ProbeSetID2='1423898_a_at',CellID2='',rank='1')) | [0.615 38](javascript:showCorrelationPlot2(db='MA_M2M_0706_R',ProbeSetID='1460420_a_at',CellID='',db2='MA_M2M_0706_R',ProbeSetID2='1438579_at',CellID2='',rank='1')) | [-0.644 38](javascript:showCorrelationPlot2(db='MA_M2M_0706_R',ProbeSetID='1460420_a_at',CellID='',db2='MA_M2M_0706_R',ProbeSetID2='1448102_a_at',CellID2='',rank='1')) | [0.613 38](javascript:showCorrelationPlot2(db='MA_M2M_0706_R',ProbeSetID='1460420_a_at',CellID='',db2='MA_M2M_0706_R',ProbeSetID2='1420943_at',CellID2='',rank='1')) | [0.712 38](javascript:showCorrelationPlot2(db='MA_M2M_0706_R',ProbeSetID='1460420_a_at',CellID='',db2='MA_M2M_0706_R',ProbeSetID2='1447326_s_at',CellID2='',rank='1')) |
|  | [Trait 23: MA_M2M_0706_R::1451530_at](javascript:showDatabase2('MA_M2M_0706_R','1451530_at','');)  Egfr on Chr 11 @ 16.809893 Mb  epidermal growth factor receptor  last 3 exons | [-0.524 38](javascript:showCorrelationPlot2(db='MA_M2M_0706_R',ProbeSetID='1451530_at',CellID='',db2='MA_M2M_0706_R',ProbeSetID2='1445101_at',CellID2='',rank='0')) | [0.491 38](javascript:showCorrelationPlot2(db='MA_M2M_0706_R',ProbeSetID='1451530_at',CellID='',db2='MA_M2M_0706_R',ProbeSetID2='1451285_at',CellID2='',rank='0')) | [0.623 38](javascript:showCorrelationPlot2(db='MA_M2M_0706_R',ProbeSetID='1451530_at',CellID='',db2='MA_M2M_0706_R',ProbeSetID2='1441347_at',CellID2='',rank='0')) | [-0.452 38](javascript:showCorrelationPlot2(db='MA_M2M_0706_R',ProbeSetID='1451530_at',CellID='',db2='MA_M2M_0706_R',ProbeSetID2='1426900_at',CellID2='',rank='0')) | [-0.370 38](javascript:showCorrelationPlot2(db='MA_M2M_0706_R',ProbeSetID='1451530_at',CellID='',db2='MA_M2M_0706_R',ProbeSetID2='1434888_a_at',CellID2='',rank='0')) | [-0.546 38](javascript:showCorrelationPlot2(db='MA_M2M_0706_R',ProbeSetID='1451530_at',CellID='',db2='MA_M2M_0706_R',ProbeSetID2='1434773_a_at',CellID2='',rank='0')) | [0.492 38](javascript:showCorrelationPlot2(db='MA_M2M_0706_R',ProbeSetID='1451530_at',CellID='',db2='MA_M2M_0706_R',ProbeSetID2='1452851_at',CellID2='',rank='0')) | [-0.485 38](javascript:showCorrelationPlot2(db='MA_M2M_0706_R',ProbeSetID='1451530_at',CellID='',db2='MA_M2M_0706_R',ProbeSetID2='1441948_x_at',CellID2='',rank='0')) | [0.387 38](javascript:showCorrelationPlot2(db='MA_M2M_0706_R',ProbeSetID='1451530_at',CellID='',db2='MA_M2M_0706_R',ProbeSetID2='1440160_x_at',CellID2='',rank='0')) | [-0.328 38](javascript:showCorrelationPlot2(db='MA_M2M_0706_R',ProbeSetID='1451530_at',CellID='',db2='MA_M2M_0706_R',ProbeSetID2='1424692_at',CellID2='',rank='0')) | [0.701 38](javascript:showCorrelationPlot2(db='MA_M2M_0706_R',ProbeSetID='1451530_at',CellID='',db2='MA_M2M_0706_R',ProbeSetID2='1433112_at',CellID2='',rank='0')) | [-0.564 38](javascript:showCorrelationPlot2(db='MA_M2M_0706_R',ProbeSetID='1451530_at',CellID='',db2='MA_M2M_0706_R',ProbeSetID2='1459962_at',CellID2='',rank='0')) | [0.501 38](javascript:showCorrelationPlot2(db='MA_M2M_0706_R',ProbeSetID='1451530_at',CellID='',db2='MA_M2M_0706_R',ProbeSetID2='1455331_at',CellID2='',rank='0')) | [0.468 38](javascript:showCorrelationPlot2(db='MA_M2M_0706_R',ProbeSetID='1451530_at',CellID='',db2='MA_M2M_0706_R',ProbeSetID2='1447399_at',CellID2='',rank='0')) | [-0.310 38](javascript:showCorrelationPlot2(db='MA_M2M_0706_R',ProbeSetID='1451530_at',CellID='',db2='MA_M2M_0706_R',ProbeSetID2='1434968_a_at',CellID2='',rank='0')) | [0.618 38](javascript:showCorrelationPlot2(db='MA_M2M_0706_R',ProbeSetID='1451530_at',CellID='',db2='MA_M2M_0706_R',ProbeSetID2='1447335_x_at',CellID2='',rank='0')) | [0.619 38](javascript:showCorrelationPlot2(db='MA_M2M_0706_R',ProbeSetID='1451530_at',CellID='',db2='MA_M2M_0706_R',ProbeSetID2='1442239_at',CellID2='',rank='0')) | [0.354 38](javascript:showCorrelationPlot2(db='MA_M2M_0706_R',ProbeSetID='1451530_at',CellID='',db2='MA_M2M_0706_R',ProbeSetID2='1459865_x_at',CellID2='',rank='0')) | [-0.394 38](javascript:showCorrelationPlot2(db='MA_M2M_0706_R',ProbeSetID='1451530_at',CellID='',db2='MA_M2M_0706_R',ProbeSetID2='1438647_x_at',CellID2='',rank='0')) | [0.493 38](javascript:showCorrelationPlot2(db='MA_M2M_0706_R',ProbeSetID='1451530_at',CellID='',db2='MA_M2M_0706_R',ProbeSetID2='1432333_a_at',CellID2='',rank='0')) | [-0.609 38](javascript:showCorrelationPlot2(db='MA_M2M_0706_R',ProbeSetID='1451530_at',CellID='',db2='MA_M2M_0706_R',ProbeSetID2='1444028_s_at',CellID2='',rank='0')) | [0.641 38](javascript:showCorrelationPlot2(db='MA_M2M_0706_R',ProbeSetID='1451530_at',CellID='',db2='MA_M2M_0706_R',ProbeSetID2='1460420_a_at',CellID2='',rank='0')) | [n 38](javascript:showDatabase2('MA_M2M_0706_R','1451530_at','')) | [-0.566 38](javascript:showCorrelationPlot2(db='MA_M2M_0706_R',ProbeSetID='1451530_at',CellID='',db2='MA_M2M_0706_R',ProbeSetID2='1423785_at',CellID2='',rank='1')) | [0.382 38](javascript:showCorrelationPlot2(db='MA_M2M_0706_R',ProbeSetID='1451530_at',CellID='',db2='MA_M2M_0706_R',ProbeSetID2='1431972_a_at',CellID2='',rank='1')) | [-0.534 38](javascript:showCorrelationPlot2(db='MA_M2M_0706_R',ProbeSetID='1451530_at',CellID='',db2='MA_M2M_0706_R',ProbeSetID2='1448347_a_at',CellID2='',rank='1')) | [0.579 38](javascript:showCorrelationPlot2(db='MA_M2M_0706_R',ProbeSetID='1451530_at',CellID='',db2='MA_M2M_0706_R',ProbeSetID2='1418492_at',CellID2='',rank='1')) | [-0.507 38](javascript:showCorrelationPlot2(db='MA_M2M_0706_R',ProbeSetID='1451530_at',CellID='',db2='MA_M2M_0706_R',ProbeSetID2='1427185_at',CellID2='',rank='1')) | [-0.493 38](javascript:showCorrelationPlot2(db='MA_M2M_0706_R',ProbeSetID='1451530_at',CellID='',db2='MA_M2M_0706_R',ProbeSetID2='1430500_s_at',CellID2='',rank='1')) | [0.611 38](javascript:showCorrelationPlot2(db='MA_M2M_0706_R',ProbeSetID='1451530_at',CellID='',db2='MA_M2M_0706_R',ProbeSetID2='1447549_x_at',CellID2='',rank='1')) | [-0.397 38](javascript:showCorrelationPlot2(db='MA_M2M_0706_R',ProbeSetID='1451530_at',CellID='',db2='MA_M2M_0706_R',ProbeSetID2='1448943_at',CellID2='',rank='1')) | [0.547 38](javascript:showCorrelationPlot2(db='MA_M2M_0706_R',ProbeSetID='1451530_at',CellID='',db2='MA_M2M_0706_R',ProbeSetID2='1433162_at',CellID2='',rank='1')) | [0.640 38](javascript:showCorrelationPlot2(db='MA_M2M_0706_R',ProbeSetID='1451530_at',CellID='',db2='MA_M2M_0706_R',ProbeSetID2='1443494_at',CellID2='',rank='1')) | [-0.368 38](javascript:showCorrelationPlot2(db='MA_M2M_0706_R',ProbeSetID='1451530_at',CellID='',db2='MA_M2M_0706_R',ProbeSetID2='1417426_at',CellID2='',rank='1')) | [0.586 38](javascript:showCorrelationPlot2(db='MA_M2M_0706_R',ProbeSetID='1451530_at',CellID='',db2='MA_M2M_0706_R',ProbeSetID2='1416588_at',CellID2='',rank='1')) | [-0.479 38](javascript:showCorrelationPlot2(db='MA_M2M_0706_R',ProbeSetID='1451530_at',CellID='',db2='MA_M2M_0706_R',ProbeSetID2='1450994_at',CellID2='',rank='1')) | [0.773 38](javascript:showCorrelationPlot2(db='MA_M2M_0706_R',ProbeSetID='1451530_at',CellID='',db2='MA_M2M_0706_R',ProbeSetID2='1420304_x_at',CellID2='',rank='1')) | [-0.566 38](javascript:showCorrelationPlot2(db='MA_M2M_0706_R',ProbeSetID='1451530_at',CellID='',db2='MA_M2M_0706_R',ProbeSetID2='1416500_at',CellID2='',rank='1')) | [0.578 38](javascript:showCorrelationPlot2(db='MA_M2M_0706_R',ProbeSetID='1451530_at',CellID='',db2='MA_M2M_0706_R',ProbeSetID2='1424199_at',CellID2='',rank='1')) | [-0.488 38](javascript:showCorrelationPlot2(db='MA_M2M_0706_R',ProbeSetID='1451530_at',CellID='',db2='MA_M2M_0706_R',ProbeSetID2='1437995_x_at',CellID2='',rank='1')) | [-0.420 38](javascript:showCorrelationPlot2(db='MA_M2M_0706_R',ProbeSetID='1451530_at',CellID='',db2='MA_M2M_0706_R',ProbeSetID2='1427914_a_at',CellID2='',rank='1')) | [0.576 38](javascript:showCorrelationPlot2(db='MA_M2M_0706_R',ProbeSetID='1451530_at',CellID='',db2='MA_M2M_0706_R',ProbeSetID2='1420570_x_at',CellID2='',rank='1')) | [0.552 38](javascript:showCorrelationPlot2(db='MA_M2M_0706_R',ProbeSetID='1451530_at',CellID='',db2='MA_M2M_0706_R',ProbeSetID2='1431015_at',CellID2='',rank='1')) | [-0.520 38](javascript:showCorrelationPlot2(db='MA_M2M_0706_R',ProbeSetID='1451530_at',CellID='',db2='MA_M2M_0706_R',ProbeSetID2='1423852_at',CellID2='',rank='1')) | [0.247 38](javascript:showCorrelationPlot2(db='MA_M2M_0706_R',ProbeSetID='1451530_at',CellID='',db2='MA_M2M_0706_R',ProbeSetID2='1431799_at',CellID2='',rank='1')) | [-0.440 38](javascript:showCorrelationPlot2(db='MA_M2M_0706_R',ProbeSetID='1451530_at',CellID='',db2='MA_M2M_0706_R',ProbeSetID2='1423898_a_at',CellID2='',rank='1')) | [0.552 38](javascript:showCorrelationPlot2(db='MA_M2M_0706_R',ProbeSetID='1451530_at',CellID='',db2='MA_M2M_0706_R',ProbeSetID2='1438579_at',CellID2='',rank='1')) | [-0.615 38](javascript:showCorrelationPlot2(db='MA_M2M_0706_R',ProbeSetID='1451530_at',CellID='',db2='MA_M2M_0706_R',ProbeSetID2='1448102_a_at',CellID2='',rank='1')) | [0.617 38](javascript:showCorrelationPlot2(db='MA_M2M_0706_R',ProbeSetID='1451530_at',CellID='',db2='MA_M2M_0706_R',ProbeSetID2='1420943_at',CellID2='',rank='1')) | [0.612 38](javascript:showCorrelationPlot2(db='MA_M2M_0706_R',ProbeSetID='1451530_at',CellID='',db2='MA_M2M_0706_R',ProbeSetID2='1447326_s_at',CellID2='',rank='1')) |
|  | [Trait 24: MA_M2M_0706_R::1423785_at](javascript:showDatabase2('MA_M2M_0706_R','1423785_at','');)  Egln1 on Chr 8 @ 127.432581 Mb  EGL nine homolog 1  distal 3' UTR | [0.552 38](javascript:showCorrelationPlot2(db='MA_M2M_0706_R',ProbeSetID='1423785_at',CellID='',db2='MA_M2M_0706_R',ProbeSetID2='1445101_at',CellID2='',rank='0')) | [-0.446 38](javascript:showCorrelationPlot2(db='MA_M2M_0706_R',ProbeSetID='1423785_at',CellID='',db2='MA_M2M_0706_R',ProbeSetID2='1451285_at',CellID2='',rank='0')) | [-0.588 38](javascript:showCorrelationPlot2(db='MA_M2M_0706_R',ProbeSetID='1423785_at',CellID='',db2='MA_M2M_0706_R',ProbeSetID2='1441347_at',CellID2='',rank='0')) | [0.655 38](javascript:showCorrelationPlot2(db='MA_M2M_0706_R',ProbeSetID='1423785_at',CellID='',db2='MA_M2M_0706_R',ProbeSetID2='1426900_at',CellID2='',rank='0')) | [0.459 38](javascript:showCorrelationPlot2(db='MA_M2M_0706_R',ProbeSetID='1423785_at',CellID='',db2='MA_M2M_0706_R',ProbeSetID2='1434888_a_at',CellID2='',rank='0')) | [0.311 38](javascript:showCorrelationPlot2(db='MA_M2M_0706_R',ProbeSetID='1423785_at',CellID='',db2='MA_M2M_0706_R',ProbeSetID2='1434773_a_at',CellID2='',rank='0')) | [-0.528 38](javascript:showCorrelationPlot2(db='MA_M2M_0706_R',ProbeSetID='1423785_at',CellID='',db2='MA_M2M_0706_R',ProbeSetID2='1452851_at',CellID2='',rank='0')) | [0.428 38](javascript:showCorrelationPlot2(db='MA_M2M_0706_R',ProbeSetID='1423785_at',CellID='',db2='MA_M2M_0706_R',ProbeSetID2='1441948_x_at',CellID2='',rank='0')) | [-0.483 38](javascript:showCorrelationPlot2(db='MA_M2M_0706_R',ProbeSetID='1423785_at',CellID='',db2='MA_M2M_0706_R',ProbeSetID2='1440160_x_at',CellID2='',rank='0')) | [0.420 38](javascript:showCorrelationPlot2(db='MA_M2M_0706_R',ProbeSetID='1423785_at',CellID='',db2='MA_M2M_0706_R',ProbeSetID2='1424692_at',CellID2='',rank='0')) | [-0.458 38](javascript:showCorrelationPlot2(db='MA_M2M_0706_R',ProbeSetID='1423785_at',CellID='',db2='MA_M2M_0706_R',ProbeSetID2='1433112_at',CellID2='',rank='0')) | [0.446 38](javascript:showCorrelationPlot2(db='MA_M2M_0706_R',ProbeSetID='1423785_at',CellID='',db2='MA_M2M_0706_R',ProbeSetID2='1459962_at',CellID2='',rank='0')) | [-0.555 38](javascript:showCorrelationPlot2(db='MA_M2M_0706_R',ProbeSetID='1423785_at',CellID='',db2='MA_M2M_0706_R',ProbeSetID2='1455331_at',CellID2='',rank='0')) | [-0.451 38](javascript:showCorrelationPlot2(db='MA_M2M_0706_R',ProbeSetID='1423785_at',CellID='',db2='MA_M2M_0706_R',ProbeSetID2='1447399_at',CellID2='',rank='0')) | [0.430 38](javascript:showCorrelationPlot2(db='MA_M2M_0706_R',ProbeSetID='1423785_at',CellID='',db2='MA_M2M_0706_R',ProbeSetID2='1434968_a_at',CellID2='',rank='0')) | [-0.577 38](javascript:showCorrelationPlot2(db='MA_M2M_0706_R',ProbeSetID='1423785_at',CellID='',db2='MA_M2M_0706_R',ProbeSetID2='1447335_x_at',CellID2='',rank='0')) | [-0.515 38](javascript:showCorrelationPlot2(db='MA_M2M_0706_R',ProbeSetID='1423785_at',CellID='',db2='MA_M2M_0706_R',ProbeSetID2='1442239_at',CellID2='',rank='0')) | [-0.439 38](javascript:showCorrelationPlot2(db='MA_M2M_0706_R',ProbeSetID='1423785_at',CellID='',db2='MA_M2M_0706_R',ProbeSetID2='1459865_x_at',CellID2='',rank='0')) | [0.313 38](javascript:showCorrelationPlot2(db='MA_M2M_0706_R',ProbeSetID='1423785_at',CellID='',db2='MA_M2M_0706_R',ProbeSetID2='1438647_x_at',CellID2='',rank='0')) | [-0.634 38](javascript:showCorrelationPlot2(db='MA_M2M_0706_R',ProbeSetID='1423785_at',CellID='',db2='MA_M2M_0706_R',ProbeSetID2='1432333_a_at',CellID2='',rank='0')) | [0.517 38](javascript:showCorrelationPlot2(db='MA_M2M_0706_R',ProbeSetID='1423785_at',CellID='',db2='MA_M2M_0706_R',ProbeSetID2='1444028_s_at',CellID2='',rank='0')) | [-0.609 38](javascript:showCorrelationPlot2(db='MA_M2M_0706_R',ProbeSetID='1423785_at',CellID='',db2='MA_M2M_0706_R',ProbeSetID2='1460420_a_at',CellID2='',rank='0')) | [-0.587 38](javascript:showCorrelationPlot2(db='MA_M2M_0706_R',ProbeSetID='1423785_at',CellID='',db2='MA_M2M_0706_R',ProbeSetID2='1451530_at',CellID2='',rank='0')) | [n 38](javascript:showDatabase2('MA_M2M_0706_R','1423785_at','')) | [-0.653 38](javascript:showCorrelationPlot2(db='MA_M2M_0706_R',ProbeSetID='1423785_at',CellID='',db2='MA_M2M_0706_R',ProbeSetID2='1431972_a_at',CellID2='',rank='1')) | [0.584 38](javascript:showCorrelationPlot2(db='MA_M2M_0706_R',ProbeSetID='1423785_at',CellID='',db2='MA_M2M_0706_R',ProbeSetID2='1448347_a_at',CellID2='',rank='1')) | [-0.542 38](javascript:showCorrelationPlot2(db='MA_M2M_0706_R',ProbeSetID='1423785_at',CellID='',db2='MA_M2M_0706_R',ProbeSetID2='1418492_at',CellID2='',rank='1')) | [0.576 38](javascript:showCorrelationPlot2(db='MA_M2M_0706_R',ProbeSetID='1423785_at',CellID='',db2='MA_M2M_0706_R',ProbeSetID2='1427185_at',CellID2='',rank='1')) | [0.424 38](javascript:showCorrelationPlot2(db='MA_M2M_0706_R',ProbeSetID='1423785_at',CellID='',db2='MA_M2M_0706_R',ProbeSetID2='1430500_s_at',CellID2='',rank='1')) | [-0.481 38](javascript:showCorrelationPlot2(db='MA_M2M_0706_R',ProbeSetID='1423785_at',CellID='',db2='MA_M2M_0706_R',ProbeSetID2='1447549_x_at',CellID2='',rank='1')) | [0.511 38](javascript:showCorrelationPlot2(db='MA_M2M_0706_R',ProbeSetID='1423785_at',CellID='',db2='MA_M2M_0706_R',ProbeSetID2='1448943_at',CellID2='',rank='1')) | [-0.602 38](javascript:showCorrelationPlot2(db='MA_M2M_0706_R',ProbeSetID='1423785_at',CellID='',db2='MA_M2M_0706_R',ProbeSetID2='1433162_at',CellID2='',rank='1')) | [-0.614 38](javascript:showCorrelationPlot2(db='MA_M2M_0706_R',ProbeSetID='1423785_at',CellID='',db2='MA_M2M_0706_R',ProbeSetID2='1443494_at',CellID2='',rank='1')) | [0.391 38](javascript:showCorrelationPlot2(db='MA_M2M_0706_R',ProbeSetID='1423785_at',CellID='',db2='MA_M2M_0706_R',ProbeSetID2='1417426_at',CellID2='',rank='1')) | [-0.656 38](javascript:showCorrelationPlot2(db='MA_M2M_0706_R',ProbeSetID='1423785_at',CellID='',db2='MA_M2M_0706_R',ProbeSetID2='1416588_at',CellID2='',rank='1')) | [0.501 38](javascript:showCorrelationPlot2(db='MA_M2M_0706_R',ProbeSetID='1423785_at',CellID='',db2='MA_M2M_0706_R',ProbeSetID2='1450994_at',CellID2='',rank='1')) | [-0.523 38](javascript:showCorrelationPlot2(db='MA_M2M_0706_R',ProbeSetID='1423785_at',CellID='',db2='MA_M2M_0706_R',ProbeSetID2='1420304_x_at',CellID2='',rank='1')) | [0.561 38](javascript:showCorrelationPlot2(db='MA_M2M_0706_R',ProbeSetID='1423785_at',CellID='',db2='MA_M2M_0706_R',ProbeSetID2='1416500_at',CellID2='',rank='1')) | [-0.550 38](javascript:showCorrelationPlot2(db='MA_M2M_0706_R',ProbeSetID='1423785_at',CellID='',db2='MA_M2M_0706_R',ProbeSetID2='1424199_at',CellID2='',rank='1')) | [0.584 38](javascript:showCorrelationPlot2(db='MA_M2M_0706_R',ProbeSetID='1423785_at',CellID='',db2='MA_M2M_0706_R',ProbeSetID2='1437995_x_at',CellID2='',rank='1')) | [0.262 38](javascript:showCorrelationPlot2(db='MA_M2M_0706_R',ProbeSetID='1423785_at',CellID='',db2='MA_M2M_0706_R',ProbeSetID2='1427914_a_at',CellID2='',rank='1')) | [-0.399 38](javascript:showCorrelationPlot2(db='MA_M2M_0706_R',ProbeSetID='1423785_at',CellID='',db2='MA_M2M_0706_R',ProbeSetID2='1420570_x_at',CellID2='',rank='1')) | [-0.550 38](javascript:showCorrelationPlot2(db='MA_M2M_0706_R',ProbeSetID='1423785_at',CellID='',db2='MA_M2M_0706_R',ProbeSetID2='1431015_at',CellID2='',rank='1')) | [0.404 38](javascript:showCorrelationPlot2(db='MA_M2M_0706_R',ProbeSetID='1423785_at',CellID='',db2='MA_M2M_0706_R',ProbeSetID2='1423852_at',CellID2='',rank='1')) | [-0.323 38](javascript:showCorrelationPlot2(db='MA_M2M_0706_R',ProbeSetID='1423785_at',CellID='',db2='MA_M2M_0706_R',ProbeSetID2='1431799_at',CellID2='',rank='1')) | [0.634 38](javascript:showCorrelationPlot2(db='MA_M2M_0706_R',ProbeSetID='1423785_at',CellID='',db2='MA_M2M_0706_R',ProbeSetID2='1423898_a_at',CellID2='',rank='1')) | [-0.553 38](javascript:showCorrelationPlot2(db='MA_M2M_0706_R',ProbeSetID='1423785_at',CellID='',db2='MA_M2M_0706_R',ProbeSetID2='1438579_at',CellID2='',rank='1')) | [0.420 38](javascript:showCorrelationPlot2(db='MA_M2M_0706_R',ProbeSetID='1423785_at',CellID='',db2='MA_M2M_0706_R',ProbeSetID2='1448102_a_at',CellID2='',rank='1')) | [-0.453 38](javascript:showCorrelationPlot2(db='MA_M2M_0706_R',ProbeSetID='1423785_at',CellID='',db2='MA_M2M_0706_R',ProbeSetID2='1420943_at',CellID2='',rank='1')) | [-0.478 38](javascript:showCorrelationPlot2(db='MA_M2M_0706_R',ProbeSetID='1423785_at',CellID='',db2='MA_M2M_0706_R',ProbeSetID2='1447326_s_at',CellID2='',rank='1')) |
|  | [Trait 25: MA_M2M_0706_R::1431972_a_at](javascript:showDatabase2('MA_M2M_0706_R','1431972_a_at','');)  Gcap14 on Chr 14 @ 37.692122 Mb  granule cell antiserum positive 14 | [-0.389 38](javascript:showCorrelationPlot2(db='MA_M2M_0706_R',ProbeSetID='1431972_a_at',CellID='',db2='MA_M2M_0706_R',ProbeSetID2='1445101_at',CellID2='',rank='0')) | [0.462 38](javascript:showCorrelationPlot2(db='MA_M2M_0706_R',ProbeSetID='1431972_a_at',CellID='',db2='MA_M2M_0706_R',ProbeSetID2='1451285_at',CellID2='',rank='0')) | [0.377 38](javascript:showCorrelationPlot2(db='MA_M2M_0706_R',ProbeSetID='1431972_a_at',CellID='',db2='MA_M2M_0706_R',ProbeSetID2='1441347_at',CellID2='',rank='0')) | [-0.474 38](javascript:showCorrelationPlot2(db='MA_M2M_0706_R',ProbeSetID='1431972_a_at',CellID='',db2='MA_M2M_0706_R',ProbeSetID2='1426900_at',CellID2='',rank='0')) | [-0.644 38](javascript:showCorrelationPlot2(db='MA_M2M_0706_R',ProbeSetID='1431972_a_at',CellID='',db2='MA_M2M_0706_R',ProbeSetID2='1434888_a_at',CellID2='',rank='0')) | [-0.554 38](javascript:showCorrelationPlot2(db='MA_M2M_0706_R',ProbeSetID='1431972_a_at',CellID='',db2='MA_M2M_0706_R',ProbeSetID2='1434773_a_at',CellID2='',rank='0')) | [0.405 38](javascript:showCorrelationPlot2(db='MA_M2M_0706_R',ProbeSetID='1431972_a_at',CellID='',db2='MA_M2M_0706_R',ProbeSetID2='1452851_at',CellID2='',rank='0')) | [-0.341 38](javascript:showCorrelationPlot2(db='MA_M2M_0706_R',ProbeSetID='1431972_a_at',CellID='',db2='MA_M2M_0706_R',ProbeSetID2='1441948_x_at',CellID2='',rank='0')) | [0.436 38](javascript:showCorrelationPlot2(db='MA_M2M_0706_R',ProbeSetID='1431972_a_at',CellID='',db2='MA_M2M_0706_R',ProbeSetID2='1440160_x_at',CellID2='',rank='0')) | [-0.339 38](javascript:showCorrelationPlot2(db='MA_M2M_0706_R',ProbeSetID='1431972_a_at',CellID='',db2='MA_M2M_0706_R',ProbeSetID2='1424692_at',CellID2='',rank='0')) | [0.357 38](javascript:showCorrelationPlot2(db='MA_M2M_0706_R',ProbeSetID='1431972_a_at',CellID='',db2='MA_M2M_0706_R',ProbeSetID2='1433112_at',CellID2='',rank='0')) | [-0.489 38](javascript:showCorrelationPlot2(db='MA_M2M_0706_R',ProbeSetID='1431972_a_at',CellID='',db2='MA_M2M_0706_R',ProbeSetID2='1459962_at',CellID2='',rank='0')) | [0.316 38](javascript:showCorrelationPlot2(db='MA_M2M_0706_R',ProbeSetID='1431972_a_at',CellID='',db2='MA_M2M_0706_R',ProbeSetID2='1455331_at',CellID2='',rank='0')) | [0.292 38](javascript:showCorrelationPlot2(db='MA_M2M_0706_R',ProbeSetID='1431972_a_at',CellID='',db2='MA_M2M_0706_R',ProbeSetID2='1447399_at',CellID2='',rank='0')) | [-0.504 38](javascript:showCorrelationPlot2(db='MA_M2M_0706_R',ProbeSetID='1431972_a_at',CellID='',db2='MA_M2M_0706_R',ProbeSetID2='1434968_a_at',CellID2='',rank='0')) | [0.381 38](javascript:showCorrelationPlot2(db='MA_M2M_0706_R',ProbeSetID='1431972_a_at',CellID='',db2='MA_M2M_0706_R',ProbeSetID2='1447335_x_at',CellID2='',rank='0')) | [0.364 38](javascript:showCorrelationPlot2(db='MA_M2M_0706_R',ProbeSetID='1431972_a_at',CellID='',db2='MA_M2M_0706_R',ProbeSetID2='1442239_at',CellID2='',rank='0')) | [0.414 38](javascript:showCorrelationPlot2(db='MA_M2M_0706_R',ProbeSetID='1431972_a_at',CellID='',db2='MA_M2M_0706_R',ProbeSetID2='1459865_x_at',CellID2='',rank='0')) | [-0.459 38](javascript:showCorrelationPlot2(db='MA_M2M_0706_R',ProbeSetID='1431972_a_at',CellID='',db2='MA_M2M_0706_R',ProbeSetID2='1438647_x_at',CellID2='',rank='0')) | [0.513 38](javascript:showCorrelationPlot2(db='MA_M2M_0706_R',ProbeSetID='1431972_a_at',CellID='',db2='MA_M2M_0706_R',ProbeSetID2='1432333_a_at',CellID2='',rank='0')) | [-0.609 38](javascript:showCorrelationPlot2(db='MA_M2M_0706_R',ProbeSetID='1431972_a_at',CellID='',db2='MA_M2M_0706_R',ProbeSetID2='1444028_s_at',CellID2='',rank='0')) | [0.607 38](javascript:showCorrelationPlot2(db='MA_M2M_0706_R',ProbeSetID='1431972_a_at',CellID='',db2='MA_M2M_0706_R',ProbeSetID2='1460420_a_at',CellID2='',rank='0')) | [0.445 38](javascript:showCorrelationPlot2(db='MA_M2M_0706_R',ProbeSetID='1431972_a_at',CellID='',db2='MA_M2M_0706_R',ProbeSetID2='1451530_at',CellID2='',rank='0')) | [-0.553 38](javascript:showCorrelationPlot2(db='MA_M2M_0706_R',ProbeSetID='1431972_a_at',CellID='',db2='MA_M2M_0706_R',ProbeSetID2='1423785_at',CellID2='',rank='0')) | [n 38](javascript:showDatabase2('MA_M2M_0706_R','1431972_a_at','')) | [-0.519 38](javascript:showCorrelationPlot2(db='MA_M2M_0706_R',ProbeSetID='1431972_a_at',CellID='',db2='MA_M2M_0706_R',ProbeSetID2='1448347_a_at',CellID2='',rank='1')) | [0.355 38](javascript:showCorrelationPlot2(db='MA_M2M_0706_R',ProbeSetID='1431972_a_at',CellID='',db2='MA_M2M_0706_R',ProbeSetID2='1418492_at',CellID2='',rank='1')) | [-0.363 38](javascript:showCorrelationPlot2(db='MA_M2M_0706_R',ProbeSetID='1431972_a_at',CellID='',db2='MA_M2M_0706_R',ProbeSetID2='1427185_at',CellID2='',rank='1')) | [-0.350 38](javascript:showCorrelationPlot2(db='MA_M2M_0706_R',ProbeSetID='1431972_a_at',CellID='',db2='MA_M2M_0706_R',ProbeSetID2='1430500_s_at',CellID2='',rank='1')) | [0.390 38](javascript:showCorrelationPlot2(db='MA_M2M_0706_R',ProbeSetID='1431972_a_at',CellID='',db2='MA_M2M_0706_R',ProbeSetID2='1447549_x_at',CellID2='',rank='1')) | [-0.444 38](javascript:showCorrelationPlot2(db='MA_M2M_0706_R',ProbeSetID='1431972_a_at',CellID='',db2='MA_M2M_0706_R',ProbeSetID2='1448943_at',CellID2='',rank='1')) | [0.328 38](javascript:showCorrelationPlot2(db='MA_M2M_0706_R',ProbeSetID='1431972_a_at',CellID='',db2='MA_M2M_0706_R',ProbeSetID2='1433162_at',CellID2='',rank='1')) | [0.475 38](javascript:showCorrelationPlot2(db='MA_M2M_0706_R',ProbeSetID='1431972_a_at',CellID='',db2='MA_M2M_0706_R',ProbeSetID2='1443494_at',CellID2='',rank='1')) | [-0.375 38](javascript:showCorrelationPlot2(db='MA_M2M_0706_R',ProbeSetID='1431972_a_at',CellID='',db2='MA_M2M_0706_R',ProbeSetID2='1417426_at',CellID2='',rank='1')) | [0.381 38](javascript:showCorrelationPlot2(db='MA_M2M_0706_R',ProbeSetID='1431972_a_at',CellID='',db2='MA_M2M_0706_R',ProbeSetID2='1416588_at',CellID2='',rank='1')) | [-0.428 38](javascript:showCorrelationPlot2(db='MA_M2M_0706_R',ProbeSetID='1431972_a_at',CellID='',db2='MA_M2M_0706_R',ProbeSetID2='1450994_at',CellID2='',rank='1')) | [0.466 38](javascript:showCorrelationPlot2(db='MA_M2M_0706_R',ProbeSetID='1431972_a_at',CellID='',db2='MA_M2M_0706_R',ProbeSetID2='1420304_x_at',CellID2='',rank='1')) | [-0.515 38](javascript:showCorrelationPlot2(db='MA_M2M_0706_R',ProbeSetID='1431972_a_at',CellID='',db2='MA_M2M_0706_R',ProbeSetID2='1416500_at',CellID2='',rank='1')) | [0.374 38](javascript:showCorrelationPlot2(db='MA_M2M_0706_R',ProbeSetID='1431972_a_at',CellID='',db2='MA_M2M_0706_R',ProbeSetID2='1424199_at',CellID2='',rank='1')) | [-0.634 38](javascript:showCorrelationPlot2(db='MA_M2M_0706_R',ProbeSetID='1431972_a_at',CellID='',db2='MA_M2M_0706_R',ProbeSetID2='1437995_x_at',CellID2='',rank='1')) | [-0.272 38](javascript:showCorrelationPlot2(db='MA_M2M_0706_R',ProbeSetID='1431972_a_at',CellID='',db2='MA_M2M_0706_R',ProbeSetID2='1427914_a_at',CellID2='',rank='1')) | [0.378 38](javascript:showCorrelationPlot2(db='MA_M2M_0706_R',ProbeSetID='1431972_a_at',CellID='',db2='MA_M2M_0706_R',ProbeSetID2='1420570_x_at',CellID2='',rank='1')) | [0.511 38](javascript:showCorrelationPlot2(db='MA_M2M_0706_R',ProbeSetID='1431972_a_at',CellID='',db2='MA_M2M_0706_R',ProbeSetID2='1431015_at',CellID2='',rank='1')) | [-0.349 38](javascript:showCorrelationPlot2(db='MA_M2M_0706_R',ProbeSetID='1431972_a_at',CellID='',db2='MA_M2M_0706_R',ProbeSetID2='1423852_at',CellID2='',rank='1')) | [0.516 38](javascript:showCorrelationPlot2(db='MA_M2M_0706_R',ProbeSetID='1431972_a_at',CellID='',db2='MA_M2M_0706_R',ProbeSetID2='1431799_at',CellID2='',rank='1')) | [-0.552 38](javascript:showCorrelationPlot2(db='MA_M2M_0706_R',ProbeSetID='1431972_a_at',CellID='',db2='MA_M2M_0706_R',ProbeSetID2='1423898_a_at',CellID2='',rank='1')) | [0.409 38](javascript:showCorrelationPlot2(db='MA_M2M_0706_R',ProbeSetID='1431972_a_at',CellID='',db2='MA_M2M_0706_R',ProbeSetID2='1438579_at',CellID2='',rank='1')) | [-0.413 38](javascript:showCorrelationPlot2(db='MA_M2M_0706_R',ProbeSetID='1431972_a_at',CellID='',db2='MA_M2M_0706_R',ProbeSetID2='1448102_a_at',CellID2='',rank='1')) | [0.390 38](javascript:showCorrelationPlot2(db='MA_M2M_0706_R',ProbeSetID='1431972_a_at',CellID='',db2='MA_M2M_0706_R',ProbeSetID2='1420943_at',CellID2='',rank='1')) | [0.419 38](javascript:showCorrelationPlot2(db='MA_M2M_0706_R',ProbeSetID='1431972_a_at',CellID='',db2='MA_M2M_0706_R',ProbeSetID2='1447326_s_at',CellID2='',rank='1')) |
|  | [Trait 26: MA_M2M_0706_R::1448347_a_at](javascript:showDatabase2('MA_M2M_0706_R','1448347_a_at','');)  Gpiap1 on Chr 2 @ 103.605228 Mb  cell cycle associated protein 1 (cytoplasmic activation- and proliferation-associated protein 1)  distal 3'UTR | [0.528 38](javascript:showCorrelationPlot2(db='MA_M2M_0706_R',ProbeSetID='1448347_a_at',CellID='',db2='MA_M2M_0706_R',ProbeSetID2='1445101_at',CellID2='',rank='0')) | [-0.669 38](javascript:showCorrelationPlot2(db='MA_M2M_0706_R',ProbeSetID='1448347_a_at',CellID='',db2='MA_M2M_0706_R',ProbeSetID2='1451285_at',CellID2='',rank='0')) | [-0.549 38](javascript:showCorrelationPlot2(db='MA_M2M_0706_R',ProbeSetID='1448347_a_at',CellID='',db2='MA_M2M_0706_R',ProbeSetID2='1441347_at',CellID2='',rank='0')) | [0.594 38](javascript:showCorrelationPlot2(db='MA_M2M_0706_R',ProbeSetID='1448347_a_at',CellID='',db2='MA_M2M_0706_R',ProbeSetID2='1426900_at',CellID2='',rank='0')) | [0.541 38](javascript:showCorrelationPlot2(db='MA_M2M_0706_R',ProbeSetID='1448347_a_at',CellID='',db2='MA_M2M_0706_R',ProbeSetID2='1434888_a_at',CellID2='',rank='0')) | [0.625 38](javascript:showCorrelationPlot2(db='MA_M2M_0706_R',ProbeSetID='1448347_a_at',CellID='',db2='MA_M2M_0706_R',ProbeSetID2='1434773_a_at',CellID2='',rank='0')) | [-0.474 38](javascript:showCorrelationPlot2(db='MA_M2M_0706_R',ProbeSetID='1448347_a_at',CellID='',db2='MA_M2M_0706_R',ProbeSetID2='1452851_at',CellID2='',rank='0')) | [0.578 38](javascript:showCorrelationPlot2(db='MA_M2M_0706_R',ProbeSetID='1448347_a_at',CellID='',db2='MA_M2M_0706_R',ProbeSetID2='1441948_x_at',CellID2='',rank='0')) | [-0.425 38](javascript:showCorrelationPlot2(db='MA_M2M_0706_R',ProbeSetID='1448347_a_at',CellID='',db2='MA_M2M_0706_R',ProbeSetID2='1440160_x_at',CellID2='',rank='0')) | [0.413 38](javascript:showCorrelationPlot2(db='MA_M2M_0706_R',ProbeSetID='1448347_a_at',CellID='',db2='MA_M2M_0706_R',ProbeSetID2='1424692_at',CellID2='',rank='0')) | [-0.577 38](javascript:showCorrelationPlot2(db='MA_M2M_0706_R',ProbeSetID='1448347_a_at',CellID='',db2='MA_M2M_0706_R',ProbeSetID2='1433112_at',CellID2='',rank='0')) | [0.614 38](javascript:showCorrelationPlot2(db='MA_M2M_0706_R',ProbeSetID='1448347_a_at',CellID='',db2='MA_M2M_0706_R',ProbeSetID2='1459962_at',CellID2='',rank='0')) | [-0.536 38](javascript:showCorrelationPlot2(db='MA_M2M_0706_R',ProbeSetID='1448347_a_at',CellID='',db2='MA_M2M_0706_R',ProbeSetID2='1455331_at',CellID2='',rank='0')) | [-0.479 38](javascript:showCorrelationPlot2(db='MA_M2M_0706_R',ProbeSetID='1448347_a_at',CellID='',db2='MA_M2M_0706_R',ProbeSetID2='1447399_at',CellID2='',rank='0')) | [0.459 38](javascript:showCorrelationPlot2(db='MA_M2M_0706_R',ProbeSetID='1448347_a_at',CellID='',db2='MA_M2M_0706_R',ProbeSetID2='1434968_a_at',CellID2='',rank='0')) | [-0.609 38](javascript:showCorrelationPlot2(db='MA_M2M_0706_R',ProbeSetID='1448347_a_at',CellID='',db2='MA_M2M_0706_R',ProbeSetID2='1447335_x_at',CellID2='',rank='0')) | [-0.499 38](javascript:showCorrelationPlot2(db='MA_M2M_0706_R',ProbeSetID='1448347_a_at',CellID='',db2='MA_M2M_0706_R',ProbeSetID2='1442239_at',CellID2='',rank='0')) | [-0.514 38](javascript:showCorrelationPlot2(db='MA_M2M_0706_R',ProbeSetID='1448347_a_at',CellID='',db2='MA_M2M_0706_R',ProbeSetID2='1459865_x_at',CellID2='',rank='0')) | [0.531 38](javascript:showCorrelationPlot2(db='MA_M2M_0706_R',ProbeSetID='1448347_a_at',CellID='',db2='MA_M2M_0706_R',ProbeSetID2='1438647_x_at',CellID2='',rank='0')) | [-0.628 38](javascript:showCorrelationPlot2(db='MA_M2M_0706_R',ProbeSetID='1448347_a_at',CellID='',db2='MA_M2M_0706_R',ProbeSetID2='1432333_a_at',CellID2='',rank='0')) | [0.652 38](javascript:showCorrelationPlot2(db='MA_M2M_0706_R',ProbeSetID='1448347_a_at',CellID='',db2='MA_M2M_0706_R',ProbeSetID2='1444028_s_at',CellID2='',rank='0')) | [-0.639 38](javascript:showCorrelationPlot2(db='MA_M2M_0706_R',ProbeSetID='1448347_a_at',CellID='',db2='MA_M2M_0706_R',ProbeSetID2='1460420_a_at',CellID2='',rank='0')) | [-0.582 38](javascript:showCorrelationPlot2(db='MA_M2M_0706_R',ProbeSetID='1448347_a_at',CellID='',db2='MA_M2M_0706_R',ProbeSetID2='1451530_at',CellID2='',rank='0')) | [0.599 38](javascript:showCorrelationPlot2(db='MA_M2M_0706_R',ProbeSetID='1448347_a_at',CellID='',db2='MA_M2M_0706_R',ProbeSetID2='1423785_at',CellID2='',rank='0')) | [-0.570 38](javascript:showCorrelationPlot2(db='MA_M2M_0706_R',ProbeSetID='1448347_a_at',CellID='',db2='MA_M2M_0706_R',ProbeSetID2='1431972_a_at',CellID2='',rank='0')) | [n 38](javascript:showDatabase2('MA_M2M_0706_R','1448347_a_at','')) | [-0.491 38](javascript:showCorrelationPlot2(db='MA_M2M_0706_R',ProbeSetID='1448347_a_at',CellID='',db2='MA_M2M_0706_R',ProbeSetID2='1418492_at',CellID2='',rank='1')) | [0.414 38](javascript:showCorrelationPlot2(db='MA_M2M_0706_R',ProbeSetID='1448347_a_at',CellID='',db2='MA_M2M_0706_R',ProbeSetID2='1427185_at',CellID2='',rank='1')) | [0.473 38](javascript:showCorrelationPlot2(db='MA_M2M_0706_R',ProbeSetID='1448347_a_at',CellID='',db2='MA_M2M_0706_R',ProbeSetID2='1430500_s_at',CellID2='',rank='1')) | [-0.535 38](javascript:showCorrelationPlot2(db='MA_M2M_0706_R',ProbeSetID='1448347_a_at',CellID='',db2='MA_M2M_0706_R',ProbeSetID2='1447549_x_at',CellID2='',rank='1')) | [0.594 38](javascript:showCorrelationPlot2(db='MA_M2M_0706_R',ProbeSetID='1448347_a_at',CellID='',db2='MA_M2M_0706_R',ProbeSetID2='1448943_at',CellID2='',rank='1')) | [-0.587 38](javascript:showCorrelationPlot2(db='MA_M2M_0706_R',ProbeSetID='1448347_a_at',CellID='',db2='MA_M2M_0706_R',ProbeSetID2='1433162_at',CellID2='',rank='1')) | [-0.471 38](javascript:showCorrelationPlot2(db='MA_M2M_0706_R',ProbeSetID='1448347_a_at',CellID='',db2='MA_M2M_0706_R',ProbeSetID2='1443494_at',CellID2='',rank='1')) | [0.478 38](javascript:showCorrelationPlot2(db='MA_M2M_0706_R',ProbeSetID='1448347_a_at',CellID='',db2='MA_M2M_0706_R',ProbeSetID2='1417426_at',CellID2='',rank='1')) | [-0.673 38](javascript:showCorrelationPlot2(db='MA_M2M_0706_R',ProbeSetID='1448347_a_at',CellID='',db2='MA_M2M_0706_R',ProbeSetID2='1416588_at',CellID2='',rank='1')) | [0.592 38](javascript:showCorrelationPlot2(db='MA_M2M_0706_R',ProbeSetID='1448347_a_at',CellID='',db2='MA_M2M_0706_R',ProbeSetID2='1450994_at',CellID2='',rank='1')) | [-0.592 38](javascript:showCorrelationPlot2(db='MA_M2M_0706_R',ProbeSetID='1448347_a_at',CellID='',db2='MA_M2M_0706_R',ProbeSetID2='1420304_x_at',CellID2='',rank='1')) | [0.664 38](javascript:showCorrelationPlot2(db='MA_M2M_0706_R',ProbeSetID='1448347_a_at',CellID='',db2='MA_M2M_0706_R',ProbeSetID2='1416500_at',CellID2='',rank='1')) | [-0.664 38](javascript:showCorrelationPlot2(db='MA_M2M_0706_R',ProbeSetID='1448347_a_at',CellID='',db2='MA_M2M_0706_R',ProbeSetID2='1424199_at',CellID2='',rank='1')) | [0.687 38](javascript:showCorrelationPlot2(db='MA_M2M_0706_R',ProbeSetID='1448347_a_at',CellID='',db2='MA_M2M_0706_R',ProbeSetID2='1437995_x_at',CellID2='',rank='1')) | [0.590 38](javascript:showCorrelationPlot2(db='MA_M2M_0706_R',ProbeSetID='1448347_a_at',CellID='',db2='MA_M2M_0706_R',ProbeSetID2='1427914_a_at',CellID2='',rank='1')) | [-0.414 38](javascript:showCorrelationPlot2(db='MA_M2M_0706_R',ProbeSetID='1448347_a_at',CellID='',db2='MA_M2M_0706_R',ProbeSetID2='1420570_x_at',CellID2='',rank='1')) | [-0.552 38](javascript:showCorrelationPlot2(db='MA_M2M_0706_R',ProbeSetID='1448347_a_at',CellID='',db2='MA_M2M_0706_R',ProbeSetID2='1431015_at',CellID2='',rank='1')) | [0.378 38](javascript:showCorrelationPlot2(db='MA_M2M_0706_R',ProbeSetID='1448347_a_at',CellID='',db2='MA_M2M_0706_R',ProbeSetID2='1423852_at',CellID2='',rank='1')) | [-0.397 38](javascript:showCorrelationPlot2(db='MA_M2M_0706_R',ProbeSetID='1448347_a_at',CellID='',db2='MA_M2M_0706_R',ProbeSetID2='1431799_at',CellID2='',rank='1')) | [0.701 38](javascript:showCorrelationPlot2(db='MA_M2M_0706_R',ProbeSetID='1448347_a_at',CellID='',db2='MA_M2M_0706_R',ProbeSetID2='1423898_a_at',CellID2='',rank='1')) | [-0.584 38](javascript:showCorrelationPlot2(db='MA_M2M_0706_R',ProbeSetID='1448347_a_at',CellID='',db2='MA_M2M_0706_R',ProbeSetID2='1438579_at',CellID2='',rank='1')) | [0.419 38](javascript:showCorrelationPlot2(db='MA_M2M_0706_R',ProbeSetID='1448347_a_at',CellID='',db2='MA_M2M_0706_R',ProbeSetID2='1448102_a_at',CellID2='',rank='1')) | [-0.348 38](javascript:showCorrelationPlot2(db='MA_M2M_0706_R',ProbeSetID='1448347_a_at',CellID='',db2='MA_M2M_0706_R',ProbeSetID2='1420943_at',CellID2='',rank='1')) | [-0.543 38](javascript:showCorrelationPlot2(db='MA_M2M_0706_R',ProbeSetID='1448347_a_at',CellID='',db2='MA_M2M_0706_R',ProbeSetID2='1447326_s_at',CellID2='',rank='1')) |
|  | [Trait 27: MA_M2M_0706_R::1418492_at](javascript:showDatabase2('MA_M2M_0706_R','1418492_at','');)  Grem2 on Chr 1 @ 176.763973 Mb  gremlin 2 homolog, cysteine knot superfamily (Xenopus laevis)  distal 3' UTR | [-0.565 38](javascript:showCorrelationPlot2(db='MA_M2M_0706_R',ProbeSetID='1418492_at',CellID='',db2='MA_M2M_0706_R',ProbeSetID2='1445101_at',CellID2='',rank='0')) | [0.448 38](javascript:showCorrelationPlot2(db='MA_M2M_0706_R',ProbeSetID='1418492_at',CellID='',db2='MA_M2M_0706_R',ProbeSetID2='1451285_at',CellID2='',rank='0')) | [0.769 38](javascript:showCorrelationPlot2(db='MA_M2M_0706_R',ProbeSetID='1418492_at',CellID='',db2='MA_M2M_0706_R',ProbeSetID2='1441347_at',CellID2='',rank='0')) | [-0.498 38](javascript:showCorrelationPlot2(db='MA_M2M_0706_R',ProbeSetID='1418492_at',CellID='',db2='MA_M2M_0706_R',ProbeSetID2='1426900_at',CellID2='',rank='0')) | [-0.451 38](javascript:showCorrelationPlot2(db='MA_M2M_0706_R',ProbeSetID='1418492_at',CellID='',db2='MA_M2M_0706_R',ProbeSetID2='1434888_a_at',CellID2='',rank='0')) | [-0.438 38](javascript:showCorrelationPlot2(db='MA_M2M_0706_R',ProbeSetID='1418492_at',CellID='',db2='MA_M2M_0706_R',ProbeSetID2='1434773_a_at',CellID2='',rank='0')) | [0.495 38](javascript:showCorrelationPlot2(db='MA_M2M_0706_R',ProbeSetID='1418492_at',CellID='',db2='MA_M2M_0706_R',ProbeSetID2='1452851_at',CellID2='',rank='0')) | [-0.484 38](javascript:showCorrelationPlot2(db='MA_M2M_0706_R',ProbeSetID='1418492_at',CellID='',db2='MA_M2M_0706_R',ProbeSetID2='1441948_x_at',CellID2='',rank='0')) | [0.664 38](javascript:showCorrelationPlot2(db='MA_M2M_0706_R',ProbeSetID='1418492_at',CellID='',db2='MA_M2M_0706_R',ProbeSetID2='1440160_x_at',CellID2='',rank='0')) | [-0.376 38](javascript:showCorrelationPlot2(db='MA_M2M_0706_R',ProbeSetID='1418492_at',CellID='',db2='MA_M2M_0706_R',ProbeSetID2='1424692_at',CellID2='',rank='0')) | [0.553 38](javascript:showCorrelationPlot2(db='MA_M2M_0706_R',ProbeSetID='1418492_at',CellID='',db2='MA_M2M_0706_R',ProbeSetID2='1433112_at',CellID2='',rank='0')) | [-0.493 38](javascript:showCorrelationPlot2(db='MA_M2M_0706_R',ProbeSetID='1418492_at',CellID='',db2='MA_M2M_0706_R',ProbeSetID2='1459962_at',CellID2='',rank='0')) | [0.567 38](javascript:showCorrelationPlot2(db='MA_M2M_0706_R',ProbeSetID='1418492_at',CellID='',db2='MA_M2M_0706_R',ProbeSetID2='1455331_at',CellID2='',rank='0')) | [0.620 38](javascript:showCorrelationPlot2(db='MA_M2M_0706_R',ProbeSetID='1418492_at',CellID='',db2='MA_M2M_0706_R',ProbeSetID2='1447399_at',CellID2='',rank='0')) | [-0.360 38](javascript:showCorrelationPlot2(db='MA_M2M_0706_R',ProbeSetID='1418492_at',CellID='',db2='MA_M2M_0706_R',ProbeSetID2='1434968_a_at',CellID2='',rank='0')) | [0.595 38](javascript:showCorrelationPlot2(db='MA_M2M_0706_R',ProbeSetID='1418492_at',CellID='',db2='MA_M2M_0706_R',ProbeSetID2='1447335_x_at',CellID2='',rank='0')) | [0.558 38](javascript:showCorrelationPlot2(db='MA_M2M_0706_R',ProbeSetID='1418492_at',CellID='',db2='MA_M2M_0706_R',ProbeSetID2='1442239_at',CellID2='',rank='0')) | [0.610 38](javascript:showCorrelationPlot2(db='MA_M2M_0706_R',ProbeSetID='1418492_at',CellID='',db2='MA_M2M_0706_R',ProbeSetID2='1459865_x_at',CellID2='',rank='0')) | [-0.615 38](javascript:showCorrelationPlot2(db='MA_M2M_0706_R',ProbeSetID='1418492_at',CellID='',db2='MA_M2M_0706_R',ProbeSetID2='1438647_x_at',CellID2='',rank='0')) | [0.399 38](javascript:showCorrelationPlot2(db='MA_M2M_0706_R',ProbeSetID='1418492_at',CellID='',db2='MA_M2M_0706_R',ProbeSetID2='1432333_a_at',CellID2='',rank='0')) | [-0.258 38](javascript:showCorrelationPlot2(db='MA_M2M_0706_R',ProbeSetID='1418492_at',CellID='',db2='MA_M2M_0706_R',ProbeSetID2='1444028_s_at',CellID2='',rank='0')) | [0.624 38](javascript:showCorrelationPlot2(db='MA_M2M_0706_R',ProbeSetID='1418492_at',CellID='',db2='MA_M2M_0706_R',ProbeSetID2='1460420_a_at',CellID2='',rank='0')) | [0.508 38](javascript:showCorrelationPlot2(db='MA_M2M_0706_R',ProbeSetID='1418492_at',CellID='',db2='MA_M2M_0706_R',ProbeSetID2='1451530_at',CellID2='',rank='0')) | [-0.460 38](javascript:showCorrelationPlot2(db='MA_M2M_0706_R',ProbeSetID='1418492_at',CellID='',db2='MA_M2M_0706_R',ProbeSetID2='1423785_at',CellID2='',rank='0')) | [0.319 38](javascript:showCorrelationPlot2(db='MA_M2M_0706_R',ProbeSetID='1418492_at',CellID='',db2='MA_M2M_0706_R',ProbeSetID2='1431972_a_at',CellID2='',rank='0')) | [-0.442 38](javascript:showCorrelationPlot2(db='MA_M2M_0706_R',ProbeSetID='1418492_at',CellID='',db2='MA_M2M_0706_R',ProbeSetID2='1448347_a_at',CellID2='',rank='0')) | [n 38](javascript:showDatabase2('MA_M2M_0706_R','1418492_at','')) | [-0.520 38](javascript:showCorrelationPlot2(db='MA_M2M_0706_R',ProbeSetID='1418492_at',CellID='',db2='MA_M2M_0706_R',ProbeSetID2='1427185_at',CellID2='',rank='1')) | [-0.659 38](javascript:showCorrelationPlot2(db='MA_M2M_0706_R',ProbeSetID='1418492_at',CellID='',db2='MA_M2M_0706_R',ProbeSetID2='1430500_s_at',CellID2='',rank='1')) | [0.631 38](javascript:showCorrelationPlot2(db='MA_M2M_0706_R',ProbeSetID='1418492_at',CellID='',db2='MA_M2M_0706_R',ProbeSetID2='1447549_x_at',CellID2='',rank='1')) | [-0.349 38](javascript:showCorrelationPlot2(db='MA_M2M_0706_R',ProbeSetID='1418492_at',CellID='',db2='MA_M2M_0706_R',ProbeSetID2='1448943_at',CellID2='',rank='1')) | [0.584 38](javascript:showCorrelationPlot2(db='MA_M2M_0706_R',ProbeSetID='1418492_at',CellID='',db2='MA_M2M_0706_R',ProbeSetID2='1433162_at',CellID2='',rank='1')) | [0.609 38](javascript:showCorrelationPlot2(db='MA_M2M_0706_R',ProbeSetID='1418492_at',CellID='',db2='MA_M2M_0706_R',ProbeSetID2='1443494_at',CellID2='',rank='1')) | [-0.344 38](javascript:showCorrelationPlot2(db='MA_M2M_0706_R',ProbeSetID='1418492_at',CellID='',db2='MA_M2M_0706_R',ProbeSetID2='1417426_at',CellID2='',rank='1')) | [0.505 38](javascript:showCorrelationPlot2(db='MA_M2M_0706_R',ProbeSetID='1418492_at',CellID='',db2='MA_M2M_0706_R',ProbeSetID2='1416588_at',CellID2='',rank='1')) | [-0.504 38](javascript:showCorrelationPlot2(db='MA_M2M_0706_R',ProbeSetID='1418492_at',CellID='',db2='MA_M2M_0706_R',ProbeSetID2='1450994_at',CellID2='',rank='1')) | [0.608 38](javascript:showCorrelationPlot2(db='MA_M2M_0706_R',ProbeSetID='1418492_at',CellID='',db2='MA_M2M_0706_R',ProbeSetID2='1420304_x_at',CellID2='',rank='1')) | [-0.522 38](javascript:showCorrelationPlot2(db='MA_M2M_0706_R',ProbeSetID='1418492_at',CellID='',db2='MA_M2M_0706_R',ProbeSetID2='1416500_at',CellID2='',rank='1')) | [0.711 38](javascript:showCorrelationPlot2(db='MA_M2M_0706_R',ProbeSetID='1418492_at',CellID='',db2='MA_M2M_0706_R',ProbeSetID2='1424199_at',CellID2='',rank='1')) | [-0.445 38](javascript:showCorrelationPlot2(db='MA_M2M_0706_R',ProbeSetID='1418492_at',CellID='',db2='MA_M2M_0706_R',ProbeSetID2='1437995_x_at',CellID2='',rank='1')) | [-0.412 38](javascript:showCorrelationPlot2(db='MA_M2M_0706_R',ProbeSetID='1418492_at',CellID='',db2='MA_M2M_0706_R',ProbeSetID2='1427914_a_at',CellID2='',rank='1')) | [0.619 38](javascript:showCorrelationPlot2(db='MA_M2M_0706_R',ProbeSetID='1418492_at',CellID='',db2='MA_M2M_0706_R',ProbeSetID2='1420570_x_at',CellID2='',rank='1')) | [0.407 38](javascript:showCorrelationPlot2(db='MA_M2M_0706_R',ProbeSetID='1418492_at',CellID='',db2='MA_M2M_0706_R',ProbeSetID2='1431015_at',CellID2='',rank='1')) | [-0.391 38](javascript:showCorrelationPlot2(db='MA_M2M_0706_R',ProbeSetID='1418492_at',CellID='',db2='MA_M2M_0706_R',ProbeSetID2='1423852_at',CellID2='',rank='1')) | [0.310 38](javascript:showCorrelationPlot2(db='MA_M2M_0706_R',ProbeSetID='1418492_at',CellID='',db2='MA_M2M_0706_R',ProbeSetID2='1431799_at',CellID2='',rank='1')) | [-0.438 38](javascript:showCorrelationPlot2(db='MA_M2M_0706_R',ProbeSetID='1418492_at',CellID='',db2='MA_M2M_0706_R',ProbeSetID2='1423898_a_at',CellID2='',rank='1')) | [0.607 38](javascript:showCorrelationPlot2(db='MA_M2M_0706_R',ProbeSetID='1418492_at',CellID='',db2='MA_M2M_0706_R',ProbeSetID2='1438579_at',CellID2='',rank='1')) | [-0.481 38](javascript:showCorrelationPlot2(db='MA_M2M_0706_R',ProbeSetID='1418492_at',CellID='',db2='MA_M2M_0706_R',ProbeSetID2='1448102_a_at',CellID2='',rank='1')) | [0.500 38](javascript:showCorrelationPlot2(db='MA_M2M_0706_R',ProbeSetID='1418492_at',CellID='',db2='MA_M2M_0706_R',ProbeSetID2='1420943_at',CellID2='',rank='1')) | [0.574 38](javascript:showCorrelationPlot2(db='MA_M2M_0706_R',ProbeSetID='1418492_at',CellID='',db2='MA_M2M_0706_R',ProbeSetID2='1447326_s_at',CellID2='',rank='1')) |
|  | [Trait 28: MA_M2M_0706_R::1427185_at](javascript:showDatabase2('MA_M2M_0706_R','1427185_at','');)  Mef2a on Chr 7 @ 74.376247 Mb  myocyte enhancer factor 2A  distal 3' UTR | [0.433 38](javascript:showCorrelationPlot2(db='MA_M2M_0706_R',ProbeSetID='1427185_at',CellID='',db2='MA_M2M_0706_R',ProbeSetID2='1445101_at',CellID2='',rank='0')) | [-0.403 38](javascript:showCorrelationPlot2(db='MA_M2M_0706_R',ProbeSetID='1427185_at',CellID='',db2='MA_M2M_0706_R',ProbeSetID2='1451285_at',CellID2='',rank='0')) | [-0.534 38](javascript:showCorrelationPlot2(db='MA_M2M_0706_R',ProbeSetID='1427185_at',CellID='',db2='MA_M2M_0706_R',ProbeSetID2='1441347_at',CellID2='',rank='0')) | [0.585 38](javascript:showCorrelationPlot2(db='MA_M2M_0706_R',ProbeSetID='1427185_at',CellID='',db2='MA_M2M_0706_R',ProbeSetID2='1426900_at',CellID2='',rank='0')) | [0.571 38](javascript:showCorrelationPlot2(db='MA_M2M_0706_R',ProbeSetID='1427185_at',CellID='',db2='MA_M2M_0706_R',ProbeSetID2='1434888_a_at',CellID2='',rank='0')) | [0.560 38](javascript:showCorrelationPlot2(db='MA_M2M_0706_R',ProbeSetID='1427185_at',CellID='',db2='MA_M2M_0706_R',ProbeSetID2='1434773_a_at',CellID2='',rank='0')) | [-0.652 38](javascript:showCorrelationPlot2(db='MA_M2M_0706_R',ProbeSetID='1427185_at',CellID='',db2='MA_M2M_0706_R',ProbeSetID2='1452851_at',CellID2='',rank='0')) | [0.527 38](javascript:showCorrelationPlot2(db='MA_M2M_0706_R',ProbeSetID='1427185_at',CellID='',db2='MA_M2M_0706_R',ProbeSetID2='1441948_x_at',CellID2='',rank='0')) | [-0.480 38](javascript:showCorrelationPlot2(db='MA_M2M_0706_R',ProbeSetID='1427185_at',CellID='',db2='MA_M2M_0706_R',ProbeSetID2='1440160_x_at',CellID2='',rank='0')) | [0.541 38](javascript:showCorrelationPlot2(db='MA_M2M_0706_R',ProbeSetID='1427185_at',CellID='',db2='MA_M2M_0706_R',ProbeSetID2='1424692_at',CellID2='',rank='0')) | [-0.422 38](javascript:showCorrelationPlot2(db='MA_M2M_0706_R',ProbeSetID='1427185_at',CellID='',db2='MA_M2M_0706_R',ProbeSetID2='1433112_at',CellID2='',rank='0')) | [0.442 38](javascript:showCorrelationPlot2(db='MA_M2M_0706_R',ProbeSetID='1427185_at',CellID='',db2='MA_M2M_0706_R',ProbeSetID2='1459962_at',CellID2='',rank='0')) | [-0.518 38](javascript:showCorrelationPlot2(db='MA_M2M_0706_R',ProbeSetID='1427185_at',CellID='',db2='MA_M2M_0706_R',ProbeSetID2='1455331_at',CellID2='',rank='0')) | [-0.466 38](javascript:showCorrelationPlot2(db='MA_M2M_0706_R',ProbeSetID='1427185_at',CellID='',db2='MA_M2M_0706_R',ProbeSetID2='1447399_at',CellID2='',rank='0')) | [0.428 38](javascript:showCorrelationPlot2(db='MA_M2M_0706_R',ProbeSetID='1427185_at',CellID='',db2='MA_M2M_0706_R',ProbeSetID2='1434968_a_at',CellID2='',rank='0')) | [-0.590 38](javascript:showCorrelationPlot2(db='MA_M2M_0706_R',ProbeSetID='1427185_at',CellID='',db2='MA_M2M_0706_R',ProbeSetID2='1447335_x_at',CellID2='',rank='0')) | [-0.432 38](javascript:showCorrelationPlot2(db='MA_M2M_0706_R',ProbeSetID='1427185_at',CellID='',db2='MA_M2M_0706_R',ProbeSetID2='1442239_at',CellID2='',rank='0')) | [-0.505 38](javascript:showCorrelationPlot2(db='MA_M2M_0706_R',ProbeSetID='1427185_at',CellID='',db2='MA_M2M_0706_R',ProbeSetID2='1459865_x_at',CellID2='',rank='0')) | [0.470 38](javascript:showCorrelationPlot2(db='MA_M2M_0706_R',ProbeSetID='1427185_at',CellID='',db2='MA_M2M_0706_R',ProbeSetID2='1438647_x_at',CellID2='',rank='0')) | [-0.646 38](javascript:showCorrelationPlot2(db='MA_M2M_0706_R',ProbeSetID='1427185_at',CellID='',db2='MA_M2M_0706_R',ProbeSetID2='1432333_a_at',CellID2='',rank='0')) | [0.542 38](javascript:showCorrelationPlot2(db='MA_M2M_0706_R',ProbeSetID='1427185_at',CellID='',db2='MA_M2M_0706_R',ProbeSetID2='1444028_s_at',CellID2='',rank='0')) | [-0.659 38](javascript:showCorrelationPlot2(db='MA_M2M_0706_R',ProbeSetID='1427185_at',CellID='',db2='MA_M2M_0706_R',ProbeSetID2='1460420_a_at',CellID2='',rank='0')) | [-0.481 38](javascript:showCorrelationPlot2(db='MA_M2M_0706_R',ProbeSetID='1427185_at',CellID='',db2='MA_M2M_0706_R',ProbeSetID2='1451530_at',CellID2='',rank='0')) | [0.554 38](javascript:showCorrelationPlot2(db='MA_M2M_0706_R',ProbeSetID='1427185_at',CellID='',db2='MA_M2M_0706_R',ProbeSetID2='1423785_at',CellID2='',rank='0')) | [-0.407 38](javascript:showCorrelationPlot2(db='MA_M2M_0706_R',ProbeSetID='1427185_at',CellID='',db2='MA_M2M_0706_R',ProbeSetID2='1431972_a_at',CellID2='',rank='0')) | [0.423 38](javascript:showCorrelationPlot2(db='MA_M2M_0706_R',ProbeSetID='1427185_at',CellID='',db2='MA_M2M_0706_R',ProbeSetID2='1448347_a_at',CellID2='',rank='0')) | [-0.498 38](javascript:showCorrelationPlot2(db='MA_M2M_0706_R',ProbeSetID='1427185_at',CellID='',db2='MA_M2M_0706_R',ProbeSetID2='1418492_at',CellID2='',rank='0')) | [n 38](javascript:showDatabase2('MA_M2M_0706_R','1427185_at','')) | [0.368 38](javascript:showCorrelationPlot2(db='MA_M2M_0706_R',ProbeSetID='1427185_at',CellID='',db2='MA_M2M_0706_R',ProbeSetID2='1430500_s_at',CellID2='',rank='1')) | [-0.471 38](javascript:showCorrelationPlot2(db='MA_M2M_0706_R',ProbeSetID='1427185_at',CellID='',db2='MA_M2M_0706_R',ProbeSetID2='1447549_x_at',CellID2='',rank='1')) | [0.556 38](javascript:showCorrelationPlot2(db='MA_M2M_0706_R',ProbeSetID='1427185_at',CellID='',db2='MA_M2M_0706_R',ProbeSetID2='1448943_at',CellID2='',rank='1')) | [-0.401 38](javascript:showCorrelationPlot2(db='MA_M2M_0706_R',ProbeSetID='1427185_at',CellID='',db2='MA_M2M_0706_R',ProbeSetID2='1433162_at',CellID2='',rank='1')) | [-0.424 38](javascript:showCorrelationPlot2(db='MA_M2M_0706_R',ProbeSetID='1427185_at',CellID='',db2='MA_M2M_0706_R',ProbeSetID2='1443494_at',CellID2='',rank='1')) | [0.574 38](javascript:showCorrelationPlot2(db='MA_M2M_0706_R',ProbeSetID='1427185_at',CellID='',db2='MA_M2M_0706_R',ProbeSetID2='1417426_at',CellID2='',rank='1')) | [-0.526 38](javascript:showCorrelationPlot2(db='MA_M2M_0706_R',ProbeSetID='1427185_at',CellID='',db2='MA_M2M_0706_R',ProbeSetID2='1416588_at',CellID2='',rank='1')) | [0.448 38](javascript:showCorrelationPlot2(db='MA_M2M_0706_R',ProbeSetID='1427185_at',CellID='',db2='MA_M2M_0706_R',ProbeSetID2='1450994_at',CellID2='',rank='1')) | [-0.475 38](javascript:showCorrelationPlot2(db='MA_M2M_0706_R',ProbeSetID='1427185_at',CellID='',db2='MA_M2M_0706_R',ProbeSetID2='1420304_x_at',CellID2='',rank='1')) | [0.569 38](javascript:showCorrelationPlot2(db='MA_M2M_0706_R',ProbeSetID='1427185_at',CellID='',db2='MA_M2M_0706_R',ProbeSetID2='1416500_at',CellID2='',rank='1')) | [-0.504 38](javascript:showCorrelationPlot2(db='MA_M2M_0706_R',ProbeSetID='1427185_at',CellID='',db2='MA_M2M_0706_R',ProbeSetID2='1424199_at',CellID2='',rank='1')) | [0.605 38](javascript:showCorrelationPlot2(db='MA_M2M_0706_R',ProbeSetID='1427185_at',CellID='',db2='MA_M2M_0706_R',ProbeSetID2='1437995_x_at',CellID2='',rank='1')) | [0.271 38](javascript:showCorrelationPlot2(db='MA_M2M_0706_R',ProbeSetID='1427185_at',CellID='',db2='MA_M2M_0706_R',ProbeSetID2='1427914_a_at',CellID2='',rank='1')) | [-0.558 38](javascript:showCorrelationPlot2(db='MA_M2M_0706_R',ProbeSetID='1427185_at',CellID='',db2='MA_M2M_0706_R',ProbeSetID2='1420570_x_at',CellID2='',rank='1')) | [-0.492 38](javascript:showCorrelationPlot2(db='MA_M2M_0706_R',ProbeSetID='1427185_at',CellID='',db2='MA_M2M_0706_R',ProbeSetID2='1431015_at',CellID2='',rank='1')) | [0.449 38](javascript:showCorrelationPlot2(db='MA_M2M_0706_R',ProbeSetID='1427185_at',CellID='',db2='MA_M2M_0706_R',ProbeSetID2='1423852_at',CellID2='',rank='1')) | [-0.274 38](javascript:showCorrelationPlot2(db='MA_M2M_0706_R',ProbeSetID='1427185_at',CellID='',db2='MA_M2M_0706_R',ProbeSetID2='1431799_at',CellID2='',rank='1')) | [0.394 38](javascript:showCorrelationPlot2(db='MA_M2M_0706_R',ProbeSetID='1427185_at',CellID='',db2='MA_M2M_0706_R',ProbeSetID2='1423898_a_at',CellID2='',rank='1')) | [-0.527 38](javascript:showCorrelationPlot2(db='MA_M2M_0706_R',ProbeSetID='1427185_at',CellID='',db2='MA_M2M_0706_R',ProbeSetID2='1438579_at',CellID2='',rank='1')) | [0.500 38](javascript:showCorrelationPlot2(db='MA_M2M_0706_R',ProbeSetID='1427185_at',CellID='',db2='MA_M2M_0706_R',ProbeSetID2='1448102_a_at',CellID2='',rank='1')) | [-0.543 38](javascript:showCorrelationPlot2(db='MA_M2M_0706_R',ProbeSetID='1427185_at',CellID='',db2='MA_M2M_0706_R',ProbeSetID2='1420943_at',CellID2='',rank='1')) | [-0.372 38](javascript:showCorrelationPlot2(db='MA_M2M_0706_R',ProbeSetID='1427185_at',CellID='',db2='MA_M2M_0706_R',ProbeSetID2='1447326_s_at',CellID2='',rank='1')) |
|  | [Trait 29: MA_M2M_0706_R::1430500_s_at](javascript:showDatabase2('MA_M2M_0706_R','1430500_s_at','');)  Mtx2 on Chr 2 @ 74.707405 Mb  metaxin 2 | [0.518 38](javascript:showCorrelationPlot2(db='MA_M2M_0706_R',ProbeSetID='1430500_s_at',CellID='',db2='MA_M2M_0706_R',ProbeSetID2='1445101_at',CellID2='',rank='0')) | [-0.430 38](javascript:showCorrelationPlot2(db='MA_M2M_0706_R',ProbeSetID='1430500_s_at',CellID='',db2='MA_M2M_0706_R',ProbeSetID2='1451285_at',CellID2='',rank='0')) | [-0.521 38](javascript:showCorrelationPlot2(db='MA_M2M_0706_R',ProbeSetID='1430500_s_at',CellID='',db2='MA_M2M_0706_R',ProbeSetID2='1441347_at',CellID2='',rank='0')) | [0.482 38](javascript:showCorrelationPlot2(db='MA_M2M_0706_R',ProbeSetID='1430500_s_at',CellID='',db2='MA_M2M_0706_R',ProbeSetID2='1426900_at',CellID2='',rank='0')) | [0.587 38](javascript:showCorrelationPlot2(db='MA_M2M_0706_R',ProbeSetID='1430500_s_at',CellID='',db2='MA_M2M_0706_R',ProbeSetID2='1434888_a_at',CellID2='',rank='0')) | [0.643 38](javascript:showCorrelationPlot2(db='MA_M2M_0706_R',ProbeSetID='1430500_s_at',CellID='',db2='MA_M2M_0706_R',ProbeSetID2='1434773_a_at',CellID2='',rank='0')) | [-0.423 38](javascript:showCorrelationPlot2(db='MA_M2M_0706_R',ProbeSetID='1430500_s_at',CellID='',db2='MA_M2M_0706_R',ProbeSetID2='1452851_at',CellID2='',rank='0')) | [0.516 38](javascript:showCorrelationPlot2(db='MA_M2M_0706_R',ProbeSetID='1430500_s_at',CellID='',db2='MA_M2M_0706_R',ProbeSetID2='1441948_x_at',CellID2='',rank='0')) | [-0.543 38](javascript:showCorrelationPlot2(db='MA_M2M_0706_R',ProbeSetID='1430500_s_at',CellID='',db2='MA_M2M_0706_R',ProbeSetID2='1440160_x_at',CellID2='',rank='0')) | [0.512 38](javascript:showCorrelationPlot2(db='MA_M2M_0706_R',ProbeSetID='1430500_s_at',CellID='',db2='MA_M2M_0706_R',ProbeSetID2='1424692_at',CellID2='',rank='0')) | [-0.457 38](javascript:showCorrelationPlot2(db='MA_M2M_0706_R',ProbeSetID='1430500_s_at',CellID='',db2='MA_M2M_0706_R',ProbeSetID2='1433112_at',CellID2='',rank='0')) | [0.325 38](javascript:showCorrelationPlot2(db='MA_M2M_0706_R',ProbeSetID='1430500_s_at',CellID='',db2='MA_M2M_0706_R',ProbeSetID2='1459962_at',CellID2='',rank='0')) | [-0.341 38](javascript:showCorrelationPlot2(db='MA_M2M_0706_R',ProbeSetID='1430500_s_at',CellID='',db2='MA_M2M_0706_R',ProbeSetID2='1455331_at',CellID2='',rank='0')) | [-0.409 38](javascript:showCorrelationPlot2(db='MA_M2M_0706_R',ProbeSetID='1430500_s_at',CellID='',db2='MA_M2M_0706_R',ProbeSetID2='1447399_at',CellID2='',rank='0')) | [0.453 38](javascript:showCorrelationPlot2(db='MA_M2M_0706_R',ProbeSetID='1430500_s_at',CellID='',db2='MA_M2M_0706_R',ProbeSetID2='1434968_a_at',CellID2='',rank='0')) | [-0.580 38](javascript:showCorrelationPlot2(db='MA_M2M_0706_R',ProbeSetID='1430500_s_at',CellID='',db2='MA_M2M_0706_R',ProbeSetID2='1447335_x_at',CellID2='',rank='0')) | [-0.519 38](javascript:showCorrelationPlot2(db='MA_M2M_0706_R',ProbeSetID='1430500_s_at',CellID='',db2='MA_M2M_0706_R',ProbeSetID2='1442239_at',CellID2='',rank='0')) | [-0.645 38](javascript:showCorrelationPlot2(db='MA_M2M_0706_R',ProbeSetID='1430500_s_at',CellID='',db2='MA_M2M_0706_R',ProbeSetID2='1459865_x_at',CellID2='',rank='0')) | [0.677 38](javascript:showCorrelationPlot2(db='MA_M2M_0706_R',ProbeSetID='1430500_s_at',CellID='',db2='MA_M2M_0706_R',ProbeSetID2='1438647_x_at',CellID2='',rank='0')) | [-0.367 38](javascript:showCorrelationPlot2(db='MA_M2M_0706_R',ProbeSetID='1430500_s_at',CellID='',db2='MA_M2M_0706_R',ProbeSetID2='1432333_a_at',CellID2='',rank='0')) | [0.293 38](javascript:showCorrelationPlot2(db='MA_M2M_0706_R',ProbeSetID='1430500_s_at',CellID='',db2='MA_M2M_0706_R',ProbeSetID2='1444028_s_at',CellID2='',rank='0')) | [-0.619 38](javascript:showCorrelationPlot2(db='MA_M2M_0706_R',ProbeSetID='1430500_s_at',CellID='',db2='MA_M2M_0706_R',ProbeSetID2='1460420_a_at',CellID2='',rank='0')) | [-0.482 38](javascript:showCorrelationPlot2(db='MA_M2M_0706_R',ProbeSetID='1430500_s_at',CellID='',db2='MA_M2M_0706_R',ProbeSetID2='1451530_at',CellID2='',rank='0')) | [0.370 38](javascript:showCorrelationPlot2(db='MA_M2M_0706_R',ProbeSetID='1430500_s_at',CellID='',db2='MA_M2M_0706_R',ProbeSetID2='1423785_at',CellID2='',rank='0')) | [-0.354 38](javascript:showCorrelationPlot2(db='MA_M2M_0706_R',ProbeSetID='1430500_s_at',CellID='',db2='MA_M2M_0706_R',ProbeSetID2='1431972_a_at',CellID2='',rank='0')) | [0.517 38](javascript:showCorrelationPlot2(db='MA_M2M_0706_R',ProbeSetID='1430500_s_at',CellID='',db2='MA_M2M_0706_R',ProbeSetID2='1448347_a_at',CellID2='',rank='0')) | [-0.663 38](javascript:showCorrelationPlot2(db='MA_M2M_0706_R',ProbeSetID='1430500_s_at',CellID='',db2='MA_M2M_0706_R',ProbeSetID2='1418492_at',CellID2='',rank='0')) | [0.368 38](javascript:showCorrelationPlot2(db='MA_M2M_0706_R',ProbeSetID='1430500_s_at',CellID='',db2='MA_M2M_0706_R',ProbeSetID2='1427185_at',CellID2='',rank='0')) | [n 38](javascript:showDatabase2('MA_M2M_0706_R','1430500_s_at','')) | [-0.622 38](javascript:showCorrelationPlot2(db='MA_M2M_0706_R',ProbeSetID='1430500_s_at',CellID='',db2='MA_M2M_0706_R',ProbeSetID2='1447549_x_at',CellID2='',rank='1')) | [0.317 38](javascript:showCorrelationPlot2(db='MA_M2M_0706_R',ProbeSetID='1430500_s_at',CellID='',db2='MA_M2M_0706_R',ProbeSetID2='1448943_at',CellID2='',rank='1')) | [-0.510 38](javascript:showCorrelationPlot2(db='MA_M2M_0706_R',ProbeSetID='1430500_s_at',CellID='',db2='MA_M2M_0706_R',ProbeSetID2='1433162_at',CellID2='',rank='1')) | [-0.598 38](javascript:showCorrelationPlot2(db='MA_M2M_0706_R',ProbeSetID='1430500_s_at',CellID='',db2='MA_M2M_0706_R',ProbeSetID2='1443494_at',CellID2='',rank='1')) | [0.306 38](javascript:showCorrelationPlot2(db='MA_M2M_0706_R',ProbeSetID='1430500_s_at',CellID='',db2='MA_M2M_0706_R',ProbeSetID2='1417426_at',CellID2='',rank='1')) | [-0.586 38](javascript:showCorrelationPlot2(db='MA_M2M_0706_R',ProbeSetID='1430500_s_at',CellID='',db2='MA_M2M_0706_R',ProbeSetID2='1416588_at',CellID2='',rank='1')) | [0.530 38](javascript:showCorrelationPlot2(db='MA_M2M_0706_R',ProbeSetID='1430500_s_at',CellID='',db2='MA_M2M_0706_R',ProbeSetID2='1450994_at',CellID2='',rank='1')) | [-0.690 38](javascript:showCorrelationPlot2(db='MA_M2M_0706_R',ProbeSetID='1430500_s_at',CellID='',db2='MA_M2M_0706_R',ProbeSetID2='1420304_x_at',CellID2='',rank='1')) | [0.582 38](javascript:showCorrelationPlot2(db='MA_M2M_0706_R',ProbeSetID='1430500_s_at',CellID='',db2='MA_M2M_0706_R',ProbeSetID2='1416500_at',CellID2='',rank='1')) | [-0.530 38](javascript:showCorrelationPlot2(db='MA_M2M_0706_R',ProbeSetID='1430500_s_at',CellID='',db2='MA_M2M_0706_R',ProbeSetID2='1424199_at',CellID2='',rank='1')) | [0.567 38](javascript:showCorrelationPlot2(db='MA_M2M_0706_R',ProbeSetID='1430500_s_at',CellID='',db2='MA_M2M_0706_R',ProbeSetID2='1437995_x_at',CellID2='',rank='1')) | [0.617 38](javascript:showCorrelationPlot2(db='MA_M2M_0706_R',ProbeSetID='1430500_s_at',CellID='',db2='MA_M2M_0706_R',ProbeSetID2='1427914_a_at',CellID2='',rank='1')) | [-0.567 38](javascript:showCorrelationPlot2(db='MA_M2M_0706_R',ProbeSetID='1430500_s_at',CellID='',db2='MA_M2M_0706_R',ProbeSetID2='1420570_x_at',CellID2='',rank='1')) | [-0.519 38](javascript:showCorrelationPlot2(db='MA_M2M_0706_R',ProbeSetID='1430500_s_at',CellID='',db2='MA_M2M_0706_R',ProbeSetID2='1431015_at',CellID2='',rank='1')) | [0.411 38](javascript:showCorrelationPlot2(db='MA_M2M_0706_R',ProbeSetID='1430500_s_at',CellID='',db2='MA_M2M_0706_R',ProbeSetID2='1423852_at',CellID2='',rank='1')) | [-0.439 38](javascript:showCorrelationPlot2(db='MA_M2M_0706_R',ProbeSetID='1430500_s_at',CellID='',db2='MA_M2M_0706_R',ProbeSetID2='1431799_at',CellID2='',rank='1')) | [0.554 38](javascript:showCorrelationPlot2(db='MA_M2M_0706_R',ProbeSetID='1430500_s_at',CellID='',db2='MA_M2M_0706_R',ProbeSetID2='1423898_a_at',CellID2='',rank='1')) | [-0.520 38](javascript:showCorrelationPlot2(db='MA_M2M_0706_R',ProbeSetID='1430500_s_at',CellID='',db2='MA_M2M_0706_R',ProbeSetID2='1438579_at',CellID2='',rank='1')) | [0.629 38](javascript:showCorrelationPlot2(db='MA_M2M_0706_R',ProbeSetID='1430500_s_at',CellID='',db2='MA_M2M_0706_R',ProbeSetID2='1448102_a_at',CellID2='',rank='1')) | [-0.563 38](javascript:showCorrelationPlot2(db='MA_M2M_0706_R',ProbeSetID='1430500_s_at',CellID='',db2='MA_M2M_0706_R',ProbeSetID2='1420943_at',CellID2='',rank='1')) | [-0.704 38](javascript:showCorrelationPlot2(db='MA_M2M_0706_R',ProbeSetID='1430500_s_at',CellID='',db2='MA_M2M_0706_R',ProbeSetID2='1447326_s_at',CellID2='',rank='1')) |
|  | [Trait 30: MA_M2M_0706_R::1447549_x_at](javascript:showDatabase2('MA_M2M_0706_R','1447549_x_at','');)  Ninj1 on Chr 13 @ 49.291335 Mb  ninjurin 1  mid 3' UTR | [-0.412 38](javascript:showCorrelationPlot2(db='MA_M2M_0706_R',ProbeSetID='1447549_x_at',CellID='',db2='MA_M2M_0706_R',ProbeSetID2='1445101_at',CellID2='',rank='0')) | [0.416 38](javascript:showCorrelationPlot2(db='MA_M2M_0706_R',ProbeSetID='1447549_x_at',CellID='',db2='MA_M2M_0706_R',ProbeSetID2='1451285_at',CellID2='',rank='0')) | [0.641 38](javascript:showCorrelationPlot2(db='MA_M2M_0706_R',ProbeSetID='1447549_x_at',CellID='',db2='MA_M2M_0706_R',ProbeSetID2='1441347_at',CellID2='',rank='0')) | [-0.444 38](javascript:showCorrelationPlot2(db='MA_M2M_0706_R',ProbeSetID='1447549_x_at',CellID='',db2='MA_M2M_0706_R',ProbeSetID2='1426900_at',CellID2='',rank='0')) | [-0.394 38](javascript:showCorrelationPlot2(db='MA_M2M_0706_R',ProbeSetID='1447549_x_at',CellID='',db2='MA_M2M_0706_R',ProbeSetID2='1434888_a_at',CellID2='',rank='0')) | [-0.480 38](javascript:showCorrelationPlot2(db='MA_M2M_0706_R',ProbeSetID='1447549_x_at',CellID='',db2='MA_M2M_0706_R',ProbeSetID2='1434773_a_at',CellID2='',rank='0')) | [0.567 38](javascript:showCorrelationPlot2(db='MA_M2M_0706_R',ProbeSetID='1447549_x_at',CellID='',db2='MA_M2M_0706_R',ProbeSetID2='1452851_at',CellID2='',rank='0')) | [-0.367 38](javascript:showCorrelationPlot2(db='MA_M2M_0706_R',ProbeSetID='1447549_x_at',CellID='',db2='MA_M2M_0706_R',ProbeSetID2='1441948_x_at',CellID2='',rank='0')) | [0.554 38](javascript:showCorrelationPlot2(db='MA_M2M_0706_R',ProbeSetID='1447549_x_at',CellID='',db2='MA_M2M_0706_R',ProbeSetID2='1440160_x_at',CellID2='',rank='0')) | [-0.254 38](javascript:showCorrelationPlot2(db='MA_M2M_0706_R',ProbeSetID='1447549_x_at',CellID='',db2='MA_M2M_0706_R',ProbeSetID2='1424692_at',CellID2='',rank='0')) | [0.536 38](javascript:showCorrelationPlot2(db='MA_M2M_0706_R',ProbeSetID='1447549_x_at',CellID='',db2='MA_M2M_0706_R',ProbeSetID2='1433112_at',CellID2='',rank='0')) | [-0.448 38](javascript:showCorrelationPlot2(db='MA_M2M_0706_R',ProbeSetID='1447549_x_at',CellID='',db2='MA_M2M_0706_R',ProbeSetID2='1459962_at',CellID2='',rank='0')) | [0.645 38](javascript:showCorrelationPlot2(db='MA_M2M_0706_R',ProbeSetID='1447549_x_at',CellID='',db2='MA_M2M_0706_R',ProbeSetID2='1455331_at',CellID2='',rank='0')) | [0.492 38](javascript:showCorrelationPlot2(db='MA_M2M_0706_R',ProbeSetID='1447549_x_at',CellID='',db2='MA_M2M_0706_R',ProbeSetID2='1447399_at',CellID2='',rank='0')) | [-0.267 38](javascript:showCorrelationPlot2(db='MA_M2M_0706_R',ProbeSetID='1447549_x_at',CellID='',db2='MA_M2M_0706_R',ProbeSetID2='1434968_a_at',CellID2='',rank='0')) | [0.742 38](javascript:showCorrelationPlot2(db='MA_M2M_0706_R',ProbeSetID='1447549_x_at',CellID='',db2='MA_M2M_0706_R',ProbeSetID2='1447335_x_at',CellID2='',rank='0')) | [0.584 38](javascript:showCorrelationPlot2(db='MA_M2M_0706_R',ProbeSetID='1447549_x_at',CellID='',db2='MA_M2M_0706_R',ProbeSetID2='1442239_at',CellID2='',rank='0')) | [0.630 38](javascript:showCorrelationPlot2(db='MA_M2M_0706_R',ProbeSetID='1447549_x_at',CellID='',db2='MA_M2M_0706_R',ProbeSetID2='1459865_x_at',CellID2='',rank='0')) | [-0.416 38](javascript:showCorrelationPlot2(db='MA_M2M_0706_R',ProbeSetID='1447549_x_at',CellID='',db2='MA_M2M_0706_R',ProbeSetID2='1438647_x_at',CellID2='',rank='0')) | [0.456 38](javascript:showCorrelationPlot2(db='MA_M2M_0706_R',ProbeSetID='1447549_x_at',CellID='',db2='MA_M2M_0706_R',ProbeSetID2='1432333_a_at',CellID2='',rank='0')) | [-0.459 38](javascript:showCorrelationPlot2(db='MA_M2M_0706_R',ProbeSetID='1447549_x_at',CellID='',db2='MA_M2M_0706_R',ProbeSetID2='1444028_s_at',CellID2='',rank='0')) | [0.598 38](javascript:showCorrelationPlot2(db='MA_M2M_0706_R',ProbeSetID='1447549_x_at',CellID='',db2='MA_M2M_0706_R',ProbeSetID2='1460420_a_at',CellID2='',rank='0')) | [0.586 38](javascript:showCorrelationPlot2(db='MA_M2M_0706_R',ProbeSetID='1447549_x_at',CellID='',db2='MA_M2M_0706_R',ProbeSetID2='1451530_at',CellID2='',rank='0')) | [-0.460 38](javascript:showCorrelationPlot2(db='MA_M2M_0706_R',ProbeSetID='1447549_x_at',CellID='',db2='MA_M2M_0706_R',ProbeSetID2='1423785_at',CellID2='',rank='0')) | [0.405 38](javascript:showCorrelationPlot2(db='MA_M2M_0706_R',ProbeSetID='1447549_x_at',CellID='',db2='MA_M2M_0706_R',ProbeSetID2='1431972_a_at',CellID2='',rank='0')) | [-0.447 38](javascript:showCorrelationPlot2(db='MA_M2M_0706_R',ProbeSetID='1447549_x_at',CellID='',db2='MA_M2M_0706_R',ProbeSetID2='1448347_a_at',CellID2='',rank='0')) | [0.639 38](javascript:showCorrelationPlot2(db='MA_M2M_0706_R',ProbeSetID='1447549_x_at',CellID='',db2='MA_M2M_0706_R',ProbeSetID2='1418492_at',CellID2='',rank='0')) | [-0.516 38](javascript:showCorrelationPlot2(db='MA_M2M_0706_R',ProbeSetID='1447549_x_at',CellID='',db2='MA_M2M_0706_R',ProbeSetID2='1427185_at',CellID2='',rank='0')) | [-0.589 38](javascript:showCorrelationPlot2(db='MA_M2M_0706_R',ProbeSetID='1447549_x_at',CellID='',db2='MA_M2M_0706_R',ProbeSetID2='1430500_s_at',CellID2='',rank='0')) | [n 38](javascript:showDatabase2('MA_M2M_0706_R','1447549_x_at','')) | [-0.287 38](javascript:showCorrelationPlot2(db='MA_M2M_0706_R',ProbeSetID='1447549_x_at',CellID='',db2='MA_M2M_0706_R',ProbeSetID2='1448943_at',CellID2='',rank='1')) | [0.501 38](javascript:showCorrelationPlot2(db='MA_M2M_0706_R',ProbeSetID='1447549_x_at',CellID='',db2='MA_M2M_0706_R',ProbeSetID2='1433162_at',CellID2='',rank='1')) | [0.537 38](javascript:showCorrelationPlot2(db='MA_M2M_0706_R',ProbeSetID='1447549_x_at',CellID='',db2='MA_M2M_0706_R',ProbeSetID2='1443494_at',CellID2='',rank='1')) | [-0.351 38](javascript:showCorrelationPlot2(db='MA_M2M_0706_R',ProbeSetID='1447549_x_at',CellID='',db2='MA_M2M_0706_R',ProbeSetID2='1417426_at',CellID2='',rank='1')) | [0.522 38](javascript:showCorrelationPlot2(db='MA_M2M_0706_R',ProbeSetID='1447549_x_at',CellID='',db2='MA_M2M_0706_R',ProbeSetID2='1416588_at',CellID2='',rank='1')) | [-0.528 38](javascript:showCorrelationPlot2(db='MA_M2M_0706_R',ProbeSetID='1447549_x_at',CellID='',db2='MA_M2M_0706_R',ProbeSetID2='1450994_at',CellID2='',rank='1')) | [0.662 38](javascript:showCorrelationPlot2(db='MA_M2M_0706_R',ProbeSetID='1447549_x_at',CellID='',db2='MA_M2M_0706_R',ProbeSetID2='1420304_x_at',CellID2='',rank='1')) | [-0.690 38](javascript:showCorrelationPlot2(db='MA_M2M_0706_R',ProbeSetID='1447549_x_at',CellID='',db2='MA_M2M_0706_R',ProbeSetID2='1416500_at',CellID2='',rank='1')) | [0.552 38](javascript:showCorrelationPlot2(db='MA_M2M_0706_R',ProbeSetID='1447549_x_at',CellID='',db2='MA_M2M_0706_R',ProbeSetID2='1424199_at',CellID2='',rank='1')) | [-0.470 38](javascript:showCorrelationPlot2(db='MA_M2M_0706_R',ProbeSetID='1447549_x_at',CellID='',db2='MA_M2M_0706_R',ProbeSetID2='1437995_x_at',CellID2='',rank='1')) | [-0.457 38](javascript:showCorrelationPlot2(db='MA_M2M_0706_R',ProbeSetID='1447549_x_at',CellID='',db2='MA_M2M_0706_R',ProbeSetID2='1427914_a_at',CellID2='',rank='1')) | [0.683 38](javascript:showCorrelationPlot2(db='MA_M2M_0706_R',ProbeSetID='1447549_x_at',CellID='',db2='MA_M2M_0706_R',ProbeSetID2='1420570_x_at',CellID2='',rank='1')) | [0.531 38](javascript:showCorrelationPlot2(db='MA_M2M_0706_R',ProbeSetID='1447549_x_at',CellID='',db2='MA_M2M_0706_R',ProbeSetID2='1431015_at',CellID2='',rank='1')) | [-0.428 38](javascript:showCorrelationPlot2(db='MA_M2M_0706_R',ProbeSetID='1447549_x_at',CellID='',db2='MA_M2M_0706_R',ProbeSetID2='1423852_at',CellID2='',rank='1')) | [0.277 38](javascript:showCorrelationPlot2(db='MA_M2M_0706_R',ProbeSetID='1447549_x_at',CellID='',db2='MA_M2M_0706_R',ProbeSetID2='1431799_at',CellID2='',rank='1')) | [-0.325 38](javascript:showCorrelationPlot2(db='MA_M2M_0706_R',ProbeSetID='1447549_x_at',CellID='',db2='MA_M2M_0706_R',ProbeSetID2='1423898_a_at',CellID2='',rank='1')) | [0.527 38](javascript:showCorrelationPlot2(db='MA_M2M_0706_R',ProbeSetID='1447549_x_at',CellID='',db2='MA_M2M_0706_R',ProbeSetID2='1438579_at',CellID2='',rank='1')) | [-0.484 38](javascript:showCorrelationPlot2(db='MA_M2M_0706_R',ProbeSetID='1447549_x_at',CellID='',db2='MA_M2M_0706_R',ProbeSetID2='1448102_a_at',CellID2='',rank='1')) | [0.466 38](javascript:showCorrelationPlot2(db='MA_M2M_0706_R',ProbeSetID='1447549_x_at',CellID='',db2='MA_M2M_0706_R',ProbeSetID2='1420943_at',CellID2='',rank='1')) | [0.661 38](javascript:showCorrelationPlot2(db='MA_M2M_0706_R',ProbeSetID='1447549_x_at',CellID='',db2='MA_M2M_0706_R',ProbeSetID2='1447326_s_at',CellID2='',rank='1')) |
|  | [Trait 31: MA_M2M_0706_R::1448943_at](javascript:showDatabase2('MA_M2M_0706_R','1448943_at','');)  Nrp1 on Chr 8 @ 131.028801 Mb  neuropilin 1  distal 3' UTR | [0.409 38](javascript:showCorrelationPlot2(db='MA_M2M_0706_R',ProbeSetID='1448943_at',CellID='',db2='MA_M2M_0706_R',ProbeSetID2='1445101_at',CellID2='',rank='0')) | [-0.261 38](javascript:showCorrelationPlot2(db='MA_M2M_0706_R',ProbeSetID='1448943_at',CellID='',db2='MA_M2M_0706_R',ProbeSetID2='1451285_at',CellID2='',rank='0')) | [-0.266 38](javascript:showCorrelationPlot2(db='MA_M2M_0706_R',ProbeSetID='1448943_at',CellID='',db2='MA_M2M_0706_R',ProbeSetID2='1441347_at',CellID2='',rank='0')) | [0.618 38](javascript:showCorrelationPlot2(db='MA_M2M_0706_R',ProbeSetID='1448943_at',CellID='',db2='MA_M2M_0706_R',ProbeSetID2='1426900_at',CellID2='',rank='0')) | [0.443 38](javascript:showCorrelationPlot2(db='MA_M2M_0706_R',ProbeSetID='1448943_at',CellID='',db2='MA_M2M_0706_R',ProbeSetID2='1434888_a_at',CellID2='',rank='0')) | [0.531 38](javascript:showCorrelationPlot2(db='MA_M2M_0706_R',ProbeSetID='1448943_at',CellID='',db2='MA_M2M_0706_R',ProbeSetID2='1434773_a_at',CellID2='',rank='0')) | [-0.577 38](javascript:showCorrelationPlot2(db='MA_M2M_0706_R',ProbeSetID='1448943_at',CellID='',db2='MA_M2M_0706_R',ProbeSetID2='1452851_at',CellID2='',rank='0')) | [0.318 38](javascript:showCorrelationPlot2(db='MA_M2M_0706_R',ProbeSetID='1448943_at',CellID='',db2='MA_M2M_0706_R',ProbeSetID2='1441948_x_at',CellID2='',rank='0')) | [-0.438 38](javascript:showCorrelationPlot2(db='MA_M2M_0706_R',ProbeSetID='1448943_at',CellID='',db2='MA_M2M_0706_R',ProbeSetID2='1440160_x_at',CellID2='',rank='0')) | [0.501 38](javascript:showCorrelationPlot2(db='MA_M2M_0706_R',ProbeSetID='1448943_at',CellID='',db2='MA_M2M_0706_R',ProbeSetID2='1424692_at',CellID2='',rank='0')) | [-0.483 38](javascript:showCorrelationPlot2(db='MA_M2M_0706_R',ProbeSetID='1448943_at',CellID='',db2='MA_M2M_0706_R',ProbeSetID2='1433112_at',CellID2='',rank='0')) | [0.368 38](javascript:showCorrelationPlot2(db='MA_M2M_0706_R',ProbeSetID='1448943_at',CellID='',db2='MA_M2M_0706_R',ProbeSetID2='1459962_at',CellID2='',rank='0')) | [-0.395 38](javascript:showCorrelationPlot2(db='MA_M2M_0706_R',ProbeSetID='1448943_at',CellID='',db2='MA_M2M_0706_R',ProbeSetID2='1455331_at',CellID2='',rank='0')) | [-0.416 38](javascript:showCorrelationPlot2(db='MA_M2M_0706_R',ProbeSetID='1448943_at',CellID='',db2='MA_M2M_0706_R',ProbeSetID2='1447399_at',CellID2='',rank='0')) | [0.512 38](javascript:showCorrelationPlot2(db='MA_M2M_0706_R',ProbeSetID='1448943_at',CellID='',db2='MA_M2M_0706_R',ProbeSetID2='1434968_a_at',CellID2='',rank='0')) | [-0.585 38](javascript:showCorrelationPlot2(db='MA_M2M_0706_R',ProbeSetID='1448943_at',CellID='',db2='MA_M2M_0706_R',ProbeSetID2='1447335_x_at',CellID2='',rank='0')) | [-0.403 38](javascript:showCorrelationPlot2(db='MA_M2M_0706_R',ProbeSetID='1448943_at',CellID='',db2='MA_M2M_0706_R',ProbeSetID2='1442239_at',CellID2='',rank='0')) | [-0.554 38](javascript:showCorrelationPlot2(db='MA_M2M_0706_R',ProbeSetID='1448943_at',CellID='',db2='MA_M2M_0706_R',ProbeSetID2='1459865_x_at',CellID2='',rank='0')) | [0.303 38](javascript:showCorrelationPlot2(db='MA_M2M_0706_R',ProbeSetID='1448943_at',CellID='',db2='MA_M2M_0706_R',ProbeSetID2='1438647_x_at',CellID2='',rank='0')) | [-0.548 38](javascript:showCorrelationPlot2(db='MA_M2M_0706_R',ProbeSetID='1448943_at',CellID='',db2='MA_M2M_0706_R',ProbeSetID2='1432333_a_at',CellID2='',rank='0')) | [0.687 38](javascript:showCorrelationPlot2(db='MA_M2M_0706_R',ProbeSetID='1448943_at',CellID='',db2='MA_M2M_0706_R',ProbeSetID2='1444028_s_at',CellID2='',rank='0')) | [-0.641 38](javascript:showCorrelationPlot2(db='MA_M2M_0706_R',ProbeSetID='1448943_at',CellID='',db2='MA_M2M_0706_R',ProbeSetID2='1460420_a_at',CellID2='',rank='0')) | [-0.452 38](javascript:showCorrelationPlot2(db='MA_M2M_0706_R',ProbeSetID='1448943_at',CellID='',db2='MA_M2M_0706_R',ProbeSetID2='1451530_at',CellID2='',rank='0')) | [0.472 38](javascript:showCorrelationPlot2(db='MA_M2M_0706_R',ProbeSetID='1448943_at',CellID='',db2='MA_M2M_0706_R',ProbeSetID2='1423785_at',CellID2='',rank='0')) | [-0.466 38](javascript:showCorrelationPlot2(db='MA_M2M_0706_R',ProbeSetID='1448943_at',CellID='',db2='MA_M2M_0706_R',ProbeSetID2='1431972_a_at',CellID2='',rank='0')) | [0.558 38](javascript:showCorrelationPlot2(db='MA_M2M_0706_R',ProbeSetID='1448943_at',CellID='',db2='MA_M2M_0706_R',ProbeSetID2='1448347_a_at',CellID2='',rank='0')) | [-0.310 38](javascript:showCorrelationPlot2(db='MA_M2M_0706_R',ProbeSetID='1448943_at',CellID='',db2='MA_M2M_0706_R',ProbeSetID2='1418492_at',CellID2='',rank='0')) | [0.614 38](javascript:showCorrelationPlot2(db='MA_M2M_0706_R',ProbeSetID='1448943_at',CellID='',db2='MA_M2M_0706_R',ProbeSetID2='1427185_at',CellID2='',rank='0')) | [0.328 38](javascript:showCorrelationPlot2(db='MA_M2M_0706_R',ProbeSetID='1448943_at',CellID='',db2='MA_M2M_0706_R',ProbeSetID2='1430500_s_at',CellID2='',rank='0')) | [-0.371 38](javascript:showCorrelationPlot2(db='MA_M2M_0706_R',ProbeSetID='1448943_at',CellID='',db2='MA_M2M_0706_R',ProbeSetID2='1447549_x_at',CellID2='',rank='0')) | [n 38](javascript:showDatabase2('MA_M2M_0706_R','1448943_at','')) | [-0.422 38](javascript:showCorrelationPlot2(db='MA_M2M_0706_R',ProbeSetID='1448943_at',CellID='',db2='MA_M2M_0706_R',ProbeSetID2='1433162_at',CellID2='',rank='1')) | [-0.284 38](javascript:showCorrelationPlot2(db='MA_M2M_0706_R',ProbeSetID='1448943_at',CellID='',db2='MA_M2M_0706_R',ProbeSetID2='1443494_at',CellID2='',rank='1')) | [0.653 38](javascript:showCorrelationPlot2(db='MA_M2M_0706_R',ProbeSetID='1448943_at',CellID='',db2='MA_M2M_0706_R',ProbeSetID2='1417426_at',CellID2='',rank='1')) | [-0.492 38](javascript:showCorrelationPlot2(db='MA_M2M_0706_R',ProbeSetID='1448943_at',CellID='',db2='MA_M2M_0706_R',ProbeSetID2='1416588_at',CellID2='',rank='1')) | [0.545 38](javascript:showCorrelationPlot2(db='MA_M2M_0706_R',ProbeSetID='1448943_at',CellID='',db2='MA_M2M_0706_R',ProbeSetID2='1450994_at',CellID2='',rank='1')) | [-0.427 38](javascript:showCorrelationPlot2(db='MA_M2M_0706_R',ProbeSetID='1448943_at',CellID='',db2='MA_M2M_0706_R',ProbeSetID2='1420304_x_at',CellID2='',rank='1')) | [0.438 38](javascript:showCorrelationPlot2(db='MA_M2M_0706_R',ProbeSetID='1448943_at',CellID='',db2='MA_M2M_0706_R',ProbeSetID2='1416500_at',CellID2='',rank='1')) | [-0.376 38](javascript:showCorrelationPlot2(db='MA_M2M_0706_R',ProbeSetID='1448943_at',CellID='',db2='MA_M2M_0706_R',ProbeSetID2='1424199_at',CellID2='',rank='1')) | [0.689 38](javascript:showCorrelationPlot2(db='MA_M2M_0706_R',ProbeSetID='1448943_at',CellID='',db2='MA_M2M_0706_R',ProbeSetID2='1437995_x_at',CellID2='',rank='1')) | [0.490 38](javascript:showCorrelationPlot2(db='MA_M2M_0706_R',ProbeSetID='1448943_at',CellID='',db2='MA_M2M_0706_R',ProbeSetID2='1427914_a_at',CellID2='',rank='1')) | [-0.351 38](javascript:showCorrelationPlot2(db='MA_M2M_0706_R',ProbeSetID='1448943_at',CellID='',db2='MA_M2M_0706_R',ProbeSetID2='1420570_x_at',CellID2='',rank='1')) | [-0.440 38](javascript:showCorrelationPlot2(db='MA_M2M_0706_R',ProbeSetID='1448943_at',CellID='',db2='MA_M2M_0706_R',ProbeSetID2='1431015_at',CellID2='',rank='1')) | [0.288 38](javascript:showCorrelationPlot2(db='MA_M2M_0706_R',ProbeSetID='1448943_at',CellID='',db2='MA_M2M_0706_R',ProbeSetID2='1423852_at',CellID2='',rank='1')) | [-0.451 38](javascript:showCorrelationPlot2(db='MA_M2M_0706_R',ProbeSetID='1448943_at',CellID='',db2='MA_M2M_0706_R',ProbeSetID2='1431799_at',CellID2='',rank='1')) | [0.461 38](javascript:showCorrelationPlot2(db='MA_M2M_0706_R',ProbeSetID='1448943_at',CellID='',db2='MA_M2M_0706_R',ProbeSetID2='1423898_a_at',CellID2='',rank='1')) | [-0.475 38](javascript:showCorrelationPlot2(db='MA_M2M_0706_R',ProbeSetID='1448943_at',CellID='',db2='MA_M2M_0706_R',ProbeSetID2='1438579_at',CellID2='',rank='1')) | [0.262 38](javascript:showCorrelationPlot2(db='MA_M2M_0706_R',ProbeSetID='1448943_at',CellID='',db2='MA_M2M_0706_R',ProbeSetID2='1448102_a_at',CellID2='',rank='1')) | [-0.354 38](javascript:showCorrelationPlot2(db='MA_M2M_0706_R',ProbeSetID='1448943_at',CellID='',db2='MA_M2M_0706_R',ProbeSetID2='1420943_at',CellID2='',rank='1')) | [-0.292 38](javascript:showCorrelationPlot2(db='MA_M2M_0706_R',ProbeSetID='1448943_at',CellID='',db2='MA_M2M_0706_R',ProbeSetID2='1447326_s_at',CellID2='',rank='1')) |
|  | [Trait 32: MA_M2M_0706_R::1433162_at](javascript:showDatabase2('MA_M2M_0706_R','1433162_at','');)  Plek on Chr 11 @ 16.858909 Mb  pleckstrin associated EST AK008484  antisense in AK008484 but same sense as Plek | [-0.667 38](javascript:showCorrelationPlot2(db='MA_M2M_0706_R',ProbeSetID='1433162_at',CellID='',db2='MA_M2M_0706_R',ProbeSetID2='1445101_at',CellID2='',rank='0')) | [0.348 38](javascript:showCorrelationPlot2(db='MA_M2M_0706_R',ProbeSetID='1433162_at',CellID='',db2='MA_M2M_0706_R',ProbeSetID2='1451285_at',CellID2='',rank='0')) | [0.615 38](javascript:showCorrelationPlot2(db='MA_M2M_0706_R',ProbeSetID='1433162_at',CellID='',db2='MA_M2M_0706_R',ProbeSetID2='1441347_at',CellID2='',rank='0')) | [-0.563 38](javascript:showCorrelationPlot2(db='MA_M2M_0706_R',ProbeSetID='1433162_at',CellID='',db2='MA_M2M_0706_R',ProbeSetID2='1426900_at',CellID2='',rank='0')) | [-0.438 38](javascript:showCorrelationPlot2(db='MA_M2M_0706_R',ProbeSetID='1433162_at',CellID='',db2='MA_M2M_0706_R',ProbeSetID2='1434888_a_at',CellID2='',rank='0')) | [-0.517 38](javascript:showCorrelationPlot2(db='MA_M2M_0706_R',ProbeSetID='1433162_at',CellID='',db2='MA_M2M_0706_R',ProbeSetID2='1434773_a_at',CellID2='',rank='0')) | [0.682 38](javascript:showCorrelationPlot2(db='MA_M2M_0706_R',ProbeSetID='1433162_at',CellID='',db2='MA_M2M_0706_R',ProbeSetID2='1452851_at',CellID2='',rank='0')) | [-0.523 38](javascript:showCorrelationPlot2(db='MA_M2M_0706_R',ProbeSetID='1433162_at',CellID='',db2='MA_M2M_0706_R',ProbeSetID2='1441948_x_at',CellID2='',rank='0')) | [0.748 38](javascript:showCorrelationPlot2(db='MA_M2M_0706_R',ProbeSetID='1433162_at',CellID='',db2='MA_M2M_0706_R',ProbeSetID2='1440160_x_at',CellID2='',rank='0')) | [-0.641 38](javascript:showCorrelationPlot2(db='MA_M2M_0706_R',ProbeSetID='1433162_at',CellID='',db2='MA_M2M_0706_R',ProbeSetID2='1424692_at',CellID2='',rank='0')) | [0.731 38](javascript:showCorrelationPlot2(db='MA_M2M_0706_R',ProbeSetID='1433162_at',CellID='',db2='MA_M2M_0706_R',ProbeSetID2='1433112_at',CellID2='',rank='0')) | [-0.532 38](javascript:showCorrelationPlot2(db='MA_M2M_0706_R',ProbeSetID='1433162_at',CellID='',db2='MA_M2M_0706_R',ProbeSetID2='1459962_at',CellID2='',rank='0')) | [0.625 38](javascript:showCorrelationPlot2(db='MA_M2M_0706_R',ProbeSetID='1433162_at',CellID='',db2='MA_M2M_0706_R',ProbeSetID2='1455331_at',CellID2='',rank='0')) | [0.540 38](javascript:showCorrelationPlot2(db='MA_M2M_0706_R',ProbeSetID='1433162_at',CellID='',db2='MA_M2M_0706_R',ProbeSetID2='1447399_at',CellID2='',rank='0')) | [-0.551 38](javascript:showCorrelationPlot2(db='MA_M2M_0706_R',ProbeSetID='1433162_at',CellID='',db2='MA_M2M_0706_R',ProbeSetID2='1434968_a_at',CellID2='',rank='0')) | [0.754 38](javascript:showCorrelationPlot2(db='MA_M2M_0706_R',ProbeSetID='1433162_at',CellID='',db2='MA_M2M_0706_R',ProbeSetID2='1447335_x_at',CellID2='',rank='0')) | [0.759 38](javascript:showCorrelationPlot2(db='MA_M2M_0706_R',ProbeSetID='1433162_at',CellID='',db2='MA_M2M_0706_R',ProbeSetID2='1442239_at',CellID2='',rank='0')) | [0.666 38](javascript:showCorrelationPlot2(db='MA_M2M_0706_R',ProbeSetID='1433162_at',CellID='',db2='MA_M2M_0706_R',ProbeSetID2='1459865_x_at',CellID2='',rank='0')) | [-0.392 38](javascript:showCorrelationPlot2(db='MA_M2M_0706_R',ProbeSetID='1433162_at',CellID='',db2='MA_M2M_0706_R',ProbeSetID2='1438647_x_at',CellID2='',rank='0')) | [0.575 38](javascript:showCorrelationPlot2(db='MA_M2M_0706_R',ProbeSetID='1433162_at',CellID='',db2='MA_M2M_0706_R',ProbeSetID2='1432333_a_at',CellID2='',rank='0')) | [-0.488 38](javascript:showCorrelationPlot2(db='MA_M2M_0706_R',ProbeSetID='1433162_at',CellID='',db2='MA_M2M_0706_R',ProbeSetID2='1444028_s_at',CellID2='',rank='0')) | [0.637 38](javascript:showCorrelationPlot2(db='MA_M2M_0706_R',ProbeSetID='1433162_at',CellID='',db2='MA_M2M_0706_R',ProbeSetID2='1460420_a_at',CellID2='',rank='0')) | [0.658 38](javascript:showCorrelationPlot2(db='MA_M2M_0706_R',ProbeSetID='1433162_at',CellID='',db2='MA_M2M_0706_R',ProbeSetID2='1451530_at',CellID2='',rank='0')) | [-0.582 38](javascript:showCorrelationPlot2(db='MA_M2M_0706_R',ProbeSetID='1433162_at',CellID='',db2='MA_M2M_0706_R',ProbeSetID2='1423785_at',CellID2='',rank='0')) | [0.365 38](javascript:showCorrelationPlot2(db='MA_M2M_0706_R',ProbeSetID='1433162_at',CellID='',db2='MA_M2M_0706_R',ProbeSetID2='1431972_a_at',CellID2='',rank='0')) | [-0.614 38](javascript:showCorrelationPlot2(db='MA_M2M_0706_R',ProbeSetID='1433162_at',CellID='',db2='MA_M2M_0706_R',ProbeSetID2='1448347_a_at',CellID2='',rank='0')) | [0.551 38](javascript:showCorrelationPlot2(db='MA_M2M_0706_R',ProbeSetID='1433162_at',CellID='',db2='MA_M2M_0706_R',ProbeSetID2='1418492_at',CellID2='',rank='0')) | [-0.469 38](javascript:showCorrelationPlot2(db='MA_M2M_0706_R',ProbeSetID='1433162_at',CellID='',db2='MA_M2M_0706_R',ProbeSetID2='1427185_at',CellID2='',rank='0')) | [-0.525 38](javascript:showCorrelationPlot2(db='MA_M2M_0706_R',ProbeSetID='1433162_at',CellID='',db2='MA_M2M_0706_R',ProbeSetID2='1430500_s_at',CellID2='',rank='0')) | [0.565 38](javascript:showCorrelationPlot2(db='MA_M2M_0706_R',ProbeSetID='1433162_at',CellID='',db2='MA_M2M_0706_R',ProbeSetID2='1447549_x_at',CellID2='',rank='0')) | [-0.496 38](javascript:showCorrelationPlot2(db='MA_M2M_0706_R',ProbeSetID='1433162_at',CellID='',db2='MA_M2M_0706_R',ProbeSetID2='1448943_at',CellID2='',rank='0')) | [n 38](javascript:showDatabase2('MA_M2M_0706_R','1433162_at','')) | [0.609 38](javascript:showCorrelationPlot2(db='MA_M2M_0706_R',ProbeSetID='1433162_at',CellID='',db2='MA_M2M_0706_R',ProbeSetID2='1443494_at',CellID2='',rank='1')) | [-0.253 38](javascript:showCorrelationPlot2(db='MA_M2M_0706_R',ProbeSetID='1433162_at',CellID='',db2='MA_M2M_0706_R',ProbeSetID2='1417426_at',CellID2='',rank='1')) | [0.743 38](javascript:showCorrelationPlot2(db='MA_M2M_0706_R',ProbeSetID='1433162_at',CellID='',db2='MA_M2M_0706_R',ProbeSetID2='1416588_at',CellID2='',rank='1')) | [-0.457 38](javascript:showCorrelationPlot2(db='MA_M2M_0706_R',ProbeSetID='1433162_at',CellID='',db2='MA_M2M_0706_R',ProbeSetID2='1450994_at',CellID2='',rank='1')) | [0.572 38](javascript:showCorrelationPlot2(db='MA_M2M_0706_R',ProbeSetID='1433162_at',CellID='',db2='MA_M2M_0706_R',ProbeSetID2='1420304_x_at',CellID2='',rank='1')) | [-0.512 38](javascript:showCorrelationPlot2(db='MA_M2M_0706_R',ProbeSetID='1433162_at',CellID='',db2='MA_M2M_0706_R',ProbeSetID2='1416500_at',CellID2='',rank='1')) | [0.692 38](javascript:showCorrelationPlot2(db='MA_M2M_0706_R',ProbeSetID='1433162_at',CellID='',db2='MA_M2M_0706_R',ProbeSetID2='1424199_at',CellID2='',rank='1')) | [-0.400 38](javascript:showCorrelationPlot2(db='MA_M2M_0706_R',ProbeSetID='1433162_at',CellID='',db2='MA_M2M_0706_R',ProbeSetID2='1437995_x_at',CellID2='',rank='1')) | [-0.453 38](javascript:showCorrelationPlot2(db='MA_M2M_0706_R',ProbeSetID='1433162_at',CellID='',db2='MA_M2M_0706_R',ProbeSetID2='1427914_a_at',CellID2='',rank='1')) | [0.700 38](javascript:showCorrelationPlot2(db='MA_M2M_0706_R',ProbeSetID='1433162_at',CellID='',db2='MA_M2M_0706_R',ProbeSetID2='1420570_x_at',CellID2='',rank='1')) | [0.520 38](javascript:showCorrelationPlot2(db='MA_M2M_0706_R',ProbeSetID='1433162_at',CellID='',db2='MA_M2M_0706_R',ProbeSetID2='1431015_at',CellID2='',rank='1')) | [-0.476 38](javascript:showCorrelationPlot2(db='MA_M2M_0706_R',ProbeSetID='1433162_at',CellID='',db2='MA_M2M_0706_R',ProbeSetID2='1423852_at',CellID2='',rank='1')) | [0.411 38](javascript:showCorrelationPlot2(db='MA_M2M_0706_R',ProbeSetID='1433162_at',CellID='',db2='MA_M2M_0706_R',ProbeSetID2='1431799_at',CellID2='',rank='1')) | [-0.719 38](javascript:showCorrelationPlot2(db='MA_M2M_0706_R',ProbeSetID='1433162_at',CellID='',db2='MA_M2M_0706_R',ProbeSetID2='1423898_a_at',CellID2='',rank='1')) | [0.490 38](javascript:showCorrelationPlot2(db='MA_M2M_0706_R',ProbeSetID='1433162_at',CellID='',db2='MA_M2M_0706_R',ProbeSetID2='1438579_at',CellID2='',rank='1')) | [-0.407 38](javascript:showCorrelationPlot2(db='MA_M2M_0706_R',ProbeSetID='1433162_at',CellID='',db2='MA_M2M_0706_R',ProbeSetID2='1448102_a_at',CellID2='',rank='1')) | [0.384 38](javascript:showCorrelationPlot2(db='MA_M2M_0706_R',ProbeSetID='1433162_at',CellID='',db2='MA_M2M_0706_R',ProbeSetID2='1420943_at',CellID2='',rank='1')) | [0.540 38](javascript:showCorrelationPlot2(db='MA_M2M_0706_R',ProbeSetID='1433162_at',CellID='',db2='MA_M2M_0706_R',ProbeSetID2='1447326_s_at',CellID2='',rank='1')) |
|  | [Trait 33: MA_M2M_0706_R::1443494_at](javascript:showDatabase2('MA_M2M_0706_R','1443494_at','');)  Polr3b on Chr 10 @ 84.097688 Mb  polymerase (RNA) III (DNA directed) polypeptide B | [-0.592 38](javascript:showCorrelationPlot2(db='MA_M2M_0706_R',ProbeSetID='1443494_at',CellID='',db2='MA_M2M_0706_R',ProbeSetID2='1445101_at',CellID2='',rank='0')) | [0.392 38](javascript:showCorrelationPlot2(db='MA_M2M_0706_R',ProbeSetID='1443494_at',CellID='',db2='MA_M2M_0706_R',ProbeSetID2='1451285_at',CellID2='',rank='0')) | [0.592 38](javascript:showCorrelationPlot2(db='MA_M2M_0706_R',ProbeSetID='1443494_at',CellID='',db2='MA_M2M_0706_R',ProbeSetID2='1441347_at',CellID2='',rank='0')) | [-0.552 38](javascript:showCorrelationPlot2(db='MA_M2M_0706_R',ProbeSetID='1443494_at',CellID='',db2='MA_M2M_0706_R',ProbeSetID2='1426900_at',CellID2='',rank='0')) | [-0.492 38](javascript:showCorrelationPlot2(db='MA_M2M_0706_R',ProbeSetID='1443494_at',CellID='',db2='MA_M2M_0706_R',ProbeSetID2='1434888_a_at',CellID2='',rank='0')) | [-0.449 38](javascript:showCorrelationPlot2(db='MA_M2M_0706_R',ProbeSetID='1443494_at',CellID='',db2='MA_M2M_0706_R',ProbeSetID2='1434773_a_at',CellID2='',rank='0')) | [0.564 38](javascript:showCorrelationPlot2(db='MA_M2M_0706_R',ProbeSetID='1443494_at',CellID='',db2='MA_M2M_0706_R',ProbeSetID2='1452851_at',CellID2='',rank='0')) | [-0.408 38](javascript:showCorrelationPlot2(db='MA_M2M_0706_R',ProbeSetID='1443494_at',CellID='',db2='MA_M2M_0706_R',ProbeSetID2='1441948_x_at',CellID2='',rank='0')) | [0.584 38](javascript:showCorrelationPlot2(db='MA_M2M_0706_R',ProbeSetID='1443494_at',CellID='',db2='MA_M2M_0706_R',ProbeSetID2='1440160_x_at',CellID2='',rank='0')) | [-0.452 38](javascript:showCorrelationPlot2(db='MA_M2M_0706_R',ProbeSetID='1443494_at',CellID='',db2='MA_M2M_0706_R',ProbeSetID2='1424692_at',CellID2='',rank='0')) | [0.566 38](javascript:showCorrelationPlot2(db='MA_M2M_0706_R',ProbeSetID='1443494_at',CellID='',db2='MA_M2M_0706_R',ProbeSetID2='1433112_at',CellID2='',rank='0')) | [-0.466 38](javascript:showCorrelationPlot2(db='MA_M2M_0706_R',ProbeSetID='1443494_at',CellID='',db2='MA_M2M_0706_R',ProbeSetID2='1459962_at',CellID2='',rank='0')) | [0.392 38](javascript:showCorrelationPlot2(db='MA_M2M_0706_R',ProbeSetID='1443494_at',CellID='',db2='MA_M2M_0706_R',ProbeSetID2='1455331_at',CellID2='',rank='0')) | [0.422 38](javascript:showCorrelationPlot2(db='MA_M2M_0706_R',ProbeSetID='1443494_at',CellID='',db2='MA_M2M_0706_R',ProbeSetID2='1447399_at',CellID2='',rank='0')) | [-0.404 38](javascript:showCorrelationPlot2(db='MA_M2M_0706_R',ProbeSetID='1443494_at',CellID='',db2='MA_M2M_0706_R',ProbeSetID2='1434968_a_at',CellID2='',rank='0')) | [0.589 38](javascript:showCorrelationPlot2(db='MA_M2M_0706_R',ProbeSetID='1443494_at',CellID='',db2='MA_M2M_0706_R',ProbeSetID2='1447335_x_at',CellID2='',rank='0')) | [0.639 38](javascript:showCorrelationPlot2(db='MA_M2M_0706_R',ProbeSetID='1443494_at',CellID='',db2='MA_M2M_0706_R',ProbeSetID2='1442239_at',CellID2='',rank='0')) | [0.593 38](javascript:showCorrelationPlot2(db='MA_M2M_0706_R',ProbeSetID='1443494_at',CellID='',db2='MA_M2M_0706_R',ProbeSetID2='1459865_x_at',CellID2='',rank='0')) | [-0.582 38](javascript:showCorrelationPlot2(db='MA_M2M_0706_R',ProbeSetID='1443494_at',CellID='',db2='MA_M2M_0706_R',ProbeSetID2='1438647_x_at',CellID2='',rank='0')) | [0.438 38](javascript:showCorrelationPlot2(db='MA_M2M_0706_R',ProbeSetID='1443494_at',CellID='',db2='MA_M2M_0706_R',ProbeSetID2='1432333_a_at',CellID2='',rank='0')) | [-0.412 38](javascript:showCorrelationPlot2(db='MA_M2M_0706_R',ProbeSetID='1443494_at',CellID='',db2='MA_M2M_0706_R',ProbeSetID2='1444028_s_at',CellID2='',rank='0')) | [0.633 38](javascript:showCorrelationPlot2(db='MA_M2M_0706_R',ProbeSetID='1443494_at',CellID='',db2='MA_M2M_0706_R',ProbeSetID2='1460420_a_at',CellID2='',rank='0')) | [0.593 38](javascript:showCorrelationPlot2(db='MA_M2M_0706_R',ProbeSetID='1443494_at',CellID='',db2='MA_M2M_0706_R',ProbeSetID2='1451530_at',CellID2='',rank='0')) | [-0.488 38](javascript:showCorrelationPlot2(db='MA_M2M_0706_R',ProbeSetID='1443494_at',CellID='',db2='MA_M2M_0706_R',ProbeSetID2='1423785_at',CellID2='',rank='0')) | [0.434 38](javascript:showCorrelationPlot2(db='MA_M2M_0706_R',ProbeSetID='1443494_at',CellID='',db2='MA_M2M_0706_R',ProbeSetID2='1431972_a_at',CellID2='',rank='0')) | [-0.447 38](javascript:showCorrelationPlot2(db='MA_M2M_0706_R',ProbeSetID='1443494_at',CellID='',db2='MA_M2M_0706_R',ProbeSetID2='1448347_a_at',CellID2='',rank='0')) | [0.546 38](javascript:showCorrelationPlot2(db='MA_M2M_0706_R',ProbeSetID='1443494_at',CellID='',db2='MA_M2M_0706_R',ProbeSetID2='1418492_at',CellID2='',rank='0')) | [-0.448 38](javascript:showCorrelationPlot2(db='MA_M2M_0706_R',ProbeSetID='1443494_at',CellID='',db2='MA_M2M_0706_R',ProbeSetID2='1427185_at',CellID2='',rank='0')) | [-0.561 38](javascript:showCorrelationPlot2(db='MA_M2M_0706_R',ProbeSetID='1443494_at',CellID='',db2='MA_M2M_0706_R',ProbeSetID2='1430500_s_at',CellID2='',rank='0')) | [0.635 38](javascript:showCorrelationPlot2(db='MA_M2M_0706_R',ProbeSetID='1443494_at',CellID='',db2='MA_M2M_0706_R',ProbeSetID2='1447549_x_at',CellID2='',rank='0')) | [-0.448 38](javascript:showCorrelationPlot2(db='MA_M2M_0706_R',ProbeSetID='1443494_at',CellID='',db2='MA_M2M_0706_R',ProbeSetID2='1448943_at',CellID2='',rank='0')) | [0.626 38](javascript:showCorrelationPlot2(db='MA_M2M_0706_R',ProbeSetID='1443494_at',CellID='',db2='MA_M2M_0706_R',ProbeSetID2='1433162_at',CellID2='',rank='0')) | [n 38](javascript:showDatabase2('MA_M2M_0706_R','1443494_at','')) | [-0.221 38](javascript:showCorrelationPlot2(db='MA_M2M_0706_R',ProbeSetID='1443494_at',CellID='',db2='MA_M2M_0706_R',ProbeSetID2='1417426_at',CellID2='',rank='1')) | [0.626 38](javascript:showCorrelationPlot2(db='MA_M2M_0706_R',ProbeSetID='1443494_at',CellID='',db2='MA_M2M_0706_R',ProbeSetID2='1416588_at',CellID2='',rank='1')) | [-0.418 38](javascript:showCorrelationPlot2(db='MA_M2M_0706_R',ProbeSetID='1443494_at',CellID='',db2='MA_M2M_0706_R',ProbeSetID2='1450994_at',CellID2='',rank='1')) | [0.602 38](javascript:showCorrelationPlot2(db='MA_M2M_0706_R',ProbeSetID='1443494_at',CellID='',db2='MA_M2M_0706_R',ProbeSetID2='1420304_x_at',CellID2='',rank='1')) | [-0.604 38](javascript:showCorrelationPlot2(db='MA_M2M_0706_R',ProbeSetID='1443494_at',CellID='',db2='MA_M2M_0706_R',ProbeSetID2='1416500_at',CellID2='',rank='1')) | [0.642 38](javascript:showCorrelationPlot2(db='MA_M2M_0706_R',ProbeSetID='1443494_at',CellID='',db2='MA_M2M_0706_R',ProbeSetID2='1424199_at',CellID2='',rank='1')) | [-0.501 38](javascript:showCorrelationPlot2(db='MA_M2M_0706_R',ProbeSetID='1443494_at',CellID='',db2='MA_M2M_0706_R',ProbeSetID2='1437995_x_at',CellID2='',rank='1')) | [-0.414 38](javascript:showCorrelationPlot2(db='MA_M2M_0706_R',ProbeSetID='1443494_at',CellID='',db2='MA_M2M_0706_R',ProbeSetID2='1427914_a_at',CellID2='',rank='1')) | [0.578 38](javascript:showCorrelationPlot2(db='MA_M2M_0706_R',ProbeSetID='1443494_at',CellID='',db2='MA_M2M_0706_R',ProbeSetID2='1420570_x_at',CellID2='',rank='1')) | [0.662 38](javascript:showCorrelationPlot2(db='MA_M2M_0706_R',ProbeSetID='1443494_at',CellID='',db2='MA_M2M_0706_R',ProbeSetID2='1431015_at',CellID2='',rank='1')) | [-0.457 38](javascript:showCorrelationPlot2(db='MA_M2M_0706_R',ProbeSetID='1443494_at',CellID='',db2='MA_M2M_0706_R',ProbeSetID2='1423852_at',CellID2='',rank='1')) | [0.431 38](javascript:showCorrelationPlot2(db='MA_M2M_0706_R',ProbeSetID='1443494_at',CellID='',db2='MA_M2M_0706_R',ProbeSetID2='1431799_at',CellID2='',rank='1')) | [-0.543 38](javascript:showCorrelationPlot2(db='MA_M2M_0706_R',ProbeSetID='1443494_at',CellID='',db2='MA_M2M_0706_R',ProbeSetID2='1423898_a_at',CellID2='',rank='1')) | [0.543 38](javascript:showCorrelationPlot2(db='MA_M2M_0706_R',ProbeSetID='1443494_at',CellID='',db2='MA_M2M_0706_R',ProbeSetID2='1438579_at',CellID2='',rank='1')) | [-0.626 38](javascript:showCorrelationPlot2(db='MA_M2M_0706_R',ProbeSetID='1443494_at',CellID='',db2='MA_M2M_0706_R',ProbeSetID2='1448102_a_at',CellID2='',rank='1')) | [0.458 38](javascript:showCorrelationPlot2(db='MA_M2M_0706_R',ProbeSetID='1443494_at',CellID='',db2='MA_M2M_0706_R',ProbeSetID2='1420943_at',CellID2='',rank='1')) | [0.603 38](javascript:showCorrelationPlot2(db='MA_M2M_0706_R',ProbeSetID='1443494_at',CellID='',db2='MA_M2M_0706_R',ProbeSetID2='1447326_s_at',CellID2='',rank='1')) |
|  | [Trait 34: MA_M2M_0706_R::1417426_at](javascript:showDatabase2('MA_M2M_0706_R','1417426_at','');)  Prg1 on Chr 10 @ 61.957393 Mb  proteoglycan 1, secretory granule  last exon and 3' UTR | [0.226 38](javascript:showCorrelationPlot2(db='MA_M2M_0706_R',ProbeSetID='1417426_at',CellID='',db2='MA_M2M_0706_R',ProbeSetID2='1445101_at',CellID2='',rank='0')) | [-0.369 38](javascript:showCorrelationPlot2(db='MA_M2M_0706_R',ProbeSetID='1417426_at',CellID='',db2='MA_M2M_0706_R',ProbeSetID2='1451285_at',CellID2='',rank='0')) | [-0.359 38](javascript:showCorrelationPlot2(db='MA_M2M_0706_R',ProbeSetID='1417426_at',CellID='',db2='MA_M2M_0706_R',ProbeSetID2='1441347_at',CellID2='',rank='0')) | [0.458 38](javascript:showCorrelationPlot2(db='MA_M2M_0706_R',ProbeSetID='1417426_at',CellID='',db2='MA_M2M_0706_R',ProbeSetID2='1426900_at',CellID2='',rank='0')) | [0.425 38](javascript:showCorrelationPlot2(db='MA_M2M_0706_R',ProbeSetID='1417426_at',CellID='',db2='MA_M2M_0706_R',ProbeSetID2='1434888_a_at',CellID2='',rank='0')) | [0.596 38](javascript:showCorrelationPlot2(db='MA_M2M_0706_R',ProbeSetID='1417426_at',CellID='',db2='MA_M2M_0706_R',ProbeSetID2='1434773_a_at',CellID2='',rank='0')) | [-0.617 38](javascript:showCorrelationPlot2(db='MA_M2M_0706_R',ProbeSetID='1417426_at',CellID='',db2='MA_M2M_0706_R',ProbeSetID2='1452851_at',CellID2='',rank='0')) | [0.529 38](javascript:showCorrelationPlot2(db='MA_M2M_0706_R',ProbeSetID='1417426_at',CellID='',db2='MA_M2M_0706_R',ProbeSetID2='1441948_x_at',CellID2='',rank='0')) | [-0.389 38](javascript:showCorrelationPlot2(db='MA_M2M_0706_R',ProbeSetID='1417426_at',CellID='',db2='MA_M2M_0706_R',ProbeSetID2='1440160_x_at',CellID2='',rank='0')) | [0.442 38](javascript:showCorrelationPlot2(db='MA_M2M_0706_R',ProbeSetID='1417426_at',CellID='',db2='MA_M2M_0706_R',ProbeSetID2='1424692_at',CellID2='',rank='0')) | [-0.350 38](javascript:showCorrelationPlot2(db='MA_M2M_0706_R',ProbeSetID='1417426_at',CellID='',db2='MA_M2M_0706_R',ProbeSetID2='1433112_at',CellID2='',rank='0')) | [0.528 38](javascript:showCorrelationPlot2(db='MA_M2M_0706_R',ProbeSetID='1417426_at',CellID='',db2='MA_M2M_0706_R',ProbeSetID2='1459962_at',CellID2='',rank='0')) | [-0.442 38](javascript:showCorrelationPlot2(db='MA_M2M_0706_R',ProbeSetID='1417426_at',CellID='',db2='MA_M2M_0706_R',ProbeSetID2='1455331_at',CellID2='',rank='0')) | [-0.484 38](javascript:showCorrelationPlot2(db='MA_M2M_0706_R',ProbeSetID='1417426_at',CellID='',db2='MA_M2M_0706_R',ProbeSetID2='1447399_at',CellID2='',rank='0')) | [0.242 38](javascript:showCorrelationPlot2(db='MA_M2M_0706_R',ProbeSetID='1417426_at',CellID='',db2='MA_M2M_0706_R',ProbeSetID2='1434968_a_at',CellID2='',rank='0')) | [-0.455 38](javascript:showCorrelationPlot2(db='MA_M2M_0706_R',ProbeSetID='1417426_at',CellID='',db2='MA_M2M_0706_R',ProbeSetID2='1447335_x_at',CellID2='',rank='0')) | [-0.282 38](javascript:showCorrelationPlot2(db='MA_M2M_0706_R',ProbeSetID='1417426_at',CellID='',db2='MA_M2M_0706_R',ProbeSetID2='1442239_at',CellID2='',rank='0')) | [-0.460 38](javascript:showCorrelationPlot2(db='MA_M2M_0706_R',ProbeSetID='1417426_at',CellID='',db2='MA_M2M_0706_R',ProbeSetID2='1459865_x_at',CellID2='',rank='0')) | [0.400 38](javascript:showCorrelationPlot2(db='MA_M2M_0706_R',ProbeSetID='1417426_at',CellID='',db2='MA_M2M_0706_R',ProbeSetID2='1438647_x_at',CellID2='',rank='0')) | [-0.420 38](javascript:showCorrelationPlot2(db='MA_M2M_0706_R',ProbeSetID='1417426_at',CellID='',db2='MA_M2M_0706_R',ProbeSetID2='1432333_a_at',CellID2='',rank='0')) | [0.586 38](javascript:showCorrelationPlot2(db='MA_M2M_0706_R',ProbeSetID='1417426_at',CellID='',db2='MA_M2M_0706_R',ProbeSetID2='1444028_s_at',CellID2='',rank='0')) | [-0.632 38](javascript:showCorrelationPlot2(db='MA_M2M_0706_R',ProbeSetID='1417426_at',CellID='',db2='MA_M2M_0706_R',ProbeSetID2='1460420_a_at',CellID2='',rank='0')) | [-0.373 38](javascript:showCorrelationPlot2(db='MA_M2M_0706_R',ProbeSetID='1417426_at',CellID='',db2='MA_M2M_0706_R',ProbeSetID2='1451530_at',CellID2='',rank='0')) | [0.391 38](javascript:showCorrelationPlot2(db='MA_M2M_0706_R',ProbeSetID='1417426_at',CellID='',db2='MA_M2M_0706_R',ProbeSetID2='1423785_at',CellID2='',rank='0')) | [-0.423 38](javascript:showCorrelationPlot2(db='MA_M2M_0706_R',ProbeSetID='1417426_at',CellID='',db2='MA_M2M_0706_R',ProbeSetID2='1431972_a_at',CellID2='',rank='0')) | [0.497 38](javascript:showCorrelationPlot2(db='MA_M2M_0706_R',ProbeSetID='1417426_at',CellID='',db2='MA_M2M_0706_R',ProbeSetID2='1448347_a_at',CellID2='',rank='0')) | [-0.382 38](javascript:showCorrelationPlot2(db='MA_M2M_0706_R',ProbeSetID='1417426_at',CellID='',db2='MA_M2M_0706_R',ProbeSetID2='1418492_at',CellID2='',rank='0')) | [0.612 38](javascript:showCorrelationPlot2(db='MA_M2M_0706_R',ProbeSetID='1417426_at',CellID='',db2='MA_M2M_0706_R',ProbeSetID2='1427185_at',CellID2='',rank='0')) | [0.342 38](javascript:showCorrelationPlot2(db='MA_M2M_0706_R',ProbeSetID='1417426_at',CellID='',db2='MA_M2M_0706_R',ProbeSetID2='1430500_s_at',CellID2='',rank='0')) | [-0.413 38](javascript:showCorrelationPlot2(db='MA_M2M_0706_R',ProbeSetID='1417426_at',CellID='',db2='MA_M2M_0706_R',ProbeSetID2='1447549_x_at',CellID2='',rank='0')) | [0.637 38](javascript:showCorrelationPlot2(db='MA_M2M_0706_R',ProbeSetID='1417426_at',CellID='',db2='MA_M2M_0706_R',ProbeSetID2='1448943_at',CellID2='',rank='0')) | [-0.335 38](javascript:showCorrelationPlot2(db='MA_M2M_0706_R',ProbeSetID='1417426_at',CellID='',db2='MA_M2M_0706_R',ProbeSetID2='1433162_at',CellID2='',rank='0')) | [-0.268 38](javascript:showCorrelationPlot2(db='MA_M2M_0706_R',ProbeSetID='1417426_at',CellID='',db2='MA_M2M_0706_R',ProbeSetID2='1443494_at',CellID2='',rank='0')) | [n 38](javascript:showDatabase2('MA_M2M_0706_R','1417426_at','')) | [-0.425 38](javascript:showCorrelationPlot2(db='MA_M2M_0706_R',ProbeSetID='1417426_at',CellID='',db2='MA_M2M_0706_R',ProbeSetID2='1416588_at',CellID2='',rank='1')) | [0.601 38](javascript:showCorrelationPlot2(db='MA_M2M_0706_R',ProbeSetID='1417426_at',CellID='',db2='MA_M2M_0706_R',ProbeSetID2='1450994_at',CellID2='',rank='1')) | [-0.439 38](javascript:showCorrelationPlot2(db='MA_M2M_0706_R',ProbeSetID='1417426_at',CellID='',db2='MA_M2M_0706_R',ProbeSetID2='1420304_x_at',CellID2='',rank='1')) | [0.451 38](javascript:showCorrelationPlot2(db='MA_M2M_0706_R',ProbeSetID='1417426_at',CellID='',db2='MA_M2M_0706_R',ProbeSetID2='1416500_at',CellID2='',rank='1')) | [-0.369 38](javascript:showCorrelationPlot2(db='MA_M2M_0706_R',ProbeSetID='1417426_at',CellID='',db2='MA_M2M_0706_R',ProbeSetID2='1424199_at',CellID2='',rank='1')) | [0.550 38](javascript:showCorrelationPlot2(db='MA_M2M_0706_R',ProbeSetID='1417426_at',CellID='',db2='MA_M2M_0706_R',ProbeSetID2='1437995_x_at',CellID2='',rank='1')) | [0.560 38](javascript:showCorrelationPlot2(db='MA_M2M_0706_R',ProbeSetID='1417426_at',CellID='',db2='MA_M2M_0706_R',ProbeSetID2='1427914_a_at',CellID2='',rank='1')) | [-0.288 38](javascript:showCorrelationPlot2(db='MA_M2M_0706_R',ProbeSetID='1417426_at',CellID='',db2='MA_M2M_0706_R',ProbeSetID2='1420570_x_at',CellID2='',rank='1')) | [-0.394 38](javascript:showCorrelationPlot2(db='MA_M2M_0706_R',ProbeSetID='1417426_at',CellID='',db2='MA_M2M_0706_R',ProbeSetID2='1431015_at',CellID2='',rank='1')) | [0.323 38](javascript:showCorrelationPlot2(db='MA_M2M_0706_R',ProbeSetID='1417426_at',CellID='',db2='MA_M2M_0706_R',ProbeSetID2='1423852_at',CellID2='',rank='1')) | [-0.479 38](javascript:showCorrelationPlot2(db='MA_M2M_0706_R',ProbeSetID='1417426_at',CellID='',db2='MA_M2M_0706_R',ProbeSetID2='1431799_at',CellID2='',rank='1')) | [0.402 38](javascript:showCorrelationPlot2(db='MA_M2M_0706_R',ProbeSetID='1417426_at',CellID='',db2='MA_M2M_0706_R',ProbeSetID2='1423898_a_at',CellID2='',rank='1')) | [-0.518 38](javascript:showCorrelationPlot2(db='MA_M2M_0706_R',ProbeSetID='1417426_at',CellID='',db2='MA_M2M_0706_R',ProbeSetID2='1438579_at',CellID2='',rank='1')) | [0.383 38](javascript:showCorrelationPlot2(db='MA_M2M_0706_R',ProbeSetID='1417426_at',CellID='',db2='MA_M2M_0706_R',ProbeSetID2='1448102_a_at',CellID2='',rank='1')) | [-0.360 38](javascript:showCorrelationPlot2(db='MA_M2M_0706_R',ProbeSetID='1417426_at',CellID='',db2='MA_M2M_0706_R',ProbeSetID2='1420943_at',CellID2='',rank='1')) | [-0.267 38](javascript:showCorrelationPlot2(db='MA_M2M_0706_R',ProbeSetID='1417426_at',CellID='',db2='MA_M2M_0706_R',ProbeSetID2='1447326_s_at',CellID2='',rank='1')) |
|  | [Trait 35: MA_M2M_0706_R::1416588_at](javascript:showDatabase2('MA_M2M_0706_R','1416588_at','');)  Ptprn on Chr 1 @ 75.243778 Mb  protein tyrosine phosphatase, receptor type, N  last exon and 3' UTR | [-0.681 38](javascript:showCorrelationPlot2(db='MA_M2M_0706_R',ProbeSetID='1416588_at',CellID='',db2='MA_M2M_0706_R',ProbeSetID2='1445101_at',CellID2='',rank='0')) | [0.484 38](javascript:showCorrelationPlot2(db='MA_M2M_0706_R',ProbeSetID='1416588_at',CellID='',db2='MA_M2M_0706_R',ProbeSetID2='1451285_at',CellID2='',rank='0')) | [0.659 38](javascript:showCorrelationPlot2(db='MA_M2M_0706_R',ProbeSetID='1416588_at',CellID='',db2='MA_M2M_0706_R',ProbeSetID2='1441347_at',CellID2='',rank='0')) | [-0.618 38](javascript:showCorrelationPlot2(db='MA_M2M_0706_R',ProbeSetID='1416588_at',CellID='',db2='MA_M2M_0706_R',ProbeSetID2='1426900_at',CellID2='',rank='0')) | [-0.515 38](javascript:showCorrelationPlot2(db='MA_M2M_0706_R',ProbeSetID='1416588_at',CellID='',db2='MA_M2M_0706_R',ProbeSetID2='1434888_a_at',CellID2='',rank='0')) | [-0.607 38](javascript:showCorrelationPlot2(db='MA_M2M_0706_R',ProbeSetID='1416588_at',CellID='',db2='MA_M2M_0706_R',ProbeSetID2='1434773_a_at',CellID2='',rank='0')) | [0.660 38](javascript:showCorrelationPlot2(db='MA_M2M_0706_R',ProbeSetID='1416588_at',CellID='',db2='MA_M2M_0706_R',ProbeSetID2='1452851_at',CellID2='',rank='0')) | [-0.595 38](javascript:showCorrelationPlot2(db='MA_M2M_0706_R',ProbeSetID='1416588_at',CellID='',db2='MA_M2M_0706_R',ProbeSetID2='1441948_x_at',CellID2='',rank='0')) | [0.761 38](javascript:showCorrelationPlot2(db='MA_M2M_0706_R',ProbeSetID='1416588_at',CellID='',db2='MA_M2M_0706_R',ProbeSetID2='1440160_x_at',CellID2='',rank='0')) | [-0.707 38](javascript:showCorrelationPlot2(db='MA_M2M_0706_R',ProbeSetID='1416588_at',CellID='',db2='MA_M2M_0706_R',ProbeSetID2='1424692_at',CellID2='',rank='0')) | [0.680 38](javascript:showCorrelationPlot2(db='MA_M2M_0706_R',ProbeSetID='1416588_at',CellID='',db2='MA_M2M_0706_R',ProbeSetID2='1433112_at',CellID2='',rank='0')) | [-0.506 38](javascript:showCorrelationPlot2(db='MA_M2M_0706_R',ProbeSetID='1416588_at',CellID='',db2='MA_M2M_0706_R',ProbeSetID2='1459962_at',CellID2='',rank='0')) | [0.714 38](javascript:showCorrelationPlot2(db='MA_M2M_0706_R',ProbeSetID='1416588_at',CellID='',db2='MA_M2M_0706_R',ProbeSetID2='1455331_at',CellID2='',rank='0')) | [0.638 38](javascript:showCorrelationPlot2(db='MA_M2M_0706_R',ProbeSetID='1416588_at',CellID='',db2='MA_M2M_0706_R',ProbeSetID2='1447399_at',CellID2='',rank='0')) | [-0.627 38](javascript:showCorrelationPlot2(db='MA_M2M_0706_R',ProbeSetID='1416588_at',CellID='',db2='MA_M2M_0706_R',ProbeSetID2='1434968_a_at',CellID2='',rank='0')) | [0.686 38](javascript:showCorrelationPlot2(db='MA_M2M_0706_R',ProbeSetID='1416588_at',CellID='',db2='MA_M2M_0706_R',ProbeSetID2='1447335_x_at',CellID2='',rank='0')) | [0.732 38](javascript:showCorrelationPlot2(db='MA_M2M_0706_R',ProbeSetID='1416588_at',CellID='',db2='MA_M2M_0706_R',ProbeSetID2='1442239_at',CellID2='',rank='0')) | [0.602 38](javascript:showCorrelationPlot2(db='MA_M2M_0706_R',ProbeSetID='1416588_at',CellID='',db2='MA_M2M_0706_R',ProbeSetID2='1459865_x_at',CellID2='',rank='0')) | [-0.485 38](javascript:showCorrelationPlot2(db='MA_M2M_0706_R',ProbeSetID='1416588_at',CellID='',db2='MA_M2M_0706_R',ProbeSetID2='1438647_x_at',CellID2='',rank='0')) | [0.612 38](javascript:showCorrelationPlot2(db='MA_M2M_0706_R',ProbeSetID='1416588_at',CellID='',db2='MA_M2M_0706_R',ProbeSetID2='1432333_a_at',CellID2='',rank='0')) | [-0.594 38](javascript:showCorrelationPlot2(db='MA_M2M_0706_R',ProbeSetID='1416588_at',CellID='',db2='MA_M2M_0706_R',ProbeSetID2='1444028_s_at',CellID2='',rank='0')) | [0.661 38](javascript:showCorrelationPlot2(db='MA_M2M_0706_R',ProbeSetID='1416588_at',CellID='',db2='MA_M2M_0706_R',ProbeSetID2='1460420_a_at',CellID2='',rank='0')) | [0.663 38](javascript:showCorrelationPlot2(db='MA_M2M_0706_R',ProbeSetID='1416588_at',CellID='',db2='MA_M2M_0706_R',ProbeSetID2='1451530_at',CellID2='',rank='0')) | [-0.610 38](javascript:showCorrelationPlot2(db='MA_M2M_0706_R',ProbeSetID='1416588_at',CellID='',db2='MA_M2M_0706_R',ProbeSetID2='1423785_at',CellID2='',rank='0')) | [0.377 38](javascript:showCorrelationPlot2(db='MA_M2M_0706_R',ProbeSetID='1416588_at',CellID='',db2='MA_M2M_0706_R',ProbeSetID2='1431972_a_at',CellID2='',rank='0')) | [-0.644 38](javascript:showCorrelationPlot2(db='MA_M2M_0706_R',ProbeSetID='1416588_at',CellID='',db2='MA_M2M_0706_R',ProbeSetID2='1448347_a_at',CellID2='',rank='0')) | [0.522 38](javascript:showCorrelationPlot2(db='MA_M2M_0706_R',ProbeSetID='1416588_at',CellID='',db2='MA_M2M_0706_R',ProbeSetID2='1418492_at',CellID2='',rank='0')) | [-0.568 38](javascript:showCorrelationPlot2(db='MA_M2M_0706_R',ProbeSetID='1416588_at',CellID='',db2='MA_M2M_0706_R',ProbeSetID2='1427185_at',CellID2='',rank='0')) | [-0.518 38](javascript:showCorrelationPlot2(db='MA_M2M_0706_R',ProbeSetID='1416588_at',CellID='',db2='MA_M2M_0706_R',ProbeSetID2='1430500_s_at',CellID2='',rank='0')) | [0.562 38](javascript:showCorrelationPlot2(db='MA_M2M_0706_R',ProbeSetID='1416588_at',CellID='',db2='MA_M2M_0706_R',ProbeSetID2='1447549_x_at',CellID2='',rank='0')) | [-0.484 38](javascript:showCorrelationPlot2(db='MA_M2M_0706_R',ProbeSetID='1416588_at',CellID='',db2='MA_M2M_0706_R',ProbeSetID2='1448943_at',CellID2='',rank='0')) | [0.840 38](javascript:showCorrelationPlot2(db='MA_M2M_0706_R',ProbeSetID='1416588_at',CellID='',db2='MA_M2M_0706_R',ProbeSetID2='1433162_at',CellID2='',rank='0')) | [0.609 38](javascript:showCorrelationPlot2(db='MA_M2M_0706_R',ProbeSetID='1416588_at',CellID='',db2='MA_M2M_0706_R',ProbeSetID2='1443494_at',CellID2='',rank='0')) | [-0.418 38](javascript:showCorrelationPlot2(db='MA_M2M_0706_R',ProbeSetID='1416588_at',CellID='',db2='MA_M2M_0706_R',ProbeSetID2='1417426_at',CellID2='',rank='0')) | [n 38](javascript:showDatabase2('MA_M2M_0706_R','1416588_at','')) | [-0.597 38](javascript:showCorrelationPlot2(db='MA_M2M_0706_R',ProbeSetID='1416588_at',CellID='',db2='MA_M2M_0706_R',ProbeSetID2='1450994_at',CellID2='',rank='1')) | [0.688 38](javascript:showCorrelationPlot2(db='MA_M2M_0706_R',ProbeSetID='1416588_at',CellID='',db2='MA_M2M_0706_R',ProbeSetID2='1420304_x_at',CellID2='',rank='1')) | [-0.529 38](javascript:showCorrelationPlot2(db='MA_M2M_0706_R',ProbeSetID='1416588_at',CellID='',db2='MA_M2M_0706_R',ProbeSetID2='1416500_at',CellID2='',rank='1')) | [0.658 38](javascript:showCorrelationPlot2(db='MA_M2M_0706_R',ProbeSetID='1416588_at',CellID='',db2='MA_M2M_0706_R',ProbeSetID2='1424199_at',CellID2='',rank='1')) | [-0.543 38](javascript:showCorrelationPlot2(db='MA_M2M_0706_R',ProbeSetID='1416588_at',CellID='',db2='MA_M2M_0706_R',ProbeSetID2='1437995_x_at',CellID2='',rank='1')) | [-0.561 38](javascript:showCorrelationPlot2(db='MA_M2M_0706_R',ProbeSetID='1416588_at',CellID='',db2='MA_M2M_0706_R',ProbeSetID2='1427914_a_at',CellID2='',rank='1')) | [0.631 38](javascript:showCorrelationPlot2(db='MA_M2M_0706_R',ProbeSetID='1416588_at',CellID='',db2='MA_M2M_0706_R',ProbeSetID2='1420570_x_at',CellID2='',rank='1')) | [0.656 38](javascript:showCorrelationPlot2(db='MA_M2M_0706_R',ProbeSetID='1416588_at',CellID='',db2='MA_M2M_0706_R',ProbeSetID2='1431015_at',CellID2='',rank='1')) | [-0.509 38](javascript:showCorrelationPlot2(db='MA_M2M_0706_R',ProbeSetID='1416588_at',CellID='',db2='MA_M2M_0706_R',ProbeSetID2='1423852_at',CellID2='',rank='1')) | [0.456 38](javascript:showCorrelationPlot2(db='MA_M2M_0706_R',ProbeSetID='1416588_at',CellID='',db2='MA_M2M_0706_R',ProbeSetID2='1431799_at',CellID2='',rank='1')) | [-0.685 38](javascript:showCorrelationPlot2(db='MA_M2M_0706_R',ProbeSetID='1416588_at',CellID='',db2='MA_M2M_0706_R',ProbeSetID2='1423898_a_at',CellID2='',rank='1')) | [0.636 38](javascript:showCorrelationPlot2(db='MA_M2M_0706_R',ProbeSetID='1416588_at',CellID='',db2='MA_M2M_0706_R',ProbeSetID2='1438579_at',CellID2='',rank='1')) | [-0.659 38](javascript:showCorrelationPlot2(db='MA_M2M_0706_R',ProbeSetID='1416588_at',CellID='',db2='MA_M2M_0706_R',ProbeSetID2='1448102_a_at',CellID2='',rank='1')) | [0.493 38](javascript:showCorrelationPlot2(db='MA_M2M_0706_R',ProbeSetID='1416588_at',CellID='',db2='MA_M2M_0706_R',ProbeSetID2='1420943_at',CellID2='',rank='1')) | [0.585 38](javascript:showCorrelationPlot2(db='MA_M2M_0706_R',ProbeSetID='1416588_at',CellID='',db2='MA_M2M_0706_R',ProbeSetID2='1447326_s_at',CellID2='',rank='1')) |
|  | [Trait 36: MA_M2M_0706_R::1450994_at](javascript:showDatabase2('MA_M2M_0706_R','1450994_at','');)  Rock1 on Chr 18 @ 10.064493 Mb  Rho-associated coiled-coil forming kinase 1 | [0.320 38](javascript:showCorrelationPlot2(db='MA_M2M_0706_R',ProbeSetID='1450994_at',CellID='',db2='MA_M2M_0706_R',ProbeSetID2='1445101_at',CellID2='',rank='0')) | [-0.465 38](javascript:showCorrelationPlot2(db='MA_M2M_0706_R',ProbeSetID='1450994_at',CellID='',db2='MA_M2M_0706_R',ProbeSetID2='1451285_at',CellID2='',rank='0')) | [-0.312 38](javascript:showCorrelationPlot2(db='MA_M2M_0706_R',ProbeSetID='1450994_at',CellID='',db2='MA_M2M_0706_R',ProbeSetID2='1441347_at',CellID2='',rank='0')) | [0.628 38](javascript:showCorrelationPlot2(db='MA_M2M_0706_R',ProbeSetID='1450994_at',CellID='',db2='MA_M2M_0706_R',ProbeSetID2='1426900_at',CellID2='',rank='0')) | [0.552 38](javascript:showCorrelationPlot2(db='MA_M2M_0706_R',ProbeSetID='1450994_at',CellID='',db2='MA_M2M_0706_R',ProbeSetID2='1434888_a_at',CellID2='',rank='0')) | [0.645 38](javascript:showCorrelationPlot2(db='MA_M2M_0706_R',ProbeSetID='1450994_at',CellID='',db2='MA_M2M_0706_R',ProbeSetID2='1434773_a_at',CellID2='',rank='0')) | [-0.460 38](javascript:showCorrelationPlot2(db='MA_M2M_0706_R',ProbeSetID='1450994_at',CellID='',db2='MA_M2M_0706_R',ProbeSetID2='1452851_at',CellID2='',rank='0')) | [0.349 38](javascript:showCorrelationPlot2(db='MA_M2M_0706_R',ProbeSetID='1450994_at',CellID='',db2='MA_M2M_0706_R',ProbeSetID2='1441948_x_at',CellID2='',rank='0')) | [-0.411 38](javascript:showCorrelationPlot2(db='MA_M2M_0706_R',ProbeSetID='1450994_at',CellID='',db2='MA_M2M_0706_R',ProbeSetID2='1440160_x_at',CellID2='',rank='0')) | [0.453 38](javascript:showCorrelationPlot2(db='MA_M2M_0706_R',ProbeSetID='1450994_at',CellID='',db2='MA_M2M_0706_R',ProbeSetID2='1424692_at',CellID2='',rank='0')) | [-0.465 38](javascript:showCorrelationPlot2(db='MA_M2M_0706_R',ProbeSetID='1450994_at',CellID='',db2='MA_M2M_0706_R',ProbeSetID2='1433112_at',CellID2='',rank='0')) | [0.520 38](javascript:showCorrelationPlot2(db='MA_M2M_0706_R',ProbeSetID='1450994_at',CellID='',db2='MA_M2M_0706_R',ProbeSetID2='1459962_at',CellID2='',rank='0')) | [-0.363 38](javascript:showCorrelationPlot2(db='MA_M2M_0706_R',ProbeSetID='1450994_at',CellID='',db2='MA_M2M_0706_R',ProbeSetID2='1455331_at',CellID2='',rank='0')) | [-0.445 38](javascript:showCorrelationPlot2(db='MA_M2M_0706_R',ProbeSetID='1450994_at',CellID='',db2='MA_M2M_0706_R',ProbeSetID2='1447399_at',CellID2='',rank='0')) | [0.312 38](javascript:showCorrelationPlot2(db='MA_M2M_0706_R',ProbeSetID='1450994_at',CellID='',db2='MA_M2M_0706_R',ProbeSetID2='1434968_a_at',CellID2='',rank='0')) | [-0.486 38](javascript:showCorrelationPlot2(db='MA_M2M_0706_R',ProbeSetID='1450994_at',CellID='',db2='MA_M2M_0706_R',ProbeSetID2='1447335_x_at',CellID2='',rank='0')) | [-0.382 38](javascript:showCorrelationPlot2(db='MA_M2M_0706_R',ProbeSetID='1450994_at',CellID='',db2='MA_M2M_0706_R',ProbeSetID2='1442239_at',CellID2='',rank='0')) | [-0.689 38](javascript:showCorrelationPlot2(db='MA_M2M_0706_R',ProbeSetID='1450994_at',CellID='',db2='MA_M2M_0706_R',ProbeSetID2='1459865_x_at',CellID2='',rank='0')) | [0.453 38](javascript:showCorrelationPlot2(db='MA_M2M_0706_R',ProbeSetID='1450994_at',CellID='',db2='MA_M2M_0706_R',ProbeSetID2='1438647_x_at',CellID2='',rank='0')) | [-0.368 38](javascript:showCorrelationPlot2(db='MA_M2M_0706_R',ProbeSetID='1450994_at',CellID='',db2='MA_M2M_0706_R',ProbeSetID2='1432333_a_at',CellID2='',rank='0')) | [0.635 38](javascript:showCorrelationPlot2(db='MA_M2M_0706_R',ProbeSetID='1450994_at',CellID='',db2='MA_M2M_0706_R',ProbeSetID2='1444028_s_at',CellID2='',rank='0')) | [-0.689 38](javascript:showCorrelationPlot2(db='MA_M2M_0706_R',ProbeSetID='1450994_at',CellID='',db2='MA_M2M_0706_R',ProbeSetID2='1460420_a_at',CellID2='',rank='0')) | [-0.480 38](javascript:showCorrelationPlot2(db='MA_M2M_0706_R',ProbeSetID='1450994_at',CellID='',db2='MA_M2M_0706_R',ProbeSetID2='1451530_at',CellID2='',rank='0')) | [0.441 38](javascript:showCorrelationPlot2(db='MA_M2M_0706_R',ProbeSetID='1450994_at',CellID='',db2='MA_M2M_0706_R',ProbeSetID2='1423785_at',CellID2='',rank='0')) | [-0.448 38](javascript:showCorrelationPlot2(db='MA_M2M_0706_R',ProbeSetID='1450994_at',CellID='',db2='MA_M2M_0706_R',ProbeSetID2='1431972_a_at',CellID2='',rank='0')) | [0.575 38](javascript:showCorrelationPlot2(db='MA_M2M_0706_R',ProbeSetID='1450994_at',CellID='',db2='MA_M2M_0706_R',ProbeSetID2='1448347_a_at',CellID2='',rank='0')) | [-0.420 38](javascript:showCorrelationPlot2(db='MA_M2M_0706_R',ProbeSetID='1450994_at',CellID='',db2='MA_M2M_0706_R',ProbeSetID2='1418492_at',CellID2='',rank='0')) | [0.563 38](javascript:showCorrelationPlot2(db='MA_M2M_0706_R',ProbeSetID='1450994_at',CellID='',db2='MA_M2M_0706_R',ProbeSetID2='1427185_at',CellID2='',rank='0')) | [0.546 38](javascript:showCorrelationPlot2(db='MA_M2M_0706_R',ProbeSetID='1450994_at',CellID='',db2='MA_M2M_0706_R',ProbeSetID2='1430500_s_at',CellID2='',rank='0')) | [-0.579 38](javascript:showCorrelationPlot2(db='MA_M2M_0706_R',ProbeSetID='1450994_at',CellID='',db2='MA_M2M_0706_R',ProbeSetID2='1447549_x_at',CellID2='',rank='0')) | [0.574 38](javascript:showCorrelationPlot2(db='MA_M2M_0706_R',ProbeSetID='1450994_at',CellID='',db2='MA_M2M_0706_R',ProbeSetID2='1448943_at',CellID2='',rank='0')) | [-0.450 38](javascript:showCorrelationPlot2(db='MA_M2M_0706_R',ProbeSetID='1450994_at',CellID='',db2='MA_M2M_0706_R',ProbeSetID2='1433162_at',CellID2='',rank='0')) | [-0.456 38](javascript:showCorrelationPlot2(db='MA_M2M_0706_R',ProbeSetID='1450994_at',CellID='',db2='MA_M2M_0706_R',ProbeSetID2='1443494_at',CellID2='',rank='0')) | [0.644 38](javascript:showCorrelationPlot2(db='MA_M2M_0706_R',ProbeSetID='1450994_at',CellID='',db2='MA_M2M_0706_R',ProbeSetID2='1417426_at',CellID2='',rank='0')) | [-0.503 38](javascript:showCorrelationPlot2(db='MA_M2M_0706_R',ProbeSetID='1450994_at',CellID='',db2='MA_M2M_0706_R',ProbeSetID2='1416588_at',CellID2='',rank='0')) | [n 38](javascript:showDatabase2('MA_M2M_0706_R','1450994_at','')) | [-0.568 38](javascript:showCorrelationPlot2(db='MA_M2M_0706_R',ProbeSetID='1450994_at',CellID='',db2='MA_M2M_0706_R',ProbeSetID2='1420304_x_at',CellID2='',rank='1')) | [0.427 38](javascript:showCorrelationPlot2(db='MA_M2M_0706_R',ProbeSetID='1450994_at',CellID='',db2='MA_M2M_0706_R',ProbeSetID2='1416500_at',CellID2='',rank='1')) | [-0.496 38](javascript:showCorrelationPlot2(db='MA_M2M_0706_R',ProbeSetID='1450994_at',CellID='',db2='MA_M2M_0706_R',ProbeSetID2='1424199_at',CellID2='',rank='1')) | [0.523 38](javascript:showCorrelationPlot2(db='MA_M2M_0706_R',ProbeSetID='1450994_at',CellID='',db2='MA_M2M_0706_R',ProbeSetID2='1437995_x_at',CellID2='',rank='1')) | [0.701 38](javascript:showCorrelationPlot2(db='MA_M2M_0706_R',ProbeSetID='1450994_at',CellID='',db2='MA_M2M_0706_R',ProbeSetID2='1427914_a_at',CellID2='',rank='1')) | [-0.518 38](javascript:showCorrelationPlot2(db='MA_M2M_0706_R',ProbeSetID='1450994_at',CellID='',db2='MA_M2M_0706_R',ProbeSetID2='1420570_x_at',CellID2='',rank='1')) | [-0.434 38](javascript:showCorrelationPlot2(db='MA_M2M_0706_R',ProbeSetID='1450994_at',CellID='',db2='MA_M2M_0706_R',ProbeSetID2='1431015_at',CellID2='',rank='1')) | [0.315 38](javascript:showCorrelationPlot2(db='MA_M2M_0706_R',ProbeSetID='1450994_at',CellID='',db2='MA_M2M_0706_R',ProbeSetID2='1423852_at',CellID2='',rank='1')) | [-0.519 38](javascript:showCorrelationPlot2(db='MA_M2M_0706_R',ProbeSetID='1450994_at',CellID='',db2='MA_M2M_0706_R',ProbeSetID2='1431799_at',CellID2='',rank='1')) | [0.559 38](javascript:showCorrelationPlot2(db='MA_M2M_0706_R',ProbeSetID='1450994_at',CellID='',db2='MA_M2M_0706_R',ProbeSetID2='1423898_a_at',CellID2='',rank='1')) | [-0.422 38](javascript:showCorrelationPlot2(db='MA_M2M_0706_R',ProbeSetID='1450994_at',CellID='',db2='MA_M2M_0706_R',ProbeSetID2='1438579_at',CellID2='',rank='1')) | [0.362 38](javascript:showCorrelationPlot2(db='MA_M2M_0706_R',ProbeSetID='1450994_at',CellID='',db2='MA_M2M_0706_R',ProbeSetID2='1448102_a_at',CellID2='',rank='1')) | [-0.239 38](javascript:showCorrelationPlot2(db='MA_M2M_0706_R',ProbeSetID='1450994_at',CellID='',db2='MA_M2M_0706_R',ProbeSetID2='1420943_at',CellID2='',rank='1')) | [-0.461 38](javascript:showCorrelationPlot2(db='MA_M2M_0706_R',ProbeSetID='1450994_at',CellID='',db2='MA_M2M_0706_R',ProbeSetID2='1447326_s_at',CellID2='',rank='1')) |
|  | [Trait 37: MA_M2M_0706_R::1420304_x_at](javascript:showDatabase2('MA_M2M_0706_R','1420304_x_at','');)  Rps4x on Chr X @ 99.380287 Mb  ribosomal protein S4, X-linked  poor probe set specificity in 3' UTR | [-0.525 38](javascript:showCorrelationPlot2(db='MA_M2M_0706_R',ProbeSetID='1420304_x_at',CellID='',db2='MA_M2M_0706_R',ProbeSetID2='1445101_at',CellID2='',rank='0')) | [0.438 38](javascript:showCorrelationPlot2(db='MA_M2M_0706_R',ProbeSetID='1420304_x_at',CellID='',db2='MA_M2M_0706_R',ProbeSetID2='1451285_at',CellID2='',rank='0')) | [0.662 38](javascript:showCorrelationPlot2(db='MA_M2M_0706_R',ProbeSetID='1420304_x_at',CellID='',db2='MA_M2M_0706_R',ProbeSetID2='1441347_at',CellID2='',rank='0')) | [-0.518 38](javascript:showCorrelationPlot2(db='MA_M2M_0706_R',ProbeSetID='1420304_x_at',CellID='',db2='MA_M2M_0706_R',ProbeSetID2='1426900_at',CellID2='',rank='0')) | [-0.553 38](javascript:showCorrelationPlot2(db='MA_M2M_0706_R',ProbeSetID='1420304_x_at',CellID='',db2='MA_M2M_0706_R',ProbeSetID2='1434888_a_at',CellID2='',rank='0')) | [-0.687 38](javascript:showCorrelationPlot2(db='MA_M2M_0706_R',ProbeSetID='1420304_x_at',CellID='',db2='MA_M2M_0706_R',ProbeSetID2='1434773_a_at',CellID2='',rank='0')) | [0.543 38](javascript:showCorrelationPlot2(db='MA_M2M_0706_R',ProbeSetID='1420304_x_at',CellID='',db2='MA_M2M_0706_R',ProbeSetID2='1452851_at',CellID2='',rank='0')) | [-0.580 38](javascript:showCorrelationPlot2(db='MA_M2M_0706_R',ProbeSetID='1420304_x_at',CellID='',db2='MA_M2M_0706_R',ProbeSetID2='1441948_x_at',CellID2='',rank='0')) | [0.577 38](javascript:showCorrelationPlot2(db='MA_M2M_0706_R',ProbeSetID='1420304_x_at',CellID='',db2='MA_M2M_0706_R',ProbeSetID2='1440160_x_at',CellID2='',rank='0')) | [-0.486 38](javascript:showCorrelationPlot2(db='MA_M2M_0706_R',ProbeSetID='1420304_x_at',CellID='',db2='MA_M2M_0706_R',ProbeSetID2='1424692_at',CellID2='',rank='0')) | [0.753 38](javascript:showCorrelationPlot2(db='MA_M2M_0706_R',ProbeSetID='1420304_x_at',CellID='',db2='MA_M2M_0706_R',ProbeSetID2='1433112_at',CellID2='',rank='0')) | [-0.698 38](javascript:showCorrelationPlot2(db='MA_M2M_0706_R',ProbeSetID='1420304_x_at',CellID='',db2='MA_M2M_0706_R',ProbeSetID2='1459962_at',CellID2='',rank='0')) | [0.566 38](javascript:showCorrelationPlot2(db='MA_M2M_0706_R',ProbeSetID='1420304_x_at',CellID='',db2='MA_M2M_0706_R',ProbeSetID2='1455331_at',CellID2='',rank='0')) | [0.679 38](javascript:showCorrelationPlot2(db='MA_M2M_0706_R',ProbeSetID='1420304_x_at',CellID='',db2='MA_M2M_0706_R',ProbeSetID2='1447399_at',CellID2='',rank='0')) | [-0.495 38](javascript:showCorrelationPlot2(db='MA_M2M_0706_R',ProbeSetID='1420304_x_at',CellID='',db2='MA_M2M_0706_R',ProbeSetID2='1434968_a_at',CellID2='',rank='0')) | [0.610 38](javascript:showCorrelationPlot2(db='MA_M2M_0706_R',ProbeSetID='1420304_x_at',CellID='',db2='MA_M2M_0706_R',ProbeSetID2='1447335_x_at',CellID2='',rank='0')) | [0.648 38](javascript:showCorrelationPlot2(db='MA_M2M_0706_R',ProbeSetID='1420304_x_at',CellID='',db2='MA_M2M_0706_R',ProbeSetID2='1442239_at',CellID2='',rank='0')) | [0.508 38](javascript:showCorrelationPlot2(db='MA_M2M_0706_R',ProbeSetID='1420304_x_at',CellID='',db2='MA_M2M_0706_R',ProbeSetID2='1459865_x_at',CellID2='',rank='0')) | [-0.565 38](javascript:showCorrelationPlot2(db='MA_M2M_0706_R',ProbeSetID='1420304_x_at',CellID='',db2='MA_M2M_0706_R',ProbeSetID2='1438647_x_at',CellID2='',rank='0')) | [0.485 38](javascript:showCorrelationPlot2(db='MA_M2M_0706_R',ProbeSetID='1420304_x_at',CellID='',db2='MA_M2M_0706_R',ProbeSetID2='1432333_a_at',CellID2='',rank='0')) | [-0.676 38](javascript:showCorrelationPlot2(db='MA_M2M_0706_R',ProbeSetID='1420304_x_at',CellID='',db2='MA_M2M_0706_R',ProbeSetID2='1444028_s_at',CellID2='',rank='0')) | [0.699 38](javascript:showCorrelationPlot2(db='MA_M2M_0706_R',ProbeSetID='1420304_x_at',CellID='',db2='MA_M2M_0706_R',ProbeSetID2='1460420_a_at',CellID2='',rank='0')) | [0.773 38](javascript:showCorrelationPlot2(db='MA_M2M_0706_R',ProbeSetID='1420304_x_at',CellID='',db2='MA_M2M_0706_R',ProbeSetID2='1451530_at',CellID2='',rank='0')) | [-0.500 38](javascript:showCorrelationPlot2(db='MA_M2M_0706_R',ProbeSetID='1420304_x_at',CellID='',db2='MA_M2M_0706_R',ProbeSetID2='1423785_at',CellID2='',rank='0')) | [0.489 38](javascript:showCorrelationPlot2(db='MA_M2M_0706_R',ProbeSetID='1420304_x_at',CellID='',db2='MA_M2M_0706_R',ProbeSetID2='1431972_a_at',CellID2='',rank='0')) | [-0.598 38](javascript:showCorrelationPlot2(db='MA_M2M_0706_R',ProbeSetID='1420304_x_at',CellID='',db2='MA_M2M_0706_R',ProbeSetID2='1448347_a_at',CellID2='',rank='0')) | [0.570 38](javascript:showCorrelationPlot2(db='MA_M2M_0706_R',ProbeSetID='1420304_x_at',CellID='',db2='MA_M2M_0706_R',ProbeSetID2='1418492_at',CellID2='',rank='0')) | [-0.516 38](javascript:showCorrelationPlot2(db='MA_M2M_0706_R',ProbeSetID='1420304_x_at',CellID='',db2='MA_M2M_0706_R',ProbeSetID2='1427185_at',CellID2='',rank='0')) | [-0.591 38](javascript:showCorrelationPlot2(db='MA_M2M_0706_R',ProbeSetID='1420304_x_at',CellID='',db2='MA_M2M_0706_R',ProbeSetID2='1430500_s_at',CellID2='',rank='0')) | [0.661 38](javascript:showCorrelationPlot2(db='MA_M2M_0706_R',ProbeSetID='1420304_x_at',CellID='',db2='MA_M2M_0706_R',ProbeSetID2='1447549_x_at',CellID2='',rank='0')) | [-0.484 38](javascript:showCorrelationPlot2(db='MA_M2M_0706_R',ProbeSetID='1420304_x_at',CellID='',db2='MA_M2M_0706_R',ProbeSetID2='1448943_at',CellID2='',rank='0')) | [0.698 38](javascript:showCorrelationPlot2(db='MA_M2M_0706_R',ProbeSetID='1420304_x_at',CellID='',db2='MA_M2M_0706_R',ProbeSetID2='1433162_at',CellID2='',rank='0')) | [0.565 38](javascript:showCorrelationPlot2(db='MA_M2M_0706_R',ProbeSetID='1420304_x_at',CellID='',db2='MA_M2M_0706_R',ProbeSetID2='1443494_at',CellID2='',rank='0')) | [-0.485 38](javascript:showCorrelationPlot2(db='MA_M2M_0706_R',ProbeSetID='1420304_x_at',CellID='',db2='MA_M2M_0706_R',ProbeSetID2='1417426_at',CellID2='',rank='0')) | [0.723 38](javascript:showCorrelationPlot2(db='MA_M2M_0706_R',ProbeSetID='1420304_x_at',CellID='',db2='MA_M2M_0706_R',ProbeSetID2='1416588_at',CellID2='',rank='0')) | [-0.577 38](javascript:showCorrelationPlot2(db='MA_M2M_0706_R',ProbeSetID='1420304_x_at',CellID='',db2='MA_M2M_0706_R',ProbeSetID2='1450994_at',CellID2='',rank='0')) | [n 38](javascript:showDatabase2('MA_M2M_0706_R','1420304_x_at','')) | [-0.569 38](javascript:showCorrelationPlot2(db='MA_M2M_0706_R',ProbeSetID='1420304_x_at',CellID='',db2='MA_M2M_0706_R',ProbeSetID2='1416500_at',CellID2='',rank='1')) | [0.632 38](javascript:showCorrelationPlot2(db='MA_M2M_0706_R',ProbeSetID='1420304_x_at',CellID='',db2='MA_M2M_0706_R',ProbeSetID2='1424199_at',CellID2='',rank='1')) | [-0.589 38](javascript:showCorrelationPlot2(db='MA_M2M_0706_R',ProbeSetID='1420304_x_at',CellID='',db2='MA_M2M_0706_R',ProbeSetID2='1437995_x_at',CellID2='',rank='1')) | [-0.582 38](javascript:showCorrelationPlot2(db='MA_M2M_0706_R',ProbeSetID='1420304_x_at',CellID='',db2='MA_M2M_0706_R',ProbeSetID2='1427914_a_at',CellID2='',rank='1')) | [0.701 38](javascript:showCorrelationPlot2(db='MA_M2M_0706_R',ProbeSetID='1420304_x_at',CellID='',db2='MA_M2M_0706_R',ProbeSetID2='1420570_x_at',CellID2='',rank='1')) | [0.647 38](javascript:showCorrelationPlot2(db='MA_M2M_0706_R',ProbeSetID='1420304_x_at',CellID='',db2='MA_M2M_0706_R',ProbeSetID2='1431015_at',CellID2='',rank='1')) | [-0.630 38](javascript:showCorrelationPlot2(db='MA_M2M_0706_R',ProbeSetID='1420304_x_at',CellID='',db2='MA_M2M_0706_R',ProbeSetID2='1423852_at',CellID2='',rank='1')) | [0.392 38](javascript:showCorrelationPlot2(db='MA_M2M_0706_R',ProbeSetID='1420304_x_at',CellID='',db2='MA_M2M_0706_R',ProbeSetID2='1431799_at',CellID2='',rank='1')) | [-0.518 38](javascript:showCorrelationPlot2(db='MA_M2M_0706_R',ProbeSetID='1420304_x_at',CellID='',db2='MA_M2M_0706_R',ProbeSetID2='1423898_a_at',CellID2='',rank='1')) | [0.696 38](javascript:showCorrelationPlot2(db='MA_M2M_0706_R',ProbeSetID='1420304_x_at',CellID='',db2='MA_M2M_0706_R',ProbeSetID2='1438579_at',CellID2='',rank='1')) | [-0.766 38](javascript:showCorrelationPlot2(db='MA_M2M_0706_R',ProbeSetID='1420304_x_at',CellID='',db2='MA_M2M_0706_R',ProbeSetID2='1448102_a_at',CellID2='',rank='1')) | [0.705 38](javascript:showCorrelationPlot2(db='MA_M2M_0706_R',ProbeSetID='1420304_x_at',CellID='',db2='MA_M2M_0706_R',ProbeSetID2='1420943_at',CellID2='',rank='1')) | [0.714 38](javascript:showCorrelationPlot2(db='MA_M2M_0706_R',ProbeSetID='1420304_x_at',CellID='',db2='MA_M2M_0706_R',ProbeSetID2='1447326_s_at',CellID2='',rank='1')) |
|  | [Trait 38: MA_M2M_0706_R::1416500_at](javascript:showDatabase2('MA_M2M_0706_R','1416500_at','');)  Sacm1l on Chr 9 @ 123.501212 Mb  SAC1 (supressor of actin mutations 1, homolog)-like (S. cerevisiae) | [0.508 38](javascript:showCorrelationPlot2(db='MA_M2M_0706_R',ProbeSetID='1416500_at',CellID='',db2='MA_M2M_0706_R',ProbeSetID2='1445101_at',CellID2='',rank='0')) | [-0.530 38](javascript:showCorrelationPlot2(db='MA_M2M_0706_R',ProbeSetID='1416500_at',CellID='',db2='MA_M2M_0706_R',ProbeSetID2='1451285_at',CellID2='',rank='0')) | [-0.646 38](javascript:showCorrelationPlot2(db='MA_M2M_0706_R',ProbeSetID='1416500_at',CellID='',db2='MA_M2M_0706_R',ProbeSetID2='1441347_at',CellID2='',rank='0')) | [0.546 38](javascript:showCorrelationPlot2(db='MA_M2M_0706_R',ProbeSetID='1416500_at',CellID='',db2='MA_M2M_0706_R',ProbeSetID2='1426900_at',CellID2='',rank='0')) | [0.610 38](javascript:showCorrelationPlot2(db='MA_M2M_0706_R',ProbeSetID='1416500_at',CellID='',db2='MA_M2M_0706_R',ProbeSetID2='1434888_a_at',CellID2='',rank='0')) | [0.650 38](javascript:showCorrelationPlot2(db='MA_M2M_0706_R',ProbeSetID='1416500_at',CellID='',db2='MA_M2M_0706_R',ProbeSetID2='1434773_a_at',CellID2='',rank='0')) | [-0.676 38](javascript:showCorrelationPlot2(db='MA_M2M_0706_R',ProbeSetID='1416500_at',CellID='',db2='MA_M2M_0706_R',ProbeSetID2='1452851_at',CellID2='',rank='0')) | [0.628 38](javascript:showCorrelationPlot2(db='MA_M2M_0706_R',ProbeSetID='1416500_at',CellID='',db2='MA_M2M_0706_R',ProbeSetID2='1441948_x_at',CellID2='',rank='0')) | [-0.550 38](javascript:showCorrelationPlot2(db='MA_M2M_0706_R',ProbeSetID='1416500_at',CellID='',db2='MA_M2M_0706_R',ProbeSetID2='1440160_x_at',CellID2='',rank='0')) | [0.417 38](javascript:showCorrelationPlot2(db='MA_M2M_0706_R',ProbeSetID='1416500_at',CellID='',db2='MA_M2M_0706_R',ProbeSetID2='1424692_at',CellID2='',rank='0')) | [-0.557 38](javascript:showCorrelationPlot2(db='MA_M2M_0706_R',ProbeSetID='1416500_at',CellID='',db2='MA_M2M_0706_R',ProbeSetID2='1433112_at',CellID2='',rank='0')) | [0.528 38](javascript:showCorrelationPlot2(db='MA_M2M_0706_R',ProbeSetID='1416500_at',CellID='',db2='MA_M2M_0706_R',ProbeSetID2='1459962_at',CellID2='',rank='0')) | [-0.553 38](javascript:showCorrelationPlot2(db='MA_M2M_0706_R',ProbeSetID='1416500_at',CellID='',db2='MA_M2M_0706_R',ProbeSetID2='1455331_at',CellID2='',rank='0')) | [-0.529 38](javascript:showCorrelationPlot2(db='MA_M2M_0706_R',ProbeSetID='1416500_at',CellID='',db2='MA_M2M_0706_R',ProbeSetID2='1447399_at',CellID2='',rank='0')) | [0.339 38](javascript:showCorrelationPlot2(db='MA_M2M_0706_R',ProbeSetID='1416500_at',CellID='',db2='MA_M2M_0706_R',ProbeSetID2='1434968_a_at',CellID2='',rank='0')) | [-0.709 38](javascript:showCorrelationPlot2(db='MA_M2M_0706_R',ProbeSetID='1416500_at',CellID='',db2='MA_M2M_0706_R',ProbeSetID2='1447335_x_at',CellID2='',rank='0')) | [-0.580 38](javascript:showCorrelationPlot2(db='MA_M2M_0706_R',ProbeSetID='1416500_at',CellID='',db2='MA_M2M_0706_R',ProbeSetID2='1442239_at',CellID2='',rank='0')) | [-0.609 38](javascript:showCorrelationPlot2(db='MA_M2M_0706_R',ProbeSetID='1416500_at',CellID='',db2='MA_M2M_0706_R',ProbeSetID2='1459865_x_at',CellID2='',rank='0')) | [0.631 38](javascript:showCorrelationPlot2(db='MA_M2M_0706_R',ProbeSetID='1416500_at',CellID='',db2='MA_M2M_0706_R',ProbeSetID2='1438647_x_at',CellID2='',rank='0')) | [-0.645 38](javascript:showCorrelationPlot2(db='MA_M2M_0706_R',ProbeSetID='1416500_at',CellID='',db2='MA_M2M_0706_R',ProbeSetID2='1432333_a_at',CellID2='',rank='0')) | [0.431 38](javascript:showCorrelationPlot2(db='MA_M2M_0706_R',ProbeSetID='1416500_at',CellID='',db2='MA_M2M_0706_R',ProbeSetID2='1444028_s_at',CellID2='',rank='0')) | [-0.593 38](javascript:showCorrelationPlot2(db='MA_M2M_0706_R',ProbeSetID='1416500_at',CellID='',db2='MA_M2M_0706_R',ProbeSetID2='1460420_a_at',CellID2='',rank='0')) | [-0.585 38](javascript:showCorrelationPlot2(db='MA_M2M_0706_R',ProbeSetID='1416500_at',CellID='',db2='MA_M2M_0706_R',ProbeSetID2='1451530_at',CellID2='',rank='0')) | [0.502 38](javascript:showCorrelationPlot2(db='MA_M2M_0706_R',ProbeSetID='1416500_at',CellID='',db2='MA_M2M_0706_R',ProbeSetID2='1423785_at',CellID2='',rank='0')) | [-0.547 38](javascript:showCorrelationPlot2(db='MA_M2M_0706_R',ProbeSetID='1416500_at',CellID='',db2='MA_M2M_0706_R',ProbeSetID2='1431972_a_at',CellID2='',rank='0')) | [0.672 38](javascript:showCorrelationPlot2(db='MA_M2M_0706_R',ProbeSetID='1416500_at',CellID='',db2='MA_M2M_0706_R',ProbeSetID2='1448347_a_at',CellID2='',rank='0')) | [-0.564 38](javascript:showCorrelationPlot2(db='MA_M2M_0706_R',ProbeSetID='1416500_at',CellID='',db2='MA_M2M_0706_R',ProbeSetID2='1418492_at',CellID2='',rank='0')) | [0.566 38](javascript:showCorrelationPlot2(db='MA_M2M_0706_R',ProbeSetID='1416500_at',CellID='',db2='MA_M2M_0706_R',ProbeSetID2='1427185_at',CellID2='',rank='0')) | [0.610 38](javascript:showCorrelationPlot2(db='MA_M2M_0706_R',ProbeSetID='1416500_at',CellID='',db2='MA_M2M_0706_R',ProbeSetID2='1430500_s_at',CellID2='',rank='0')) | [-0.688 38](javascript:showCorrelationPlot2(db='MA_M2M_0706_R',ProbeSetID='1416500_at',CellID='',db2='MA_M2M_0706_R',ProbeSetID2='1447549_x_at',CellID2='',rank='0')) | [0.507 38](javascript:showCorrelationPlot2(db='MA_M2M_0706_R',ProbeSetID='1416500_at',CellID='',db2='MA_M2M_0706_R',ProbeSetID2='1448943_at',CellID2='',rank='0')) | [-0.535 38](javascript:showCorrelationPlot2(db='MA_M2M_0706_R',ProbeSetID='1416500_at',CellID='',db2='MA_M2M_0706_R',ProbeSetID2='1433162_at',CellID2='',rank='0')) | [-0.660 38](javascript:showCorrelationPlot2(db='MA_M2M_0706_R',ProbeSetID='1416500_at',CellID='',db2='MA_M2M_0706_R',ProbeSetID2='1443494_at',CellID2='',rank='0')) | [0.525 38](javascript:showCorrelationPlot2(db='MA_M2M_0706_R',ProbeSetID='1416500_at',CellID='',db2='MA_M2M_0706_R',ProbeSetID2='1417426_at',CellID2='',rank='0')) | [-0.558 38](javascript:showCorrelationPlot2(db='MA_M2M_0706_R',ProbeSetID='1416500_at',CellID='',db2='MA_M2M_0706_R',ProbeSetID2='1416588_at',CellID2='',rank='0')) | [0.491 38](javascript:showCorrelationPlot2(db='MA_M2M_0706_R',ProbeSetID='1416500_at',CellID='',db2='MA_M2M_0706_R',ProbeSetID2='1450994_at',CellID2='',rank='0')) | [-0.553 38](javascript:showCorrelationPlot2(db='MA_M2M_0706_R',ProbeSetID='1416500_at',CellID='',db2='MA_M2M_0706_R',ProbeSetID2='1420304_x_at',CellID2='',rank='0')) | [n 38](javascript:showDatabase2('MA_M2M_0706_R','1416500_at','')) | [-0.695 38](javascript:showCorrelationPlot2(db='MA_M2M_0706_R',ProbeSetID='1416500_at',CellID='',db2='MA_M2M_0706_R',ProbeSetID2='1424199_at',CellID2='',rank='1')) | [0.708 38](javascript:showCorrelationPlot2(db='MA_M2M_0706_R',ProbeSetID='1416500_at',CellID='',db2='MA_M2M_0706_R',ProbeSetID2='1437995_x_at',CellID2='',rank='1')) | [0.561 38](javascript:showCorrelationPlot2(db='MA_M2M_0706_R',ProbeSetID='1416500_at',CellID='',db2='MA_M2M_0706_R',ProbeSetID2='1427914_a_at',CellID2='',rank='1')) | [-0.502 38](javascript:showCorrelationPlot2(db='MA_M2M_0706_R',ProbeSetID='1416500_at',CellID='',db2='MA_M2M_0706_R',ProbeSetID2='1420570_x_at',CellID2='',rank='1')) | [-0.610 38](javascript:showCorrelationPlot2(db='MA_M2M_0706_R',ProbeSetID='1416500_at',CellID='',db2='MA_M2M_0706_R',ProbeSetID2='1431015_at',CellID2='',rank='1')) | [0.372 38](javascript:showCorrelationPlot2(db='MA_M2M_0706_R',ProbeSetID='1416500_at',CellID='',db2='MA_M2M_0706_R',ProbeSetID2='1423852_at',CellID2='',rank='1')) | [-0.288 38](javascript:showCorrelationPlot2(db='MA_M2M_0706_R',ProbeSetID='1416500_at',CellID='',db2='MA_M2M_0706_R',ProbeSetID2='1431799_at',CellID2='',rank='1')) | [0.681 38](javascript:showCorrelationPlot2(db='MA_M2M_0706_R',ProbeSetID='1416500_at',CellID='',db2='MA_M2M_0706_R',ProbeSetID2='1423898_a_at',CellID2='',rank='1')) | [-0.616 38](javascript:showCorrelationPlot2(db='MA_M2M_0706_R',ProbeSetID='1416500_at',CellID='',db2='MA_M2M_0706_R',ProbeSetID2='1438579_at',CellID2='',rank='1')) | [0.591 38](javascript:showCorrelationPlot2(db='MA_M2M_0706_R',ProbeSetID='1416500_at',CellID='',db2='MA_M2M_0706_R',ProbeSetID2='1448102_a_at',CellID2='',rank='1')) | [-0.464 38](javascript:showCorrelationPlot2(db='MA_M2M_0706_R',ProbeSetID='1416500_at',CellID='',db2='MA_M2M_0706_R',ProbeSetID2='1420943_at',CellID2='',rank='1')) | [-0.550 38](javascript:showCorrelationPlot2(db='MA_M2M_0706_R',ProbeSetID='1416500_at',CellID='',db2='MA_M2M_0706_R',ProbeSetID2='1447326_s_at',CellID2='',rank='1')) |
|  | [Trait 39: MA_M2M_0706_R::1424199_at](javascript:showDatabase2('MA_M2M_0706_R','1424199_at','');)  Seh1l on Chr 18 @ 67.954484 Mb  sec13-like nucleoporin protein (nup107-160 sub complex subunit) | [-0.705 38](javascript:showCorrelationPlot2(db='MA_M2M_0706_R',ProbeSetID='1424199_at',CellID='',db2='MA_M2M_0706_R',ProbeSetID2='1445101_at',CellID2='',rank='0')) | [0.597 38](javascript:showCorrelationPlot2(db='MA_M2M_0706_R',ProbeSetID='1424199_at',CellID='',db2='MA_M2M_0706_R',ProbeSetID2='1451285_at',CellID2='',rank='0')) | [0.717 38](javascript:showCorrelationPlot2(db='MA_M2M_0706_R',ProbeSetID='1424199_at',CellID='',db2='MA_M2M_0706_R',ProbeSetID2='1441347_at',CellID2='',rank='0')) | [-0.608 38](javascript:showCorrelationPlot2(db='MA_M2M_0706_R',ProbeSetID='1424199_at',CellID='',db2='MA_M2M_0706_R',ProbeSetID2='1426900_at',CellID2='',rank='0')) | [-0.557 38](javascript:showCorrelationPlot2(db='MA_M2M_0706_R',ProbeSetID='1424199_at',CellID='',db2='MA_M2M_0706_R',ProbeSetID2='1434888_a_at',CellID2='',rank='0')) | [-0.540 38](javascript:showCorrelationPlot2(db='MA_M2M_0706_R',ProbeSetID='1424199_at',CellID='',db2='MA_M2M_0706_R',ProbeSetID2='1434773_a_at',CellID2='',rank='0')) | [0.582 38](javascript:showCorrelationPlot2(db='MA_M2M_0706_R',ProbeSetID='1424199_at',CellID='',db2='MA_M2M_0706_R',ProbeSetID2='1452851_at',CellID2='',rank='0')) | [-0.684 38](javascript:showCorrelationPlot2(db='MA_M2M_0706_R',ProbeSetID='1424199_at',CellID='',db2='MA_M2M_0706_R',ProbeSetID2='1441948_x_at',CellID2='',rank='0')) | [0.748 38](javascript:showCorrelationPlot2(db='MA_M2M_0706_R',ProbeSetID='1424199_at',CellID='',db2='MA_M2M_0706_R',ProbeSetID2='1440160_x_at',CellID2='',rank='0')) | [-0.459 38](javascript:showCorrelationPlot2(db='MA_M2M_0706_R',ProbeSetID='1424199_at',CellID='',db2='MA_M2M_0706_R',ProbeSetID2='1424692_at',CellID2='',rank='0')) | [0.703 38](javascript:showCorrelationPlot2(db='MA_M2M_0706_R',ProbeSetID='1424199_at',CellID='',db2='MA_M2M_0706_R',ProbeSetID2='1433112_at',CellID2='',rank='0')) | [-0.593 38](javascript:showCorrelationPlot2(db='MA_M2M_0706_R',ProbeSetID='1424199_at',CellID='',db2='MA_M2M_0706_R',ProbeSetID2='1459962_at',CellID2='',rank='0')) | [0.756 38](javascript:showCorrelationPlot2(db='MA_M2M_0706_R',ProbeSetID='1424199_at',CellID='',db2='MA_M2M_0706_R',ProbeSetID2='1455331_at',CellID2='',rank='0')) | [0.728 38](javascript:showCorrelationPlot2(db='MA_M2M_0706_R',ProbeSetID='1424199_at',CellID='',db2='MA_M2M_0706_R',ProbeSetID2='1447399_at',CellID2='',rank='0')) | [-0.484 38](javascript:showCorrelationPlot2(db='MA_M2M_0706_R',ProbeSetID='1424199_at',CellID='',db2='MA_M2M_0706_R',ProbeSetID2='1434968_a_at',CellID2='',rank='0')) | [0.630 38](javascript:showCorrelationPlot2(db='MA_M2M_0706_R',ProbeSetID='1424199_at',CellID='',db2='MA_M2M_0706_R',ProbeSetID2='1447335_x_at',CellID2='',rank='0')) | [0.768 38](javascript:showCorrelationPlot2(db='MA_M2M_0706_R',ProbeSetID='1424199_at',CellID='',db2='MA_M2M_0706_R',ProbeSetID2='1442239_at',CellID2='',rank='0')) | [0.649 38](javascript:showCorrelationPlot2(db='MA_M2M_0706_R',ProbeSetID='1424199_at',CellID='',db2='MA_M2M_0706_R',ProbeSetID2='1459865_x_at',CellID2='',rank='0')) | [-0.596 38](javascript:showCorrelationPlot2(db='MA_M2M_0706_R',ProbeSetID='1424199_at',CellID='',db2='MA_M2M_0706_R',ProbeSetID2='1438647_x_at',CellID2='',rank='0')) | [0.615 38](javascript:showCorrelationPlot2(db='MA_M2M_0706_R',ProbeSetID='1424199_at',CellID='',db2='MA_M2M_0706_R',ProbeSetID2='1432333_a_at',CellID2='',rank='0')) | [-0.433 38](javascript:showCorrelationPlot2(db='MA_M2M_0706_R',ProbeSetID='1424199_at',CellID='',db2='MA_M2M_0706_R',ProbeSetID2='1444028_s_at',CellID2='',rank='0')) | [0.608 38](javascript:showCorrelationPlot2(db='MA_M2M_0706_R',ProbeSetID='1424199_at',CellID='',db2='MA_M2M_0706_R',ProbeSetID2='1460420_a_at',CellID2='',rank='0')) | [0.557 38](javascript:showCorrelationPlot2(db='MA_M2M_0706_R',ProbeSetID='1424199_at',CellID='',db2='MA_M2M_0706_R',ProbeSetID2='1451530_at',CellID2='',rank='0')) | [-0.519 38](javascript:showCorrelationPlot2(db='MA_M2M_0706_R',ProbeSetID='1424199_at',CellID='',db2='MA_M2M_0706_R',ProbeSetID2='1423785_at',CellID2='',rank='0')) | [0.404 38](javascript:showCorrelationPlot2(db='MA_M2M_0706_R',ProbeSetID='1424199_at',CellID='',db2='MA_M2M_0706_R',ProbeSetID2='1431972_a_at',CellID2='',rank='0')) | [-0.655 38](javascript:showCorrelationPlot2(db='MA_M2M_0706_R',ProbeSetID='1424199_at',CellID='',db2='MA_M2M_0706_R',ProbeSetID2='1448347_a_at',CellID2='',rank='0')) | [0.694 38](javascript:showCorrelationPlot2(db='MA_M2M_0706_R',ProbeSetID='1424199_at',CellID='',db2='MA_M2M_0706_R',ProbeSetID2='1418492_at',CellID2='',rank='0')) | [-0.519 38](javascript:showCorrelationPlot2(db='MA_M2M_0706_R',ProbeSetID='1424199_at',CellID='',db2='MA_M2M_0706_R',ProbeSetID2='1427185_at',CellID2='',rank='0')) | [-0.509 38](javascript:showCorrelationPlot2(db='MA_M2M_0706_R',ProbeSetID='1424199_at',CellID='',db2='MA_M2M_0706_R',ProbeSetID2='1430500_s_at',CellID2='',rank='0')) | [0.583 38](javascript:showCorrelationPlot2(db='MA_M2M_0706_R',ProbeSetID='1424199_at',CellID='',db2='MA_M2M_0706_R',ProbeSetID2='1447549_x_at',CellID2='',rank='0')) | [-0.431 38](javascript:showCorrelationPlot2(db='MA_M2M_0706_R',ProbeSetID='1424199_at',CellID='',db2='MA_M2M_0706_R',ProbeSetID2='1448943_at',CellID2='',rank='0')) | [0.727 38](javascript:showCorrelationPlot2(db='MA_M2M_0706_R',ProbeSetID='1424199_at',CellID='',db2='MA_M2M_0706_R',ProbeSetID2='1433162_at',CellID2='',rank='0')) | [0.644 38](javascript:showCorrelationPlot2(db='MA_M2M_0706_R',ProbeSetID='1424199_at',CellID='',db2='MA_M2M_0706_R',ProbeSetID2='1443494_at',CellID2='',rank='0')) | [-0.377 38](javascript:showCorrelationPlot2(db='MA_M2M_0706_R',ProbeSetID='1424199_at',CellID='',db2='MA_M2M_0706_R',ProbeSetID2='1417426_at',CellID2='',rank='0')) | [0.731 38](javascript:showCorrelationPlot2(db='MA_M2M_0706_R',ProbeSetID='1424199_at',CellID='',db2='MA_M2M_0706_R',ProbeSetID2='1416588_at',CellID2='',rank='0')) | [-0.451 38](javascript:showCorrelationPlot2(db='MA_M2M_0706_R',ProbeSetID='1424199_at',CellID='',db2='MA_M2M_0706_R',ProbeSetID2='1450994_at',CellID2='',rank='0')) | [0.640 38](javascript:showCorrelationPlot2(db='MA_M2M_0706_R',ProbeSetID='1424199_at',CellID='',db2='MA_M2M_0706_R',ProbeSetID2='1420304_x_at',CellID2='',rank='0')) | [-0.719 38](javascript:showCorrelationPlot2(db='MA_M2M_0706_R',ProbeSetID='1424199_at',CellID='',db2='MA_M2M_0706_R',ProbeSetID2='1416500_at',CellID2='',rank='0')) | [n 38](javascript:showDatabase2('MA_M2M_0706_R','1424199_at','')) | [-0.416 38](javascript:showCorrelationPlot2(db='MA_M2M_0706_R',ProbeSetID='1424199_at',CellID='',db2='MA_M2M_0706_R',ProbeSetID2='1437995_x_at',CellID2='',rank='1')) | [-0.565 38](javascript:showCorrelationPlot2(db='MA_M2M_0706_R',ProbeSetID='1424199_at',CellID='',db2='MA_M2M_0706_R',ProbeSetID2='1427914_a_at',CellID2='',rank='1')) | [0.622 38](javascript:showCorrelationPlot2(db='MA_M2M_0706_R',ProbeSetID='1424199_at',CellID='',db2='MA_M2M_0706_R',ProbeSetID2='1420570_x_at',CellID2='',rank='1')) | [0.555 38](javascript:showCorrelationPlot2(db='MA_M2M_0706_R',ProbeSetID='1424199_at',CellID='',db2='MA_M2M_0706_R',ProbeSetID2='1431015_at',CellID2='',rank='1')) | [-0.529 38](javascript:showCorrelationPlot2(db='MA_M2M_0706_R',ProbeSetID='1424199_at',CellID='',db2='MA_M2M_0706_R',ProbeSetID2='1423852_at',CellID2='',rank='1')) | [0.279 38](javascript:showCorrelationPlot2(db='MA_M2M_0706_R',ProbeSetID='1424199_at',CellID='',db2='MA_M2M_0706_R',ProbeSetID2='1431799_at',CellID2='',rank='1')) | [-0.670 38](javascript:showCorrelationPlot2(db='MA_M2M_0706_R',ProbeSetID='1424199_at',CellID='',db2='MA_M2M_0706_R',ProbeSetID2='1423898_a_at',CellID2='',rank='1')) | [0.683 38](javascript:showCorrelationPlot2(db='MA_M2M_0706_R',ProbeSetID='1424199_at',CellID='',db2='MA_M2M_0706_R',ProbeSetID2='1438579_at',CellID2='',rank='1')) | [-0.510 38](javascript:showCorrelationPlot2(db='MA_M2M_0706_R',ProbeSetID='1424199_at',CellID='',db2='MA_M2M_0706_R',ProbeSetID2='1448102_a_at',CellID2='',rank='1')) | [0.506 38](javascript:showCorrelationPlot2(db='MA_M2M_0706_R',ProbeSetID='1424199_at',CellID='',db2='MA_M2M_0706_R',ProbeSetID2='1420943_at',CellID2='',rank='1')) | [0.619 38](javascript:showCorrelationPlot2(db='MA_M2M_0706_R',ProbeSetID='1424199_at',CellID='',db2='MA_M2M_0706_R',ProbeSetID2='1447326_s_at',CellID2='',rank='1')) |
|  | [Trait 40: MA_M2M_0706_R::1437995_x_at](javascript:showDatabase2('MA_M2M_0706_R','1437995_x_at','');)  Sept7 on Chr 9 @ 25.116005 Mb  septic 7 (cell division cycle 10 homolog)  distal 3' UTR | [0.429 38](javascript:showCorrelationPlot2(db='MA_M2M_0706_R',ProbeSetID='1437995_x_at',CellID='',db2='MA_M2M_0706_R',ProbeSetID2='1445101_at',CellID2='',rank='0')) | [-0.302 38](javascript:showCorrelationPlot2(db='MA_M2M_0706_R',ProbeSetID='1437995_x_at',CellID='',db2='MA_M2M_0706_R',ProbeSetID2='1451285_at',CellID2='',rank='0')) | [-0.350 38](javascript:showCorrelationPlot2(db='MA_M2M_0706_R',ProbeSetID='1437995_x_at',CellID='',db2='MA_M2M_0706_R',ProbeSetID2='1441347_at',CellID2='',rank='0')) | [0.687 38](javascript:showCorrelationPlot2(db='MA_M2M_0706_R',ProbeSetID='1437995_x_at',CellID='',db2='MA_M2M_0706_R',ProbeSetID2='1426900_at',CellID2='',rank='0')) | [0.609 38](javascript:showCorrelationPlot2(db='MA_M2M_0706_R',ProbeSetID='1437995_x_at',CellID='',db2='MA_M2M_0706_R',ProbeSetID2='1434888_a_at',CellID2='',rank='0')) | [0.657 38](javascript:showCorrelationPlot2(db='MA_M2M_0706_R',ProbeSetID='1437995_x_at',CellID='',db2='MA_M2M_0706_R',ProbeSetID2='1434773_a_at',CellID2='',rank='0')) | [-0.441 38](javascript:showCorrelationPlot2(db='MA_M2M_0706_R',ProbeSetID='1437995_x_at',CellID='',db2='MA_M2M_0706_R',ProbeSetID2='1452851_at',CellID2='',rank='0')) | [0.433 38](javascript:showCorrelationPlot2(db='MA_M2M_0706_R',ProbeSetID='1437995_x_at',CellID='',db2='MA_M2M_0706_R',ProbeSetID2='1441948_x_at',CellID2='',rank='0')) | [-0.416 38](javascript:showCorrelationPlot2(db='MA_M2M_0706_R',ProbeSetID='1437995_x_at',CellID='',db2='MA_M2M_0706_R',ProbeSetID2='1440160_x_at',CellID2='',rank='0')) | [0.528 38](javascript:showCorrelationPlot2(db='MA_M2M_0706_R',ProbeSetID='1437995_x_at',CellID='',db2='MA_M2M_0706_R',ProbeSetID2='1424692_at',CellID2='',rank='0')) | [-0.362 38](javascript:showCorrelationPlot2(db='MA_M2M_0706_R',ProbeSetID='1437995_x_at',CellID='',db2='MA_M2M_0706_R',ProbeSetID2='1433112_at',CellID2='',rank='0')) | [0.363 38](javascript:showCorrelationPlot2(db='MA_M2M_0706_R',ProbeSetID='1437995_x_at',CellID='',db2='MA_M2M_0706_R',ProbeSetID2='1459962_at',CellID2='',rank='0')) | [-0.240 38](javascript:showCorrelationPlot2(db='MA_M2M_0706_R',ProbeSetID='1437995_x_at',CellID='',db2='MA_M2M_0706_R',ProbeSetID2='1455331_at',CellID2='',rank='0')) | [-0.339 38](javascript:showCorrelationPlot2(db='MA_M2M_0706_R',ProbeSetID='1437995_x_at',CellID='',db2='MA_M2M_0706_R',ProbeSetID2='1447399_at',CellID2='',rank='0')) | [0.535 38](javascript:showCorrelationPlot2(db='MA_M2M_0706_R',ProbeSetID='1437995_x_at',CellID='',db2='MA_M2M_0706_R',ProbeSetID2='1434968_a_at',CellID2='',rank='0')) | [-0.534 38](javascript:showCorrelationPlot2(db='MA_M2M_0706_R',ProbeSetID='1437995_x_at',CellID='',db2='MA_M2M_0706_R',ProbeSetID2='1447335_x_at',CellID2='',rank='0')) | [-0.278 38](javascript:showCorrelationPlot2(db='MA_M2M_0706_R',ProbeSetID='1437995_x_at',CellID='',db2='MA_M2M_0706_R',ProbeSetID2='1442239_at',CellID2='',rank='0')) | [-0.510 38](javascript:showCorrelationPlot2(db='MA_M2M_0706_R',ProbeSetID='1437995_x_at',CellID='',db2='MA_M2M_0706_R',ProbeSetID2='1459865_x_at',CellID2='',rank='0')) | [0.541 38](javascript:showCorrelationPlot2(db='MA_M2M_0706_R',ProbeSetID='1437995_x_at',CellID='',db2='MA_M2M_0706_R',ProbeSetID2='1438647_x_at',CellID2='',rank='0')) | [-0.570 38](javascript:showCorrelationPlot2(db='MA_M2M_0706_R',ProbeSetID='1437995_x_at',CellID='',db2='MA_M2M_0706_R',ProbeSetID2='1432333_a_at',CellID2='',rank='0')) | [0.575 38](javascript:showCorrelationPlot2(db='MA_M2M_0706_R',ProbeSetID='1437995_x_at',CellID='',db2='MA_M2M_0706_R',ProbeSetID2='1444028_s_at',CellID2='',rank='0')) | [-0.608 38](javascript:showCorrelationPlot2(db='MA_M2M_0706_R',ProbeSetID='1437995_x_at',CellID='',db2='MA_M2M_0706_R',ProbeSetID2='1460420_a_at',CellID2='',rank='0')) | [-0.422 38](javascript:showCorrelationPlot2(db='MA_M2M_0706_R',ProbeSetID='1437995_x_at',CellID='',db2='MA_M2M_0706_R',ProbeSetID2='1451530_at',CellID2='',rank='0')) | [0.497 38](javascript:showCorrelationPlot2(db='MA_M2M_0706_R',ProbeSetID='1437995_x_at',CellID='',db2='MA_M2M_0706_R',ProbeSetID2='1423785_at',CellID2='',rank='0')) | [-0.631 38](javascript:showCorrelationPlot2(db='MA_M2M_0706_R',ProbeSetID='1437995_x_at',CellID='',db2='MA_M2M_0706_R',ProbeSetID2='1431972_a_at',CellID2='',rank='0')) | [0.630 38](javascript:showCorrelationPlot2(db='MA_M2M_0706_R',ProbeSetID='1437995_x_at',CellID='',db2='MA_M2M_0706_R',ProbeSetID2='1448347_a_at',CellID2='',rank='0')) | [-0.357 38](javascript:showCorrelationPlot2(db='MA_M2M_0706_R',ProbeSetID='1437995_x_at',CellID='',db2='MA_M2M_0706_R',ProbeSetID2='1418492_at',CellID2='',rank='0')) | [0.571 38](javascript:showCorrelationPlot2(db='MA_M2M_0706_R',ProbeSetID='1437995_x_at',CellID='',db2='MA_M2M_0706_R',ProbeSetID2='1427185_at',CellID2='',rank='0')) | [0.559 38](javascript:showCorrelationPlot2(db='MA_M2M_0706_R',ProbeSetID='1437995_x_at',CellID='',db2='MA_M2M_0706_R',ProbeSetID2='1430500_s_at',CellID2='',rank='0')) | [-0.345 38](javascript:showCorrelationPlot2(db='MA_M2M_0706_R',ProbeSetID='1437995_x_at',CellID='',db2='MA_M2M_0706_R',ProbeSetID2='1447549_x_at',CellID2='',rank='0')) | [0.749 38](javascript:showCorrelationPlot2(db='MA_M2M_0706_R',ProbeSetID='1437995_x_at',CellID='',db2='MA_M2M_0706_R',ProbeSetID2='1448943_at',CellID2='',rank='0')) | [-0.383 38](javascript:showCorrelationPlot2(db='MA_M2M_0706_R',ProbeSetID='1437995_x_at',CellID='',db2='MA_M2M_0706_R',ProbeSetID2='1433162_at',CellID2='',rank='0')) | [-0.471 38](javascript:showCorrelationPlot2(db='MA_M2M_0706_R',ProbeSetID='1437995_x_at',CellID='',db2='MA_M2M_0706_R',ProbeSetID2='1443494_at',CellID2='',rank='0')) | [0.520 38](javascript:showCorrelationPlot2(db='MA_M2M_0706_R',ProbeSetID='1437995_x_at',CellID='',db2='MA_M2M_0706_R',ProbeSetID2='1417426_at',CellID2='',rank='0')) | [-0.440 38](javascript:showCorrelationPlot2(db='MA_M2M_0706_R',ProbeSetID='1437995_x_at',CellID='',db2='MA_M2M_0706_R',ProbeSetID2='1416588_at',CellID2='',rank='0')) | [0.538 38](javascript:showCorrelationPlot2(db='MA_M2M_0706_R',ProbeSetID='1437995_x_at',CellID='',db2='MA_M2M_0706_R',ProbeSetID2='1450994_at',CellID2='',rank='0')) | [-0.494 38](javascript:showCorrelationPlot2(db='MA_M2M_0706_R',ProbeSetID='1437995_x_at',CellID='',db2='MA_M2M_0706_R',ProbeSetID2='1420304_x_at',CellID2='',rank='0')) | [0.638 38](javascript:showCorrelationPlot2(db='MA_M2M_0706_R',ProbeSetID='1437995_x_at',CellID='',db2='MA_M2M_0706_R',ProbeSetID2='1416500_at',CellID2='',rank='0')) | [-0.366 38](javascript:showCorrelationPlot2(db='MA_M2M_0706_R',ProbeSetID='1437995_x_at',CellID='',db2='MA_M2M_0706_R',ProbeSetID2='1424199_at',CellID2='',rank='0')) | [n 38](javascript:showDatabase2('MA_M2M_0706_R','1437995_x_at','')) | [0.532 38](javascript:showCorrelationPlot2(db='MA_M2M_0706_R',ProbeSetID='1437995_x_at',CellID='',db2='MA_M2M_0706_R',ProbeSetID2='1427914_a_at',CellID2='',rank='1')) | [-0.439 38](javascript:showCorrelationPlot2(db='MA_M2M_0706_R',ProbeSetID='1437995_x_at',CellID='',db2='MA_M2M_0706_R',ProbeSetID2='1420570_x_at',CellID2='',rank='1')) | [-0.579 38](javascript:showCorrelationPlot2(db='MA_M2M_0706_R',ProbeSetID='1437995_x_at',CellID='',db2='MA_M2M_0706_R',ProbeSetID2='1431015_at',CellID2='',rank='1')) | [0.279 38](javascript:showCorrelationPlot2(db='MA_M2M_0706_R',ProbeSetID='1437995_x_at',CellID='',db2='MA_M2M_0706_R',ProbeSetID2='1423852_at',CellID2='',rank='1')) | [-0.415 38](javascript:showCorrelationPlot2(db='MA_M2M_0706_R',ProbeSetID='1437995_x_at',CellID='',db2='MA_M2M_0706_R',ProbeSetID2='1431799_at',CellID2='',rank='1')) | [0.613 38](javascript:showCorrelationPlot2(db='MA_M2M_0706_R',ProbeSetID='1437995_x_at',CellID='',db2='MA_M2M_0706_R',ProbeSetID2='1423898_a_at',CellID2='',rank='1')) | [-0.542 38](javascript:showCorrelationPlot2(db='MA_M2M_0706_R',ProbeSetID='1437995_x_at',CellID='',db2='MA_M2M_0706_R',ProbeSetID2='1438579_at',CellID2='',rank='1')) | [0.601 38](javascript:showCorrelationPlot2(db='MA_M2M_0706_R',ProbeSetID='1437995_x_at',CellID='',db2='MA_M2M_0706_R',ProbeSetID2='1448102_a_at',CellID2='',rank='1')) | [-0.481 38](javascript:showCorrelationPlot2(db='MA_M2M_0706_R',ProbeSetID='1437995_x_at',CellID='',db2='MA_M2M_0706_R',ProbeSetID2='1420943_at',CellID2='',rank='1')) | [-0.437 38](javascript:showCorrelationPlot2(db='MA_M2M_0706_R',ProbeSetID='1437995_x_at',CellID='',db2='MA_M2M_0706_R',ProbeSetID2='1447326_s_at',CellID2='',rank='1')) |
|  | [Trait 41: MA_M2M_0706_R::1427914_a_at](javascript:showDatabase2('MA_M2M_0706_R','1427914_a_at','');)  Tceb1 on Chr 1 @ 16.633372 Mb  transcription elongation factor B (SIII), polypeptide 1  last three exons | [0.396 38](javascript:showCorrelationPlot2(db='MA_M2M_0706_R',ProbeSetID='1427914_a_at',CellID='',db2='MA_M2M_0706_R',ProbeSetID2='1445101_at',CellID2='',rank='0')) | [-0.492 38](javascript:showCorrelationPlot2(db='MA_M2M_0706_R',ProbeSetID='1427914_a_at',CellID='',db2='MA_M2M_0706_R',ProbeSetID2='1451285_at',CellID2='',rank='0')) | [-0.474 38](javascript:showCorrelationPlot2(db='MA_M2M_0706_R',ProbeSetID='1427914_a_at',CellID='',db2='MA_M2M_0706_R',ProbeSetID2='1441347_at',CellID2='',rank='0')) | [0.550 38](javascript:showCorrelationPlot2(db='MA_M2M_0706_R',ProbeSetID='1427914_a_at',CellID='',db2='MA_M2M_0706_R',ProbeSetID2='1426900_at',CellID2='',rank='0')) | [0.547 38](javascript:showCorrelationPlot2(db='MA_M2M_0706_R',ProbeSetID='1427914_a_at',CellID='',db2='MA_M2M_0706_R',ProbeSetID2='1434888_a_at',CellID2='',rank='0')) | [0.567 38](javascript:showCorrelationPlot2(db='MA_M2M_0706_R',ProbeSetID='1427914_a_at',CellID='',db2='MA_M2M_0706_R',ProbeSetID2='1434773_a_at',CellID2='',rank='0')) | [-0.538 38](javascript:showCorrelationPlot2(db='MA_M2M_0706_R',ProbeSetID='1427914_a_at',CellID='',db2='MA_M2M_0706_R',ProbeSetID2='1452851_at',CellID2='',rank='0')) | [0.486 38](javascript:showCorrelationPlot2(db='MA_M2M_0706_R',ProbeSetID='1427914_a_at',CellID='',db2='MA_M2M_0706_R',ProbeSetID2='1441948_x_at',CellID2='',rank='0')) | [-0.512 38](javascript:showCorrelationPlot2(db='MA_M2M_0706_R',ProbeSetID='1427914_a_at',CellID='',db2='MA_M2M_0706_R',ProbeSetID2='1440160_x_at',CellID2='',rank='0')) | [0.481 38](javascript:showCorrelationPlot2(db='MA_M2M_0706_R',ProbeSetID='1427914_a_at',CellID='',db2='MA_M2M_0706_R',ProbeSetID2='1424692_at',CellID2='',rank='0')) | [-0.504 38](javascript:showCorrelationPlot2(db='MA_M2M_0706_R',ProbeSetID='1427914_a_at',CellID='',db2='MA_M2M_0706_R',ProbeSetID2='1433112_at',CellID2='',rank='0')) | [0.436 38](javascript:showCorrelationPlot2(db='MA_M2M_0706_R',ProbeSetID='1427914_a_at',CellID='',db2='MA_M2M_0706_R',ProbeSetID2='1459962_at',CellID2='',rank='0')) | [-0.421 38](javascript:showCorrelationPlot2(db='MA_M2M_0706_R',ProbeSetID='1427914_a_at',CellID='',db2='MA_M2M_0706_R',ProbeSetID2='1455331_at',CellID2='',rank='0')) | [-0.582 38](javascript:showCorrelationPlot2(db='MA_M2M_0706_R',ProbeSetID='1427914_a_at',CellID='',db2='MA_M2M_0706_R',ProbeSetID2='1447399_at',CellID2='',rank='0')) | [0.376 38](javascript:showCorrelationPlot2(db='MA_M2M_0706_R',ProbeSetID='1427914_a_at',CellID='',db2='MA_M2M_0706_R',ProbeSetID2='1434968_a_at',CellID2='',rank='0')) | [-0.552 38](javascript:showCorrelationPlot2(db='MA_M2M_0706_R',ProbeSetID='1427914_a_at',CellID='',db2='MA_M2M_0706_R',ProbeSetID2='1447335_x_at',CellID2='',rank='0')) | [-0.503 38](javascript:showCorrelationPlot2(db='MA_M2M_0706_R',ProbeSetID='1427914_a_at',CellID='',db2='MA_M2M_0706_R',ProbeSetID2='1442239_at',CellID2='',rank='0')) | [-0.667 38](javascript:showCorrelationPlot2(db='MA_M2M_0706_R',ProbeSetID='1427914_a_at',CellID='',db2='MA_M2M_0706_R',ProbeSetID2='1459865_x_at',CellID2='',rank='0')) | [0.623 38](javascript:showCorrelationPlot2(db='MA_M2M_0706_R',ProbeSetID='1427914_a_at',CellID='',db2='MA_M2M_0706_R',ProbeSetID2='1438647_x_at',CellID2='',rank='0')) | [-0.352 38](javascript:showCorrelationPlot2(db='MA_M2M_0706_R',ProbeSetID='1427914_a_at',CellID='',db2='MA_M2M_0706_R',ProbeSetID2='1432333_a_at',CellID2='',rank='0')) | [0.538 38](javascript:showCorrelationPlot2(db='MA_M2M_0706_R',ProbeSetID='1427914_a_at',CellID='',db2='MA_M2M_0706_R',ProbeSetID2='1444028_s_at',CellID2='',rank='0')) | [-0.574 38](javascript:showCorrelationPlot2(db='MA_M2M_0706_R',ProbeSetID='1427914_a_at',CellID='',db2='MA_M2M_0706_R',ProbeSetID2='1460420_a_at',CellID2='',rank='0')) | [-0.485 38](javascript:showCorrelationPlot2(db='MA_M2M_0706_R',ProbeSetID='1427914_a_at',CellID='',db2='MA_M2M_0706_R',ProbeSetID2='1451530_at',CellID2='',rank='0')) | [0.315 38](javascript:showCorrelationPlot2(db='MA_M2M_0706_R',ProbeSetID='1427914_a_at',CellID='',db2='MA_M2M_0706_R',ProbeSetID2='1423785_at',CellID2='',rank='0')) | [-0.305 38](javascript:showCorrelationPlot2(db='MA_M2M_0706_R',ProbeSetID='1427914_a_at',CellID='',db2='MA_M2M_0706_R',ProbeSetID2='1431972_a_at',CellID2='',rank='0')) | [0.605 38](javascript:showCorrelationPlot2(db='MA_M2M_0706_R',ProbeSetID='1427914_a_at',CellID='',db2='MA_M2M_0706_R',ProbeSetID2='1448347_a_at',CellID2='',rank='0')) | [-0.480 38](javascript:showCorrelationPlot2(db='MA_M2M_0706_R',ProbeSetID='1427914_a_at',CellID='',db2='MA_M2M_0706_R',ProbeSetID2='1418492_at',CellID2='',rank='0')) | [0.384 38](javascript:showCorrelationPlot2(db='MA_M2M_0706_R',ProbeSetID='1427914_a_at',CellID='',db2='MA_M2M_0706_R',ProbeSetID2='1427185_at',CellID2='',rank='0')) | [0.619 38](javascript:showCorrelationPlot2(db='MA_M2M_0706_R',ProbeSetID='1427914_a_at',CellID='',db2='MA_M2M_0706_R',ProbeSetID2='1430500_s_at',CellID2='',rank='0')) | [-0.555 38](javascript:showCorrelationPlot2(db='MA_M2M_0706_R',ProbeSetID='1427914_a_at',CellID='',db2='MA_M2M_0706_R',ProbeSetID2='1447549_x_at',CellID2='',rank='0')) | [0.561 38](javascript:showCorrelationPlot2(db='MA_M2M_0706_R',ProbeSetID='1427914_a_at',CellID='',db2='MA_M2M_0706_R',ProbeSetID2='1448943_at',CellID2='',rank='0')) | [-0.543 38](javascript:showCorrelationPlot2(db='MA_M2M_0706_R',ProbeSetID='1427914_a_at',CellID='',db2='MA_M2M_0706_R',ProbeSetID2='1433162_at',CellID2='',rank='0')) | [-0.576 38](javascript:showCorrelationPlot2(db='MA_M2M_0706_R',ProbeSetID='1427914_a_at',CellID='',db2='MA_M2M_0706_R',ProbeSetID2='1443494_at',CellID2='',rank='0')) | [0.540 38](javascript:showCorrelationPlot2(db='MA_M2M_0706_R',ProbeSetID='1427914_a_at',CellID='',db2='MA_M2M_0706_R',ProbeSetID2='1417426_at',CellID2='',rank='0')) | [-0.604 38](javascript:showCorrelationPlot2(db='MA_M2M_0706_R',ProbeSetID='1427914_a_at',CellID='',db2='MA_M2M_0706_R',ProbeSetID2='1416588_at',CellID2='',rank='0')) | [0.703 38](javascript:showCorrelationPlot2(db='MA_M2M_0706_R',ProbeSetID='1427914_a_at',CellID='',db2='MA_M2M_0706_R',ProbeSetID2='1450994_at',CellID2='',rank='0')) | [-0.595 38](javascript:showCorrelationPlot2(db='MA_M2M_0706_R',ProbeSetID='1427914_a_at',CellID='',db2='MA_M2M_0706_R',ProbeSetID2='1420304_x_at',CellID2='',rank='0')) | [0.669 38](javascript:showCorrelationPlot2(db='MA_M2M_0706_R',ProbeSetID='1427914_a_at',CellID='',db2='MA_M2M_0706_R',ProbeSetID2='1416500_at',CellID2='',rank='0')) | [-0.652 38](javascript:showCorrelationPlot2(db='MA_M2M_0706_R',ProbeSetID='1427914_a_at',CellID='',db2='MA_M2M_0706_R',ProbeSetID2='1424199_at',CellID2='',rank='0')) | [0.546 38](javascript:showCorrelationPlot2(db='MA_M2M_0706_R',ProbeSetID='1427914_a_at',CellID='',db2='MA_M2M_0706_R',ProbeSetID2='1437995_x_at',CellID2='',rank='0')) | [n 38](javascript:showDatabase2('MA_M2M_0706_R','1427914_a_at','')) | [-0.429 38](javascript:showCorrelationPlot2(db='MA_M2M_0706_R',ProbeSetID='1427914_a_at',CellID='',db2='MA_M2M_0706_R',ProbeSetID2='1420570_x_at',CellID2='',rank='1')) | [-0.526 38](javascript:showCorrelationPlot2(db='MA_M2M_0706_R',ProbeSetID='1427914_a_at',CellID='',db2='MA_M2M_0706_R',ProbeSetID2='1431015_at',CellID2='',rank='1')) | [0.318 38](javascript:showCorrelationPlot2(db='MA_M2M_0706_R',ProbeSetID='1427914_a_at',CellID='',db2='MA_M2M_0706_R',ProbeSetID2='1423852_at',CellID2='',rank='1')) | [-0.432 38](javascript:showCorrelationPlot2(db='MA_M2M_0706_R',ProbeSetID='1427914_a_at',CellID='',db2='MA_M2M_0706_R',ProbeSetID2='1431799_at',CellID2='',rank='1')) | [0.582 38](javascript:showCorrelationPlot2(db='MA_M2M_0706_R',ProbeSetID='1427914_a_at',CellID='',db2='MA_M2M_0706_R',ProbeSetID2='1423898_a_at',CellID2='',rank='1')) | [-0.438 38](javascript:showCorrelationPlot2(db='MA_M2M_0706_R',ProbeSetID='1427914_a_at',CellID='',db2='MA_M2M_0706_R',ProbeSetID2='1438579_at',CellID2='',rank='1')) | [0.474 38](javascript:showCorrelationPlot2(db='MA_M2M_0706_R',ProbeSetID='1427914_a_at',CellID='',db2='MA_M2M_0706_R',ProbeSetID2='1448102_a_at',CellID2='',rank='1')) | [-0.298 38](javascript:showCorrelationPlot2(db='MA_M2M_0706_R',ProbeSetID='1427914_a_at',CellID='',db2='MA_M2M_0706_R',ProbeSetID2='1420943_at',CellID2='',rank='1')) | [-0.379 38](javascript:showCorrelationPlot2(db='MA_M2M_0706_R',ProbeSetID='1427914_a_at',CellID='',db2='MA_M2M_0706_R',ProbeSetID2='1447326_s_at',CellID2='',rank='1')) |
|  | [Trait 42: MA_M2M_0706_R::1420570_x_at](javascript:showDatabase2('MA_M2M_0706_R','1420570_x_at','');)  Tcl1b3 on Chr 12 @ 106.433311 Mb  T-cell leukemia/lymphoma 1B, 3  proximal 3' UTR | [-0.583 38](javascript:showCorrelationPlot2(db='MA_M2M_0706_R',ProbeSetID='1420570_x_at',CellID='',db2='MA_M2M_0706_R',ProbeSetID2='1445101_at',CellID2='',rank='0')) | [0.334 38](javascript:showCorrelationPlot2(db='MA_M2M_0706_R',ProbeSetID='1420570_x_at',CellID='',db2='MA_M2M_0706_R',ProbeSetID2='1451285_at',CellID2='',rank='0')) | [0.555 38](javascript:showCorrelationPlot2(db='MA_M2M_0706_R',ProbeSetID='1420570_x_at',CellID='',db2='MA_M2M_0706_R',ProbeSetID2='1441347_at',CellID2='',rank='0')) | [-0.485 38](javascript:showCorrelationPlot2(db='MA_M2M_0706_R',ProbeSetID='1420570_x_at',CellID='',db2='MA_M2M_0706_R',ProbeSetID2='1426900_at',CellID2='',rank='0')) | [-0.578 38](javascript:showCorrelationPlot2(db='MA_M2M_0706_R',ProbeSetID='1420570_x_at',CellID='',db2='MA_M2M_0706_R',ProbeSetID2='1434888_a_at',CellID2='',rank='0')) | [-0.517 38](javascript:showCorrelationPlot2(db='MA_M2M_0706_R',ProbeSetID='1420570_x_at',CellID='',db2='MA_M2M_0706_R',ProbeSetID2='1434773_a_at',CellID2='',rank='0')) | [0.600 38](javascript:showCorrelationPlot2(db='MA_M2M_0706_R',ProbeSetID='1420570_x_at',CellID='',db2='MA_M2M_0706_R',ProbeSetID2='1452851_at',CellID2='',rank='0')) | [-0.476 38](javascript:showCorrelationPlot2(db='MA_M2M_0706_R',ProbeSetID='1420570_x_at',CellID='',db2='MA_M2M_0706_R',ProbeSetID2='1441948_x_at',CellID2='',rank='0')) | [0.783 38](javascript:showCorrelationPlot2(db='MA_M2M_0706_R',ProbeSetID='1420570_x_at',CellID='',db2='MA_M2M_0706_R',ProbeSetID2='1440160_x_at',CellID2='',rank='0')) | [-0.554 38](javascript:showCorrelationPlot2(db='MA_M2M_0706_R',ProbeSetID='1420570_x_at',CellID='',db2='MA_M2M_0706_R',ProbeSetID2='1424692_at',CellID2='',rank='0')) | [0.676 38](javascript:showCorrelationPlot2(db='MA_M2M_0706_R',ProbeSetID='1420570_x_at',CellID='',db2='MA_M2M_0706_R',ProbeSetID2='1433112_at',CellID2='',rank='0')) | [-0.435 38](javascript:showCorrelationPlot2(db='MA_M2M_0706_R',ProbeSetID='1420570_x_at',CellID='',db2='MA_M2M_0706_R',ProbeSetID2='1459962_at',CellID2='',rank='0')) | [0.598 38](javascript:showCorrelationPlot2(db='MA_M2M_0706_R',ProbeSetID='1420570_x_at',CellID='',db2='MA_M2M_0706_R',ProbeSetID2='1455331_at',CellID2='',rank='0')) | [0.574 38](javascript:showCorrelationPlot2(db='MA_M2M_0706_R',ProbeSetID='1420570_x_at',CellID='',db2='MA_M2M_0706_R',ProbeSetID2='1447399_at',CellID2='',rank='0')) | [-0.545 38](javascript:showCorrelationPlot2(db='MA_M2M_0706_R',ProbeSetID='1420570_x_at',CellID='',db2='MA_M2M_0706_R',ProbeSetID2='1434968_a_at',CellID2='',rank='0')) | [0.703 38](javascript:showCorrelationPlot2(db='MA_M2M_0706_R',ProbeSetID='1420570_x_at',CellID='',db2='MA_M2M_0706_R',ProbeSetID2='1447335_x_at',CellID2='',rank='0')) | [0.701 38](javascript:showCorrelationPlot2(db='MA_M2M_0706_R',ProbeSetID='1420570_x_at',CellID='',db2='MA_M2M_0706_R',ProbeSetID2='1442239_at',CellID2='',rank='0')) | [0.661 38](javascript:showCorrelationPlot2(db='MA_M2M_0706_R',ProbeSetID='1420570_x_at',CellID='',db2='MA_M2M_0706_R',ProbeSetID2='1459865_x_at',CellID2='',rank='0')) | [-0.413 38](javascript:showCorrelationPlot2(db='MA_M2M_0706_R',ProbeSetID='1420570_x_at',CellID='',db2='MA_M2M_0706_R',ProbeSetID2='1438647_x_at',CellID2='',rank='0')) | [0.394 38](javascript:showCorrelationPlot2(db='MA_M2M_0706_R',ProbeSetID='1420570_x_at',CellID='',db2='MA_M2M_0706_R',ProbeSetID2='1432333_a_at',CellID2='',rank='0')) | [-0.422 38](javascript:showCorrelationPlot2(db='MA_M2M_0706_R',ProbeSetID='1420570_x_at',CellID='',db2='MA_M2M_0706_R',ProbeSetID2='1444028_s_at',CellID2='',rank='0')) | [0.614 38](javascript:showCorrelationPlot2(db='MA_M2M_0706_R',ProbeSetID='1420570_x_at',CellID='',db2='MA_M2M_0706_R',ProbeSetID2='1460420_a_at',CellID2='',rank='0')) | [0.581 38](javascript:showCorrelationPlot2(db='MA_M2M_0706_R',ProbeSetID='1420570_x_at',CellID='',db2='MA_M2M_0706_R',ProbeSetID2='1451530_at',CellID2='',rank='0')) | [-0.338 38](javascript:showCorrelationPlot2(db='MA_M2M_0706_R',ProbeSetID='1420570_x_at',CellID='',db2='MA_M2M_0706_R',ProbeSetID2='1423785_at',CellID2='',rank='0')) | [0.377 38](javascript:showCorrelationPlot2(db='MA_M2M_0706_R',ProbeSetID='1420570_x_at',CellID='',db2='MA_M2M_0706_R',ProbeSetID2='1431972_a_at',CellID2='',rank='0')) | [-0.355 38](javascript:showCorrelationPlot2(db='MA_M2M_0706_R',ProbeSetID='1420570_x_at',CellID='',db2='MA_M2M_0706_R',ProbeSetID2='1448347_a_at',CellID2='',rank='0')) | [0.594 38](javascript:showCorrelationPlot2(db='MA_M2M_0706_R',ProbeSetID='1420570_x_at',CellID='',db2='MA_M2M_0706_R',ProbeSetID2='1418492_at',CellID2='',rank='0')) | [-0.569 38](javascript:showCorrelationPlot2(db='MA_M2M_0706_R',ProbeSetID='1420570_x_at',CellID='',db2='MA_M2M_0706_R',ProbeSetID2='1427185_at',CellID2='',rank='0')) | [-0.522 38](javascript:showCorrelationPlot2(db='MA_M2M_0706_R',ProbeSetID='1420570_x_at',CellID='',db2='MA_M2M_0706_R',ProbeSetID2='1430500_s_at',CellID2='',rank='0')) | [0.706 38](javascript:showCorrelationPlot2(db='MA_M2M_0706_R',ProbeSetID='1420570_x_at',CellID='',db2='MA_M2M_0706_R',ProbeSetID2='1447549_x_at',CellID2='',rank='0')) | [-0.403 38](javascript:showCorrelationPlot2(db='MA_M2M_0706_R',ProbeSetID='1420570_x_at',CellID='',db2='MA_M2M_0706_R',ProbeSetID2='1448943_at',CellID2='',rank='0')) | [0.716 38](javascript:showCorrelationPlot2(db='MA_M2M_0706_R',ProbeSetID='1420570_x_at',CellID='',db2='MA_M2M_0706_R',ProbeSetID2='1433162_at',CellID2='',rank='0')) | [0.587 38](javascript:showCorrelationPlot2(db='MA_M2M_0706_R',ProbeSetID='1420570_x_at',CellID='',db2='MA_M2M_0706_R',ProbeSetID2='1443494_at',CellID2='',rank='0')) | [-0.272 38](javascript:showCorrelationPlot2(db='MA_M2M_0706_R',ProbeSetID='1420570_x_at',CellID='',db2='MA_M2M_0706_R',ProbeSetID2='1417426_at',CellID2='',rank='0')) | [0.704 38](javascript:showCorrelationPlot2(db='MA_M2M_0706_R',ProbeSetID='1420570_x_at',CellID='',db2='MA_M2M_0706_R',ProbeSetID2='1416588_at',CellID2='',rank='0')) | [-0.475 38](javascript:showCorrelationPlot2(db='MA_M2M_0706_R',ProbeSetID='1420570_x_at',CellID='',db2='MA_M2M_0706_R',ProbeSetID2='1450994_at',CellID2='',rank='0')) | [0.722 38](javascript:showCorrelationPlot2(db='MA_M2M_0706_R',ProbeSetID='1420570_x_at',CellID='',db2='MA_M2M_0706_R',ProbeSetID2='1420304_x_at',CellID2='',rank='0')) | [-0.517 38](javascript:showCorrelationPlot2(db='MA_M2M_0706_R',ProbeSetID='1420570_x_at',CellID='',db2='MA_M2M_0706_R',ProbeSetID2='1416500_at',CellID2='',rank='0')) | [0.650 38](javascript:showCorrelationPlot2(db='MA_M2M_0706_R',ProbeSetID='1420570_x_at',CellID='',db2='MA_M2M_0706_R',ProbeSetID2='1424199_at',CellID2='',rank='0')) | [-0.326 38](javascript:showCorrelationPlot2(db='MA_M2M_0706_R',ProbeSetID='1420570_x_at',CellID='',db2='MA_M2M_0706_R',ProbeSetID2='1437995_x_at',CellID2='',rank='0')) | [-0.472 38](javascript:showCorrelationPlot2(db='MA_M2M_0706_R',ProbeSetID='1420570_x_at',CellID='',db2='MA_M2M_0706_R',ProbeSetID2='1427914_a_at',CellID2='',rank='0')) | [n 38](javascript:showDatabase2('MA_M2M_0706_R','1420570_x_at','')) | [0.598 38](javascript:showCorrelationPlot2(db='MA_M2M_0706_R',ProbeSetID='1420570_x_at',CellID='',db2='MA_M2M_0706_R',ProbeSetID2='1431015_at',CellID2='',rank='1')) | [-0.596 38](javascript:showCorrelationPlot2(db='MA_M2M_0706_R',ProbeSetID='1420570_x_at',CellID='',db2='MA_M2M_0706_R',ProbeSetID2='1423852_at',CellID2='',rank='1')) | [0.370 38](javascript:showCorrelationPlot2(db='MA_M2M_0706_R',ProbeSetID='1420570_x_at',CellID='',db2='MA_M2M_0706_R',ProbeSetID2='1431799_at',CellID2='',rank='1')) | [-0.461 38](javascript:showCorrelationPlot2(db='MA_M2M_0706_R',ProbeSetID='1420570_x_at',CellID='',db2='MA_M2M_0706_R',ProbeSetID2='1423898_a_at',CellID2='',rank='1')) | [0.486 38](javascript:showCorrelationPlot2(db='MA_M2M_0706_R',ProbeSetID='1420570_x_at',CellID='',db2='MA_M2M_0706_R',ProbeSetID2='1438579_at',CellID2='',rank='1')) | [-0.612 38](javascript:showCorrelationPlot2(db='MA_M2M_0706_R',ProbeSetID='1420570_x_at',CellID='',db2='MA_M2M_0706_R',ProbeSetID2='1448102_a_at',CellID2='',rank='1')) | [0.500 38](javascript:showCorrelationPlot2(db='MA_M2M_0706_R',ProbeSetID='1420570_x_at',CellID='',db2='MA_M2M_0706_R',ProbeSetID2='1420943_at',CellID2='',rank='1')) | [0.601 38](javascript:showCorrelationPlot2(db='MA_M2M_0706_R',ProbeSetID='1420570_x_at',CellID='',db2='MA_M2M_0706_R',ProbeSetID2='1447326_s_at',CellID2='',rank='1')) |
|  | [Trait 43: MA_M2M_0706_R::1431015_at](javascript:showDatabase2('MA_M2M_0706_R','1431015_at','');)  Tmem129 on Chr 5 @ 33.997361 Mb  transmembrane protein 129  exon 3 | [-0.487 38](javascript:showCorrelationPlot2(db='MA_M2M_0706_R',ProbeSetID='1431015_at',CellID='',db2='MA_M2M_0706_R',ProbeSetID2='1445101_at',CellID2='',rank='0')) | [0.500 38](javascript:showCorrelationPlot2(db='MA_M2M_0706_R',ProbeSetID='1431015_at',CellID='',db2='MA_M2M_0706_R',ProbeSetID2='1451285_at',CellID2='',rank='0')) | [0.538 38](javascript:showCorrelationPlot2(db='MA_M2M_0706_R',ProbeSetID='1431015_at',CellID='',db2='MA_M2M_0706_R',ProbeSetID2='1441347_at',CellID2='',rank='0')) | [-0.601 38](javascript:showCorrelationPlot2(db='MA_M2M_0706_R',ProbeSetID='1431015_at',CellID='',db2='MA_M2M_0706_R',ProbeSetID2='1426900_at',CellID2='',rank='0')) | [-0.723 38](javascript:showCorrelationPlot2(db='MA_M2M_0706_R',ProbeSetID='1431015_at',CellID='',db2='MA_M2M_0706_R',ProbeSetID2='1434888_a_at',CellID2='',rank='0')) | [-0.518 38](javascript:showCorrelationPlot2(db='MA_M2M_0706_R',ProbeSetID='1431015_at',CellID='',db2='MA_M2M_0706_R',ProbeSetID2='1434773_a_at',CellID2='',rank='0')) | [0.582 38](javascript:showCorrelationPlot2(db='MA_M2M_0706_R',ProbeSetID='1431015_at',CellID='',db2='MA_M2M_0706_R',ProbeSetID2='1452851_at',CellID2='',rank='0')) | [-0.610 38](javascript:showCorrelationPlot2(db='MA_M2M_0706_R',ProbeSetID='1431015_at',CellID='',db2='MA_M2M_0706_R',ProbeSetID2='1441948_x_at',CellID2='',rank='0')) | [0.532 38](javascript:showCorrelationPlot2(db='MA_M2M_0706_R',ProbeSetID='1431015_at',CellID='',db2='MA_M2M_0706_R',ProbeSetID2='1440160_x_at',CellID2='',rank='0')) | [-0.372 38](javascript:showCorrelationPlot2(db='MA_M2M_0706_R',ProbeSetID='1431015_at',CellID='',db2='MA_M2M_0706_R',ProbeSetID2='1424692_at',CellID2='',rank='0')) | [0.512 38](javascript:showCorrelationPlot2(db='MA_M2M_0706_R',ProbeSetID='1431015_at',CellID='',db2='MA_M2M_0706_R',ProbeSetID2='1433112_at',CellID2='',rank='0')) | [-0.489 38](javascript:showCorrelationPlot2(db='MA_M2M_0706_R',ProbeSetID='1431015_at',CellID='',db2='MA_M2M_0706_R',ProbeSetID2='1459962_at',CellID2='',rank='0')) | [0.504 38](javascript:showCorrelationPlot2(db='MA_M2M_0706_R',ProbeSetID='1431015_at',CellID='',db2='MA_M2M_0706_R',ProbeSetID2='1455331_at',CellID2='',rank='0')) | [0.407 38](javascript:showCorrelationPlot2(db='MA_M2M_0706_R',ProbeSetID='1431015_at',CellID='',db2='MA_M2M_0706_R',ProbeSetID2='1447399_at',CellID2='',rank='0')) | [-0.551 38](javascript:showCorrelationPlot2(db='MA_M2M_0706_R',ProbeSetID='1431015_at',CellID='',db2='MA_M2M_0706_R',ProbeSetID2='1434968_a_at',CellID2='',rank='0')) | [0.648 38](javascript:showCorrelationPlot2(db='MA_M2M_0706_R',ProbeSetID='1431015_at',CellID='',db2='MA_M2M_0706_R',ProbeSetID2='1447335_x_at',CellID2='',rank='0')) | [0.707 38](javascript:showCorrelationPlot2(db='MA_M2M_0706_R',ProbeSetID='1431015_at',CellID='',db2='MA_M2M_0706_R',ProbeSetID2='1442239_at',CellID2='',rank='0')) | [0.582 38](javascript:showCorrelationPlot2(db='MA_M2M_0706_R',ProbeSetID='1431015_at',CellID='',db2='MA_M2M_0706_R',ProbeSetID2='1459865_x_at',CellID2='',rank='0')) | [-0.517 38](javascript:showCorrelationPlot2(db='MA_M2M_0706_R',ProbeSetID='1431015_at',CellID='',db2='MA_M2M_0706_R',ProbeSetID2='1438647_x_at',CellID2='',rank='0')) | [0.454 38](javascript:showCorrelationPlot2(db='MA_M2M_0706_R',ProbeSetID='1431015_at',CellID='',db2='MA_M2M_0706_R',ProbeSetID2='1432333_a_at',CellID2='',rank='0')) | [-0.568 38](javascript:showCorrelationPlot2(db='MA_M2M_0706_R',ProbeSetID='1431015_at',CellID='',db2='MA_M2M_0706_R',ProbeSetID2='1444028_s_at',CellID2='',rank='0')) | [0.630 38](javascript:showCorrelationPlot2(db='MA_M2M_0706_R',ProbeSetID='1431015_at',CellID='',db2='MA_M2M_0706_R',ProbeSetID2='1460420_a_at',CellID2='',rank='0')) | [0.578 38](javascript:showCorrelationPlot2(db='MA_M2M_0706_R',ProbeSetID='1431015_at',CellID='',db2='MA_M2M_0706_R',ProbeSetID2='1451530_at',CellID2='',rank='0')) | [-0.503 38](javascript:showCorrelationPlot2(db='MA_M2M_0706_R',ProbeSetID='1431015_at',CellID='',db2='MA_M2M_0706_R',ProbeSetID2='1423785_at',CellID2='',rank='0')) | [0.528 38](javascript:showCorrelationPlot2(db='MA_M2M_0706_R',ProbeSetID='1431015_at',CellID='',db2='MA_M2M_0706_R',ProbeSetID2='1431972_a_at',CellID2='',rank='0')) | [-0.602 38](javascript:showCorrelationPlot2(db='MA_M2M_0706_R',ProbeSetID='1431015_at',CellID='',db2='MA_M2M_0706_R',ProbeSetID2='1448347_a_at',CellID2='',rank='0')) | [0.399 38](javascript:showCorrelationPlot2(db='MA_M2M_0706_R',ProbeSetID='1431015_at',CellID='',db2='MA_M2M_0706_R',ProbeSetID2='1418492_at',CellID2='',rank='0')) | [-0.519 38](javascript:showCorrelationPlot2(db='MA_M2M_0706_R',ProbeSetID='1431015_at',CellID='',db2='MA_M2M_0706_R',ProbeSetID2='1427185_at',CellID2='',rank='0')) | [-0.521 38](javascript:showCorrelationPlot2(db='MA_M2M_0706_R',ProbeSetID='1431015_at',CellID='',db2='MA_M2M_0706_R',ProbeSetID2='1430500_s_at',CellID2='',rank='0')) | [0.554 38](javascript:showCorrelationPlot2(db='MA_M2M_0706_R',ProbeSetID='1431015_at',CellID='',db2='MA_M2M_0706_R',ProbeSetID2='1447549_x_at',CellID2='',rank='0')) | [-0.485 38](javascript:showCorrelationPlot2(db='MA_M2M_0706_R',ProbeSetID='1431015_at',CellID='',db2='MA_M2M_0706_R',ProbeSetID2='1448943_at',CellID2='',rank='0')) | [0.547 38](javascript:showCorrelationPlot2(db='MA_M2M_0706_R',ProbeSetID='1431015_at',CellID='',db2='MA_M2M_0706_R',ProbeSetID2='1433162_at',CellID2='',rank='0')) | [0.631 38](javascript:showCorrelationPlot2(db='MA_M2M_0706_R',ProbeSetID='1431015_at',CellID='',db2='MA_M2M_0706_R',ProbeSetID2='1443494_at',CellID2='',rank='0')) | [-0.417 38](javascript:showCorrelationPlot2(db='MA_M2M_0706_R',ProbeSetID='1431015_at',CellID='',db2='MA_M2M_0706_R',ProbeSetID2='1417426_at',CellID2='',rank='0')) | [0.607 38](javascript:showCorrelationPlot2(db='MA_M2M_0706_R',ProbeSetID='1431015_at',CellID='',db2='MA_M2M_0706_R',ProbeSetID2='1416588_at',CellID2='',rank='0')) | [-0.516 38](javascript:showCorrelationPlot2(db='MA_M2M_0706_R',ProbeSetID='1431015_at',CellID='',db2='MA_M2M_0706_R',ProbeSetID2='1450994_at',CellID2='',rank='0')) | [0.631 38](javascript:showCorrelationPlot2(db='MA_M2M_0706_R',ProbeSetID='1431015_at',CellID='',db2='MA_M2M_0706_R',ProbeSetID2='1420304_x_at',CellID2='',rank='0')) | [-0.645 38](javascript:showCorrelationPlot2(db='MA_M2M_0706_R',ProbeSetID='1431015_at',CellID='',db2='MA_M2M_0706_R',ProbeSetID2='1416500_at',CellID2='',rank='0')) | [0.587 38](javascript:showCorrelationPlot2(db='MA_M2M_0706_R',ProbeSetID='1431015_at',CellID='',db2='MA_M2M_0706_R',ProbeSetID2='1424199_at',CellID2='',rank='0')) | [-0.531 38](javascript:showCorrelationPlot2(db='MA_M2M_0706_R',ProbeSetID='1431015_at',CellID='',db2='MA_M2M_0706_R',ProbeSetID2='1437995_x_at',CellID2='',rank='0')) | [-0.599 38](javascript:showCorrelationPlot2(db='MA_M2M_0706_R',ProbeSetID='1431015_at',CellID='',db2='MA_M2M_0706_R',ProbeSetID2='1427914_a_at',CellID2='',rank='0')) | [0.584 38](javascript:showCorrelationPlot2(db='MA_M2M_0706_R',ProbeSetID='1431015_at',CellID='',db2='MA_M2M_0706_R',ProbeSetID2='1420570_x_at',CellID2='',rank='0')) | [n 38](javascript:showDatabase2('MA_M2M_0706_R','1431015_at','')) | [-0.413 38](javascript:showCorrelationPlot2(db='MA_M2M_0706_R',ProbeSetID='1431015_at',CellID='',db2='MA_M2M_0706_R',ProbeSetID2='1423852_at',CellID2='',rank='1')) | [0.408 38](javascript:showCorrelationPlot2(db='MA_M2M_0706_R',ProbeSetID='1431015_at',CellID='',db2='MA_M2M_0706_R',ProbeSetID2='1431799_at',CellID2='',rank='1')) | [-0.526 38](javascript:showCorrelationPlot2(db='MA_M2M_0706_R',ProbeSetID='1431015_at',CellID='',db2='MA_M2M_0706_R',ProbeSetID2='1423898_a_at',CellID2='',rank='1')) | [0.488 38](javascript:showCorrelationPlot2(db='MA_M2M_0706_R',ProbeSetID='1431015_at',CellID='',db2='MA_M2M_0706_R',ProbeSetID2='1438579_at',CellID2='',rank='1')) | [-0.675 38](javascript:showCorrelationPlot2(db='MA_M2M_0706_R',ProbeSetID='1431015_at',CellID='',db2='MA_M2M_0706_R',ProbeSetID2='1448102_a_at',CellID2='',rank='1')) | [0.439 38](javascript:showCorrelationPlot2(db='MA_M2M_0706_R',ProbeSetID='1431015_at',CellID='',db2='MA_M2M_0706_R',ProbeSetID2='1420943_at',CellID2='',rank='1')) | [0.534 38](javascript:showCorrelationPlot2(db='MA_M2M_0706_R',ProbeSetID='1431015_at',CellID='',db2='MA_M2M_0706_R',ProbeSetID2='1447326_s_at',CellID2='',rank='1')) |
|  | [Trait 44: MA_M2M_0706_R::1423852_at](javascript:showDatabase2('MA_M2M_0706_R','1423852_at','');)  Tmem46 on Chr 14 @ 60.249949 Mb  transmembrane protein 46 | [0.597 38](javascript:showCorrelationPlot2(db='MA_M2M_0706_R',ProbeSetID='1423852_at',CellID='',db2='MA_M2M_0706_R',ProbeSetID2='1445101_at',CellID2='',rank='0')) | [-0.464 38](javascript:showCorrelationPlot2(db='MA_M2M_0706_R',ProbeSetID='1423852_at',CellID='',db2='MA_M2M_0706_R',ProbeSetID2='1451285_at',CellID2='',rank='0')) | [-0.532 38](javascript:showCorrelationPlot2(db='MA_M2M_0706_R',ProbeSetID='1423852_at',CellID='',db2='MA_M2M_0706_R',ProbeSetID2='1441347_at',CellID2='',rank='0')) | [0.182 38](javascript:showCorrelationPlot2(db='MA_M2M_0706_R',ProbeSetID='1423852_at',CellID='',db2='MA_M2M_0706_R',ProbeSetID2='1426900_at',CellID2='',rank='0')) | [0.307 38](javascript:showCorrelationPlot2(db='MA_M2M_0706_R',ProbeSetID='1423852_at',CellID='',db2='MA_M2M_0706_R',ProbeSetID2='1434888_a_at',CellID2='',rank='0')) | [0.547 38](javascript:showCorrelationPlot2(db='MA_M2M_0706_R',ProbeSetID='1423852_at',CellID='',db2='MA_M2M_0706_R',ProbeSetID2='1434773_a_at',CellID2='',rank='0')) | [-0.571 38](javascript:showCorrelationPlot2(db='MA_M2M_0706_R',ProbeSetID='1423852_at',CellID='',db2='MA_M2M_0706_R',ProbeSetID2='1452851_at',CellID2='',rank='0')) | [0.537 38](javascript:showCorrelationPlot2(db='MA_M2M_0706_R',ProbeSetID='1423852_at',CellID='',db2='MA_M2M_0706_R',ProbeSetID2='1441948_x_at',CellID2='',rank='0')) | [-0.566 38](javascript:showCorrelationPlot2(db='MA_M2M_0706_R',ProbeSetID='1423852_at',CellID='',db2='MA_M2M_0706_R',ProbeSetID2='1440160_x_at',CellID2='',rank='0')) | [0.535 38](javascript:showCorrelationPlot2(db='MA_M2M_0706_R',ProbeSetID='1423852_at',CellID='',db2='MA_M2M_0706_R',ProbeSetID2='1424692_at',CellID2='',rank='0')) | [-0.690 38](javascript:showCorrelationPlot2(db='MA_M2M_0706_R',ProbeSetID='1423852_at',CellID='',db2='MA_M2M_0706_R',ProbeSetID2='1433112_at',CellID2='',rank='0')) | [0.538 38](javascript:showCorrelationPlot2(db='MA_M2M_0706_R',ProbeSetID='1423852_at',CellID='',db2='MA_M2M_0706_R',ProbeSetID2='1459962_at',CellID2='',rank='0')) | [-0.616 38](javascript:showCorrelationPlot2(db='MA_M2M_0706_R',ProbeSetID='1423852_at',CellID='',db2='MA_M2M_0706_R',ProbeSetID2='1455331_at',CellID2='',rank='0')) | [-0.693 38](javascript:showCorrelationPlot2(db='MA_M2M_0706_R',ProbeSetID='1423852_at',CellID='',db2='MA_M2M_0706_R',ProbeSetID2='1447399_at',CellID2='',rank='0')) | [0.437 38](javascript:showCorrelationPlot2(db='MA_M2M_0706_R',ProbeSetID='1423852_at',CellID='',db2='MA_M2M_0706_R',ProbeSetID2='1434968_a_at',CellID2='',rank='0')) | [-0.578 38](javascript:showCorrelationPlot2(db='MA_M2M_0706_R',ProbeSetID='1423852_at',CellID='',db2='MA_M2M_0706_R',ProbeSetID2='1447335_x_at',CellID2='',rank='0')) | [-0.578 38](javascript:showCorrelationPlot2(db='MA_M2M_0706_R',ProbeSetID='1423852_at',CellID='',db2='MA_M2M_0706_R',ProbeSetID2='1442239_at',CellID2='',rank='0')) | [-0.399 38](javascript:showCorrelationPlot2(db='MA_M2M_0706_R',ProbeSetID='1423852_at',CellID='',db2='MA_M2M_0706_R',ProbeSetID2='1459865_x_at',CellID2='',rank='0')) | [0.370 38](javascript:showCorrelationPlot2(db='MA_M2M_0706_R',ProbeSetID='1423852_at',CellID='',db2='MA_M2M_0706_R',ProbeSetID2='1438647_x_at',CellID2='',rank='0')) | [-0.564 38](javascript:showCorrelationPlot2(db='MA_M2M_0706_R',ProbeSetID='1423852_at',CellID='',db2='MA_M2M_0706_R',ProbeSetID2='1432333_a_at',CellID2='',rank='0')) | [0.476 38](javascript:showCorrelationPlot2(db='MA_M2M_0706_R',ProbeSetID='1423852_at',CellID='',db2='MA_M2M_0706_R',ProbeSetID2='1444028_s_at',CellID2='',rank='0')) | [-0.656 38](javascript:showCorrelationPlot2(db='MA_M2M_0706_R',ProbeSetID='1423852_at',CellID='',db2='MA_M2M_0706_R',ProbeSetID2='1460420_a_at',CellID2='',rank='0')) | [-0.507 38](javascript:showCorrelationPlot2(db='MA_M2M_0706_R',ProbeSetID='1423852_at',CellID='',db2='MA_M2M_0706_R',ProbeSetID2='1451530_at',CellID2='',rank='0')) | [0.413 38](javascript:showCorrelationPlot2(db='MA_M2M_0706_R',ProbeSetID='1423852_at',CellID='',db2='MA_M2M_0706_R',ProbeSetID2='1423785_at',CellID2='',rank='0')) | [-0.364 38](javascript:showCorrelationPlot2(db='MA_M2M_0706_R',ProbeSetID='1423852_at',CellID='',db2='MA_M2M_0706_R',ProbeSetID2='1431972_a_at',CellID2='',rank='0')) | [0.385 38](javascript:showCorrelationPlot2(db='MA_M2M_0706_R',ProbeSetID='1423852_at',CellID='',db2='MA_M2M_0706_R',ProbeSetID2='1448347_a_at',CellID2='',rank='0')) | [-0.398 38](javascript:showCorrelationPlot2(db='MA_M2M_0706_R',ProbeSetID='1423852_at',CellID='',db2='MA_M2M_0706_R',ProbeSetID2='1418492_at',CellID2='',rank='0')) | [0.490 38](javascript:showCorrelationPlot2(db='MA_M2M_0706_R',ProbeSetID='1423852_at',CellID='',db2='MA_M2M_0706_R',ProbeSetID2='1427185_at',CellID2='',rank='0')) | [0.367 38](javascript:showCorrelationPlot2(db='MA_M2M_0706_R',ProbeSetID='1423852_at',CellID='',db2='MA_M2M_0706_R',ProbeSetID2='1430500_s_at',CellID2='',rank='0')) | [-0.432 38](javascript:showCorrelationPlot2(db='MA_M2M_0706_R',ProbeSetID='1423852_at',CellID='',db2='MA_M2M_0706_R',ProbeSetID2='1447549_x_at',CellID2='',rank='0')) | [0.349 38](javascript:showCorrelationPlot2(db='MA_M2M_0706_R',ProbeSetID='1423852_at',CellID='',db2='MA_M2M_0706_R',ProbeSetID2='1448943_at',CellID2='',rank='0')) | [-0.642 38](javascript:showCorrelationPlot2(db='MA_M2M_0706_R',ProbeSetID='1423852_at',CellID='',db2='MA_M2M_0706_R',ProbeSetID2='1433162_at',CellID2='',rank='0')) | [-0.395 38](javascript:showCorrelationPlot2(db='MA_M2M_0706_R',ProbeSetID='1423852_at',CellID='',db2='MA_M2M_0706_R',ProbeSetID2='1443494_at',CellID2='',rank='0')) | [0.364 38](javascript:showCorrelationPlot2(db='MA_M2M_0706_R',ProbeSetID='1423852_at',CellID='',db2='MA_M2M_0706_R',ProbeSetID2='1417426_at',CellID2='',rank='0')) | [-0.625 38](javascript:showCorrelationPlot2(db='MA_M2M_0706_R',ProbeSetID='1423852_at',CellID='',db2='MA_M2M_0706_R',ProbeSetID2='1416588_at',CellID2='',rank='0')) | [0.376 38](javascript:showCorrelationPlot2(db='MA_M2M_0706_R',ProbeSetID='1423852_at',CellID='',db2='MA_M2M_0706_R',ProbeSetID2='1450994_at',CellID2='',rank='0')) | [-0.641 38](javascript:showCorrelationPlot2(db='MA_M2M_0706_R',ProbeSetID='1423852_at',CellID='',db2='MA_M2M_0706_R',ProbeSetID2='1420304_x_at',CellID2='',rank='0')) | [0.335 38](javascript:showCorrelationPlot2(db='MA_M2M_0706_R',ProbeSetID='1423852_at',CellID='',db2='MA_M2M_0706_R',ProbeSetID2='1416500_at',CellID2='',rank='0')) | [-0.554 38](javascript:showCorrelationPlot2(db='MA_M2M_0706_R',ProbeSetID='1423852_at',CellID='',db2='MA_M2M_0706_R',ProbeSetID2='1424199_at',CellID2='',rank='0')) | [0.228 38](javascript:showCorrelationPlot2(db='MA_M2M_0706_R',ProbeSetID='1423852_at',CellID='',db2='MA_M2M_0706_R',ProbeSetID2='1437995_x_at',CellID2='',rank='0')) | [0.368 38](javascript:showCorrelationPlot2(db='MA_M2M_0706_R',ProbeSetID='1423852_at',CellID='',db2='MA_M2M_0706_R',ProbeSetID2='1427914_a_at',CellID2='',rank='0')) | [-0.634 38](javascript:showCorrelationPlot2(db='MA_M2M_0706_R',ProbeSetID='1423852_at',CellID='',db2='MA_M2M_0706_R',ProbeSetID2='1420570_x_at',CellID2='',rank='0')) | [-0.347 38](javascript:showCorrelationPlot2(db='MA_M2M_0706_R',ProbeSetID='1423852_at',CellID='',db2='MA_M2M_0706_R',ProbeSetID2='1431015_at',CellID2='',rank='0')) | [n 38](javascript:showDatabase2('MA_M2M_0706_R','1423852_at','')) | [-0.403 38](javascript:showCorrelationPlot2(db='MA_M2M_0706_R',ProbeSetID='1423852_at',CellID='',db2='MA_M2M_0706_R',ProbeSetID2='1431799_at',CellID2='',rank='1')) | [0.295 38](javascript:showCorrelationPlot2(db='MA_M2M_0706_R',ProbeSetID='1423852_at',CellID='',db2='MA_M2M_0706_R',ProbeSetID2='1423898_a_at',CellID2='',rank='1')) | [-0.582 38](javascript:showCorrelationPlot2(db='MA_M2M_0706_R',ProbeSetID='1423852_at',CellID='',db2='MA_M2M_0706_R',ProbeSetID2='1438579_at',CellID2='',rank='1')) | [0.516 38](javascript:showCorrelationPlot2(db='MA_M2M_0706_R',ProbeSetID='1423852_at',CellID='',db2='MA_M2M_0706_R',ProbeSetID2='1448102_a_at',CellID2='',rank='1')) | [-0.626 38](javascript:showCorrelationPlot2(db='MA_M2M_0706_R',ProbeSetID='1423852_at',CellID='',db2='MA_M2M_0706_R',ProbeSetID2='1420943_at',CellID2='',rank='1')) | [-0.615 38](javascript:showCorrelationPlot2(db='MA_M2M_0706_R',ProbeSetID='1423852_at',CellID='',db2='MA_M2M_0706_R',ProbeSetID2='1447326_s_at',CellID2='',rank='1')) |
|  | [Trait 45: MA_M2M_0706_R::1431799_at](javascript:showDatabase2('MA_M2M_0706_R','1431799_at','');)  Trim68 on Chr 7 @ 109.837232 Mb  tripartite motif containing 68 | [-0.285 38](javascript:showCorrelationPlot2(db='MA_M2M_0706_R',ProbeSetID='1431799_at',CellID='',db2='MA_M2M_0706_R',ProbeSetID2='1445101_at',CellID2='',rank='0')) | [0.278 38](javascript:showCorrelationPlot2(db='MA_M2M_0706_R',ProbeSetID='1431799_at',CellID='',db2='MA_M2M_0706_R',ProbeSetID2='1451285_at',CellID2='',rank='0')) | [0.146 38](javascript:showCorrelationPlot2(db='MA_M2M_0706_R',ProbeSetID='1431799_at',CellID='',db2='MA_M2M_0706_R',ProbeSetID2='1441347_at',CellID2='',rank='0')) | [-0.338 38](javascript:showCorrelationPlot2(db='MA_M2M_0706_R',ProbeSetID='1431799_at',CellID='',db2='MA_M2M_0706_R',ProbeSetID2='1426900_at',CellID2='',rank='0')) | [-0.473 38](javascript:showCorrelationPlot2(db='MA_M2M_0706_R',ProbeSetID='1431799_at',CellID='',db2='MA_M2M_0706_R',ProbeSetID2='1434888_a_at',CellID2='',rank='0')) | [-0.524 38](javascript:showCorrelationPlot2(db='MA_M2M_0706_R',ProbeSetID='1431799_at',CellID='',db2='MA_M2M_0706_R',ProbeSetID2='1434773_a_at',CellID2='',rank='0')) | [0.361 38](javascript:showCorrelationPlot2(db='MA_M2M_0706_R',ProbeSetID='1431799_at',CellID='',db2='MA_M2M_0706_R',ProbeSetID2='1452851_at',CellID2='',rank='0')) | [-0.243 38](javascript:showCorrelationPlot2(db='MA_M2M_0706_R',ProbeSetID='1431799_at',CellID='',db2='MA_M2M_0706_R',ProbeSetID2='1441948_x_at',CellID2='',rank='0')) | [0.317 38](javascript:showCorrelationPlot2(db='MA_M2M_0706_R',ProbeSetID='1431799_at',CellID='',db2='MA_M2M_0706_R',ProbeSetID2='1440160_x_at',CellID2='',rank='0')) | [-0.422 38](javascript:showCorrelationPlot2(db='MA_M2M_0706_R',ProbeSetID='1431799_at',CellID='',db2='MA_M2M_0706_R',ProbeSetID2='1424692_at',CellID2='',rank='0')) | [0.308 38](javascript:showCorrelationPlot2(db='MA_M2M_0706_R',ProbeSetID='1431799_at',CellID='',db2='MA_M2M_0706_R',ProbeSetID2='1433112_at',CellID2='',rank='0')) | [-0.404 38](javascript:showCorrelationPlot2(db='MA_M2M_0706_R',ProbeSetID='1431799_at',CellID='',db2='MA_M2M_0706_R',ProbeSetID2='1459962_at',CellID2='',rank='0')) | [0.168 38](javascript:showCorrelationPlot2(db='MA_M2M_0706_R',ProbeSetID='1431799_at',CellID='',db2='MA_M2M_0706_R',ProbeSetID2='1455331_at',CellID2='',rank='0')) | [0.294 38](javascript:showCorrelationPlot2(db='MA_M2M_0706_R',ProbeSetID='1431799_at',CellID='',db2='MA_M2M_0706_R',ProbeSetID2='1447399_at',CellID2='',rank='0')) | [-0.377 38](javascript:showCorrelationPlot2(db='MA_M2M_0706_R',ProbeSetID='1431799_at',CellID='',db2='MA_M2M_0706_R',ProbeSetID2='1434968_a_at',CellID2='',rank='0')) | [0.352 38](javascript:showCorrelationPlot2(db='MA_M2M_0706_R',ProbeSetID='1431799_at',CellID='',db2='MA_M2M_0706_R',ProbeSetID2='1447335_x_at',CellID2='',rank='0')) | [0.337 38](javascript:showCorrelationPlot2(db='MA_M2M_0706_R',ProbeSetID='1431799_at',CellID='',db2='MA_M2M_0706_R',ProbeSetID2='1442239_at',CellID2='',rank='0')) | [0.683 38](javascript:showCorrelationPlot2(db='MA_M2M_0706_R',ProbeSetID='1431799_at',CellID='',db2='MA_M2M_0706_R',ProbeSetID2='1459865_x_at',CellID2='',rank='0')) | [-0.485 38](javascript:showCorrelationPlot2(db='MA_M2M_0706_R',ProbeSetID='1431799_at',CellID='',db2='MA_M2M_0706_R',ProbeSetID2='1438647_x_at',CellID2='',rank='0')) | [0.369 38](javascript:showCorrelationPlot2(db='MA_M2M_0706_R',ProbeSetID='1431799_at',CellID='',db2='MA_M2M_0706_R',ProbeSetID2='1432333_a_at',CellID2='',rank='0')) | [-0.589 38](javascript:showCorrelationPlot2(db='MA_M2M_0706_R',ProbeSetID='1431799_at',CellID='',db2='MA_M2M_0706_R',ProbeSetID2='1444028_s_at',CellID2='',rank='0')) | [0.667 38](javascript:showCorrelationPlot2(db='MA_M2M_0706_R',ProbeSetID='1431799_at',CellID='',db2='MA_M2M_0706_R',ProbeSetID2='1460420_a_at',CellID2='',rank='0')) | [0.276 38](javascript:showCorrelationPlot2(db='MA_M2M_0706_R',ProbeSetID='1431799_at',CellID='',db2='MA_M2M_0706_R',ProbeSetID2='1451530_at',CellID2='',rank='0')) | [-0.261 38](javascript:showCorrelationPlot2(db='MA_M2M_0706_R',ProbeSetID='1431799_at',CellID='',db2='MA_M2M_0706_R',ProbeSetID2='1423785_at',CellID2='',rank='0')) | [0.515 38](javascript:showCorrelationPlot2(db='MA_M2M_0706_R',ProbeSetID='1431799_at',CellID='',db2='MA_M2M_0706_R',ProbeSetID2='1431972_a_at',CellID2='',rank='0')) | [-0.432 38](javascript:showCorrelationPlot2(db='MA_M2M_0706_R',ProbeSetID='1431799_at',CellID='',db2='MA_M2M_0706_R',ProbeSetID2='1448347_a_at',CellID2='',rank='0')) | [0.294 38](javascript:showCorrelationPlot2(db='MA_M2M_0706_R',ProbeSetID='1431799_at',CellID='',db2='MA_M2M_0706_R',ProbeSetID2='1418492_at',CellID2='',rank='0')) | [-0.393 38](javascript:showCorrelationPlot2(db='MA_M2M_0706_R',ProbeSetID='1431799_at',CellID='',db2='MA_M2M_0706_R',ProbeSetID2='1427185_at',CellID2='',rank='0')) | [-0.396 38](javascript:showCorrelationPlot2(db='MA_M2M_0706_R',ProbeSetID='1431799_at',CellID='',db2='MA_M2M_0706_R',ProbeSetID2='1430500_s_at',CellID2='',rank='0')) | [0.318 38](javascript:showCorrelationPlot2(db='MA_M2M_0706_R',ProbeSetID='1431799_at',CellID='',db2='MA_M2M_0706_R',ProbeSetID2='1447549_x_at',CellID2='',rank='0')) | [-0.510 38](javascript:showCorrelationPlot2(db='MA_M2M_0706_R',ProbeSetID='1431799_at',CellID='',db2='MA_M2M_0706_R',ProbeSetID2='1448943_at',CellID2='',rank='0')) | [0.338 38](javascript:showCorrelationPlot2(db='MA_M2M_0706_R',ProbeSetID='1431799_at',CellID='',db2='MA_M2M_0706_R',ProbeSetID2='1433162_at',CellID2='',rank='0')) | [0.443 38](javascript:showCorrelationPlot2(db='MA_M2M_0706_R',ProbeSetID='1431799_at',CellID='',db2='MA_M2M_0706_R',ProbeSetID2='1443494_at',CellID2='',rank='0')) | [-0.535 38](javascript:showCorrelationPlot2(db='MA_M2M_0706_R',ProbeSetID='1431799_at',CellID='',db2='MA_M2M_0706_R',ProbeSetID2='1417426_at',CellID2='',rank='0')) | [0.366 38](javascript:showCorrelationPlot2(db='MA_M2M_0706_R',ProbeSetID='1431799_at',CellID='',db2='MA_M2M_0706_R',ProbeSetID2='1416588_at',CellID2='',rank='0')) | [-0.609 38](javascript:showCorrelationPlot2(db='MA_M2M_0706_R',ProbeSetID='1431799_at',CellID='',db2='MA_M2M_0706_R',ProbeSetID2='1450994_at',CellID2='',rank='0')) | [0.369 38](javascript:showCorrelationPlot2(db='MA_M2M_0706_R',ProbeSetID='1431799_at',CellID='',db2='MA_M2M_0706_R',ProbeSetID2='1420304_x_at',CellID2='',rank='0')) | [-0.343 38](javascript:showCorrelationPlot2(db='MA_M2M_0706_R',ProbeSetID='1431799_at',CellID='',db2='MA_M2M_0706_R',ProbeSetID2='1416500_at',CellID2='',rank='0')) | [0.311 38](javascript:showCorrelationPlot2(db='MA_M2M_0706_R',ProbeSetID='1431799_at',CellID='',db2='MA_M2M_0706_R',ProbeSetID2='1424199_at',CellID2='',rank='0')) | [-0.425 38](javascript:showCorrelationPlot2(db='MA_M2M_0706_R',ProbeSetID='1431799_at',CellID='',db2='MA_M2M_0706_R',ProbeSetID2='1437995_x_at',CellID2='',rank='0')) | [-0.443 38](javascript:showCorrelationPlot2(db='MA_M2M_0706_R',ProbeSetID='1431799_at',CellID='',db2='MA_M2M_0706_R',ProbeSetID2='1427914_a_at',CellID2='',rank='0')) | [0.317 38](javascript:showCorrelationPlot2(db='MA_M2M_0706_R',ProbeSetID='1431799_at',CellID='',db2='MA_M2M_0706_R',ProbeSetID2='1420570_x_at',CellID2='',rank='0')) | [0.448 38](javascript:showCorrelationPlot2(db='MA_M2M_0706_R',ProbeSetID='1431799_at',CellID='',db2='MA_M2M_0706_R',ProbeSetID2='1431015_at',CellID2='',rank='0')) | [-0.353 38](javascript:showCorrelationPlot2(db='MA_M2M_0706_R',ProbeSetID='1431799_at',CellID='',db2='MA_M2M_0706_R',ProbeSetID2='1423852_at',CellID2='',rank='0')) | [n 38](javascript:showDatabase2('MA_M2M_0706_R','1431799_at','')) | [-0.425 38](javascript:showCorrelationPlot2(db='MA_M2M_0706_R',ProbeSetID='1431799_at',CellID='',db2='MA_M2M_0706_R',ProbeSetID2='1423898_a_at',CellID2='',rank='1')) | [0.248 38](javascript:showCorrelationPlot2(db='MA_M2M_0706_R',ProbeSetID='1431799_at',CellID='',db2='MA_M2M_0706_R',ProbeSetID2='1438579_at',CellID2='',rank='1')) | [-0.362 38](javascript:showCorrelationPlot2(db='MA_M2M_0706_R',ProbeSetID='1431799_at',CellID='',db2='MA_M2M_0706_R',ProbeSetID2='1448102_a_at',CellID2='',rank='1')) | [0.252 38](javascript:showCorrelationPlot2(db='MA_M2M_0706_R',ProbeSetID='1431799_at',CellID='',db2='MA_M2M_0706_R',ProbeSetID2='1420943_at',CellID2='',rank='1')) | [0.537 38](javascript:showCorrelationPlot2(db='MA_M2M_0706_R',ProbeSetID='1431799_at',CellID='',db2='MA_M2M_0706_R',ProbeSetID2='1447326_s_at',CellID2='',rank='1')) |
|  | [Trait 46: MA_M2M_0706_R::1423898_a_at](javascript:showDatabase2('MA_M2M_0706_R','1423898_a_at','');)  Trip12 on Chr 1 @ 84.721014 Mb  thyroid hormone receptor interactor 12 | [0.583 38](javascript:showCorrelationPlot2(db='MA_M2M_0706_R',ProbeSetID='1423898_a_at',CellID='',db2='MA_M2M_0706_R',ProbeSetID2='1445101_at',CellID2='',rank='0')) | [-0.520 38](javascript:showCorrelationPlot2(db='MA_M2M_0706_R',ProbeSetID='1423898_a_at',CellID='',db2='MA_M2M_0706_R',ProbeSetID2='1451285_at',CellID2='',rank='0')) | [-0.444 38](javascript:showCorrelationPlot2(db='MA_M2M_0706_R',ProbeSetID='1423898_a_at',CellID='',db2='MA_M2M_0706_R',ProbeSetID2='1441347_at',CellID2='',rank='0')) | [0.646 38](javascript:showCorrelationPlot2(db='MA_M2M_0706_R',ProbeSetID='1423898_a_at',CellID='',db2='MA_M2M_0706_R',ProbeSetID2='1426900_at',CellID2='',rank='0')) | [0.665 38](javascript:showCorrelationPlot2(db='MA_M2M_0706_R',ProbeSetID='1423898_a_at',CellID='',db2='MA_M2M_0706_R',ProbeSetID2='1434888_a_at',CellID2='',rank='0')) | [0.668 38](javascript:showCorrelationPlot2(db='MA_M2M_0706_R',ProbeSetID='1423898_a_at',CellID='',db2='MA_M2M_0706_R',ProbeSetID2='1434773_a_at',CellID2='',rank='0')) | [-0.554 38](javascript:showCorrelationPlot2(db='MA_M2M_0706_R',ProbeSetID='1423898_a_at',CellID='',db2='MA_M2M_0706_R',ProbeSetID2='1452851_at',CellID2='',rank='0')) | [0.619 38](javascript:showCorrelationPlot2(db='MA_M2M_0706_R',ProbeSetID='1423898_a_at',CellID='',db2='MA_M2M_0706_R',ProbeSetID2='1441948_x_at',CellID2='',rank='0')) | [-0.586 38](javascript:showCorrelationPlot2(db='MA_M2M_0706_R',ProbeSetID='1423898_a_at',CellID='',db2='MA_M2M_0706_R',ProbeSetID2='1440160_x_at',CellID2='',rank='0')) | [0.615 38](javascript:showCorrelationPlot2(db='MA_M2M_0706_R',ProbeSetID='1423898_a_at',CellID='',db2='MA_M2M_0706_R',ProbeSetID2='1424692_at',CellID2='',rank='0')) | [-0.573 38](javascript:showCorrelationPlot2(db='MA_M2M_0706_R',ProbeSetID='1423898_a_at',CellID='',db2='MA_M2M_0706_R',ProbeSetID2='1433112_at',CellID2='',rank='0')) | [0.543 38](javascript:showCorrelationPlot2(db='MA_M2M_0706_R',ProbeSetID='1423898_a_at',CellID='',db2='MA_M2M_0706_R',ProbeSetID2='1459962_at',CellID2='',rank='0')) | [-0.430 38](javascript:showCorrelationPlot2(db='MA_M2M_0706_R',ProbeSetID='1423898_a_at',CellID='',db2='MA_M2M_0706_R',ProbeSetID2='1455331_at',CellID2='',rank='0')) | [-0.466 38](javascript:showCorrelationPlot2(db='MA_M2M_0706_R',ProbeSetID='1423898_a_at',CellID='',db2='MA_M2M_0706_R',ProbeSetID2='1447399_at',CellID2='',rank='0')) | [0.469 38](javascript:showCorrelationPlot2(db='MA_M2M_0706_R',ProbeSetID='1423898_a_at',CellID='',db2='MA_M2M_0706_R',ProbeSetID2='1434968_a_at',CellID2='',rank='0')) | [-0.513 38](javascript:showCorrelationPlot2(db='MA_M2M_0706_R',ProbeSetID='1423898_a_at',CellID='',db2='MA_M2M_0706_R',ProbeSetID2='1447335_x_at',CellID2='',rank='0')) | [-0.553 38](javascript:showCorrelationPlot2(db='MA_M2M_0706_R',ProbeSetID='1423898_a_at',CellID='',db2='MA_M2M_0706_R',ProbeSetID2='1442239_at',CellID2='',rank='0')) | [-0.621 38](javascript:showCorrelationPlot2(db='MA_M2M_0706_R',ProbeSetID='1423898_a_at',CellID='',db2='MA_M2M_0706_R',ProbeSetID2='1459865_x_at',CellID2='',rank='0')) | [0.592 38](javascript:showCorrelationPlot2(db='MA_M2M_0706_R',ProbeSetID='1423898_a_at',CellID='',db2='MA_M2M_0706_R',ProbeSetID2='1438647_x_at',CellID2='',rank='0')) | [-0.614 38](javascript:showCorrelationPlot2(db='MA_M2M_0706_R',ProbeSetID='1423898_a_at',CellID='',db2='MA_M2M_0706_R',ProbeSetID2='1432333_a_at',CellID2='',rank='0')) | [0.464 38](javascript:showCorrelationPlot2(db='MA_M2M_0706_R',ProbeSetID='1423898_a_at',CellID='',db2='MA_M2M_0706_R',ProbeSetID2='1444028_s_at',CellID2='',rank='0')) | [-0.591 38](javascript:showCorrelationPlot2(db='MA_M2M_0706_R',ProbeSetID='1423898_a_at',CellID='',db2='MA_M2M_0706_R',ProbeSetID2='1460420_a_at',CellID2='',rank='0')) | [-0.539 38](javascript:showCorrelationPlot2(db='MA_M2M_0706_R',ProbeSetID='1423898_a_at',CellID='',db2='MA_M2M_0706_R',ProbeSetID2='1451530_at',CellID2='',rank='0')) | [0.576 38](javascript:showCorrelationPlot2(db='MA_M2M_0706_R',ProbeSetID='1423898_a_at',CellID='',db2='MA_M2M_0706_R',ProbeSetID2='1423785_at',CellID2='',rank='0')) | [-0.531 38](javascript:showCorrelationPlot2(db='MA_M2M_0706_R',ProbeSetID='1423898_a_at',CellID='',db2='MA_M2M_0706_R',ProbeSetID2='1431972_a_at',CellID2='',rank='0')) | [0.759 38](javascript:showCorrelationPlot2(db='MA_M2M_0706_R',ProbeSetID='1423898_a_at',CellID='',db2='MA_M2M_0706_R',ProbeSetID2='1448347_a_at',CellID2='',rank='0')) | [-0.419 38](javascript:showCorrelationPlot2(db='MA_M2M_0706_R',ProbeSetID='1423898_a_at',CellID='',db2='MA_M2M_0706_R',ProbeSetID2='1418492_at',CellID2='',rank='0')) | [0.406 38](javascript:showCorrelationPlot2(db='MA_M2M_0706_R',ProbeSetID='1423898_a_at',CellID='',db2='MA_M2M_0706_R',ProbeSetID2='1427185_at',CellID2='',rank='0')) | [0.561 38](javascript:showCorrelationPlot2(db='MA_M2M_0706_R',ProbeSetID='1423898_a_at',CellID='',db2='MA_M2M_0706_R',ProbeSetID2='1430500_s_at',CellID2='',rank='0')) | [-0.341 38](javascript:showCorrelationPlot2(db='MA_M2M_0706_R',ProbeSetID='1423898_a_at',CellID='',db2='MA_M2M_0706_R',ProbeSetID2='1447549_x_at',CellID2='',rank='0')) | [0.447 38](javascript:showCorrelationPlot2(db='MA_M2M_0706_R',ProbeSetID='1423898_a_at',CellID='',db2='MA_M2M_0706_R',ProbeSetID2='1448943_at',CellID2='',rank='0')) | [-0.697 38](javascript:showCorrelationPlot2(db='MA_M2M_0706_R',ProbeSetID='1423898_a_at',CellID='',db2='MA_M2M_0706_R',ProbeSetID2='1433162_at',CellID2='',rank='0')) | [-0.548 38](javascript:showCorrelationPlot2(db='MA_M2M_0706_R',ProbeSetID='1423898_a_at',CellID='',db2='MA_M2M_0706_R',ProbeSetID2='1443494_at',CellID2='',rank='0')) | [0.400 38](javascript:showCorrelationPlot2(db='MA_M2M_0706_R',ProbeSetID='1423898_a_at',CellID='',db2='MA_M2M_0706_R',ProbeSetID2='1417426_at',CellID2='',rank='0')) | [-0.693 38](javascript:showCorrelationPlot2(db='MA_M2M_0706_R',ProbeSetID='1423898_a_at',CellID='',db2='MA_M2M_0706_R',ProbeSetID2='1416588_at',CellID2='',rank='0')) | [0.518 38](javascript:showCorrelationPlot2(db='MA_M2M_0706_R',ProbeSetID='1423898_a_at',CellID='',db2='MA_M2M_0706_R',ProbeSetID2='1450994_at',CellID2='',rank='0')) | [-0.542 38](javascript:showCorrelationPlot2(db='MA_M2M_0706_R',ProbeSetID='1423898_a_at',CellID='',db2='MA_M2M_0706_R',ProbeSetID2='1420304_x_at',CellID2='',rank='0')) | [0.718 38](javascript:showCorrelationPlot2(db='MA_M2M_0706_R',ProbeSetID='1423898_a_at',CellID='',db2='MA_M2M_0706_R',ProbeSetID2='1416500_at',CellID2='',rank='0')) | [-0.701 38](javascript:showCorrelationPlot2(db='MA_M2M_0706_R',ProbeSetID='1423898_a_at',CellID='',db2='MA_M2M_0706_R',ProbeSetID2='1424199_at',CellID2='',rank='0')) | [0.581 38](javascript:showCorrelationPlot2(db='MA_M2M_0706_R',ProbeSetID='1423898_a_at',CellID='',db2='MA_M2M_0706_R',ProbeSetID2='1437995_x_at',CellID2='',rank='0')) | [0.601 38](javascript:showCorrelationPlot2(db='MA_M2M_0706_R',ProbeSetID='1423898_a_at',CellID='',db2='MA_M2M_0706_R',ProbeSetID2='1427914_a_at',CellID2='',rank='0')) | [-0.459 38](javascript:showCorrelationPlot2(db='MA_M2M_0706_R',ProbeSetID='1423898_a_at',CellID='',db2='MA_M2M_0706_R',ProbeSetID2='1420570_x_at',CellID2='',rank='0')) | [-0.577 38](javascript:showCorrelationPlot2(db='MA_M2M_0706_R',ProbeSetID='1423898_a_at',CellID='',db2='MA_M2M_0706_R',ProbeSetID2='1431015_at',CellID2='',rank='0')) | [0.377 38](javascript:showCorrelationPlot2(db='MA_M2M_0706_R',ProbeSetID='1423898_a_at',CellID='',db2='MA_M2M_0706_R',ProbeSetID2='1423852_at',CellID2='',rank='0')) | [-0.418 38](javascript:showCorrelationPlot2(db='MA_M2M_0706_R',ProbeSetID='1423898_a_at',CellID='',db2='MA_M2M_0706_R',ProbeSetID2='1431799_at',CellID2='',rank='0')) | [n 38](javascript:showDatabase2('MA_M2M_0706_R','1423898_a_at','')) | [-0.462 38](javascript:showCorrelationPlot2(db='MA_M2M_0706_R',ProbeSetID='1423898_a_at',CellID='',db2='MA_M2M_0706_R',ProbeSetID2='1438579_at',CellID2='',rank='1')) | [0.554 38](javascript:showCorrelationPlot2(db='MA_M2M_0706_R',ProbeSetID='1423898_a_at',CellID='',db2='MA_M2M_0706_R',ProbeSetID2='1448102_a_at',CellID2='',rank='1')) | [-0.353 38](javascript:showCorrelationPlot2(db='MA_M2M_0706_R',ProbeSetID='1423898_a_at',CellID='',db2='MA_M2M_0706_R',ProbeSetID2='1420943_at',CellID2='',rank='1')) | [-0.481 38](javascript:showCorrelationPlot2(db='MA_M2M_0706_R',ProbeSetID='1423898_a_at',CellID='',db2='MA_M2M_0706_R',ProbeSetID2='1447326_s_at',CellID2='',rank='1')) |
[truncated: 33,820 more chars]
